# Rhodium-catalyzed asymmetric hydroamination and hydroindolation of keto-vinylidenecyclopropanes†
†Electronic supplementary information (ESI) available: Experimental procedures and characterization data of the new compounds. CCDC 1538522, 1525845, and 1822276. For ESI and crystallographic data in CIF or other electronic format see DOI: 10.1039/c8sc01595c


**DOI:** 10.1039/c8sc01595c

**Published:** 2018-05-11

**Authors:** Song Yang, Quan-Zhe Li, Chen Xu, Qin Xu, Min Shi

**Affiliations:** a Key Laboratory for Advanced Materials and Institute of Fine Chemicals , School of Chemistry & Molecular Engineering , East China University of Science and Technology , 130 Meilong Road , Shanghai 200237 , China . Email: mshi@sioc.ac.cn; b State Key Laboratory and Institute of Elemento-organic Chemistry , Nankai University , Tianjin 300071 , P. R. China; c State Key Laboratory of Organometallic Chemistry , Center for Excellence in Molecular Synthesis , University of Chinese Academy of Sciences , Shanghai Institute of Organic Chemistry , Chinese Academy of Sciences , 345 Lingling Road , Shanghai 200032 , China

## Abstract

We reported a highly regio- and enantioselective hydroamination and hydroindolation of keto-vinylidenecyclopropanes *via* cationic Rh(i) catalysis in this context. The combination of various secondary amines and indoles with keto-vinylidenecyclopropanes afforded the corresponding hydrofunctionalization products in good to excellent yields with outstanding ee values under mild conditions. A new TMM–Rh model complex was proposed, providing an atom economical Rh-π-allyl precursor at the same time. Moreover, the resulting products could easily be transformed into more complex polyheterocycles upon further synthetic manipulation.

## Abstract 2

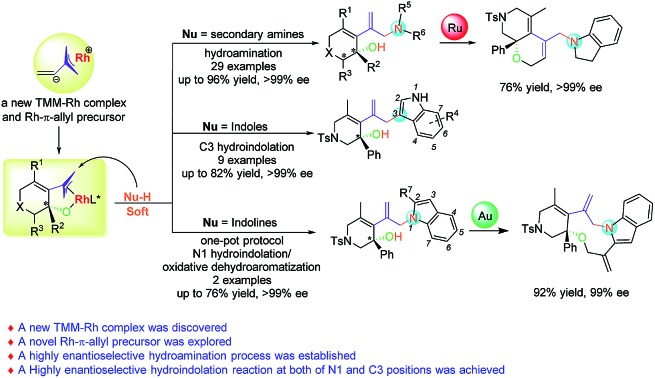
A novel rhodium-catalyzed asymmetric hydroamination and hydroindolation of keto-vinylidenecyclopropanes has been developed, affording the hydroamination and hydroindolation products in good to excellent yields with outstanding ee values through a new TMM–Rh model complex.

## Introduction

**Scheme 1:**
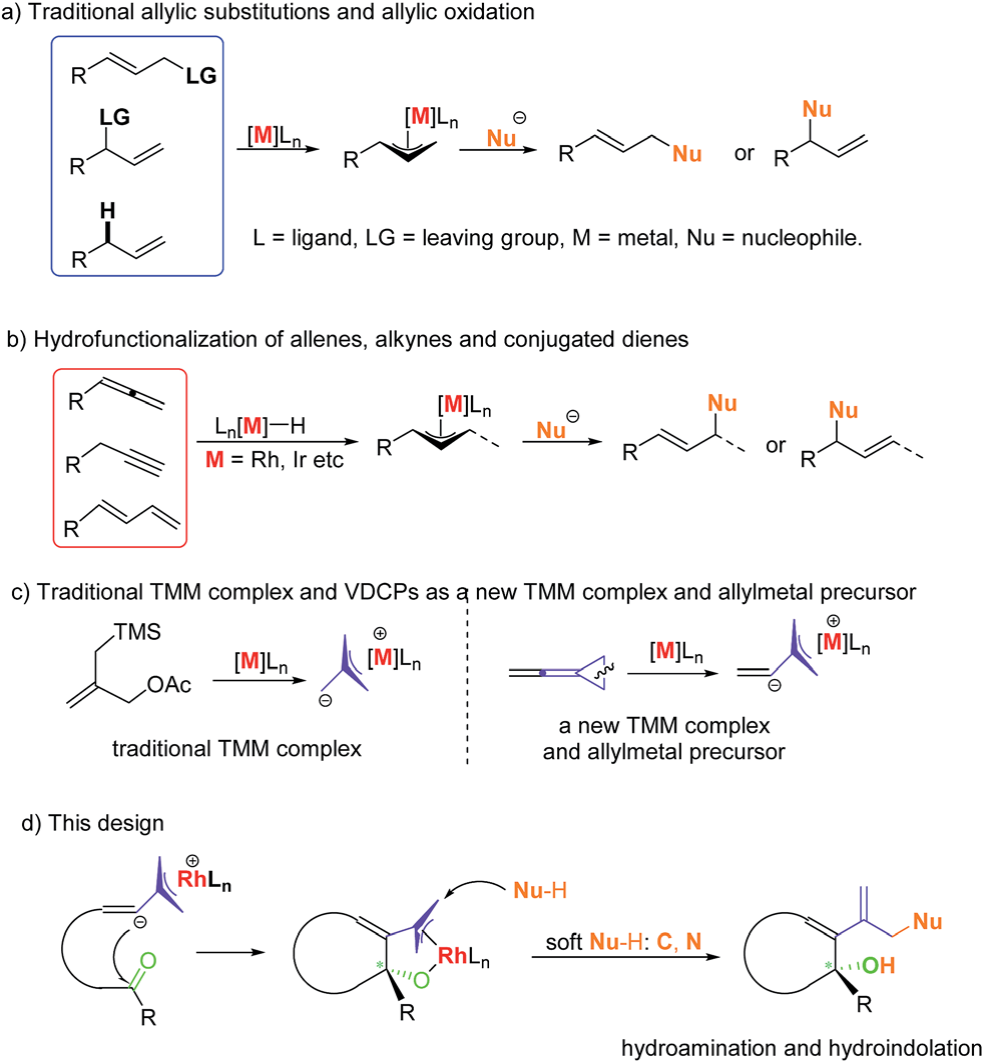
Previous work and this work.

Allylic substitutions^1^,^2^ and allylic oxidations^3^ are powerful synthetic tools for carbon–carbon and carbon–heteroatom bond formation and have a broad range of applications in the synthesis of biologically important molecules (Scheme 1a). Besides, recent studies show that even allylic alcohols can serve as allyl precursors.^4^,^5^ However, these methods suffer from drawbacks such as the required preinstallation of a leaving group or the use of stoichiometric amounts of oxidant, respectively. Thus, the development of new asymmetric carbon–carbon and carbon–heteroatom bond-forming reactions which fulfil the criteria of atom economy is of imminent importance to the evolution of chemical synthesis.^6^ In this respect, the atom economic pathway toward linear allylic products under Pd catalysis was pioneered by Trost and Yamamoto in the late 1990s and early 2000s utilizing mostly terminal allenes or internal Me-substituted alkynes.^7^ More examples using other metals were reported over the following years. Allenes,^8^,^9^ alkynes^10^,^11^ and conjugated dienes^12^,^13^ have been transformed into electrophilic metal-π-allyl intermediates using iridium, rhodium and other transition metal catalysts, which undergo nucleophilic attack to form hydrofunctionalization products (Scheme 1b). This strategy could be regarded as an atom-economic alternative to the traditional metal catalyzed allylic substitution and allylic oxidation.

Vinylidenecyclopropanes (VDCPs), bearing an allene moiety connected to a highly strained cyclopropane ring, serve as fascinating building blocks in organic synthesis and have received great attention from organic chemists.^14^ Based on our ongoing investigation on metal-catalyzed transformations of VDCPs, we found that cationic Rh(i) complexes could insert into the weaker distal bond of the three-membered ring to give new trimethylenemethane (TMM) complexes of rhodium (Scheme 1c).^15^ However, unlike the traditional TMM metal complex, which has been used extensively in [3 + 2] cycloaddition reactions,^16^ this TMM–Rh species containing an inner olefinic moiety could react with an unsaturated functional group and then generate a new more reactive electrophilic Rh-π-allyl intermediate, which might be a productive electrophile for hydrofunctionalization (allylic substitution). Thus, we envisaged that VDCPs could be excellent candidates for the exploration of new reaction modes in the atom economic pathway of allylic substitution with “soft” carbon and heteroatom nucleophiles (those from conjugate acids with a p*K*_a_ less than 25) (Scheme 1d, the design presented in this work).

## Results and discussion

### Experimental investigations

To test the feasibility of our hypothesis, we initially investigated the reaction of keto-VDCP **1a** with indoline **2a** as a coupling partner. Notably, indoline and indole moieties are ubiquitous structural elements of many natural compounds of biological interest.^17^ Therefore, the development of efficient processes for the functionalization of these compounds will facilitate access to pharmaceutically attractive molecules. To our delight, when [Rh(cod)Cl]_2_ and AgNTf_2_ were used as catalysts with (*rac*)-Binap as the ligand, hydroamination product *rac*-**3aa** could be successfully furnished in 75% yield in toluene at 90 °C (Table 1, entry 1). The structure of **3aa** has been unequivocally determined by X-ray diffraction.^18^ Some other nucleophiles were also investigated in this transformation, such as alcohols, thiophenol, benzothiazole and diketones. Disappointedly, only when dibenzoylmethane was used as the nucleophile could the corresponding hydrofunctionalization product be obtained in moderate yield (see Table S1 in the ESI†). Interestingly, 35% yield of *rac*-**3aa** could also be obtained when [Ir(cod)Cl]_2_ was used to replace [Rh(cod)Cl]_2_. A subsequent survey of other coordinating anions indicated that the sterically bulky, more weakly coordinating BAr_F_^–^ anion (BAr_F_^–^ = B[(3,5-(CF_3_)_2_C_6_H_3_)]_4_^–^) was the best choice (entries 2–5).^19^ Different solvents were surveyed next. A better yield up to 92% was realized when 1,4-dioxane was employed (entries 6–8). Encouraged by these results, various chiral bisphosphine ligands were investigated next. An excellent yield (90%) and good enantioselectivity (up to –83% ee) could be realized by using (*R*)-Binap (**L2**) as the ligand. Higher ee values were afforded when (*R*)-Tol-Binap (**L3**) and (*R*)-H_8_-Binap (**L4**) were employed (entries 10 and 11). Unexpectedly, the use of either non-biaryl bisphosphine ligand **L5** or monophosphine ligand **L6** resulted in only trace amounts of the corresponding product being produced (entries 12 and 13). Gratifyingly, the best enantioselectivity (>99% ee) was achieved by employing (*R*)-SDP (**L7**) as a ligand (entry 14). For streamlining the operation, [Rh(cod)((*R*)-SDP)]BAr_F_ was prepared in advance and used as a catalyst, providing **3aa** in 93% yield with >99% ee value (entry 15). As the catalytic activity of this [Rh(cod)((*R*)-SDP)]BAr_F_ catalyst was very high, the reaction could even be carried out in the presence of 2.5 mol% of the Rh catalyst without reduction of the product's yield and ee value (entry 16). In addition, lowering the reaction temperature did not give a better result (entry 17).

**Table 1:** Optimization of the reaction conditions for asymmetric hydroamination of Keto-VDCP **1a** with indoline **2a**^*a*^ ^*b*^ ^*c*^

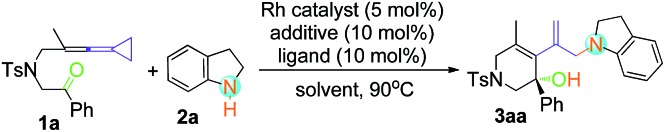						
Entry^*a*^	[Rh]	Additive	Ligand	Solvent	Yield^*b*^ [%]	ee^*c*^ [%]
1	[Rh(cod)Cl]_2_	AgNTf_2_	**L1**	Toluene	75	—
2	[Rh(cod)Cl]_2_	AgBF_4_	**L1**	Toluene	58	—
3	[Rh(cod)Cl]_2_	AgOTf	**L1**	Toluene	62	—
4	[Rh(cod)Cl]_2_	AgOTs	**L1**	Toluene	73	—
5	[Rh(cod)Cl]_2_	NaBAr_F_	**L1**	Toluene	85	—
6	[Rh(cod)Cl]_2_	NaBAr_F_	**L1**	Chlorobenzene	78	—
7	[Rh(cod)Cl]_2_	NaBAr_F_	**L1**	1,2-Dichloroethane	56	—
8	[Rh(cod)Cl]_2_	NaBAr_F_	**L1**	Dioxane	92	—
9	[Rh(cod)Cl]_2_	NaBAr_F_	**L2**	Dioxane	90	–83
10	[Rh(cod)Cl]_2_	NaBAr_F_	**L3**	Dioxane	75	–89
11	[Rh(cod)Cl]_2_	NaBAr_F_	**L4**	Dioxane	77	–93
12	[Rh(cod)Cl]_2_	NaBAr_F_	**L5**	Dioxane	Trace	—
13	[Rh(cod)Cl]_2_	NaBAr_F_	**L6**	Dioxane	Trace	—
14	[Rh(cod)Cl]_2_	NaBAr_F_	**L7**	Dioxane	92	>99
15	[Rh(cod)((*R*)-SDP)]BAr_F_	—	—	Dioxane	93	>99
**16** ^*d*^	**[Rh(cod)((R)-SDP)]BAr**_**F**_	**—**	**—**	**Dioxane**	**91**	**>99**
17^*e*^	[Rh(cod)((*R*)-SDP)]BAr_F_	—	—	Dioxane	68	>99
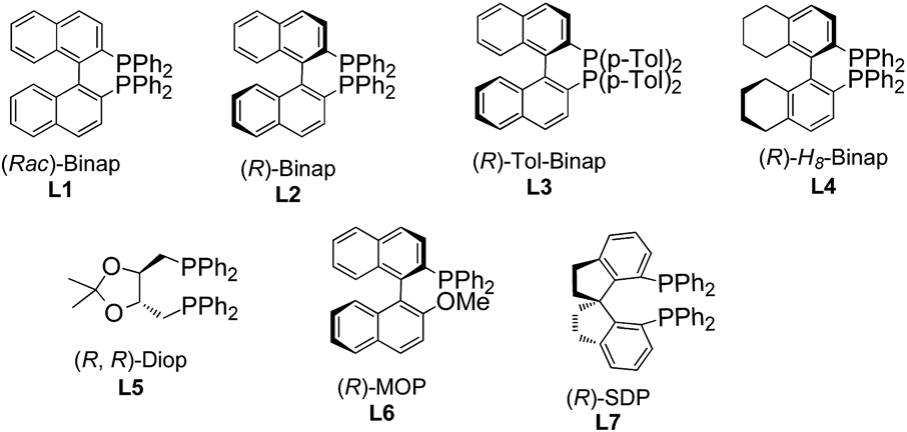						

^*a*^Reaction conditions: **1a** (0.10 mmol), **2a** (0.12 mmol), Rh catalyst (5 mol%), additive (10 mol%), ligand (10 mol%), and solvent (1.0 mL) for 4–12 h.

^*b*^Isolated yield.

^*c*^Determined by HPLC on a chiral stationary phase.

^*d*^[Rh(cod)((*R*)-SDP)]BAr_F_ (2.5 mol%) was employed.

^*e*^The reaction was conducted at 60 °C. Ts = 4-toluenesulfonyl, cod = cyclo-1,5-octadiene, and NaBAr_F_ = sodium tetrakis[3,5-bis(trifluoromethyl)phenyl]borate.

With the optimal reaction conditions in hand, the scope of this asymmetric hydroamination was then assessed through variation of the keto-VDCPs and secondary amines. We first examined the scope of keto-VDCP **1**. As shown in Table 2, the substrate scope of this protocol was broad. For substrates **1b** and **1c** (R^1^ = primary or secondary alkyl groups), the desired products **3ba** and **3ca** were obtained in good to excellent yields (82% and 88%) with outstanding ee values (>99% ee). R^1^ could also be a benzyl or a phenyl group, giving the desired products **3da** and **3ea** in 74% and 80% yields along with >99% ee, respectively. We note that the optimized conditions should be modified with regard to the substituent groups or linkers in keto-VDCP **1**. For substrates **1d**, **1e**, **1s** and **1t**, the reactions proceeded effectively to furnish the corresponding products (**3da**, **3ea**, **3sa** and **3ta**) in good yields (73–83%) with excellent ee values (up to 99% ee) in the presence of [Rh(cod)Cl]_2_, AgNTf_2_ and (*R*)-SDP in toluene at 90 °C. It is noteworthy that the product **3sa** contains a pair of diastereoisomers in a 4 : 1 ratio. The relative configuration of *syn*-**3sa** was determined by nuclear Overhauser effect spectroscopy (see page S58 in the ESI†). The ketone moiety of keto-VDCP **1** was examined next. We found that R^2^ could be an aliphatic, naphthyl or heteroaromatic group, affording the corresponding products **3fa–3ja** in good yields with excellent ee values. As for the substituents at the benzene ring, whether they were electron-rich or electron-poor, the reactions proceeded smoothly to produce the desired products **3ka–3pa** in 78–94% yields with 99% ee values, even for strongly electron-withdrawing substituents such as the nitro group. Besides, no obvious reduction of yields and ee values was observed when different halogen atoms such as F, Cl or Br were introduced. Moreover, even when the halogen substituent was at different positions of the benzene ring, the desired products were produced in similar yields as in the cases of products **3ma**, **3qa** and **3ra**. The use of NBs (4-bromobenzenesulfonyl amide) as a tether was also tolerated in this transformation, giving the corresponding product **3ta** in 83% yield with >99% ee value. However, upon changing the linker to a carbon or an oxygen atom, or extending the carbon chain using a (CH_2_)_2_ or a (CH_2_)_3_ tether, only traces of expected product could be detected by thin-layer chromatography (TLC) monitoring (see Table S2 in the ESI†).

**Table 2:** Substrate scope of the asymmetric hydroamination of Keto-VDCP **1** and indoline **2a**^*a*^ ^*b*^ ^*c*^

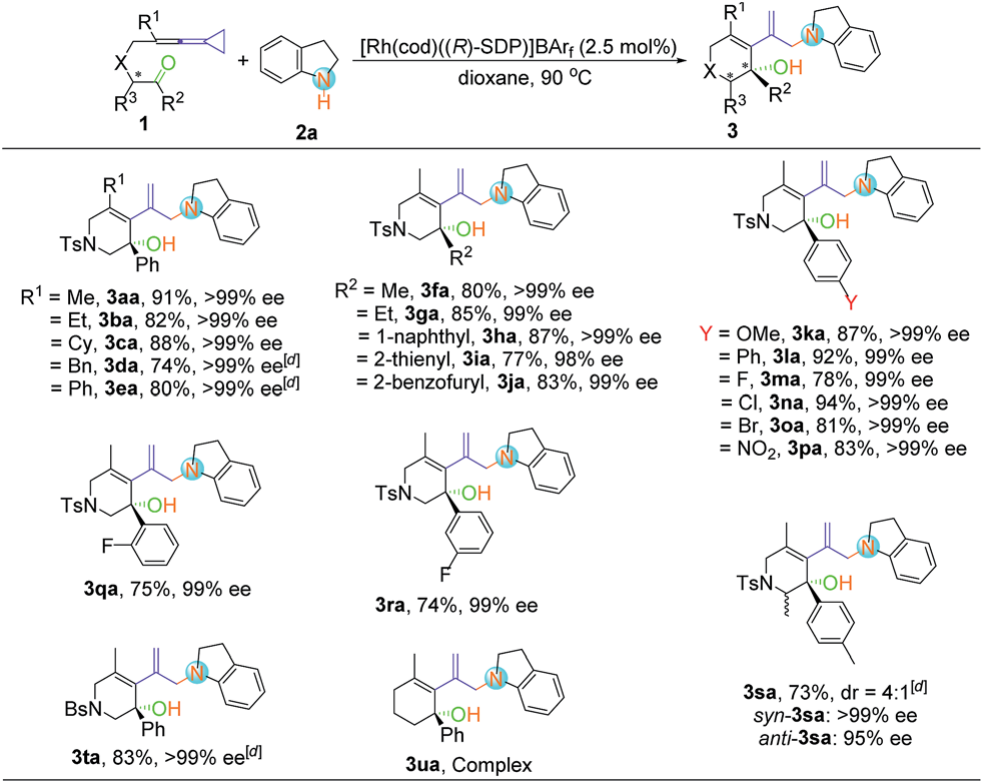

^*a*^Reactions were performed with keto-VDCP **1** (0.10 mmol), secondary amine **2a** (0.12 mmol), and [Rh(cod)((*R*)-SDP)]BAr_F_ (2.5 mol%) in dioxane (1.0 mL) at 90 °C for 4–12 h.

^*b*^Isolated yield.

^*c*^Determined by HPLC on a chiral stationary phase.

^*d*^[Rh(cod)Cl]2 (2.5 mol%), AgNTf_2_ (5.0 mol%), (*R*)-SDP (5.0 mol%), and toluene (1.0 mL) were used.

With respect to secondary amine **2**, various substituents at the indolines were firstly examined (Table 3). Substrates bearing chloro, bromo, methyl and nitryl groups at the different positions of indolines were smoothly transformed into the enantiomerically enriched hydroamination products with good yields (67–87%) and excellent enantioselectivities (97–99% ee). When 1,2,3,4-tetrahydroquinoline was used as the substrate, the desired product **3af** could also be obtained with excellent yield and ee value. However, the yield of **3ag** was slightly decreased when the N-heterocycle was extended to a 7-membered ring. As for non-cyclic secondary amines, the reactions proceeded smoothly to furnish the corresponding products **3ah–3aj** in good to excellent yields with outstanding ee values. In addition, **3ak** could be obtained with 2-anilinoethanol without the use of any protecting group. Disappointingly, none of the desired product was observed under the above conditions when dibenzylamine, diethylamine, morpholine, pyrrolidine and diphenylamine were employed (see Table S2 in the ESI†). It seems that the aryl group was essential because of its electronic nature.

**Table 3:** Substrate scope of the asymmetric hydroamination of Keto-VDCP **1a** and secondary amine **2**^*a*^ ^*b*^ ^*c*^

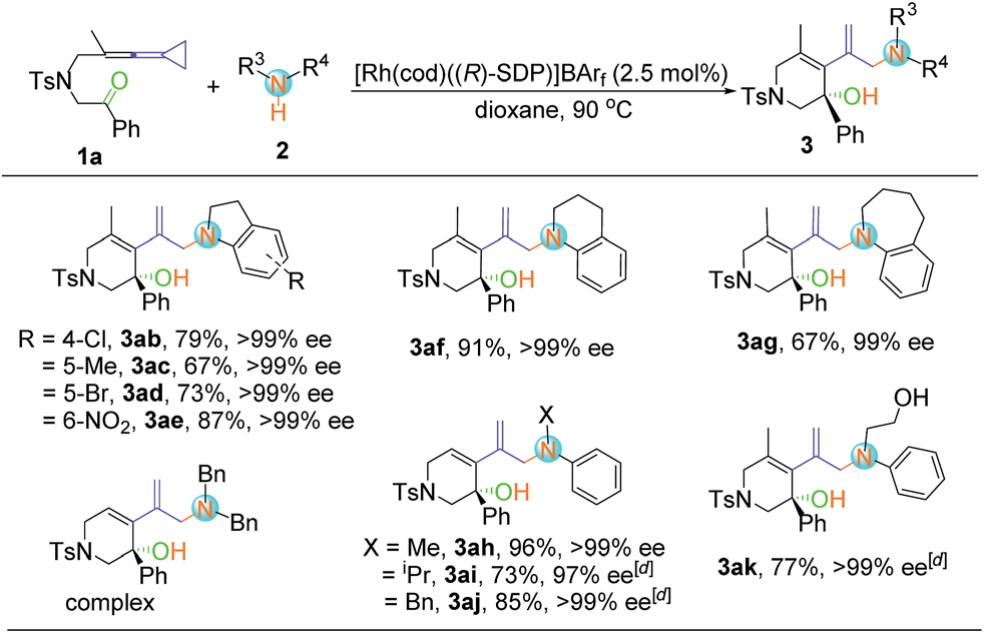

^*a*^Reactions were performed with keto-VDCP **1a** (0.10 mmol), secondary amine **2** (0.12 mmol), and [Rh(cod)((*R*)-SDP)]BAr_F_ (2.5 mol%) in dioxane (1.0 mL) at 90 °C for 4–12 h.

^*b*^Isolated yield.

^*c*^Determined by HPLC on a chiral stationary phase.

^*d*^[Rh(cod)Cl]_2_ (2.5 mol%), AgNTf_2_ (5.0 mol%), (*R*)-SDP (5.0 mol%), and toluene (1.0 mL) were used.

Despite the diverse reactivity of indoles, we observed selective bond formation at the 3-position upon coupling of keto-VDCP **1a** and indoles to yield **5** as the only regioisomer (for details about optimization of the reaction conditions, please see Table S3 in the ESI†).^20^ The structure of **5aa** has been unequivocally determined by X-ray diffraction.^21^ Then, we focused on developing an enantioselective cycloisomerization/cross coupling using indoles as the carbon nucleophile due to the importance of these heterocycles in natural and pharmaceutical products (Table 4). Efficient and selective indole–VDCP cross coupling occurs with a variety of indole substitution patterns. For example, a methyl group or methoxy group can be incorporated at the 2-, 5-, 6- and 7-positions of indole to afford the corresponding products in moderate yields, with up to 99% ee values (**5ab**, **5ac**, **5af**, and **5ag**). In comparison, a lower yield and ee value are observed with 5-bromolindole (**5ae**, 57% yield and 97% ee value). Moreover, we also observed better reactivity with indoles containing two electron-donating groups (**5ah** and **5ai**, up to 82% yield and 98% ee value).

**Table 4:** Substrate scope of the asymmetric hydroindolation of Keto-VDCP **1a** and indole **4**^*a*^ ^*b*^ ^*c*^

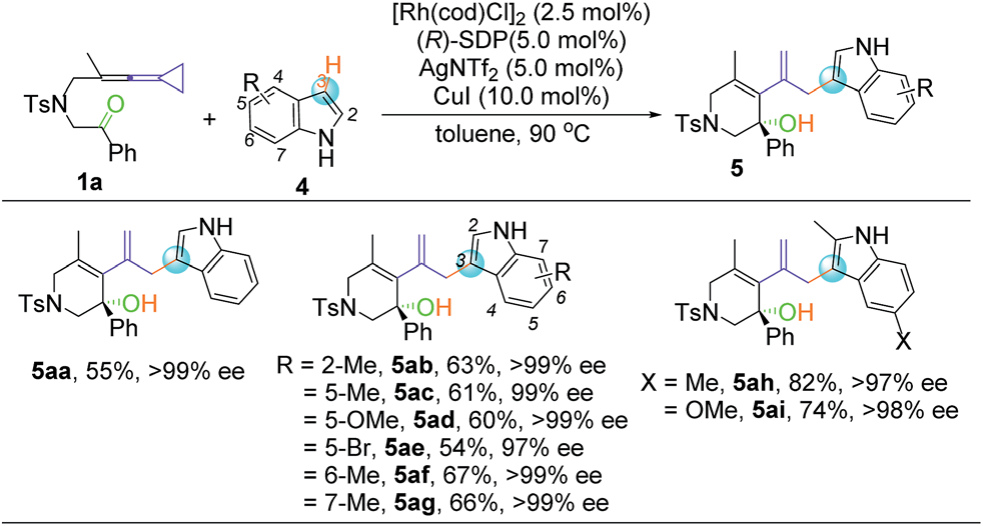

^*a*^Reactions were performed with keto-VDCP **1a** (0.10 mmol), indole **4** (0.12 mmol), [Rh(cod)Cl]_2_ (2.5 mol%), (*R*)-SDP (5.0 mol%), AgNTf_2_ (5.0 mol%) and CuI (10.0 mol%) in toluene (1.0 mL) at 90 °C for 4–12 h.

^*b*^Isolated yield.

^*c*^Determined by HPLC on a chiral stationary phase.

**Scheme 2:**
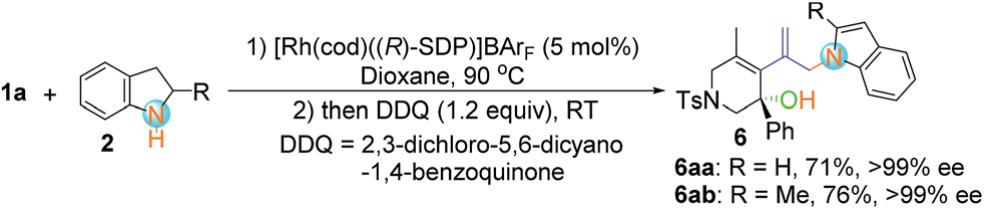
One-pot asymmetric N1 allylic alkylation of indoles.

The enantioselective *N*-substitution of indoles has rarely been explored due to the weak acidity of the N–H group, despite the fact that the products are privileged structural motifs in natural alkaloids and biologically active compounds.^22^ A one-pot protocol was thus developed, using Rh-catalyzed asymmetric hydroamination and subsequent oxidative dehydroaromatization of indoline **2**, providing facile access to N1 allylic alkylation of indoles **6aa** in 71% yield with >99% ee value and **6ab** in 76% yield with >99% ee value (Scheme 2).

**Scheme 3:**
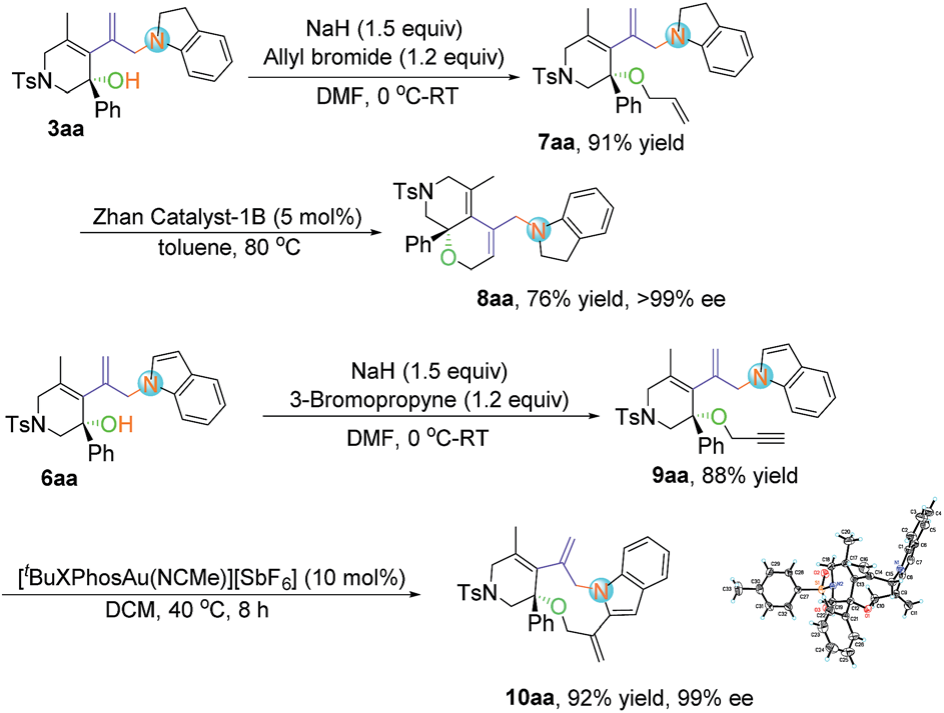
Derivatizations of the products **3aa** and **6aa**.

Considering the easy-to-handle functional groups in products **3** and **6**, further transformations of **3aa** and **6aa** were also examined (Scheme 3). The allyl-substituted product **7aa** could be obtained in 91% yield from **3aa** upon treatment with potassium carbonate and allyl bromide. The subsequent ruthenium-catalyzed intramolecular ring-closing olefin metathesis reaction of **7aa** gave the bicyclic derivative **8aa** in 71% yield with >99% ee. Its structure has been fully confirmed by NMR spectroscopic data including DEPT, COSY, HSQC and HMBC (see pages S112–S117 in the ESI†). Moreover, polycyclic indole **10aa** could be obtained from propargyl-substituted product **9aa** in the presence of [Au(^*t*^BuXPhos)(NCMe)][SbF_6_] (XPhos = 2-dicyclohexylphosphino-2′,4′,6′-triisopropylbiphenyl) (5 mol%) in 92% yield with 99% ee value. The absolute configuration of **10aa** has been determined to be *S* by X-ray diffraction. The ORTEP drawing is shown in Scheme 3 and the CIF data are summarized in the ESI.† 23

### Mechanistic proposal

#### Proposed reaction pathways

**Scheme 4:**
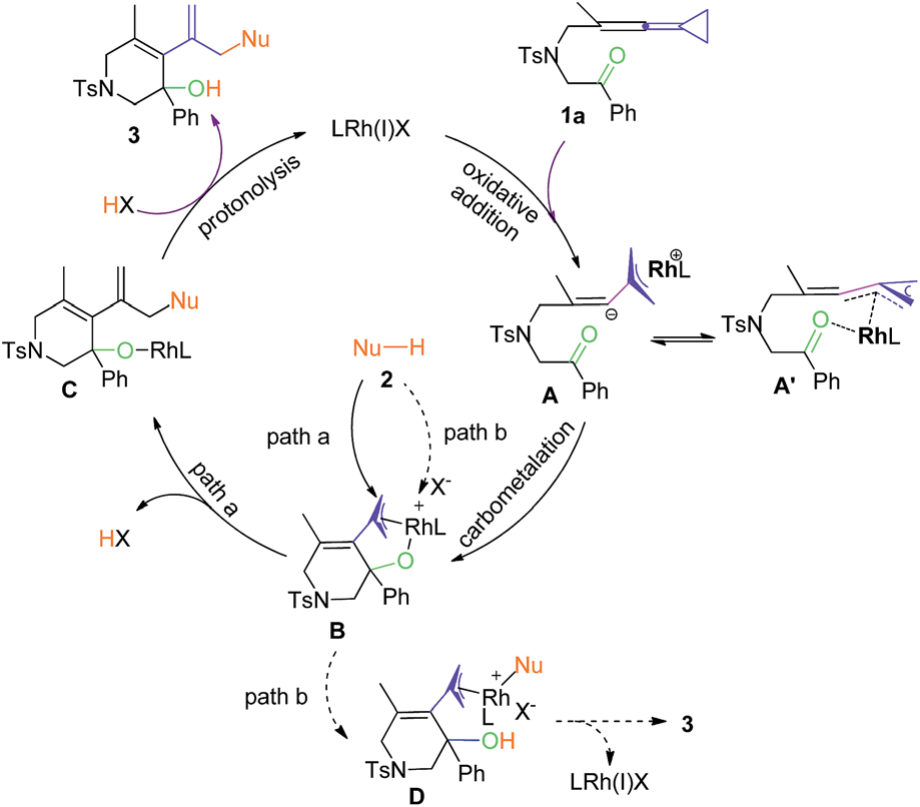
A plausible reaction mechanism.

A plausible reaction mechanism is proposed in Scheme 4 using **1a** as a model substrate for the asymmetric hydroamination and hydroindolation on the basis of previous literature and our own observations. We reasoned that a new TMM–Rh complex **A** or intermediate **A′** was generated from oxidative addition of the weaker distal C–C bond along with isomerization.^24^ A subsequent ketone carbometalation of the TMM–Rh complex **A** led to an electrophilic Rh-π-allyl intermediate **B**. From this intermediate, there are two likely pathways for nucleophilic addition. In path a, the soft nucleophile can directly attack the π-allyl moiety and then generate the corresponding alkoxy Rh intermediate **C** after reduction. Protonolysis of the alkoxy Rh intermediate **C** would afford the final asymmetric hydroamination or hydroindolation product **3**. Alternatively, in path b, the nucleophile attacks the Rh metal center in complex **B** to provide complex **D**. Reductive elimination of **D** releases the desired hydrofunctionalization product. As widely accepted paradigms for classifying the nucleophilic attacking mode on transition metal *p*-allyl intermediates in the Tsuji–Trost reaction, the “soft” nucleophiles generally attack the π-allyl moiety while “hard” nucleophiles first attack the metal center (*via* transmetallation).^25^ Thus, path a in Scheme 4 could be considered as a major process.

## Conclusions

In conclusion, we have developed a novel Rh-catalyzed highly regio- and enantioselective hydrofunctionalization of keto-VDCPs with a wide range of soft nucleophiles. The combination of various secondary amines with keto-VDCPs could afford the hydroamination products in good to excellent yields with outstanding ee values. The highly enantioselective allylic alkylation at both of the C3 and N1 positions of indoles could be realized either by using indoles as nucleophiles directly or *via* a one-pot asymmetric hydroamination and subsequent oxidative dehydroaromatization of indolines. A new TMM–Rh model complex was proposed, which can act as a new atom economical Rh-π-allyl precursor at the same time. Moreover, the resulting multiple functionalized products could easily be transformed into more complex polyheterocycles under ruthenium or gold(i) catalysis. Further investigations to examine the mechanistic details more extensively and exploration of new methodologies based on this novel TMM–metal complex generated from functionalized VDCPs are currently underway in our laboratory.

### General procedure for the synthesis of product **3**

A 10 mL dried tube was charged with keto-VDCP **1** (0.1 mmol, 1.0 equiv.) and [Rh(cod)(*R*-SDP)]BAr_F_ (2.5 mol%, 0.025 equiv.). The reaction tube was evacuated and backfilled with argon (repeated three times). Then, secondary amine **2** (0.12 mmol, 1.2 equiv.) and dioxane (1.0 mL) were added into the tube. The reaction mixture was stirred at 90 °C for 4–10 h. The solvent was removed under reduced pressure and the residue was purified by flash column chromatography (SiO_2_) to give the corresponding product **3**.

#### Compound **3aa**

A white solid, 91% yield (45 mg). M. P. 105–107 °C. ^1^H NMR (CDCl_3_, 400 MHz, TMS) *δ* 1.87 (s, 3H), 2.40 (s, 3H), 2.85 (d, *J* = 11.6 Hz, 1H), 2.87–2.91 (m, 2H), 2.95–3.02 (m, 1H), 3.09 (dd, *J*_1_ = 8.0 Hz, *J*_2_ = 16.0 Hz, 1H), 3.25 (d, *J* = 14.8 Hz, 1H), 3.43 (d, *J* = 14.8 Hz, 1H), 3.45 (d, *J* = 16.4 Hz, 1H), 3.52 (d, *J* = 11.6 Hz, 1H), 3.89 (d, *J* = 16.4 Hz, 1H), 4.72 (s, 1H), 4.82 (brs, 1H), 5.18 (d, *J* = 0.8 Hz, 1H), 6.26 (d, *J* = 7.6 Hz, 1H), 6.70 (dd, *J*_1_ = 7.2 Hz, *J*_2_ = 7.6 Hz, 1H), 6.99 (dd, *J*_1_ = 7.2 Hz, *J*_2_ = 7.6 Hz, 1H), 7.06 (d, *J* = 7.2 Hz, 1H), 7.24–7.33 (m, 5H), 7.46 (d, *J* = 8.4 Hz, 2H), 7.62 (d, *J* = 8.8 Hz, 2H). ^13^C NMR (CDCl_3_, 100 MHz, TMS) *δ* 18.6, 21.5, 28.4, 49.6, 54.1, 57.0, 57.3, 72.6, 108.9, 119.2, 119.8, 124.4, 126.5, 127.13, 127.15, 127.75, 127.77, 129.7, 130.4, 130.5, 133.0, 136.1, 141.4, 143.1, 143.6, 151.5. IR (CH_2_Cl_2_) *ν* 2971, 2920, 2850, 2360, 2342, 1603, 1518, 1486, 1456, 1343, 1305, 1249, 1158, 1090, 1022, 988, 911, 873, 857, 811, 749, 705 cm^–1^. HRMS (ESI) calcd for C_30_H_33_N_2_O_3_S (M + H)^+^: 501.2206, found: 501.2198. Enantiomeric excess was determined by HPLC with a Chiralcel AD-H column [*λ* = 254 nm; eluent: hexane/isopropanol = 80/20; flow rate: 0.50 mL min^–1^; *t*_minor_ = 26.03 min, *t*_major_ = 23.03 min; ee% > 99%; [*α*]20D = +26.2 (*c* 1.00, CH_2_Cl_2_)].

### General procedure for the synthesis of product **5**

A 10 mL dried tube was charged with Keto-VDCP **1a** (0.1 mmol, 1.0 equiv.), [Rh(COD)Cl]_2_ (0.0025 mmol, 0.025 equiv.), (*R*)-SDP (0.005 mmol, 0.05 equiv.), AgNTf_2_ (0.005 mmol, 0.05 equiv.) and CuI (0.010 mmol, 0.10 equiv.). The reaction tube was evacuated and backfilled with argon (repeated three times). Then, indole **4** (0.12 mmol, 1.2 equiv.) and toluene (1.0 mL) were added into the tube. The reaction mixture was stirred at 90 °C for 4–10 h. The solvent was removed under reduced pressure and the residue was purified by flash column chromatography (SiO_2_) to give the corresponding product **5**.

#### Compound **5aa**

A white solid, 55% yield (27 mg). M. P. 183–185 °C. ^1^H NMR (400 MHz, CDCl_3_, TMS) *δ* 1.66 (s, 3H), 2.42 (s, 3H), 2.87 (s, 1H), 2.95 (d, *J* = 11.6 Hz, 1H), 3.17 (d, *J* = 17.2 Hz, 1H), 3.29 (d, *J* = 17.2 Hz, 1H), 3.38 (d, *J* = 16.0 Hz, 1H), 3.48 (d, *J* = 11.6 Hz, 1H), 3.78 (d, *J* = 16.0 Hz, 1H), 4.60 (s, 1H), 4.82 (d, *J* = 1.6 Hz, 1H), 6.79 (d, *J* = 3.0 Hz, 1H), 6.94–6.98 (m, 1H), 7.05 (d, *J* = 8.0 Hz, 1H), 7.09–7.13 (m, 1H), 7.28–7.36 (m, 6H), 7.50 (d, *J* = 8.0 Hz, 2H), 7.62 (d, *J* = 8.0 Hz, 2H), 7.92 (s, 1H). ^13^C NMR (100 MHz, CDCl_3_, TMS) *δ* 18.3, 21.5, 32.5, 49.5, 57.6, 72.8, 110.9, 112.8, 117.7, 119.0, 119.3, 121.7, 122.7, 126.6, 127.24, 127.78, 127.83, 128.6, 129.8, 132.5, 136.2, 137.9, 141.9, 143.9, 144.5. IR (CH_2_Cl_2_): *ν* 3455, 3328, 3062, 3031, 2970, 2921, 2848, 2820, 2360, 2342, 1598, 1491, 1447, 1393, 1346, 1184, 1169, 1153, 1107, 1090, 1051, 1039, 1018, 982, 944, 918, 900, 860, 809, 742, 703, 677, 661 cm^–1^. HRMS (ESI) calcd for C_30_H_34_N_3_O_3_S (M + NH_4_)^+^: 516.2315, found: 516.2310. Enantiomeric excess was determined by HPLC with a Chiralcel IC-H column [*λ* = 254 nm; eluent: hexane/isopropanol = 80/20; flow rate: 0.50 mL min^–1^; *t*_minor_ = 13.68 min, *t*_major_ = 18.18 min; ee% > 99%; [*α*]20D = +38.2 (*c* 1.00, CH_2_Cl_2_)].

### Typical procedure for the preparation of compound **6aa**

To a flame dried Schlenk tube were added compound **3aa** (0.2 mmol), NaH (60% dispersion in mineral oil, 1.5 equiv.) and DMF (2.0 mL). The reaction mixture was stirred at 0 °C for 0.5 h before allyl bromide (1.2 equiv.) was added. The reaction mixture was stirred at 0 °C for another 4 h. Then, the reaction mixture was diluted with cold water and extracted with ether (4 mL × 3) and the combined organics were dried over anhydrous Na_2_SO_4_. The solvent was removed under reduced pressure and the residue was purified by flash column chromatography (SiO_2_) to give the corresponding product **7aa**.

Under an argon atmosphere, compound **7aa** (0.1 mmo, 1.0 equiv.), Zhan-catalyst-1B (0.10 equiv.) and toluene (10 mL) were added into a Schlenk tube and then the mixture was heated at 80 °C for 12 h. Then, the solvent was removed under reduced pressure and the residue was purified by flash column chromatography (SiO_2_) to give the corresponding product **8aa**.

#### Compound **8aa**

A white solid, 76% yield (39 mg). M. P. 90–92 °C. ^1^H NMR (CDCl_3_, 400 MHz, TMS) *δ* 1.98 (s, 3H), 2.39 (s, 3H), 2.76 (d, *J* = 10.8 Hz, 1H), 2.82–2.95 (m, 2H), 3.06–3.17 (m, 2H), 3.27 (d, *J* = 17.2 Hz, 1H), 3.79–3.85 (m, 2H), 4.00 (d, *J* = 16.0 Hz, 2H), 4.07 (d, *J* = 18.0 Hz, 1H), 4.20 (d, *J* = 18.0 Hz, 1H), 5.65 (s, 1H), 6.30 (d, *J* = 8.0 Hz, 1H), 6.63 (dd, *J*_1_ = 6.8 Hz, *J*_2_ = 7.2 Hz, 1H), 6.96 (dd, *J*_1_ = 7.2 Hz, *J*_2_ = 7.6 Hz, 1H), 7.05 (d, *J* = 7.2 Hz, 1H), 7.20 (d, *J* = 8.0 Hz, 2H), 7.28–7.33 (m, 5H), 7.44 (d, *J* = 8.4 Hz, 2H). ^13^C NMR (CDCl_3_, 125 MHz, TMS) *δ* 18.7, 21.5, 28.4, 51.3, 53.9, 54.8, 55.5, 61.9, 76.0, 106.8, 117.7, 124.4, 126.4, 127.16, 127.22, 127.38, 127.49, 127.63, 127.74, 127.95, 129.5, 129.9, 130.0, 133.2, 140.5, 143.5, 152.0. IR (CH_2_Cl_2_) *ν* 3060, 3021, 2920, 2828, 2161, 1980, 1606, 1489, 1448, 1351, 1304, 1253, 1159, 1131, 1089, 1056, 1010, 958, 902, 813, 747, 704 cm^–1^. HRMS (ESI) calcd for C_31_H_33_N_2_O_3_S (M + H)^+^: 513.2206, found: 513.2211. Enantiomeric excess was determined by HPLC with a Chiralcel IC-H column [*λ* = 230 nm; eluent: hexane/isopropanol = 80/20; flow rate: 0.50 mL min^–1^; *t*_minor_ = 37.50 min, *t*_major_ = 52.10 min; ee% > 99%; [*α*]20D = –105.1 (*c* 1.00, CH_2_Cl_2_)].

### Typical procedure for the preparation of compound **10aa**

To a flame dried Schlenk tube were added compound **6aa** (0.2 mmol), NaH (60% dispersion in mineral oil, 1.5 equiv.) and DMF (2.0 mL). The reaction mixture was stirred at 0 °C for 0.5 h before 3-bromopropyne bromide (1.2 equiv.) was added. The reaction mixture was stirred at 0 °C for another 4 h. Then, the reaction mixture was diluted with cold water and extracted with ether (4 mL × 3) and the combined organics were dried over anhydrous Na_2_SO_4_. The solvent was removed under reduced pressure and the residue was purified by flash column chromatography (SiO_2_) to give the corresponding product **9aa**.

To a flame dried Schlenk tube were added **9aa** (0.1 mmol, 1.0 equiv.), [Au(^*t*^BuXPhos)(NCMe)][SbF_6_] (10 mol%) and DCM (2.0 mL), and the resulting mixture was stirred at 10 °C for 8 h. The solvent was removed under reduced pressure and the residue was purified by flash column chromatography (SiO_2_) to give the corresponding product **10aa**.

#### Compound **10aa**

A white solid, 92% yield (49 mg). M. P. 190–192 °C. ^1^H NMR (CDCl_3_, 400 MHz, TMS) *δ* 1.41 (s, 3H), 2.39 (s, 3H), 2.75 (d, *J* = 12.4 Hz, 1H), 3.41 (d, *J* = 16.0 Hz, 1H), 3.85 (d, *J* = 16.0 Hz, 1H), 3.96 (d, *J* = 12.4 Hz, 1H), 4.25 (d, *J* = 14.8 Hz, 1H), 4.67–4.72 (s, 2H), 4.95 (d, *J* = 11.6 Hz, 1H), 5.04 (d, *J* = 14.8 Hz, 1H), 5.41 (s, 1H), 5.57 (s, 1H), 5.61 (d, *J* = 2.0 Hz, 1H), 6.29 (s, 1H), 7.03–7.13 (m, 2H), 7.20–7.23 (m, 1H), 7.26–7.29 (m, 5H), 7.40 (d, *J* = 7.6 Hz, 2H), 7.55 (m, 3H). ^13^C NMR (CDCl_3_, 125 MHz, TMS) *δ* 18.2, 21.6, 49.4, 52.5, 57.0, 68.2, 78.4, 101.6, 110.1, 119.6, 120.5, 121.5, 122.0, 123.4, 126.8, 127.2, 127.8, 128.1, 129.9, 130.7, 132.1, 135.2, 138.9, 139.1, 141.1, 142.5, 143.7, 144.0 cm^–1^. IR (CH_2_Cl_2_) *ν* 3027, 2920, 2856, 2804, 2360, 2340, 1631, 1600, 1533, 1492, 1458, 1449, 1403, 1386, 1344, 1331, 1309, 1252, 1224, 1170, 1160, 1108, 1093, 1055, 1018, 977, 958, 935, 895, 860, 807, 788, 764, 757, 743, 706, 664 cm^–1^. HRMS (ESI) calcd for C_33_H_33_N_2_O_3_S (M + H)^+^: 537.2206, found: 537.2200. Enantiomeric excess was determined by HPLC with a Chiralcel IC-H column [*λ* = 254 nm; eluent: hexane/isopropanol = 80/20; flow rate: 0.50 mL min^–1^; *t*_minor_ = 15.70 min, *t*_major_ = 18.88 min; ee% = 99%; [*α*]20D = 37.7 (*c* 1.00, CH_2_Cl_2_)].

## Conflicts of interest

There are no conflicts to declare.

## Supplementary Material

Supplementary information

Crystal structure data
