# Supplementary material for: Rhodium-catalyzed asymmetric hydroamination and hydroindolation of keto-vinylidenecyclopropanes
Source: Chem Sci. 2018 May 11;9(22):5074–81. doi: 10.1039/c8sc01595c (PMC5994874; doi:10.1039/c8sc01595c)
Supplement: Supplementary file 1 [file SC-009-C8SC01595C-s001.pdf]

Electronic Supplementary Material (ESI) for Chemical Science.
This journal is © The Royal Society of Chemistry 2018

Supporting Information

Rhodium-Catalyzed Asymmetric Hydroamination and Hydroindolation of Keto-Vinylidenecyclopropanes

Song Yang,[a] Quan-Zhe Li,[a] Chen Xu,[a] Qin Xu,[a] and Min Shi[abc]*

[a]Key Laboratory for Advanced Materials and Institute of Fine Chemicals, School of Chemistry & Molecular Engineering, East China University of Science and Technology, 130 MESI-Long Road, Shanghai 200237 China.

[b]State Key Laboratory of Organometallic Chemistry, Center for Excellence in Molecular Synthesis, Shanghai Institute of Organic Chemistry, Center for Excellence in Molecular Synthesis, University of Chinese Academy of Sciences, Chinese Academy of Sciences, 354 Fenglin Road, Shanghai 200032 China.

[c]State Key Laboratory and Institute of Element-Organic Chemistry, Collaborative Innovation Center of Chemical Science and Engineering (Tianjin), Nankai University, Tianjin 300071, China.

Email: qlzhou@nankai.edu.cn

Mshi@mail.sioc.ac.cn. Fax 86-21-64166128

CONTENTS

1. General remarks……………………………………………………………………...…S3
2. General procedure for synthesis of Keto-Vinylidenecyclopropanes **1**…..……….……......S4-S5
3. Typical procedures for synthesis of hydroamination products **3**………………………......S5
4. Unsuccessful examples for other nucleophiles about the hydrofunctionalization of Keto-VDCP **1a**…………………………………………………………………………S6
5. Unsuccessful examples for other substrates about the hydroamination of Keto-VDCPs **1** with secondary amines **2**………………………………………………………………..…S6
6. Optimization of the asymmetric hydroindolation of Keto-VDCPs **1**……………….............S7
7. Typical procedures for synthesis of hydroindolation products **5**…………………..……......S7-S8
8. Typical procedure for the one pot synthesis of N-allylic indoles **6**…………………….……S8
9. Characterization and spectra charts for compounds **S6g** and **1g**…...............................S8-S10

10. Characterization and spectra charts for products **3**, **5** and **6**……………………….………S10-S110

11. Transformations of products **3** and **6aa**…………………………………………..….S110-S123

12. X-ray crystallographic information of products **3aa**, **5aa** and **10aa**………….……….S124-S125

13. References……………………………………………………………………………...….S126

1. General Remarks. MP was obtained with a Yanagimoto micro melting point apparatus and is uncorrected. Infra-red spectra were measured on a spectrometer. ^{1}H NMR spectra were recorded for solution in $CDCl_3$ with tetramethylsilane (TMS) as internal standard; ^{19}F NMR spectra were recorded for a solution in $CDCl_3$ with $CFCl_3$ as the external reference. *J*-values are in Hz. Mass spectra were recorded with a HP-5989 instrument and HRMS was measured by a Finnigan MA+ mass spectrometer. Organic solvents used were dried by standard methods when necessary. Commercially obtained reagents were used without further purification. All reactions were monitored by TLC with Huanghai GF_{254} silica gel coated plates. Flash column chromatography was carried out using 300-400 mesh silica gel at increased pressure. All reactions were performed under argon using standard Schlenk techniques.

2. General procedure for synthesis of Keto-Vinylidenecyclopropanes 1:

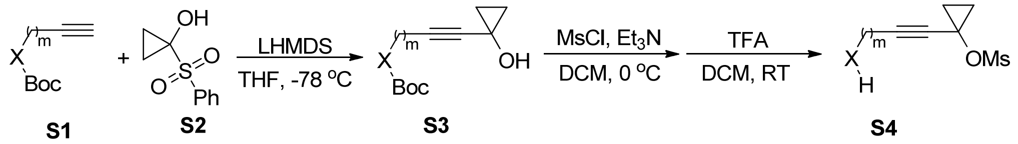

m = 1,2,3; X = NTs, NBs

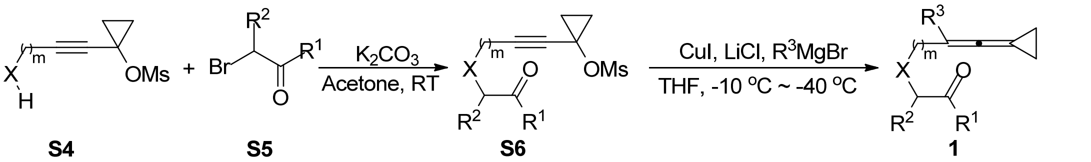

m = 1,2,3
X = NTs, NBs

R^1 = Ar, Het-Ar, Alkyl
R^2 = H, Me

m = 1,2,3
X = NTs, NBs, O, CH_2
R^1 = Ar, Het-Ar, Alkyl
R^2 = H, Me

m = 1,2,3
X = NTs, NBs, O, CH_2
R^1 = Ar, Het-Ar, Alkyl
R^2 = H, Me
R^3 = Alkyl, Ar

The synthesis and characterization of compounds **1a-1f** and **1h-1u** have been presented in our previous work.[1]

To the solution of compounds **S1** (20 mmol) in THF (30 mL) was added LMHDS (22 mmol, 1.0 M in THF) within 20 min at -78 °C under argon. The resulting solution was allowed to stir at -78 °C for 0.5 h before a solution of **S2** (10 mmol) in THF (10 mL) was added into the above mixture. Consequently, the reaction mixture was allowed to warm up to room temperature and was stirred for 8 h. Then, saturated HCl solution was added to quench the reaction. Extracted with ethyl ether, dried over anhydrous Na_2SO_4, filtered, the organic phase was purified by a flash column chromatography on silica gel to give the corresponding products **S3**. (PE/EA: 4:1~2:1).

Under argon atmosphere, compound **S3** (4.0 mmol) was dissolved in DCM (10.0 mL) at 0 °C, Et_3N (8.0 mmol) and MsCl (6.0 mmol) was added. After stirring for 1 h, the reaction was quenched with H_2O (10.0 mL), extracted with DCM (10 mL x 3), and dried over anhydrous Na_2SO_4. The solvent was removed under reduced pressure and the residue was transferred into a 50 mL flask with 10 mL DCM. Then, trifluoroacetic acid (TFA, 40 mmol) was added dropwise. After stirring for 12 h, the reaction was quenched with saturated $NaCO_3$ solution, extracted with DCM (10 mL x 3), and dried over anhydrous Na_2SO_4. The solvent was removed under reduced pressure and the residue was purified by a flash column chromatography (SiO_2) to give the corresponding product **S4**. (PE/EA: 4:1~1:1).

To the solution of **S4** (1.5 mmol) and K_2CO_3 (1.8 mmol) in acetone (10 ml) was added **S5** (1.8 mmol). The resulting solution was allowed to stir at room temperature for 8 h. Then, H_2O was

added to quench the reaction. The reaction mixture was extracted with EA twice, dried over anhydrous Na_2SO_4. The solvent was removed under reduced pressure and the residue was purified by a flash column chromatography (SiO_2) to give the corresponding product **S6** for two steps (PE/EA: 4:1~2:1).

Under argon atmosphere, CuI (2.2 mmol) and LiCl (2.2 mmol) in a three-necked bottle was dried upon heating. Then THF (10 mL) was added. At -5 ^{o}C, R^3MgBr (1.0 mol/L in THF, 2.0 mmol, 2.0 mL) was added to the reaction. 10 minutes later, the flask was moved into a -40 ^{o}C bath and stirred for a while before a solution of **S6** (1.0 mmol) in THF (10 mL) was added dropwise into the above flask. After stirring at -40 ^{o}C for 8 h, the reaction was quenched with saturated NH_4Cl solution, extracted with EA (10 mL x 3), and dried over anhydrous Na_2SO_4. The solvent was removed under reduced pressure and the residue was purified by a flash column chromatography (SiO_2) to give the corresponding product **1**. (PE/EA: 10:1)

3. Typical procedures for synthesis of hydroamination products 3

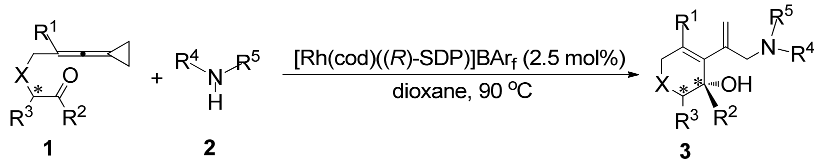

To a 10 mL dried tube was charged with Keto-VDCPs **1** (0.1 mmol, 1.0 equiv) and [Rh(cod)(R-SDP)]BArf (2.5 mol%, 0.025 equiv). The tube was evacuated and backfilled with argon (repeated three times). Then, secondary amines **2** (0.12 mmol, 1.2 equiv) and dioxane (1.0 mL) was added into the tube. The reaction mixture was stirred at 90 °C for 4-10 h. The solvent was removed under reduced pressure and the residue was purified by a flash column chromatography (SiO_2) to give the corresponding product **3**.

4. Table S1. Unsuccessful examples for other nucleophiles about the hydrofunctionalization of Keto-VDCP 1a

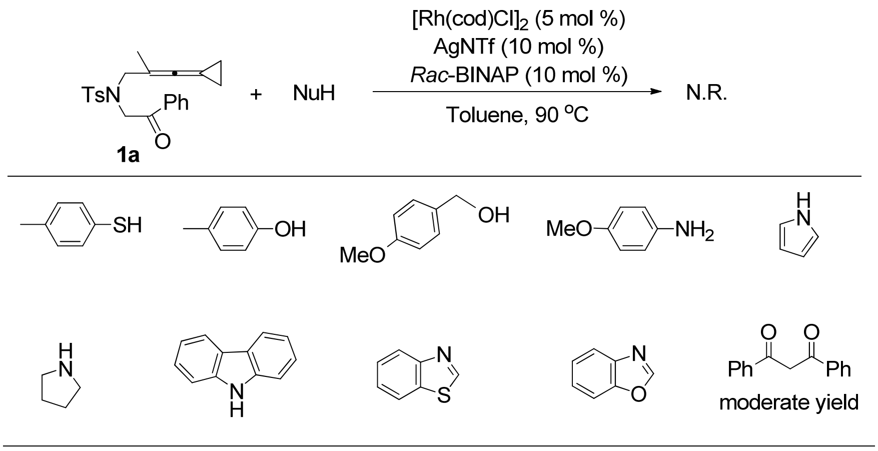

5. Table S2. Unsuccessful examples for other substrates about the hydroamination of Keto-VDCPs 1 with secondary amines 2

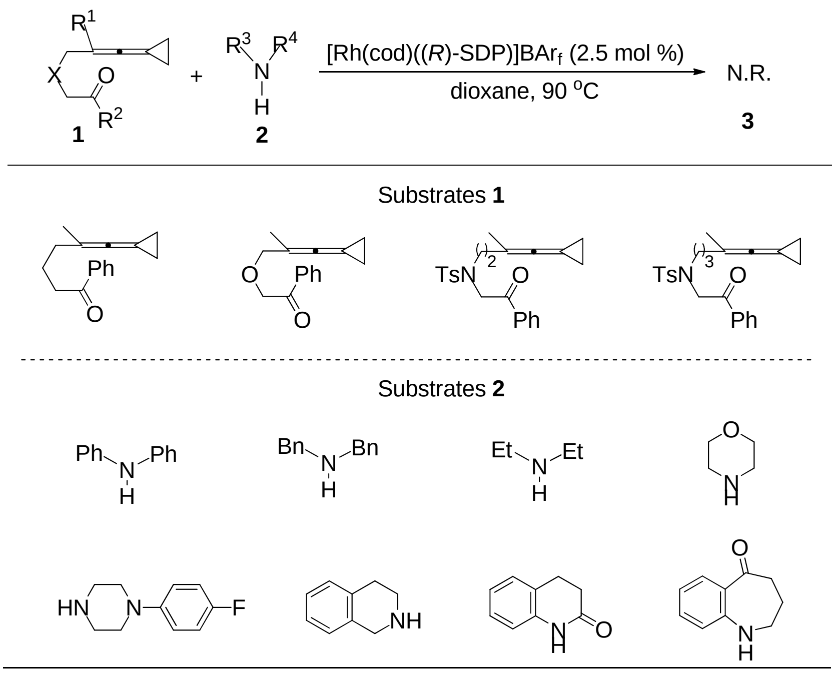

6. Table S3. Optimization of the asymmetric hydroindolation of Keto-VDCPs 1a and Indole 4b

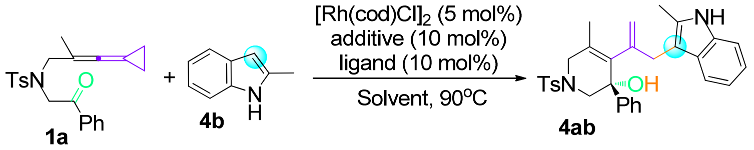

entry[a]	additive	ligand	solvent	yield[%][b]	ee[%][c]
1	$AgNTf_2$	**L1**	Dioxane	35	-
2	$AgNTf_2$	**L1**	Toluene	42	-
3	$AgNTf_2$	**L1**	DCE	14	-
4	$AgNTf_2$	**L1**	PhF	trace	-
5	$AgNTf_2$	**L1**	$PhCF_3$	Trace	-
6	$AgBF_4$	**L1**	Toluene	30	-
7	$NaBAr_F$	**L1**	Toluene	23	-
8	$AgNTf_2/FeCl_3$	**L1**	Toluene	Trace	-
9	$AgNTf_2$/CuI	**L1**	Toluene	65	-
10	$AgNTf_2$/CuI	**L2**	Toluene	48	-55
11	$AgNTf_2$/CuI	**L3**	Toluene	56	-67
12	$AgNTf_2$/CuI	**L4**	Toluene	61	-80
13	$AgNTf_2$/CuI	**L5**	Toluene	Trace	-
14	$AgNTf_2$/CuI	**L6**	Toluene	Trace	-
15	$AgNTf_2$/CuI	**L7**	Toluene	63	>99

[a] Reaction conditions: **1a** (0.10 mmol), **4b** (0.12 mmol), $[Rh(cod)Cl]_2$ (5 mol %), additive (10 mol%), ligand (10 mol%), and solvent (1.0 mL) were used. 4-12 h. [b] Isolated yield. [c] Determined by HPLC on a chiral stationary phase. cod = cyclo-1,5-octadiene, Ts = 4-toluenesulfonyl. [e] The reaction was conducted at 60 °C. cod = cyclo-1,5-octadiene, $NaBAr_F$= sodium tetrakis[3,5-bis(trifluoromethyl)phenyl] borate.

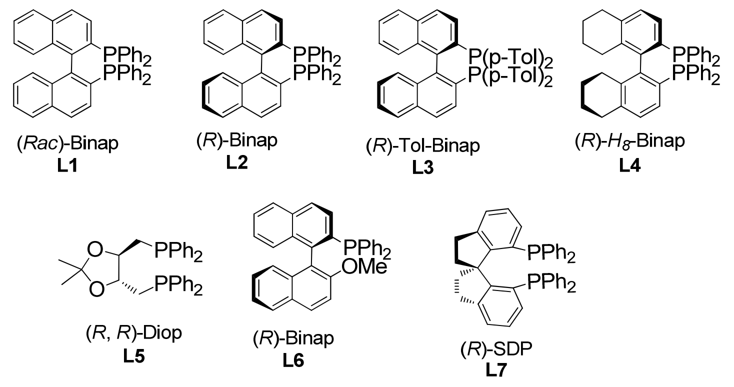

7. Typical procedures for synthesis of hydroindolation products 5

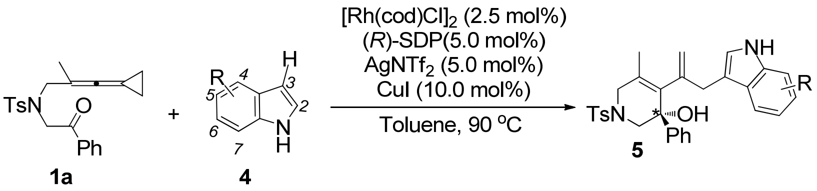

To a 10 mL dried tube was charged with Keto-VDCPs **1a** (0.1 mmol, 1.0 equiv), $[Rh(COD)Cl]_2$ (0.0025 mmol, 0.025 equiv), (*R*)-SDP (0.005 mmol, 0.05 equiv) $AgNTf_2$ (0.005 mmol, 0.05 equiv) and CuI (0.010 mmol, 0.10 equiv). The tube was evacuated and backfilled with argon (repeated three times). Then, indoles **4** (0.12 mmol, 1.2 equiv) and toluene (1.0 mL) was added into the tube. The reaction mixture was stirring at 90 °C for 4-10 h. The solvent was removed under reduced pressure and the residue was purified by a flash column chromatography (SiO_2) to give the corresponding product **5**.

8. Typical procedure for the one pot synthesis of N-allylic indoles 6

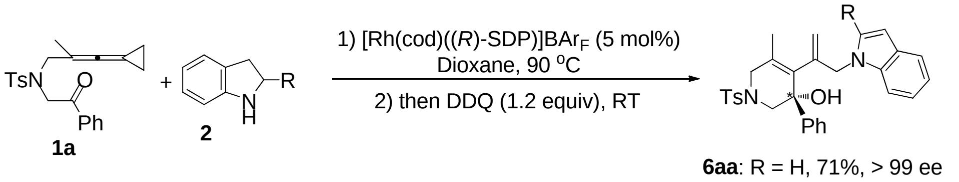

6aa: R = H, 71%, > 99 ee
6ab: R = Me, 76%, > 99 ee

To a 10 mL dried tube was charged with Keto-VDCPs **1** (0.2 mmol, 1.0 equiv) and [Rh(cod)((*R*)-SDP)]BArf (2.5 mol%, 0.025 equiv). The tube was evacuated and backfilled with argon (repeated three times). Then, secondary amines **2** (0.24 mmol, 1.2 equiv) and dioxane (2.0 mL) was added into the tube. After heating the reaction mixture at 90 °C for 4-10 h, the resulting solution was cooled to room temperature. Then DDQ (2,3-dichloro-5,6-dicyano-p-benzoquinone, 0.30 mmol) was added and the reaction system was stirred for 8 h at room temperature. The solvent was removed under reduced pressure and the residue was purified by a flash column chromatography (SiO_2) to give the corresponding product **6**.

9. Characterization and spectra charts for compounds S6g and 1g.

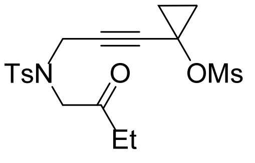

1-(3-((4-methyl-N-(2-oxobutyl)phenyl)sulfonamido)prop-1-yn-1-yl)cyclopropyl

methanesulfonate (S6g)

1.5 mmol scale, a light yellow oil, 92% yield (571 mg). ^{1}H NMR ($CDCl_3$, 400 MHz, TMS) δ 0.97 (dd, J_1 = 6.0 Hz, J_2 = 8.4 Hz, 2H), 1.08 (t, J = 7.2 Hz, 3H), 1.44 (dd, J_1 = 6.0 Hz, J_2 = 8.4 Hz, 2H), 2.44 (s, 3H), 2.54 (q, J = 7.2 Hz, 2H), 3.04 (s, 3H), 4.10 (s, 2H), 4.24 (s, 2H), 7.33 (d, J = 8.0 Hz, 2H), 7.71 (d, J = 8.0 Hz, 2H). ^{13}C NMR ($CDCl_3$, 100 MHz, TMS) δ 7.3, 16.3, 21.5, 32.9, 37.9, 39.6, 53.1, 54.3, 79.0, 83.4, 127.5, 129.7, 135.5, 144.0, 205.8. IR (CH_2Cl_2) ν 3397, 3054, 3023, 2971, 2917, 2845, 2360, 2342, 1734, 1624, 1597, 1489, 1448, 1342, 1306, 1248, 1090, 1043, 986, 940, 918, 899, 864, 805, 765, 723, 704, 663 cm^{-1}. HRMS (ESI) calcd. for $C_{18}H_{27}N_2O_6S_2$ $(M+NH_4)^+$: 431.1305, Found: 431.1299.

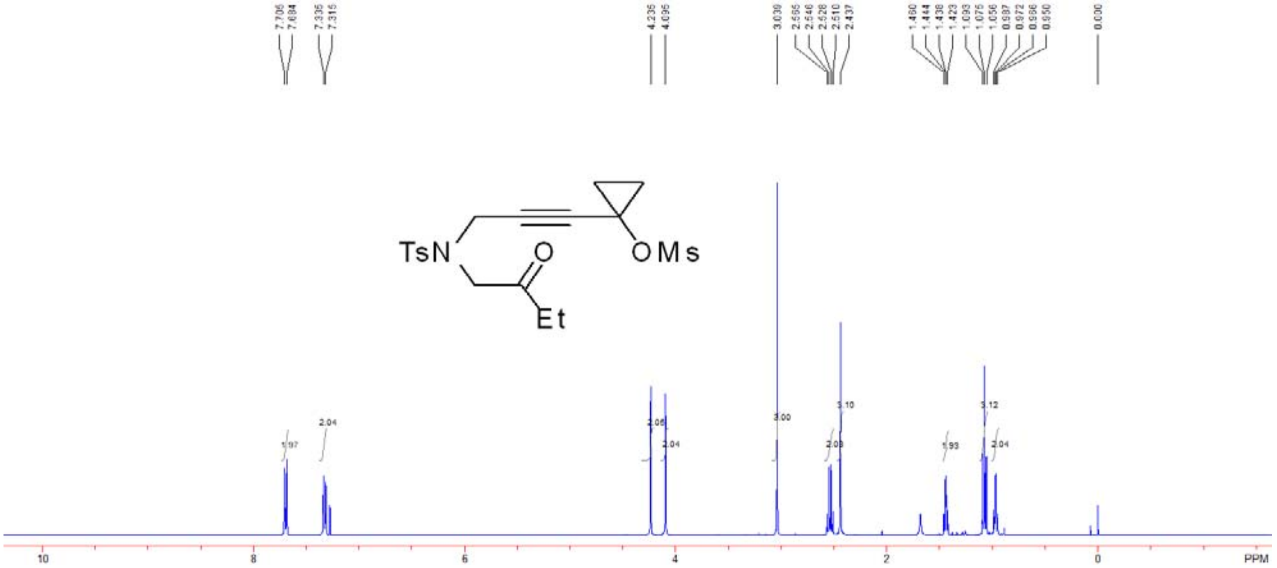

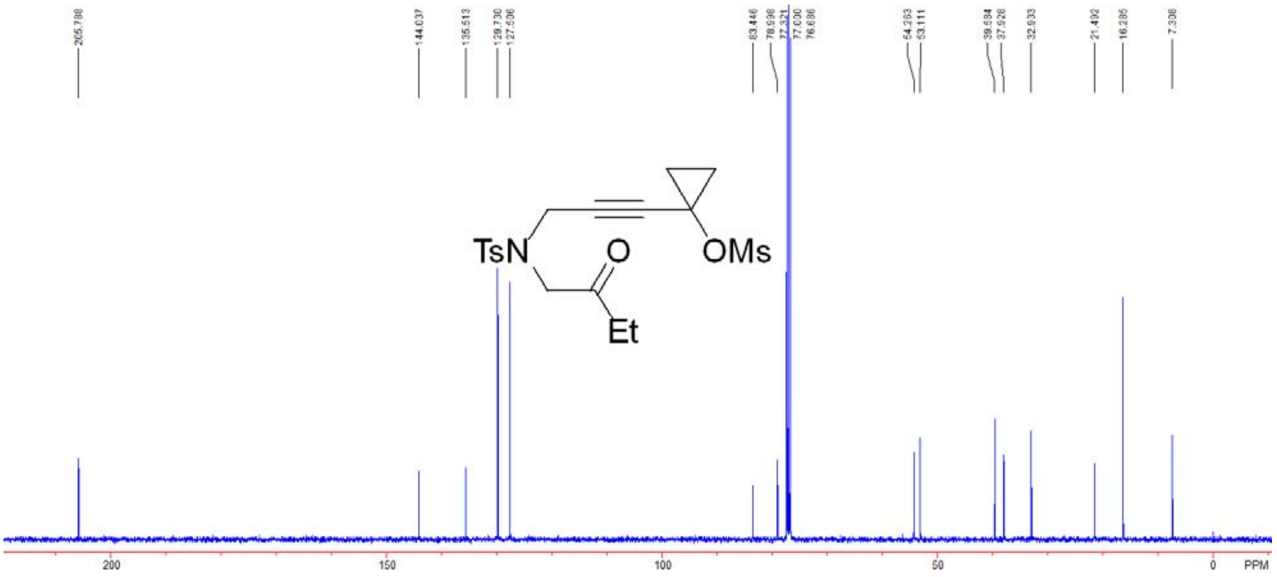

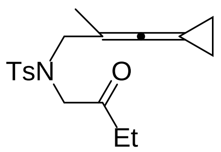

N-(3-cyclopropylidene-2-methyl-3l5-allyl)-4-methyl-N-(2-oxobutyl)benzenesulfonamide (1g)

1.0 mmol scale, a light yellow oil, 95% yield (316 mg). ^{1}H NMR ($CDCl_3$, 400 MHz, TMS) δ 1.04 (t, *J* = 7.2 Hz, 3H), 1.34-1.39 (m, 2H), 1.40-1.45 (m, 2H), 1.72 (s, 3H), 2.42 (s, 3H), 2.49 (q, *J* = 7.2 Hz, 2H), 3.80 (s, 2H), 3.97 (s, 2H), 7.29 (d, *J* = 8.0 Hz, 2H), 7.71 (d, *J* = 8.0 Hz, 2H). ^{13}C NMR ($CDCl_3$, 100 MHz, TMS) δ 7.1, 7.4, 17.0, 21.5, 32.7, 53.1, 54.6, 77.5, 96.6, 127.4, 129.5, 136.3, 143.4, 189.2, 206.4. IR (CH_2Cl_2) ν 2980, 2940, 2909, 2360, 2342, 2023, 1732, 1653, 1597, 1457, 1410, 1338, 1156, 1109, 1089, 1009, 893, 814, 769 cm^{-1}. HRMS (ESI) calcd. for $C_{18}H_{27}N_2O_3S$ $(M+NH_4)^+$: 351.1737, Found: 351.1732.

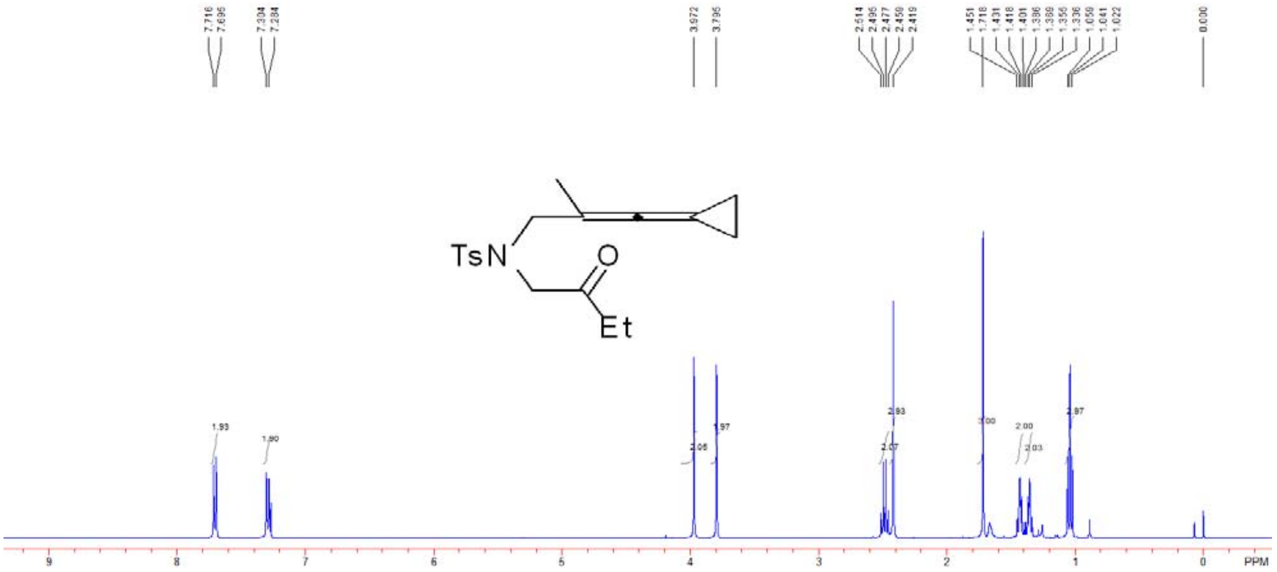

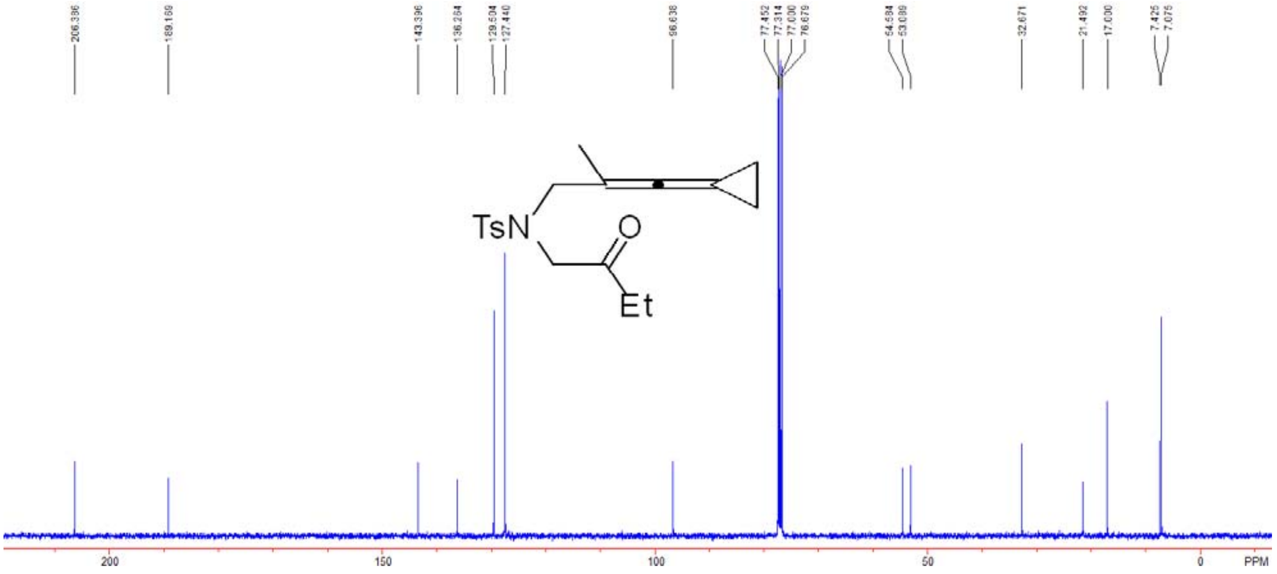

10. Characterization and spectra charts for products 3 5 and 6.

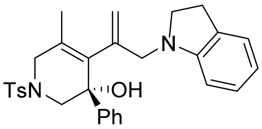

(S)-4-(3-(indolin-1-yl)prop-1-en-2-yl)-5-methyl-3-phenyl-1-tosyl-1,2,3,6-tetrahydropyridin-3-ol (3aa)

A white solid, 91% yield (45 mg). M. P. 105-107 °C. ^{1}H NMR ($CDCl_3$, 400 MHz, TMS) δ 1.87 (s, 3H), 2.40 (s, 3H), 2.85 (d, J = 11.6 Hz, 1H), 2.87-2.91 (m, 2H), 2.95-3.02 (m, 1H), 3.09 (dd, J_1 = 8.0 Hz, J_2 = 16.0 Hz, 1H), 3.25 (d, J = 14.8 Hz, 1H), 3.43 (d, J = 14.8 Hz, 1H), 3.45 (d, J = 16.4 Hz, 1H), 3.52 (d, J = 11.6 Hz, 1H), 3.89 (d, J = 16.4 Hz, 1H), 4.72 (s, 1H), 4.82 (brs, 1H), 5.18 (d, J = 0.8 Hz, 1H), 6.26 (d, J = 7.6 Hz, 1H), 6.70 (dd, J_1 = 7.2 Hz, J_2 = 7.6 Hz, 1H), 6.99 (dd, J_1 = 7.2 Hz, J_2 = 7.6 Hz, 1H), 7.06 (d, J = 7.2 Hz, 1H), 7.24-7.33 (m, 5H), 7.46 (d, J = 8.4 Hz, 2H), 7.62 (d, J = 8.8 Hz, 2H). ^{13}C NMR ($CDCl_3$, 100 MHz, TMS) δ 18.6, 21.5, 28.4, 49.6, 54.1, 57.0, 57.3, 72.6, 108.9, 119.2, 119.8, 124.4, 126.5, 127.13, 127.15, 127.75, 127.77, 129.7, 130.4, 130.5, 133.0, 136.1, 141.4, 143.1, 143.6, 151.5. IR (CH_2Cl_2) ν 2971, 2920, 2850, 2360, 2342, 1603, 1518, 1486, 1456, 1343, 1305, 1249, 1158, 1090, 1022, 988, 911, 873, 857, 811, 749, 705 cm^{-1}. HRMS (ESI) calcd. for $C_{30}H_{33}N_2O_3S$ $(M+H)^+$: 501.2206, Found: 501.2198. Enantiomeric excess was determined by HPLC with a Chiralcel AD-H column [λ = 254 nm; eluent: Hexane/Isopropanol = 80/20; Flow rate: 0.50 mL/min; t_{minor} = 26.03 min, t_{major} = 23.03 min; ee% > 99%; $[\alpha]^{20}_D$ = +26.2 (c 1.00, CH_2Cl_2)].

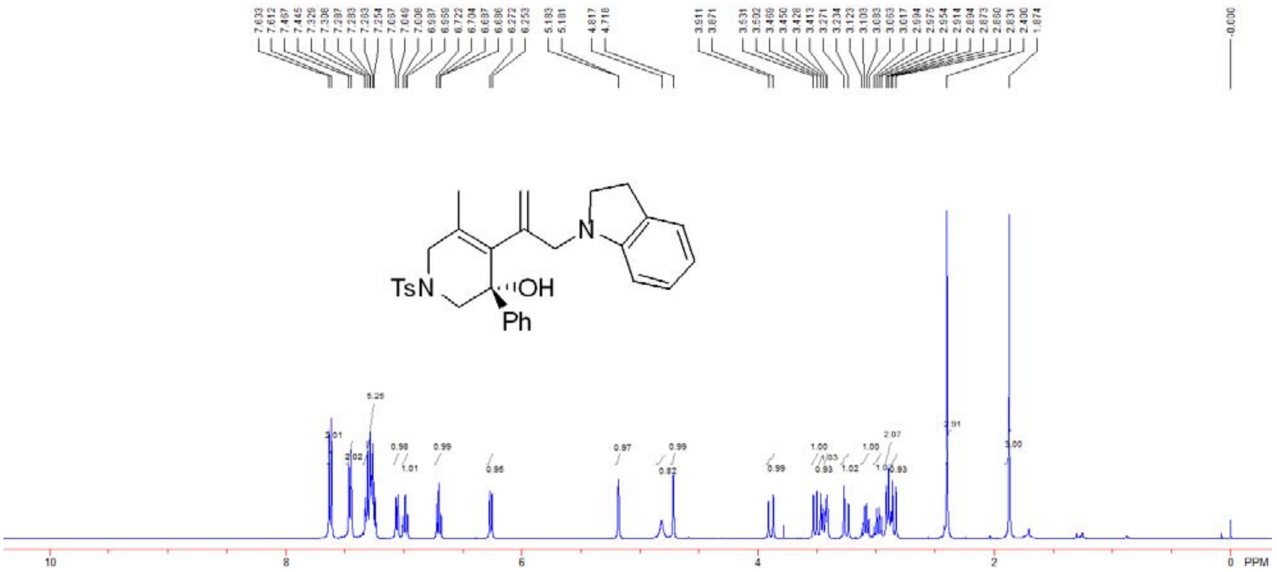

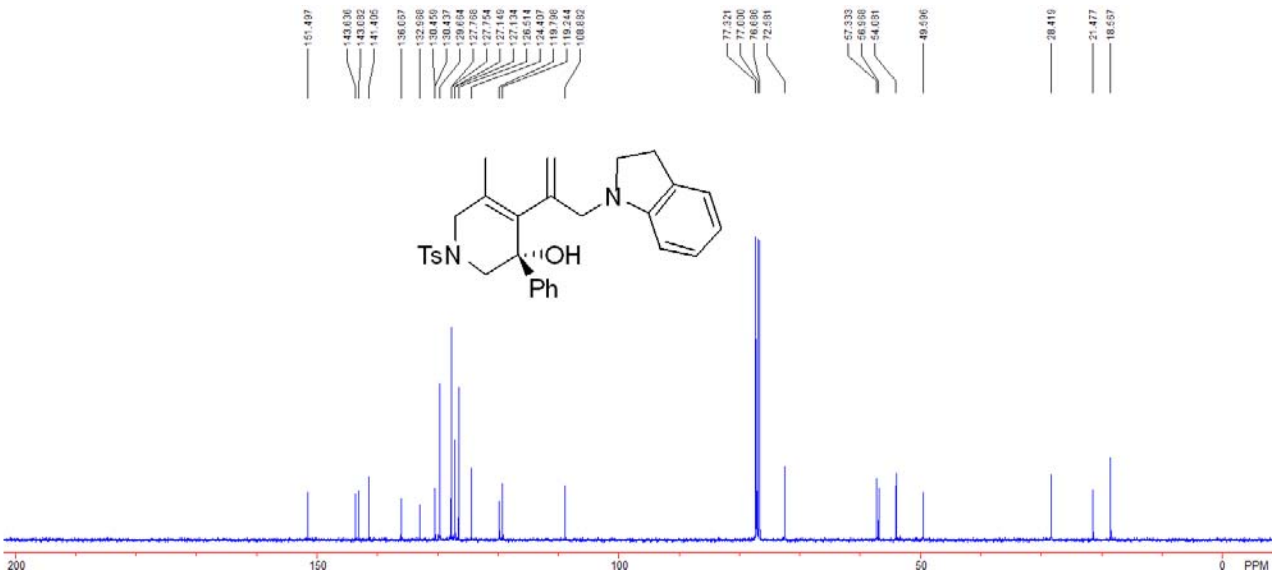

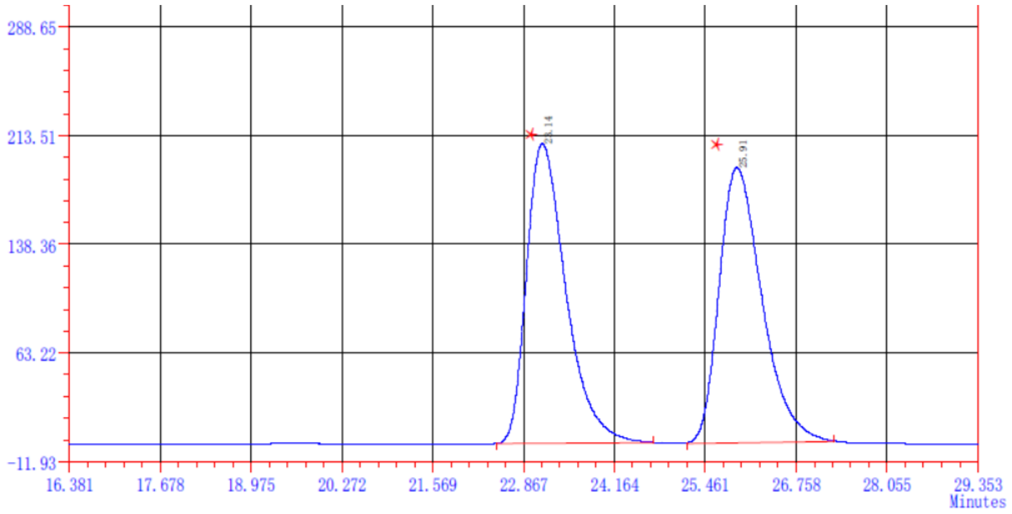

ID	组分名	保留时间	峰高	峰面积	浓度	拖尾因子	理论塔板
1		23.135	207424	8128209.9	49.9916	1.49	6947
2		25.913	190361	8130945.1	50.0084	1.41	7336
	Σ:		397785	16259155.0	100.0000		

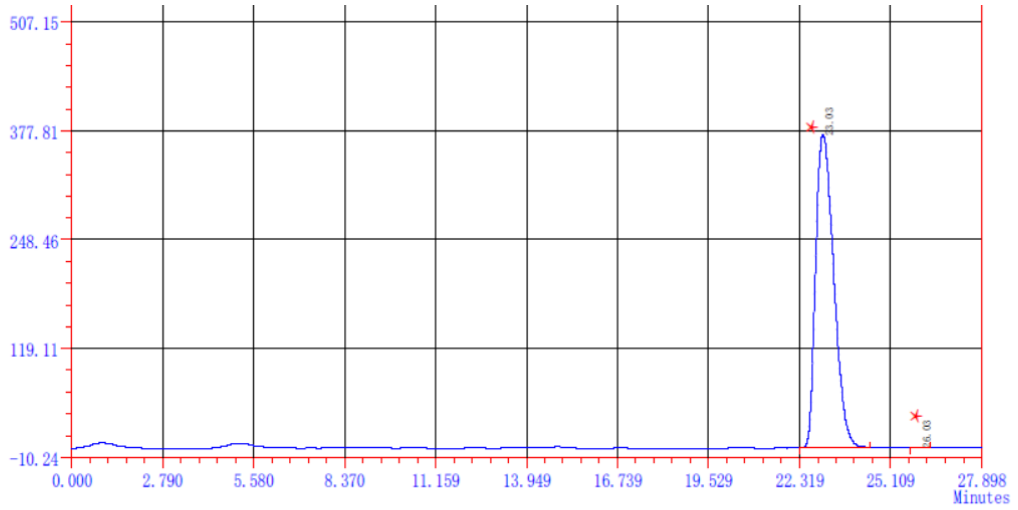

ID	组分名	保留时间	峰高	峰面积	浓度	拖尾因子	理论塔板
1		23.033	372212	14407504.2	99.9182	1.40	7057
2		26.033	425	11802.0	0.0818	0.97	17517
	Σ:		372637	14419306.2	100.0000		

Translation: Chiralcel AD-H column [λ = 254 nm; eluent: Hexane/Isopropanol = 80/20; Flow rate: 0.5 mL/min; t_{minor} = 26.03 min, t_{major} = 23.03 min; ee% > 99%].

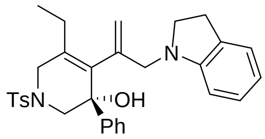

(S)-5-ethyl-4-(3-(indolin-1-yl)prop-1-en-2-yl)-3-phenyl-1-tosyl-1,2,3,6-tetrahydropyridin-3-ol (3ba)

A white solid, 82% yield (42 mg). M. P. 165-167 °C. ^{1}H NMR ($CDCl_3$, 400 MHz, TMS) δ 1.09 (t, *J* = 7.6 Hz, 3H), 2.16-2.25 (m, 1H), 2.30-2.39 (m, 1H), 2.41 (s, 3H), 2.87-2.93 (m, 3H), 3.03-3.07 (m, 2H), 3.34 (d, *J* = 15.6 Hz, 1H), 3.38 (d, *J* = 15.6 Hz, 1H), 3.46 (d, *J* = 11.6 Hz, 1H), 3.53 (d, *J* = 16.0 Hz, 1H), 3.88 (d, *J* = 16.0 Hz, 1H), 4.65 (brs, 1H), 4.71 (s, 1H), 5.12 (d, *J* = 1.2 Hz, 1H), 6.22 (d, *J* = 7.6 Hz, 1H), 6.69 (dd, J_1 = 7.2 Hz, J_2 = 7.2 Hz, 1H), 6.98 (dd, J_1 = 7.6 Hz, J_2 = 7.6 Hz, 1H), 7.05 (d, *J* = 7.2 Hz, 1H), 7.24-7.33 (m, 5H), 7.45 (d, *J* = 8.4 Hz, 2H), 7.63 (d, *J* = 8.4 Hz, 2H). ^{13}C NMR ($CDCl_3$, 100 MHz, TMS) δ 13.8, 21.5, 25.7, 28.4, 47.6, 54.1, 57.2, 57.4, 72.6, 108.6, 119.0, 119.1, 124.4, 126.5, 127.15, 127.17, 127.77, 127.80, 129.7, 130.3, 133.0, 135.5, 136.0, 141.1, 143.0, 143.7, 151.5. IR (CH_2Cl_2) ν 3065, 3029, 2970, 2924, 2845, 2360, 2342, 1605, 1489, 1447, 1348, 1249, 1168, 1152, 1090, 1050, 977, 909, 815, 757, 705 cm^{-1}. HRMS (ESI) calcd. for $C_{31}H_{35}N_2O_3S$ $(M+H)^+$: 515.2363, Found: 515.2354. Enantiomeric excess was determined by

HPLC with a Chiralcel AD-H column [λ = 254 nm; eluent: Hexane/Isopropanol = 80/20; Flow rate: 0.50 mL/min; t_{minor} = 20.73 min, t_{major} = 17.00 min; ee% > 99%; $[\alpha]^{20}_{D}$ = +91.8 (c 1.00, CH_2Cl_2)].

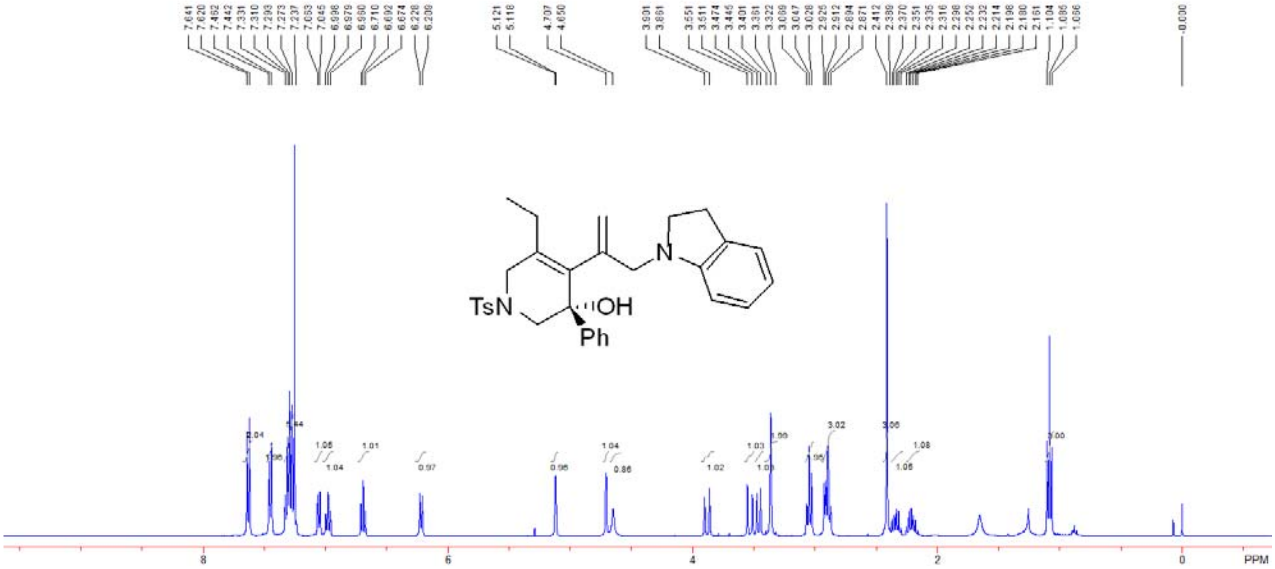

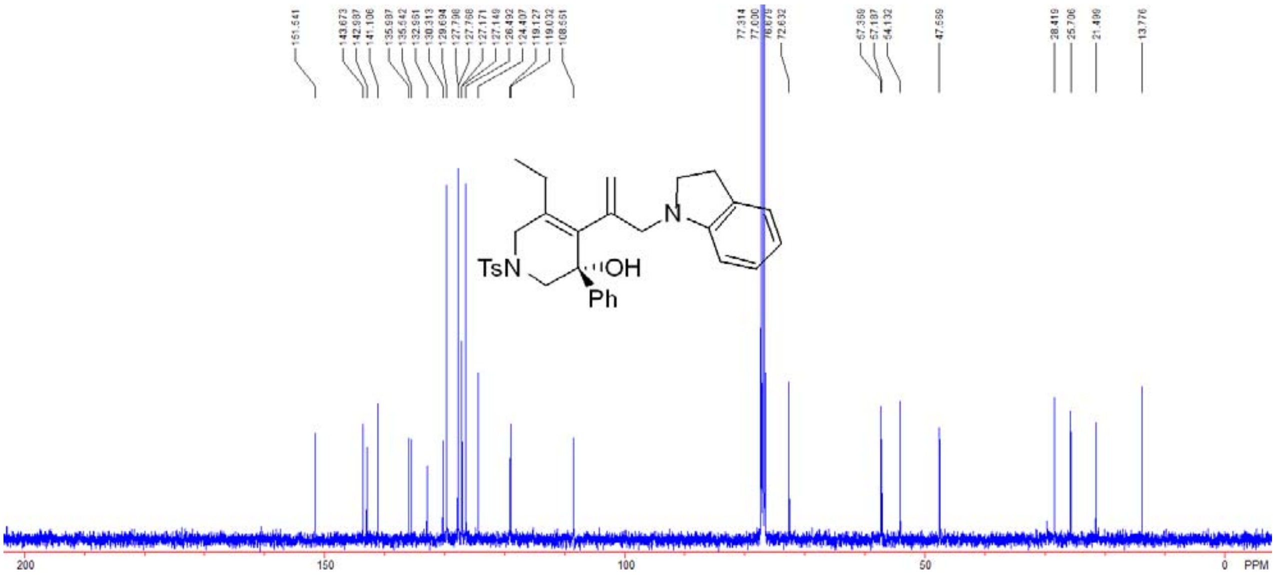

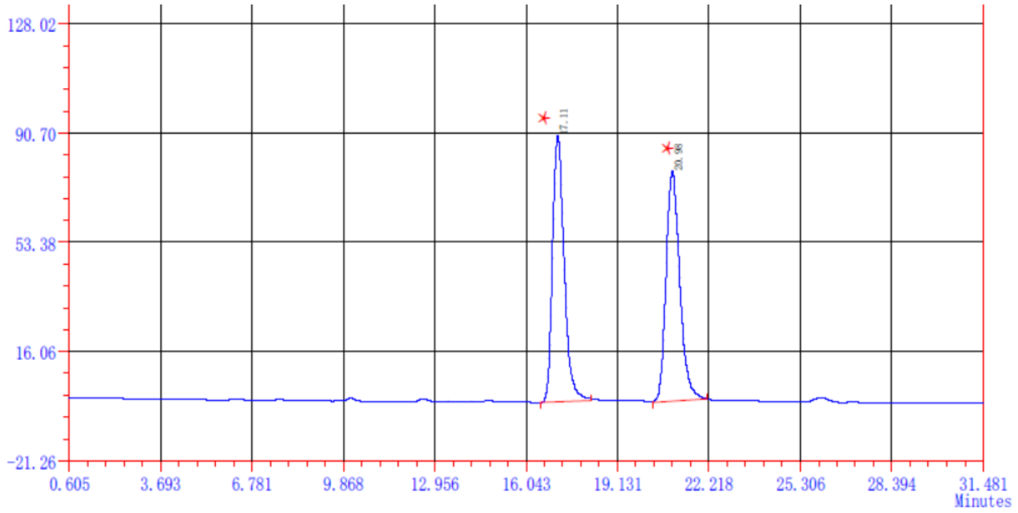

ID	组分名	保留时间	峰高	峰面积	浓度	拖尾因子	理论塔板
1		17.110	91176	2497112.0	49.5763	1.30	7779
4	组份 4	20.980	78763	2539798.6	50.4237	1.28	8437
	Σ:		169939	5036910.7	100.0000		

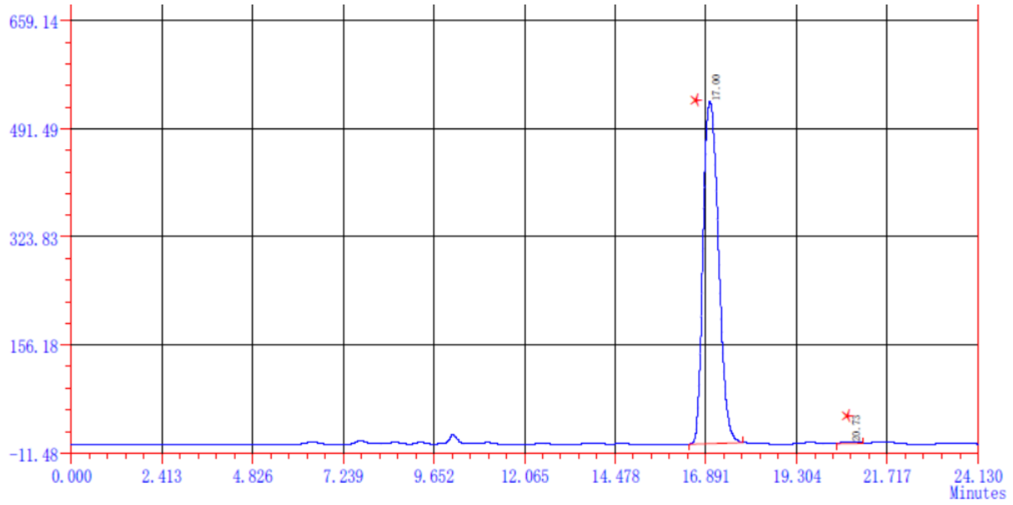

ID	组分名	保留时间	峰高	峰面积	浓度	拖尾因子	理论塔板
1		16.997	531452	15218302.4	99.5489	1.25	7022
4	组份 4	20.732	2958	68965.5	0.4511	1.05	15759
	Σ:		534410	15287268.0	100.0000		

Translation: Chiralcel AD-H column [λ = 254 nm; eluent: Hexane/Isopropanol = 80/20; Flow rate: 0.5 mL/min; t_{minor} = 20.73 min, t_{major} = 17.00 min; ee% > 99%].

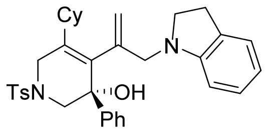

(S)-5-cyclohexyl-4-(3-(indolin-1-yl)prop-1-en-2-yl)-3-phenyl-1-tosyl-1,2,3,6-tetrahydropyridin-3-ol (3ca)

A white solid. 88% yield (50 mg). M. P. 85-87 °C. ^{1}H NMR (400 MHz, $CDCl_3$, TMS) δ 1.17-1.27 (m, 4H), 1.35-1.44 (m, 2H), 1.55-1.58 (m, 2H), 1.66-1.71 (m, 1H), 1.77-1.79 (m, 2H), 2.42 (s, 3H), 2.62-2.68 (m, 1H), 2.87-2.92 (m, 2H), 2.98-3.03 (m, 1H), 3.05-3.17 (m, 2H), 3.27-3.37 (m, 2H), 3.38 (d, J = 11.6 Hz, 1H), 3.49 (d, J = 16.0 Hz, 1H), 3.89 (d, J = 11.6 Hz, 1H), 4.64 (brs, 1H), 5.10 (s, 1H), 6.23 (brs, 1H), 6.64-6.70 (m, 1H), 6.98 (dd, J_1 = 7.2 Hz, J_2 = 7.6 Hz, 1H), 7.05 (d, J = 7.2 Hz, 1H), 7.26-7.33 (m, 5H), 7.45 (d, J = 8.4 Hz, 2H), 7.63 (d, J = 8.4 Hz, 2H). ^{13}C NMR (100 MHz, $CDCl_3$, TMS) δ 21.5, 25.8, 26.3, 26.4, 28.4, 31.3, 31.6, 41.4, 44.7, 54.2, 57.2, 72.6, 108.1, 109.4, 118.6, 124.4, 124.5, 126.4, 127.11, 127.72, 127.74, 129.7, 130.1, 132.9, 135.2, 138.2, 141.1, 142.8, 143.7, 151.7. IR (CH_2Cl_2): ν 3056, 3026, 2924, 2850, 2342, 1605, 1490, 1448, 1346, 1162, 1091, 982, 964, 909, 812, 733, 703, 669 cm^{-1}. HRMS (ESI) calcd. for $C_{35}H_{41}N_2O_3S$ $(M+H)^+$: 569.2832, Found: 569.2824. Enantiomeric excess was determined by HPLC with a Chiralcel AD-H column [λ = 254 nm; eluent: Hexane/Isopropanol = 80/20; Flow rate: 0.50 mL/min; t_{minor} = 29.55 min, t_{major} = 16.75 min; ee% >99%; $[\alpha]^{20}_{D}$ = +34.4 (c 1.00, CH_2Cl_2)].

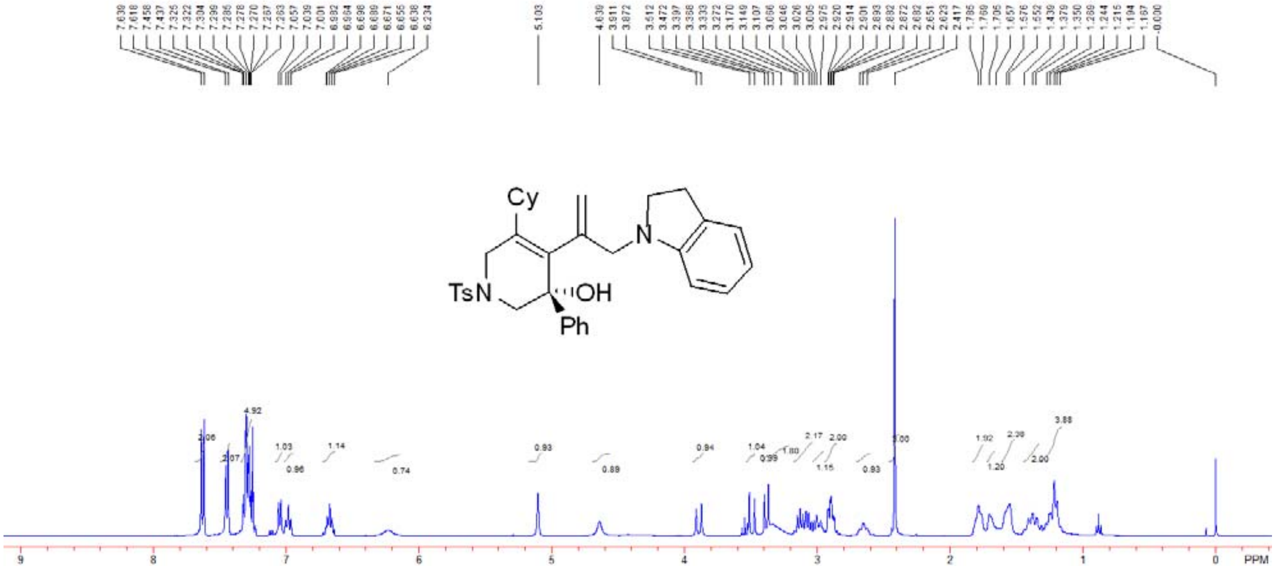

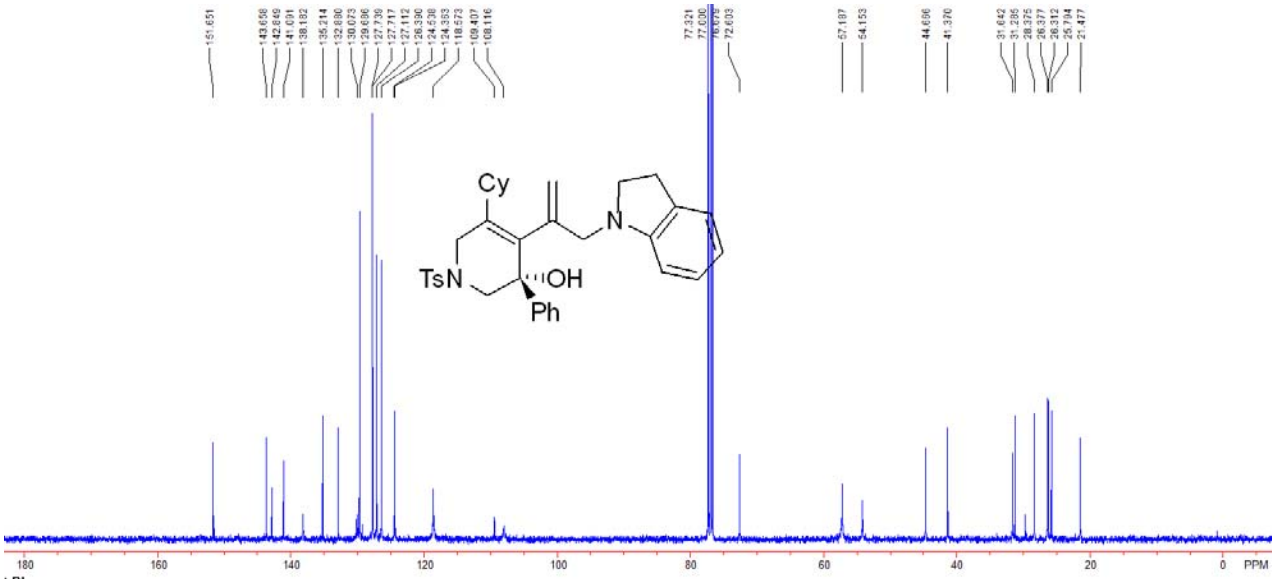

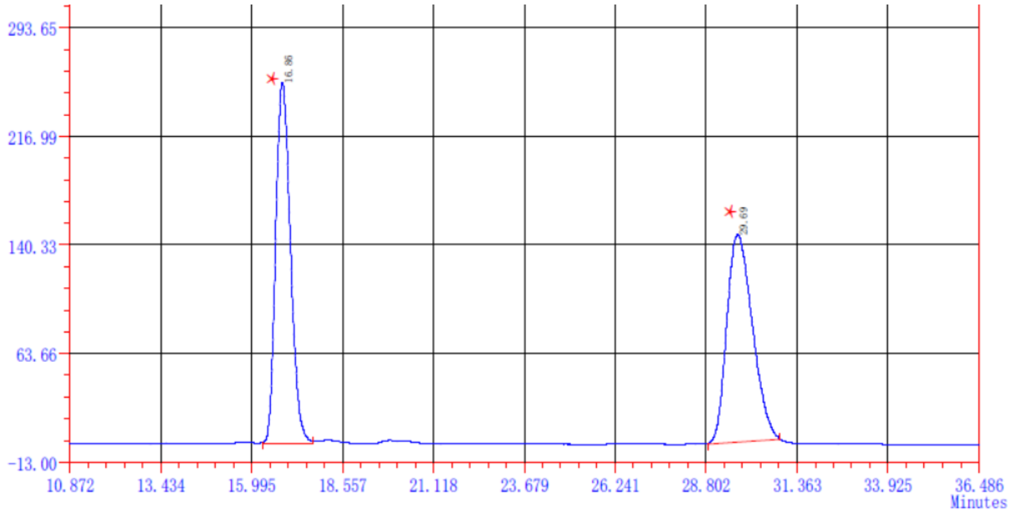

ID	组分名	保留时间	峰高	峰面积	浓度	拖尾因子	理论塔板
1		16.860	254221	7337550.1	49.8532	1.22	6801
2		29.690	146650	7380776.4	50.1468	1.32	6936
	Σ:		400871	14718326.5	100.0000		

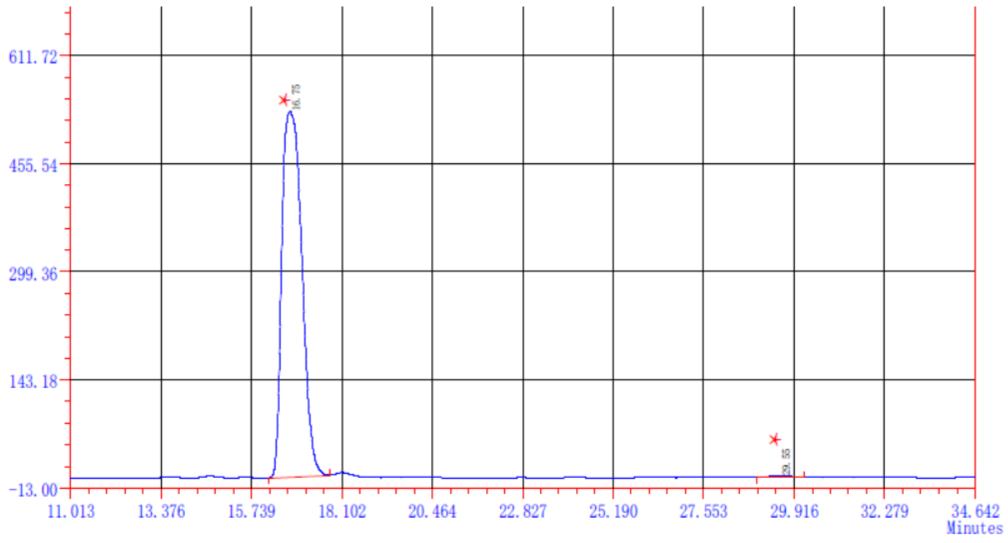

ID	组分名	保留时间	峰高	峰面积	浓度	拖尾因子	理论塔板
1		16.750	528803	19227915.5	99.3866	1.36	4229
2		29.550	2964	118680.2	0.6134	1.05	10855
	Σ:		531767	19346595.7	100.0000		

Translation: Chiralcel AD-H column [λ = 254 nm; eluent: Hexane/Isopropanol = 80/20; Flow rate: 0.50 mL/min; t_{minor} = 29.55 min, t_{major} = 16.75 min; ee% > 99%].

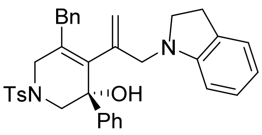

(S)-5-benzyl-4-(3-(indolin-1-yl)prop-1-en-2-yl)-3-phenyl-1-tosyl-1,2,3,6-tetrahydropyridin-3-ol (3da)

A light yellow oil. 74% yield (43 mg). ^{1}H NMR (400 MHz, $CDCl_3$, TMS) δ 2.38 (s, 3H), 2.88 (t, J = 8.0 Hz, 2H), 2.95 (d, J = 11.6 Hz, 1H), 3.03 (t, J = 8.0 Hz, 2H), 3.39-3.43 (m, 3H), 3.53 (d, J = 11.6 Hz, 1H), 3.61 (d, J = 15.2 Hz, 1H), 3.68 (d, J = 15.2 Hz, 1H), 3.85 (d, J = 16.4 Hz, 1H), 4.71 (brs, 1H), 5.16 (s, 1H), 5.28 (s, 1H), 6.20 (d, J = 8.0 Hz, 1H), 6.70 (dd, J_1 = 7.2 Hz, J_2 = 7.2 Hz, 1H), 6.97 (dd, J_1 = 7.6 Hz, J_2 = 7.6 Hz, 1H), 7.06 (d, J = 7.6 Hz, 1H), 7.16-7.28 (m, 6H), 7.30-7.36 (m, 4H), 7.50 (d, J = 8.0 Hz, 2H), 7.54 (d, J = 8.0 Hz, 2H). ^{13}C NMR (100 MHz, $CDCl_3$, TMS) δ 21.5, 28.4, 38.2, 48.0, 54.2, 57.3, 72.8, 108.6, 119.1, 119.8, 124.4, 126.5, 126.6, 127.2, 127.3, 127.7, 127.8, 128.2, 128.8, 129.6, 130.3, 132.4, 133.1, 138.4, 138.5, 141.1, 142.7, 143.6, 151.5. IR (CH_2Cl_2): ν 3398, 3090, 3059, 3031, 2970, 2918, 2851, 2360, 2342, 1734, 1653, 1624, 1598, 1541, 1492, 1449, 1344, 1306, 1248, 1091, 1049, 987, 971, 917, 864, 806, 765, 746, 703, 666 cm^{-1}. HRMS (ESI) calcd. for $C_{36}H_{37}N_2O_3S$ $(M+H)^+$: 577.2519, Found: 577.2510. Enantiomeric excess was determined by HPLC with a Chiralcel IC-H column [λ = 230 nm; eluent: Hexane/Isopropanol

= 80/20; Flow rate: 0.50 mL/min; t_{minor} = 24.79 min, t_{major} = 29.22 min; ee% > 99%; $[\alpha]^{20}_{D}$ = +29.8 (c 1.00, CH_2Cl_2)].

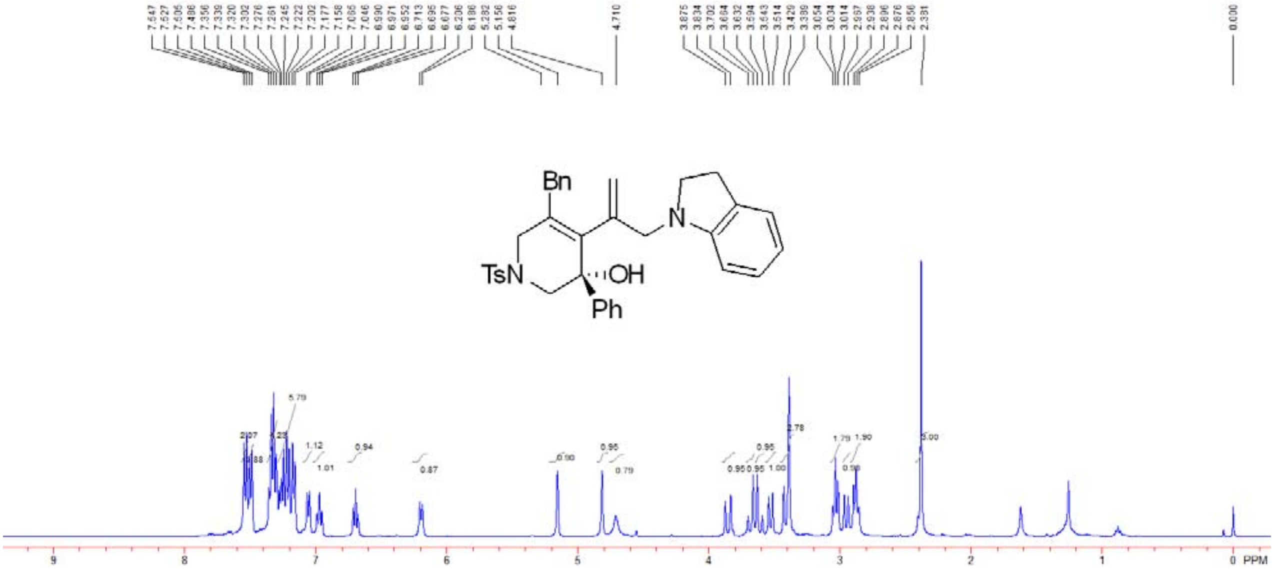

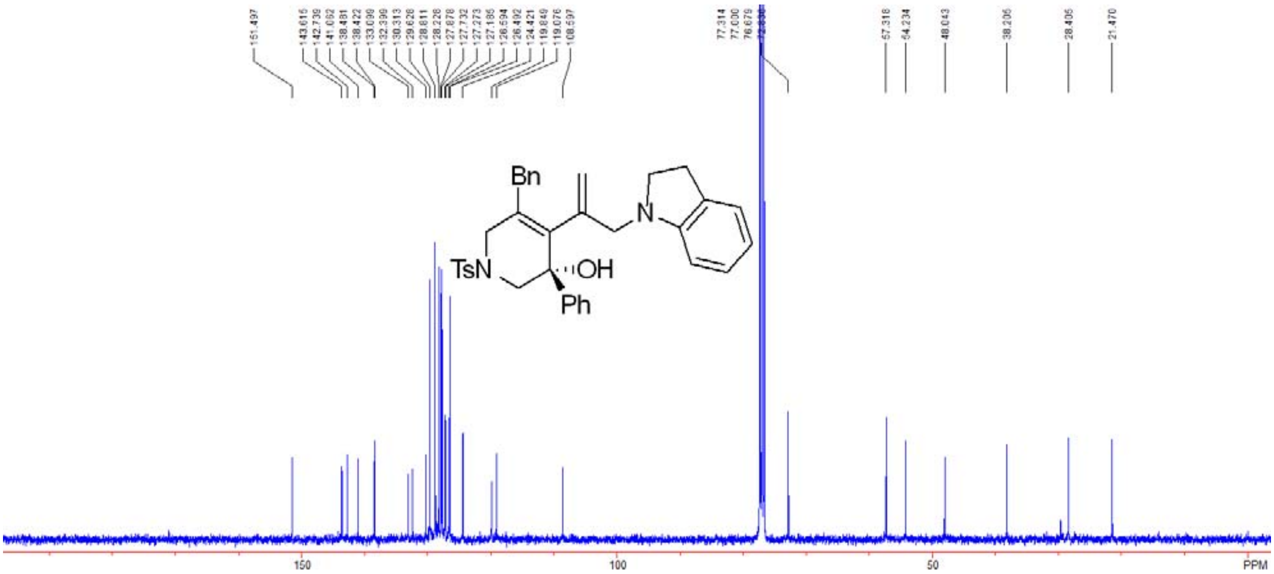

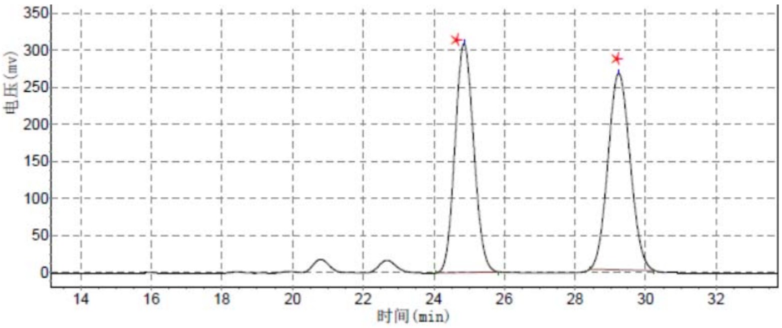

分析结果表

峰号	峰名	保留时间	峰高	峰面积	含量
1		24.855	308826.938	11411196.000	49.7170
2		29.238	264966.844	11541090.000	50.2830
总计			573793.781	22952286.000	100.0000

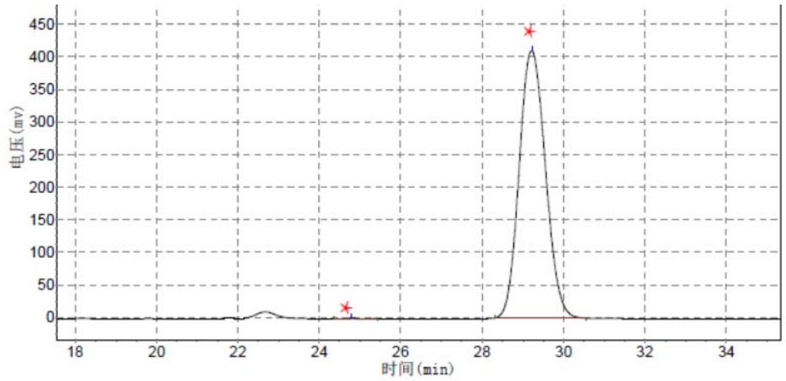

分析结果表

峰号	峰名	保留时间	峰高	峰面积	含量
1		24. 793	755. 697	24746. 498	0. 1346
2		29. 217	410381. 188	18355080. 000	99. 8654
总计			411136. 884	18379826. 498	100. 0000

Translation: Chiralcel AD-H column [λ = 254 nm; eluent: Hexane/Isopropanol = 80/20; Flow rate: 0.5 mL/min; t_{minor} = 24.79 min, t_{major} = 29.22 min; ee% > 99%].

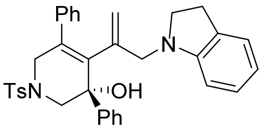

(S)-4-(3-(indolin-1-yl)prop-1-en-2-yl)-3,5-diphenyl-1-tosyl-1,2,3,6-tetrahydropyridin-3-ol (3ea)

A white solid. 80% yield (45 mg). M. P. 118-120 ^{o}C. ^{1}H NMR (400 MHz, $CDCl_3$, TMS) δ 2.42 (s, 3H), 2.80-2.89 (m, 4H), 2.96 (d, J = 15.6 Hz, 1H), 3.20 (d, J = 15.6 Hz, 1H), 3.25 (d, J = 11.6 Hz, 1H), 3.47 (d, J = 11.6 Hz, 1H), 3.83 (d, J = 16.4 Hz, 1H), 4.02 (d, J = 16.4 Hz, 1H), 4.42 (brs, 1H), 4.66 (s, 1H), 4.92 (d, J = 1.6 Hz, 1H), 5.80 (d, J = 7.6 Hz, 1H), 6.61 (dd, J_1 = 7.6 Hz, J_2 = 8.0 Hz, 1H), 6.87 (dd, J_1 = 6.8 Hz, J_2 = 7.6 Hz, 1H), 6.99 (d, J = 6.8 Hz, 1H), 7.24-7.37 (m, 11H), 7.55 (d, J = 8.4 Hz, 2H), 7.61 (d, J = 8.4 Hz, 2H). ^{13}C NMR (100 MHz, $CDCl_3$, TMS) δ 21.5, 28.3, 50.0, 53.8, 56.5, 57.0, 73.1, 107.8, 118.4, 120.4, 124.3, 126.4, 127.1, 127.4, 127.8, 128.0, 128.3, 128.5, 129.8, 129.9, 132.9, 134.6, 138.2, 138.8, 140.1, 142.7, 143.8, 151.6. IR (CH_2Cl_2): ν 3392, 3059, 3031, 2976, 2918, 2851, 2360, 2342, 1734, 1598, 1492, 1449, 1343, 1307, 1159, 1091, 1043, 1020, 987, 972, 939, 900, 874, 806, 764, 702 cm^{-1}. HRMS (ESI) calcd. for $C_{35}H_{35}N_2O_3S$ $(M+H)^+$: 563.2363, Found: 563.2355. Enantiomeric excess was determined by HPLC with a Chiralcel AD-H column [λ = 230 nm; eluent: Hexane/Isopropanol = 80/20; Flow rate: 0.50 mL/min; t_{minor} = 36.01 min, t_{major} = 14.31 min; ee% > 99%; $[\alpha]^{20}_{D}$ = +50.0 (c 1.00, CH_2Cl_2)].

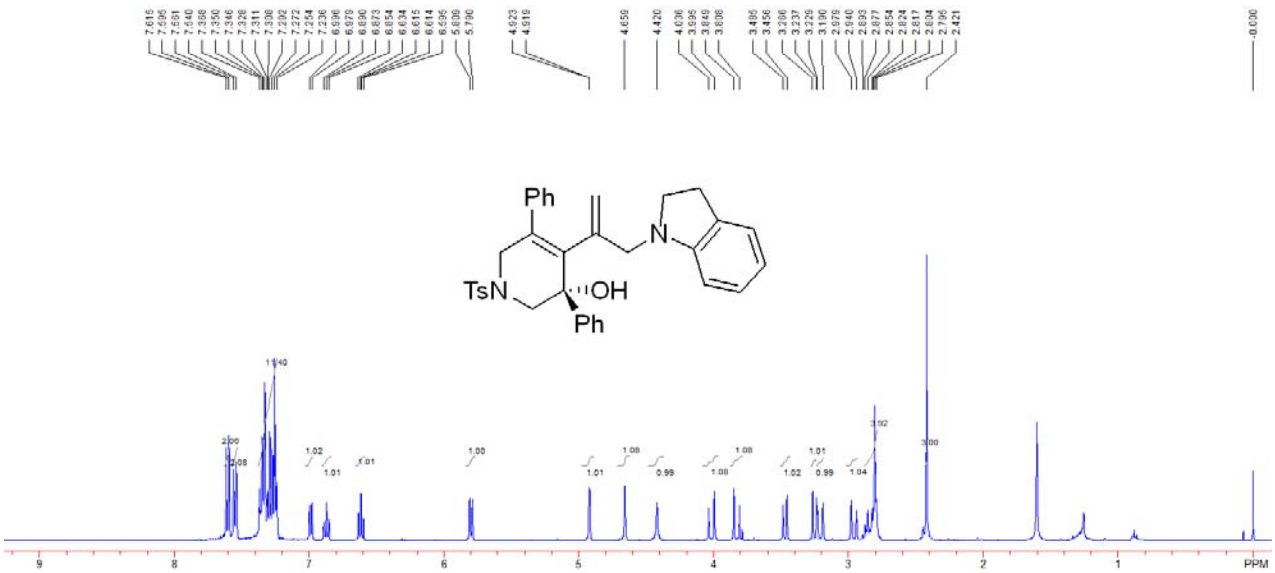

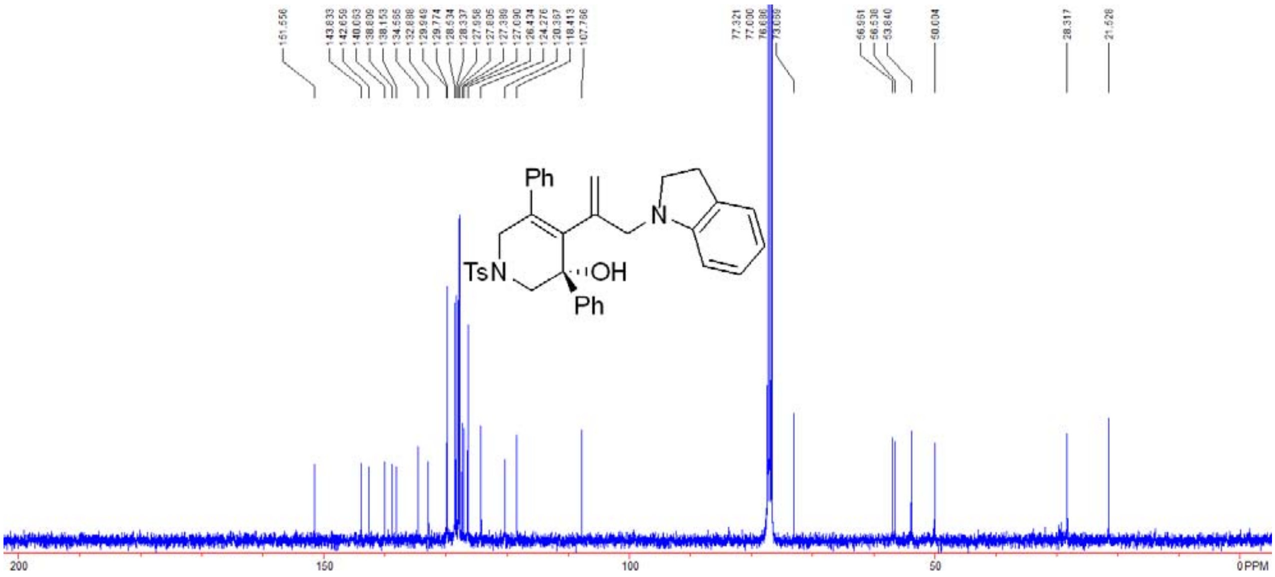

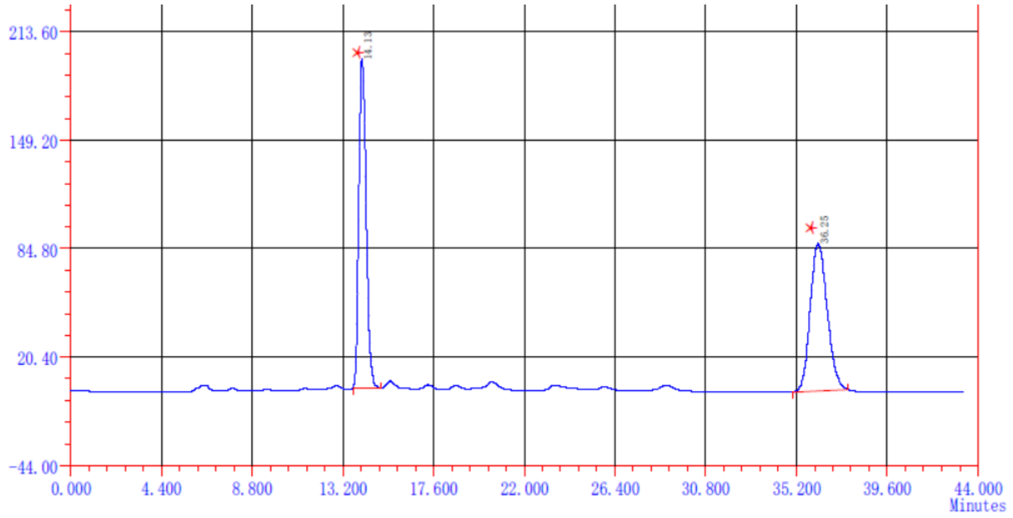

ID	组分名	保留时间	峰高	峰面积	浓度	拖尾因子	理论塔板
1	组份 1	14.132	195616	4874077.3	49.2454	1.24	6411
2		36.248	87729	5023443.5	50.7546	1.17	7987
	Σ:		283345	9897520.8	100.0000		

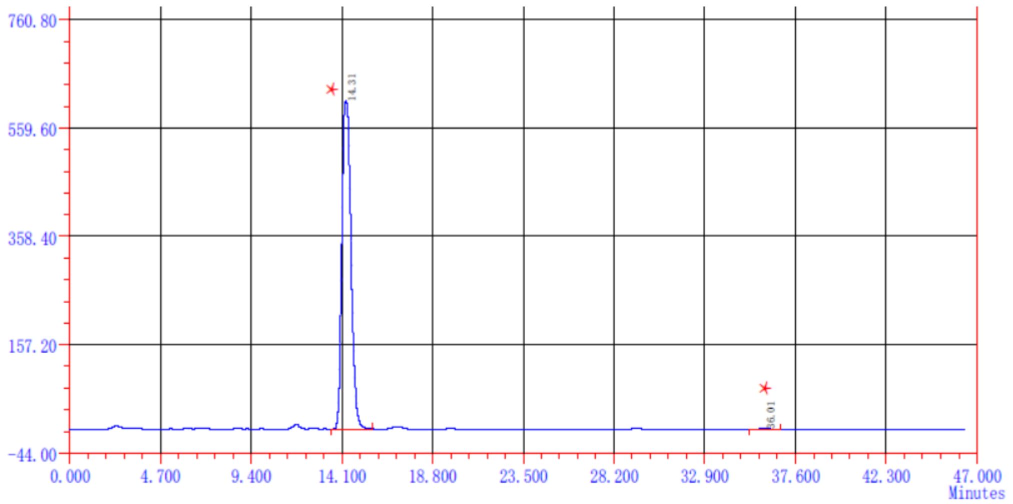

ID	组分名	保留时间	峰高	峰面积	浓度	拖尾因子	理论塔板
1	组份 1	14.313	609897	20117466.6	99.5830	1.21	3753
2		36.013	1426	84239.0	0.4170	1.02	7407
	Σ:		611323	20201705.6	100.0000		

Translation: Chiralcel AD-H column [λ = 230 nm; eluent: Hexane/Isopropanol = 80/20; Flow rate: 0.5 mL/min; t_{minor} = 36.01 min, t_{major} = 14.31 min; ee% > 99%].

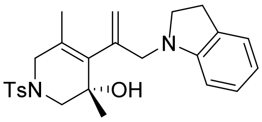

(S)-4-(3-(indolin-1-yl)prop-1-en-2-yl)-3,5-dimethyl-1-tosyl-1,2,3,6-tetrahydropyridin-3-ol (3fa)

A white solid. 80% yield (35 mg). M. P. 166-168 °C. ^{1}H NMR (400 MHz, $CDCl_3$, TMS) δ 1.31 (s, 3H), 1.68 (s, 3H), 2.43 (s, 3H), 2.83 (d, J = 11.6 Hz, 1H), 2.95-2.99 (m, 2H), 3.22 (d, J = 11.6 Hz, 1H), 3.29-3.35 (m, 2H), 3.41 (dd, J_1 = 8.4 Hz, J_2 = 16.8 Hz, 1H), 3.61 (d, J = 16.0 Hz, 1H), 3.67 (d, J = 16.0 Hz, 1H), 3.73 (d, J = 16.0 Hz, 1H), 4.84 (s, 1H), 5.37 (d, J = 1.6 Hz, 1H), 6.50 (d, J = 8.0 Hz, 1H), 6.68 (dd, J_1 = 7.2 Hz, J_2 = 7.6 Hz, 1H), 7.03 (dd, J_1 = 7.2 Hz, J_2 = 8.0 Hz, 1H), 7.08 (d, J = 7.2 Hz, 1H), 7.34 (d, J = 8.0 Hz, 2H), 7.69 (d, J = 8.0 Hz, 2H). ^{13}C NMR (100 MHz, $CDCl_3$, TMS) δ 17.7, 21.5, 25.0, 28.5, 29.6, 49.5, 54.4, 56.1, 56.8, 68.5, 107.7, 117.3, 118.4, 124.5, 127.3, 127.5, 127.8, 129.7, 130.0, 132.6, 137.2, 141.8, 143.8, 151.9. IR (CH_2Cl_2): ν 2979, 2919, 2849, 2360, 1979, 1065, 1488, 1452, 1400, 1340, 1305, 1246, 1157, 1089, 1043, 1014, 988, 924, 865, 829, 815, 748, 707, 661 cm^{-1}. HRMS (ESI) calcd. for $C_{25}H_{31}N_2O_3S$ $(M+H)^+$: 439.2050, Found: 439.2044. Enantiomeric excess was determined by HPLC with a Chiralcel IC-H column [λ = 254 nm; eluent: Hexane/Isopropanol = 80/20; Flow rate: 0.50 mL/min; t_{minor} = 44.58 min, t_{major} = 40.36 min; ee% > 99%; $[\alpha]^{20}_{D}$ = -22.0 (c 1.00, CH_2Cl_2)].

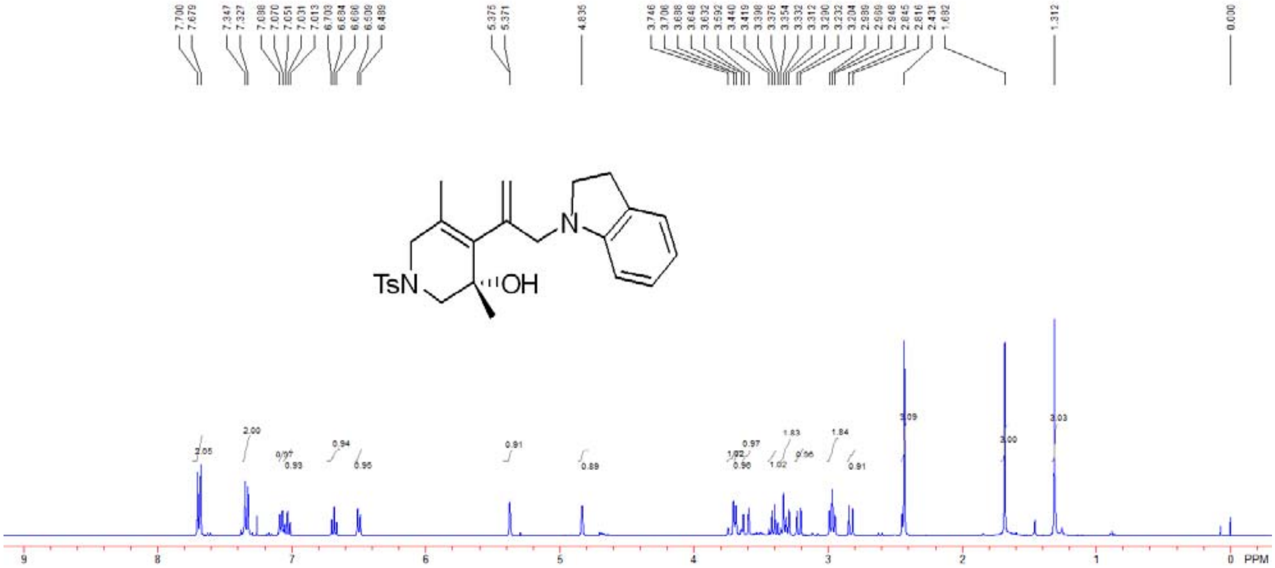

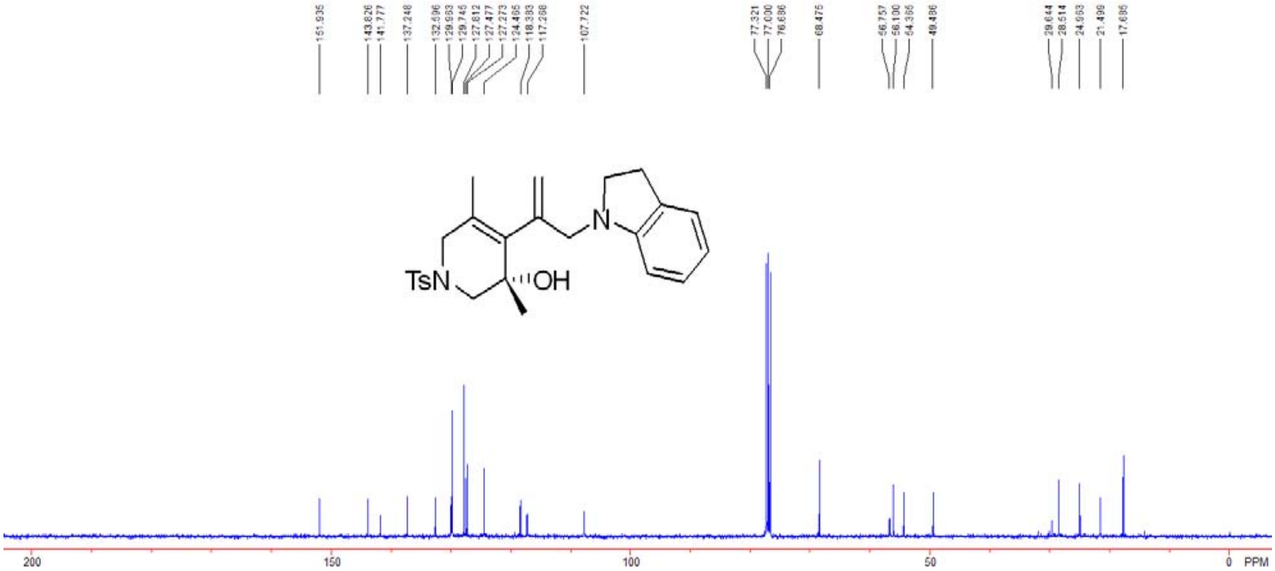

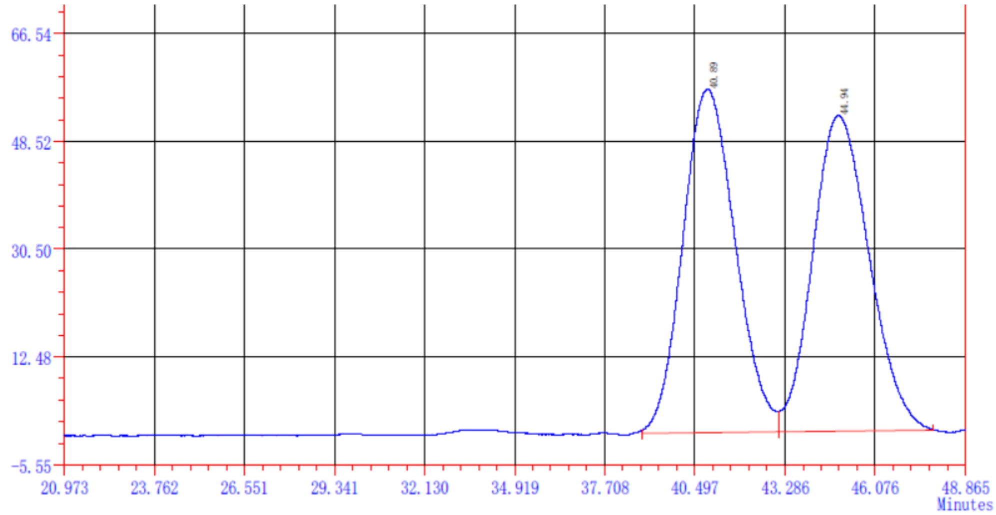

ID	组分名	保留时间	峰高	峰面积	浓度	拖尾因子	理论塔板
1		40. 893	57424	6486602. 1	50. 1998	1. 16	2612
2		44. 942	52830	6434972. 9	49. 8002	1. 11	2713
	Σ:		110254	12921574. 9	100. 0000		

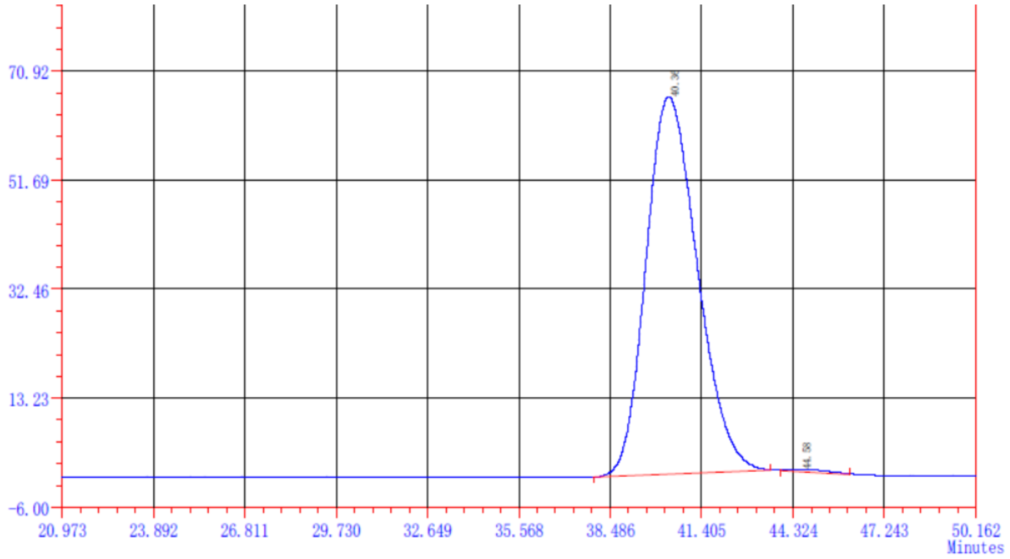

ID	组分名	保留时间	峰高	峰面积	浓度	拖尾因子	理论塔板
1		40.358	66597	7606599.5	99.5025	1.24	2488
2		44.582	403	38032.2	0.4975	1.18	4448
	Σ:		67000	7644631.7	100.0000		

Translation: Chiralcel IC-H column [λ = 254 nm; eluent: Hexane/Isopropanol = 80/20; Flow rate: 0.5 mL/min; t_{minor} = 44.58 min, t_{major} = 40.36 min; ee% =99%].

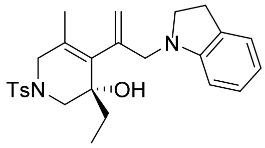

(S)-3-ethyl-4-(3-(indolin-1-yl)prop-1-en-2-yl)-5-methyl-1-tosyl-1,2,3,6-tetrahydropyridin-3-ol (3ga)

A light yellow oil. 85% yield (38 mg). ^{1}H NMR (400 MHz, $CDCl_3$, TMS) δ 0.94 (t, J = 7.2 Hz, 3H), 1.62-1.65 (m, 1H), 1.69 (s, 3H), 1.73-1.80 (m, 1H), 2.43 (s, 3H), 2.94-3.00 (m, 3H), 3.08 (d, J = 11.2 Hz, 1H), 3.30 (dd, J_1 = 8.8 Hz, J_2 = 16.8 Hz, 1H), 3.38-3.42 (m, 2H), 3.50 (d, J = 16.0 Hz, 1H), 3.66 (d, J = 15.6 Hz, 1H), 3.72 (d, J = 15.6 Hz, 1H), 4.86 (s, 1H), 5.38 (s, 1H), 6.49 (d, J = 8.0 Hz, 1H), 6.68 (dd, J_1 = 7.2 Hz, J_2 = 7.6 Hz, 1H), 7.02 (dd, J_1 = 7.6 Hz, J_2 = 8.0 Hz, 1H), 7.08 (d, J = 7.2 Hz, 1H), 7.34 (d, J = 8.0 Hz, 2H), 7.69 (d, J = 8.0 Hz, 2H). ^{13}C NMR (125 MHz, $CDCl_3$, TMS) δ 7.8, 17.9, 21.5, 28.5, 30.3, 49.5, 52.5, 54.4, 57.1, 70.7, 107.9, 117.7, 118.5, 124.5, 127.3, 127.8, 128.0, 129.7, 130.0, 132.6, 137.6, 141.8, 143.8, 151.9. IR (CH_2Cl_2): ν 2979, 2919, 2849, 2360, 1979, 1065, 1488, 1452, 1400, 1340, 1305, 1246, 1157, 1089, 1043, 1014, 988, 924, 865, 829, 815, 748, 707, 661 cm^{-1}. HRMS (ESI) calcd. for $C_{26}H_{33}N_2O_3S$ $(M+H)^+$: 453.2206, Found:

453.2200. Enantiomeric excess was determined by HPLC with a Chiralcel IC-H column [λ = 254 nm; eluent: Hexane/Isopropanol = 80/20; Flow rate: 0.50 mL/min; t_{minor} = 31.51 min, t_{major} = 35.57 min; ee% > 99%; $[\alpha]^{20}_{D}$ = +16.4 (c 1.00, CH_2Cl_2)].

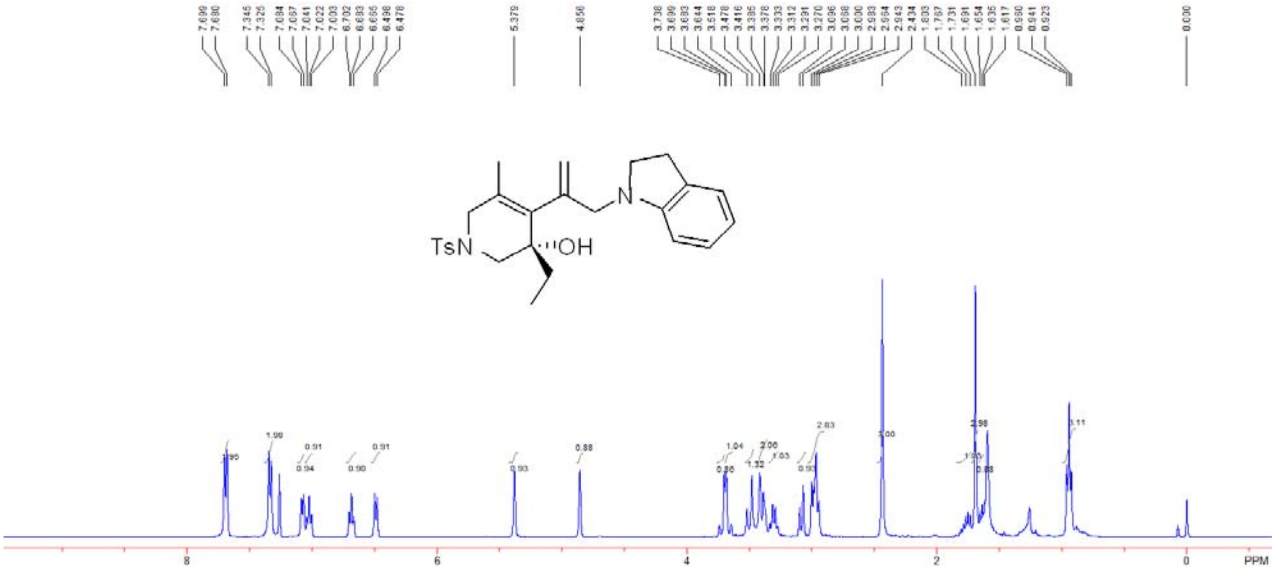

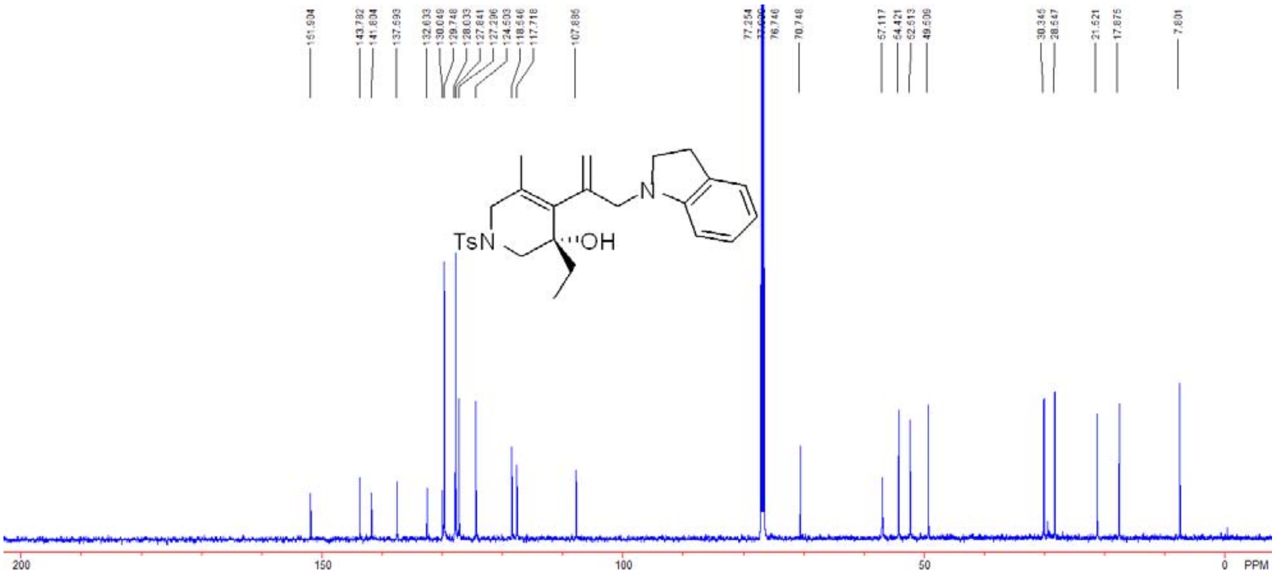

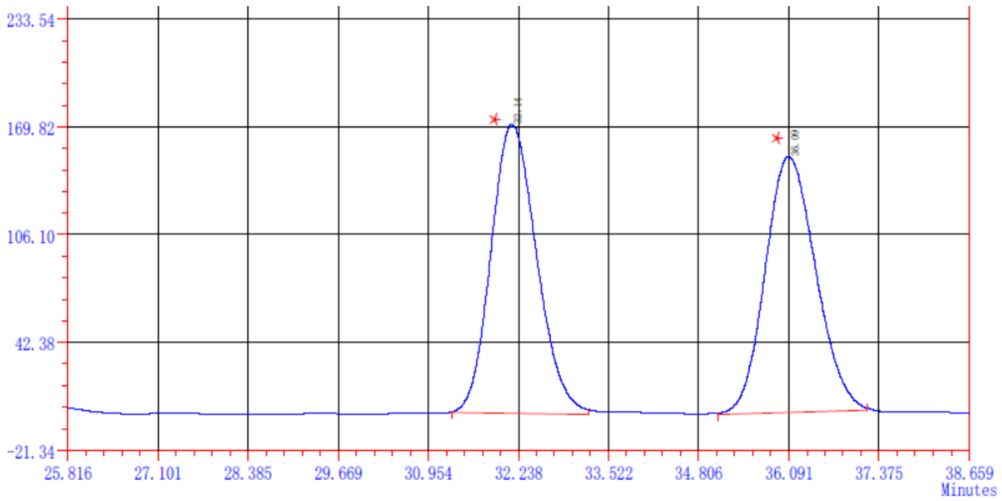

ID	组分名	保留时间	峰高	峰面积	浓度	拖尾因子	理论塔板
1		32. 142	171130	7749349. 2	50. 5432	1. 16	10041
2		36. 093	151550	7582774. 5	49. 4568	1. 15	10371
	Σ:		322680	15332123. 7	100. 0000		

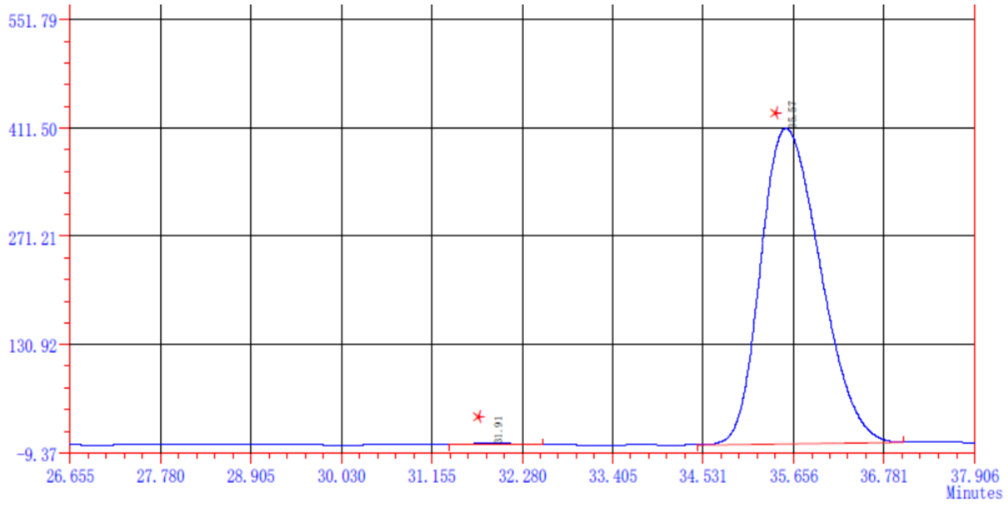

ID	组分名	保留时间	峰高	峰面积	浓度	拖尾因子	理论塔板
1		31. 907	1811	70901. 7	0. 3348	1. 09	13238
2		35. 565	408917	21108576. 2	99. 6652	1. 27	9461
	Σ:		410728	21179477. 8	100. 0000		

Translation: Chiralcel IC-H column [λ = 254 nm; eluent: Hexane/Isopropanol = 80/20; Flow rate: 0.5 mL/min; t_{minor} = 31.91 min, t_{major} = 35.57 min; ee% > 99%].

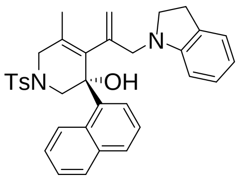

(S)-4-(3-(indolin-1-yl)prop-1-en-2-yl)-5-methyl-3-(naphthalen-1-yl)-1-tosyl-1,2,3,6-tetrahydro pyridin-3-ol (3ha)

A light yellow oil. 87% yield (48 mg). ^{1}H NMR (400 MHz, $CDCl_3$, TMS) δ 1.92 (s, 3H), 2.39 (s, 3H), 2.87-3.00 (m, 4H), 3.07 (dd, J_1 = 8.0 Hz, J_1 = 15.6 Hz, 1H), 3.26 (d, J = 14.8 Hz, 1H), 3.46 (d, J = 16.8 Hz, 1H), 3.50 (d, J = 16.8 Hz, 1H), 3.58 (d, J = 11.6 Hz, 1H), 3.96 (d, J = 15.6 Hz, 1H), 4.76 (s, 1H), 5.09 (brs, 1H), 5.15 (d, J = 0.8 Hz, 1H), 6.19 (d, J = 7.6 Hz, 1H), 6.69 (dd, J_1 = 7.2 Hz, J_1 = 7.6 Hz, 1H), 6.86 (dd, J_1 = 7.6 Hz, J_1 = 8.0 Hz, 1H), 7.05 (d, J = 7.2 Hz, 1H), 7.24 (d, J = 8.0 Hz, 2H), 7.42 (dd, J_1 = 1.6 Hz, J_1 = 8.8 Hz, 1H), 7.46-7.62 (m, 2H), 7.61 (d, J = 8.0 Hz, 2H), 7.74 (d, J = 8.4 Hz, 1H), 7.81-7.84 (m, 2H), 8.05 (s, 1H). ^{13}C NMR (125 MHz, $CDCl_3$, TMS) δ 18.6, 21.5, 28.4, 49.6, 54.2, 57.18, 57.22, 72.8, 109.1, 119.4, 120.1, 124.4, 124.9, 125.4, 125.9, 126.0, 127.1, 127.2, 127.4, 127.7, 128.2, 129.6, 130.5, 130.8, 132.5, 132.9, 133.0, 135.9, 140.7, 141.4, 143.6, 151.4. IR (CH_2Cl_2): ν 3488, 3062, 2970, 2922, 2852, 2360, 2342, 1600, 1574, 1506, 1492, 1448, 1398, 1342, 1305, 1249, 1158, 1122, 1090, 1046, 989, 968, 910, 863, 814, 791, 763, 744, 701 cm^{-1}. HRMS (ESI) calcd. for $C_{34}H_{35}N_2O_3S$ $(M+H)^+$: 551.2363, Found: 551.2354. Enantiomeric excess was determined by HPLC with a Chiralcel AD-H column [λ = 254 nm; eluent: Hexane/Isopropanol = 70/30; Flow rate: 0.50 mL/min; t_{minor} = 21.19 min, t_{major} = 31.24 min; ee% > 99%; $[\alpha]^{20}_D$ = +78.3 (c 1.00, CH_2Cl_2)].

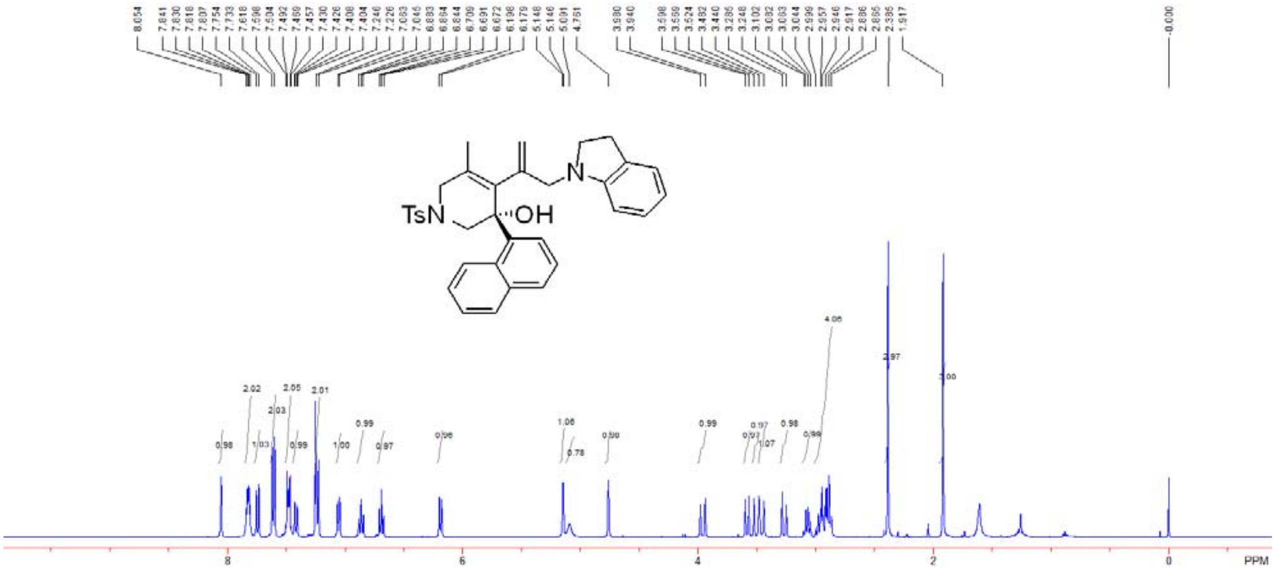

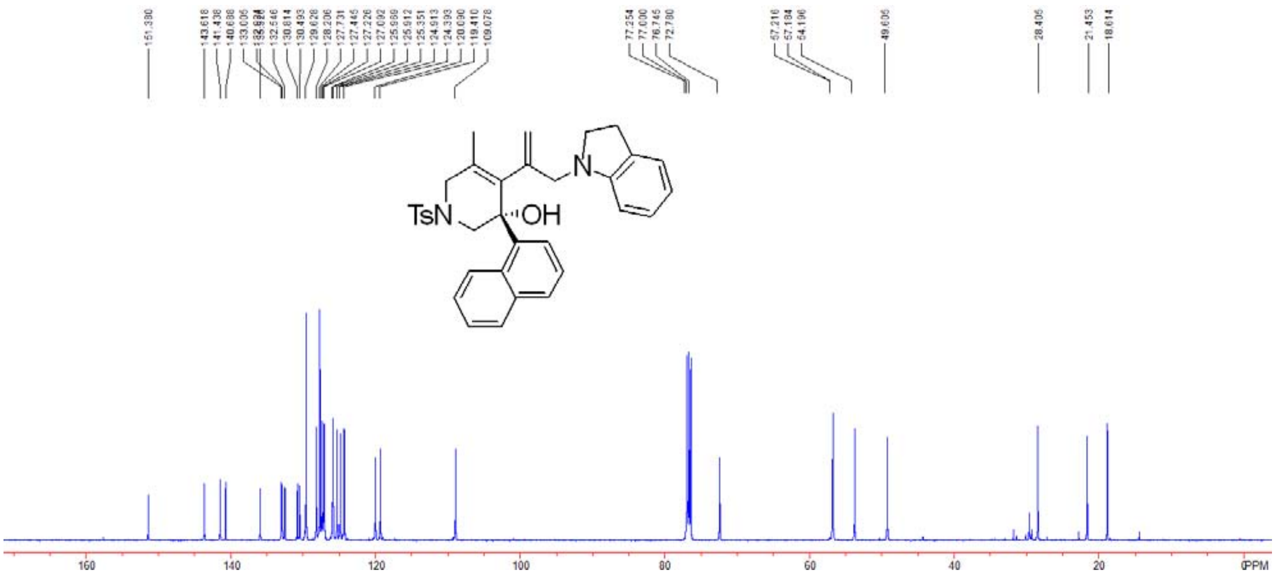

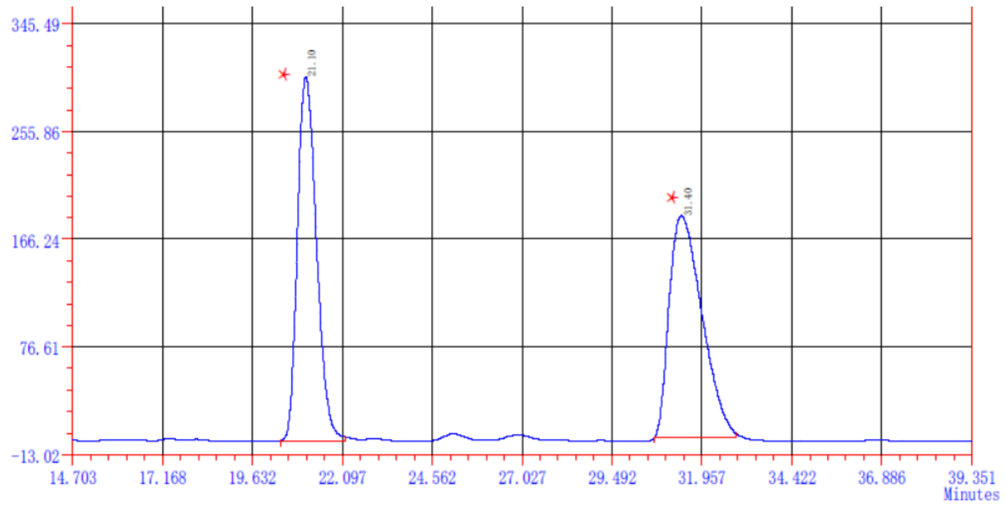

ID	组分名	保留时间	峰高	峰面积	浓度	拖尾因子	理论塔板
4	组份 4	21.100	303411	11079994.8	49.0722	1.26	6654
2		31.402	184421	11498986.4	50.9278	1.53	5055
	Σ:		487832	22578981.3	100.0000		

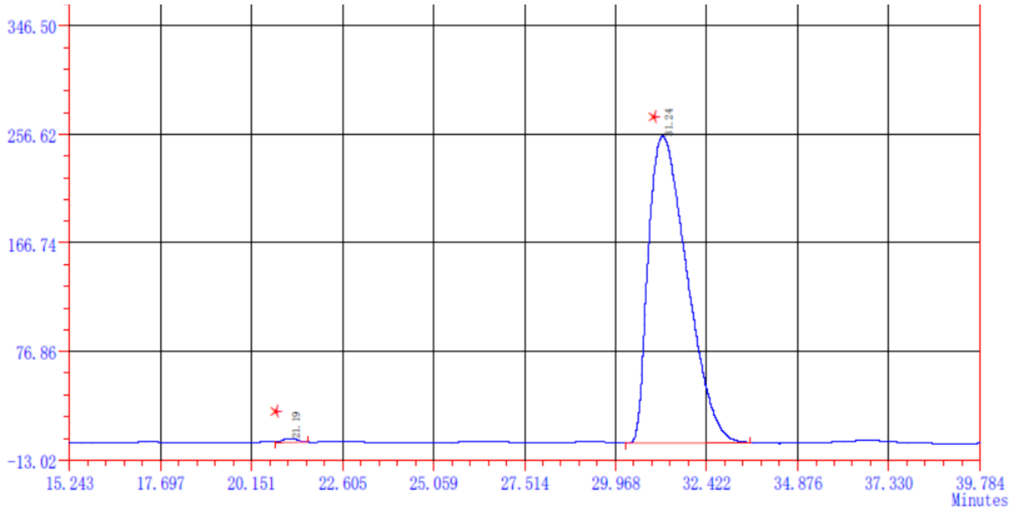

ID	组分名	保留时间	峰高	峰面积	浓度	拖尾因子	理论塔板
4	组份 4	21.193	3002	83603.8	0.4519	1.03	11542
2		31.237	254465	18418157.7	99.5481	1.62	3712
	Σ:		257467	18501761.5	100.0000		

Translation: Chiralcel AD-H column [λ = 254 nm; eluent: Hexane/Isopropano] l = 70/30; Flow rate: 0.5 mL/min; t_{minor} = 21.19 min, t_{major} = 31.24 min; ee% > 99%].

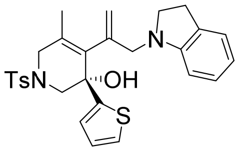

(S)-4-(3-(indolin-1-yl)prop-1-en-2-yl)-5-methyl-3-(thiophen-2-yl)-1-tosyl-1,2,3,6-tetrahydropyridin-3-ol (3ia)

A light yellow solid. 77% yield (39 mg). M. P. 99-101 °C. ^{1}H NMR (400 MHz, $CDCl_3$, TMS) δ 1.82 (s, 3H), 2.41 (s, 3H), 2.90-2.94 (m, 2H), 3.09 (dd, J_1 = 8.8 Hz, J_2 = 16.4 Hz, 1H), 3.18 (dd, J_1 = 8.8 Hz, J_2 = 16.4 Hz, 1H), 3.26 (d, J = 11.2 Hz, 1H), 3.30 (d, J = 11.2 Hz, 1H), 3.43 (d, J = 15.2 Hz, 1H), 3.47 (d, J = 15.2 Hz, 1H), 3.61 (d, J = 16.4 Hz, 1H), 3.65 (d, J = 16.4 Hz, 1H), 4.74 (d, J = 1.6 Hz, 1H), 5.26 (d, J = 1.6 Hz, 1H), 5.61 (brs, 1H), 6.41 (d, J = 8.0 Hz, 1H), 6.75 (dd, J_1 = 6.8 Hz, J_2 = 7.6 Hz, 1H), 6.90 (dd, J_1 = 1.2 Hz, J_2 = 7.6 Hz, 1H), 6.95 (dd, J_1 = 7.6 Hz, J_2 = 8.8 Hz, 1H), 7.02 (dd, J_1 = 7.2 Hz, J_2 = 8.0 Hz, 1H), 7.09 (d, J = 7.2 Hz, 1H), 7.21 (dd, J_1 = 1.2 Hz, J_2 = 9.2 Hz, 1H), 7.29 (d, J = 8.0 Hz, 2H), 7.64 (d, J = 8.4 Hz, 2H). ^{13}C NMR (100 MHz, $CDCl_3$, TMS) δ 18.3, 21.5, 28.4, 49.4, 54.5, 56.8, 57.6, 71.9, 109.2, 119.8, 120.6, 124.38, 124.44, 124.56, 126.6, 127.2, 127.8, 129.5, 129.7, 130.7, 133.0, 136.4, 140.8, 143.7, 149.0, 151.3. IR (CH_2Cl_2): ν 3052, 2915, 2849, 2359, 1974, 1605, 1486, 1457, 1400, 1341, 1305, 1247, 1155, 1090, 1015, 912, 864,

813, 747, 708 cm^{-1}. HRMS (ESI) calcd. for $C_{28}H_{31}N_2O_3S_2$ $(M+H)^+$: 507.1771, Found: 507.1763. Enantiomeric excess was determined by HPLC with a Chiralcel AD-H column [λ = 254 nm; eluent: Hexane/Isopropanol = 70/30; Flow rate: 0.50 mL/min; t_{minor} = 20.39 min, t_{major} = 18.50 min; ee% = 98%; $[\alpha]^{20}_{D}$ = -55.3 (c 1.00, CH_2Cl_2)].

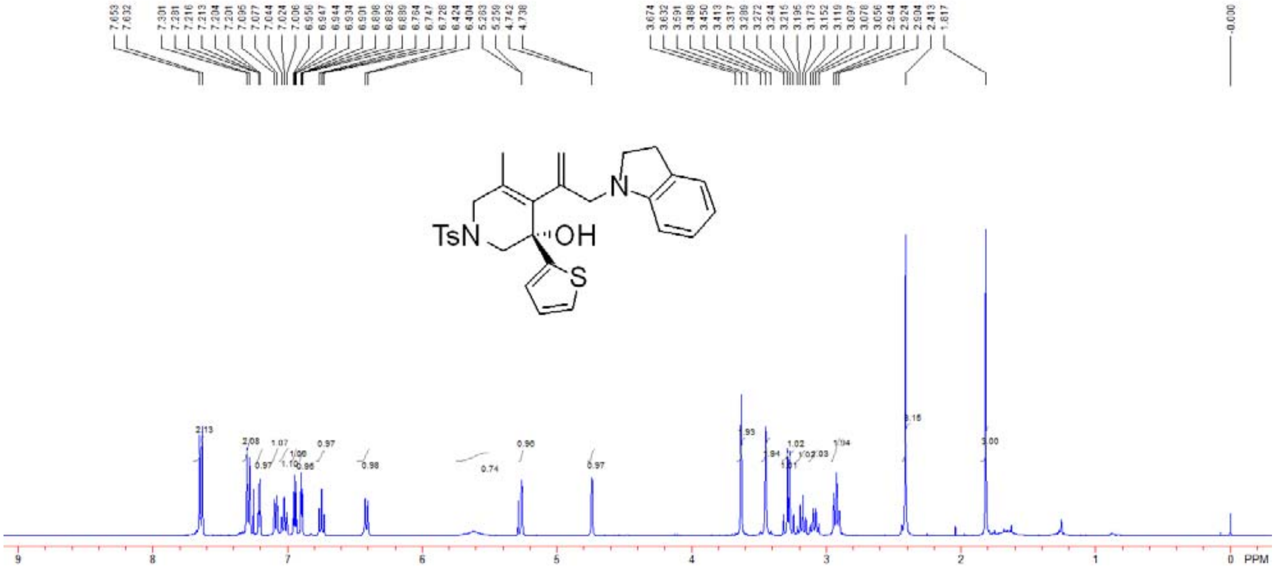

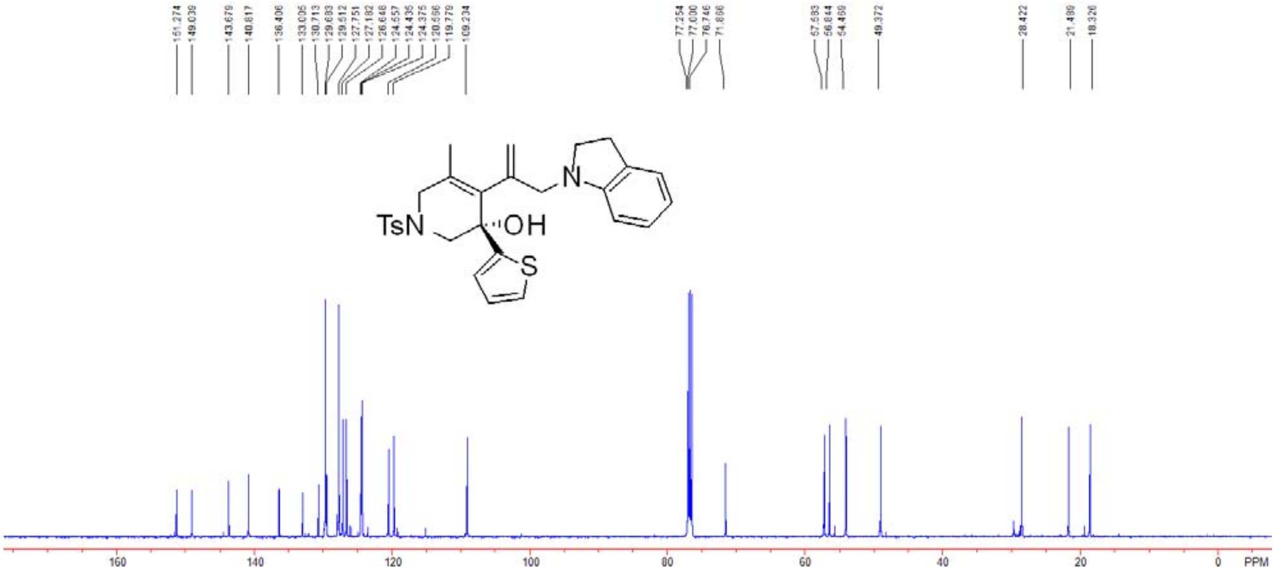

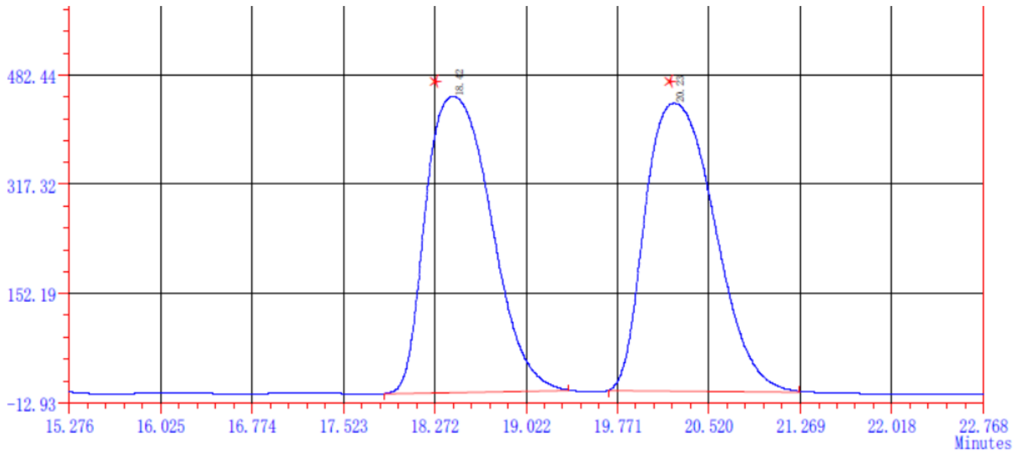

ID	组分名	保留时间	峰高	峰面积	浓度	拖尾因子	理论塔板
2	组份 2	18.423	448267	16760897.8	49.1327	1.37	4839
4	组份 4	20.232	436014	17352653.5	50.8673	1.33	5151
	Σ:		884281	34113551.4	100.0000		

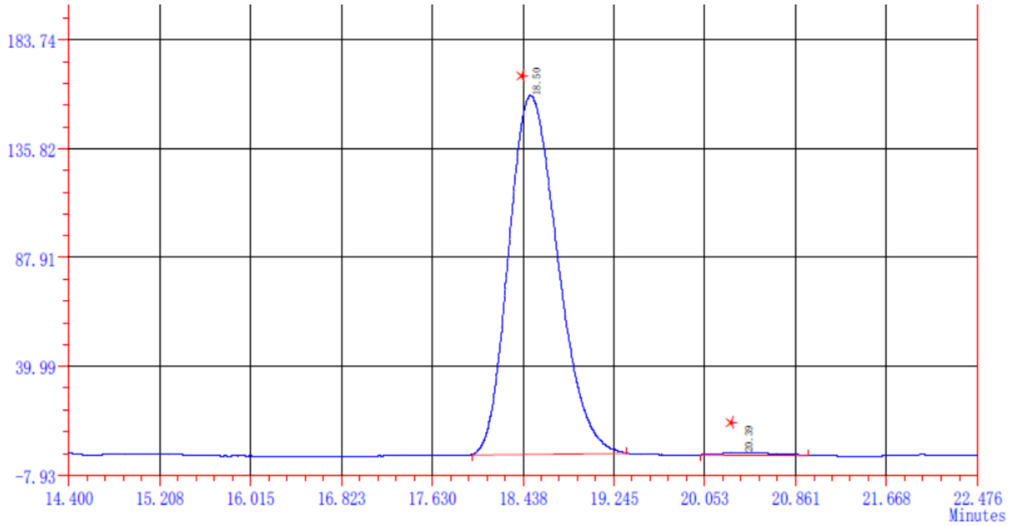

ID	组分名	保留时间	峰高	峰面积	浓度	拖尾因子	理论塔板
3	组份 3	18.502	157903	4779296.9	99.1434	1.24	7447
4	组份 4	20.388	1282	41294.0	0.8566	1.21	7985
	Σ:		159185	4820590.9	100.0000		

Translation: Chiralcel AD-H column [λ = 254 nm; eluent: Hexane/Isopropanol = 70/30; Flow rate: 0.5 mL/min; t_{minor} = 20.39 min, t_{major} = 18.50 min; ee% = 98%].

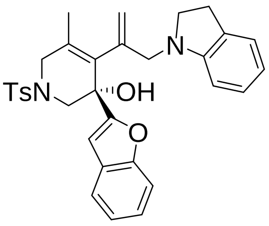

(S)-3-(benzofuran-2-yl)-4-(3-(indolin-1-yl)prop-1-en-2-yl)-5-methyl-1-tosyl-1,2,3,6-tetrahydro pyridin-3-ol (3ja)

A white solid. 83% yield (45 mg). M. P. 124-126 ^{o}C. ^{1}H NMR (400 MHz, $CDCl_3$, TMS) δ 1.83 (s, 3H), 2.39 (s, 3H), 2.91-2.95 (m, 2H), 3.08 (dd, J_1 = 8.4 Hz, J_2 = 16.4 Hz, 1H), 3.16-3.24 (m, 2H), 3.41 (d, J = 16.4 Hz, 1H), 3.48 (d, J = 15.6 Hz, 1H), 3.52 (d, J = 15.6 Hz, 1H), 3.69 (d, J = 11.6 Hz, 1H), 3.96 (d, J = 16.4 Hz, 1H), 4.76 (s, 1H), 5.11 (s, 1H), 5.46 (brs, 1H), 6.43 (d, J = 8.0 Hz, 1H), 6.75 (dd, J_1 = 7.2 Hz, J_2 = 7.6 Hz, 1H), 6.82 (s, 1H), 7.00 (dd, J_1 = 7.6 Hz, J_2 = 7.6 Hz, 1H), 7.09 (d, J = 7.2 Hz, 1H), 7.20-7.23 (m, 2H), 7.26 (d, J = 8.0 Hz, 2H), 7.34-7.36 (m, 1H), 7.53-7.55 (m, 1H), 7.65 (d, J = 8.4 Hz, 2H). ^{13}C NMR (100 MHz, $CDCl_3$, TMS) δ 18.1, 21.5, 28.4, 49.1, 53.8, 54.8, 58.0, 70.8, 104.6, 109.4, 111.0, 119.4, 121.1, 122.8, 123.8, 124.5, 127.1, 127.7, 128.1, 129.7, 130.7, 131.0, 133.1, 134.3, 141.3, 143.7, 151.5, 154.7, 159.2. IR (CH_2Cl_2): ν 3470, 2998, 2963, 2912, 2850, 2360, 2342, 1597, 1521, 1490, 1454, 1380, 1346, 1246, 1170, 1155, 1090, 1065, 1038, 994, 964, 910, 857, 812, 763, 681, 660 cm^{-1}. HRMS (ESI) calcd. for $C_{32}H_{33}N_2O_4S$ $(M+H)^+$: 541.2156, Found: 541.2162. Enantiomeric excess was determined by HPLC with a Chiralcel AD-H column [λ = 254 nm; eluent: Hexane/Isopropanol = 80/20; Flow rate: 0.50 mL/min; t_{minor} = 24.15 min, t_{major} = 20.13 min; ee% > 99%; $[\alpha]^{20}_{D}$ = +32.3 (c 1.00, CH_2Cl_2)].

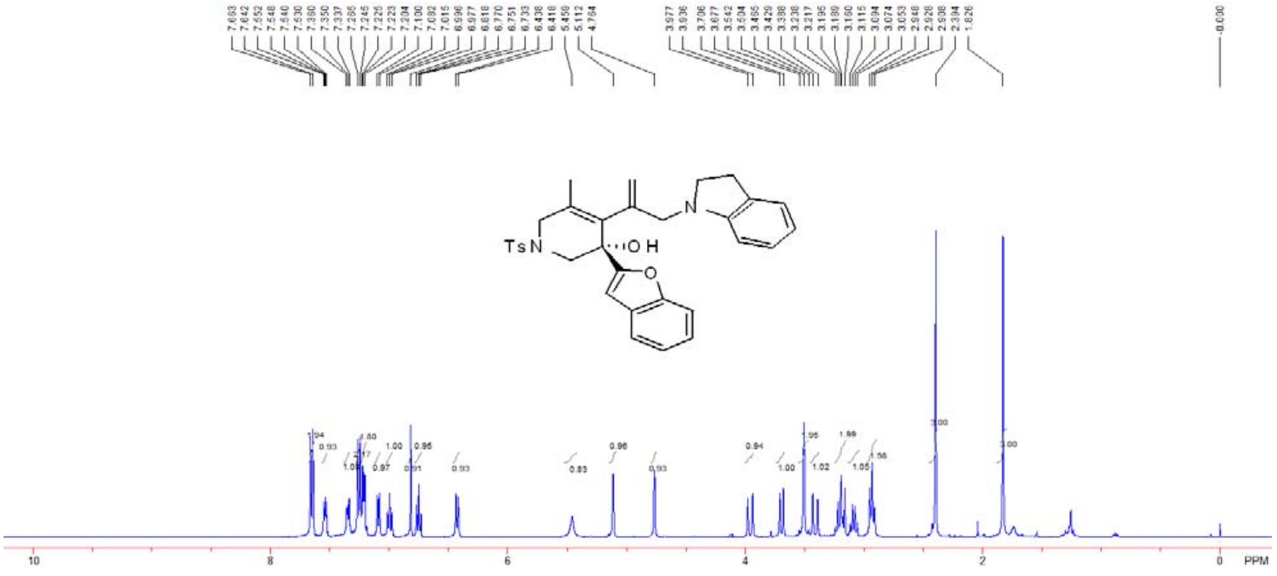

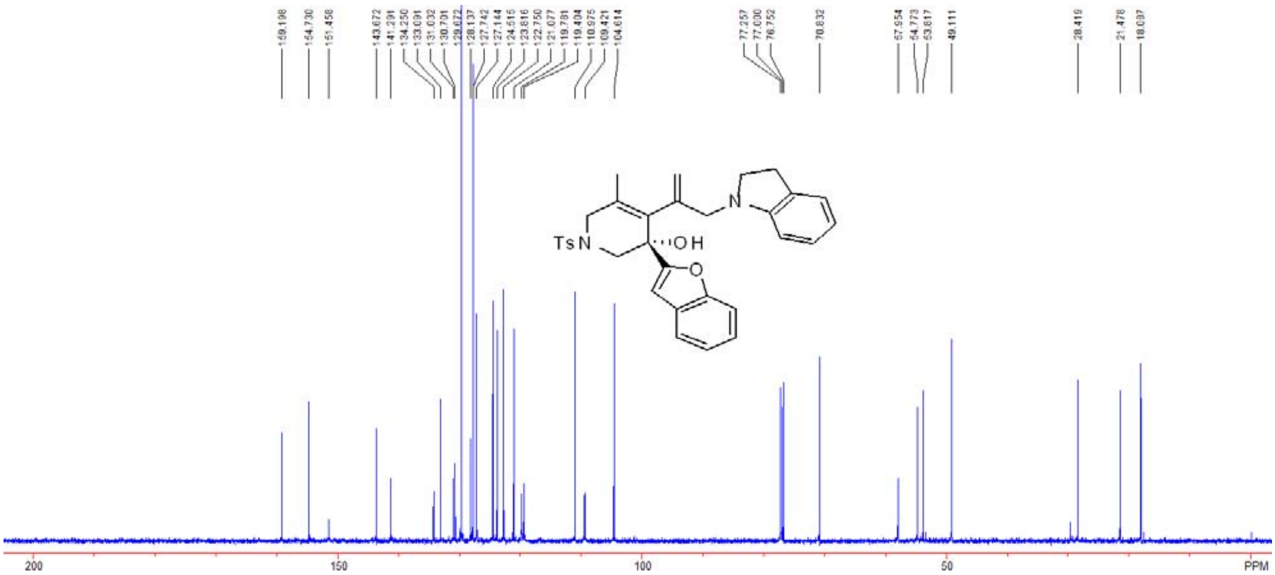

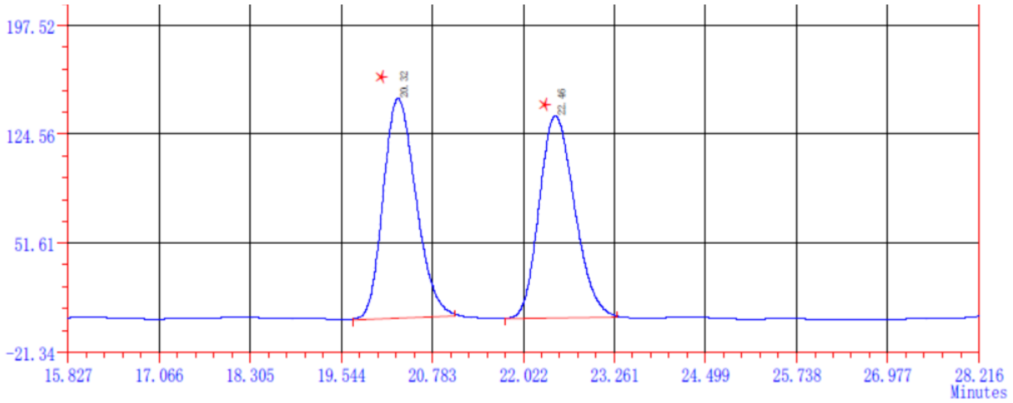

ID	组分名	保留时间	峰高	峰面积	浓度	拖尾因子	理论塔板
4	组份 4	20.318	147306	4570564.4	50.1367	1.24	8547
2		22.463	135560	4545633.3	49.8633	1.18	8944
	Σ:		282866	9116197.7	100.0000		

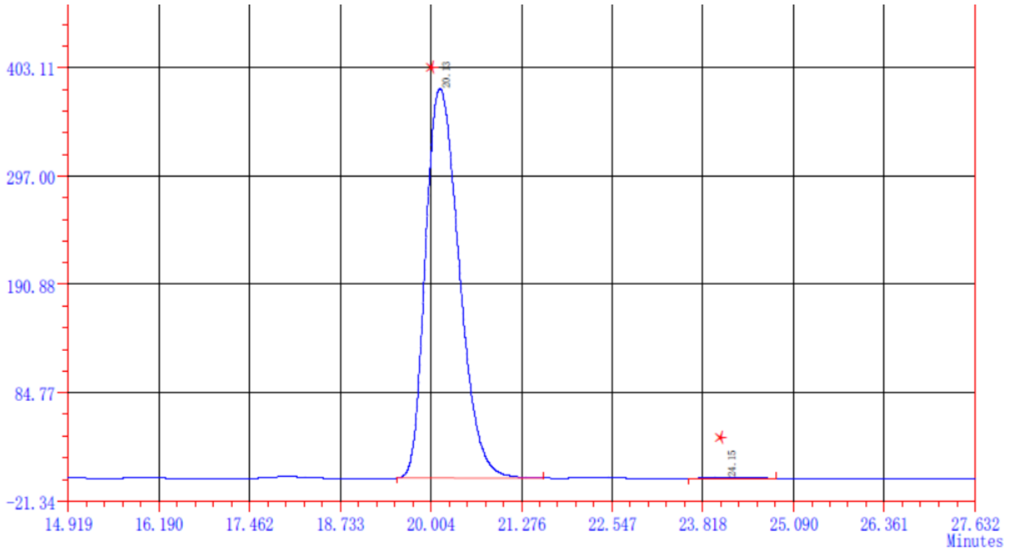

ID	组分名	保留时间	峰高	峰面积	浓度	拖尾因子	理论塔板
4	组份 4	20.132	382021	12518015.3	99.6737	1.30	7523
2		24.145	913	40979.3	0.3263	1.19	5768
	Σ:		382934	12558994.7	100.0000		

Translation: Chiralcel AD-H column [λ = 254 nm; eluent: Hexane/Isopropanol = 80/20; Flow rate: 0.5 mL/min; t_{minor} = 24.15 min, t_{major} = 20.13 min; ee% >99%].

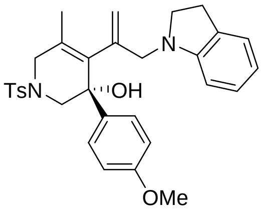

(S)-4-(3-(indolin-1-yl)prop-1-en-2-yl)-3-(4-methoxyphenyl)-5-methyl-1-tosyl-1,2,3,6-tetrahydropyridin-3-ol (3ka)

A light yellow oil, 87% yield (46 mg). ^{1}H NMR ($CDCl_3$, 400 MHz, TMS) δ 1.86 (s, 3H), 2.41 (s, 3H), 2.83 (d, J = 11.6 Hz, 1H), 2.88-2.92 (m, 2H), 3.01 (dd, J_1 = 8.8 Hz, J_2 = 16.4 Hz, 1H), 3.12 (dd, J_1 = 8.8 Hz, J_2 = 16.4 Hz, 1H), 3.26 (d, J = 14.8 Hz, 1H), 3.43-3.48 (m, 3H), 3.81 (s, 3H), 3.86 (d, J = 16.4 Hz, 1H), 4.72 (s, 1H), 4.77 (brs, 1H), 5.20 (d, J = 1.6 Hz, 1H), 6.26 (d, J = 7.6 Hz, 1H), 6.71 (ddd, J_1 = 0.4 Hz, J_2 = 7.2 Hz, J_3 = 7.6 Hz, 1H), 6.84 (d, J = 8.8 Hz, 2H), 6.99 (dd, J_1 = 7.2 Hz, J_2 = 8.0 Hz, 1H), 7.06 (d, J = 6.8 Hz, 1H), 7.28 (d, J = 8.0 Hz, 2H), 7.36 (d, J = 8.8 Hz, 2H), 7.63 (d, J = 8.0 Hz, 2H). ^{13}C NMR (100 MHz, $CDCl_3$, TMS) δ 18.6, 21.5, 28.4, 49.6, 54.1, 55.2, 57.0, 57.4, 72.3, 108.9, 113.1, 119.3, 119.7, 124.4, 127.1, 127.7, 127.8, 129.7, 130.2, 130.5, 132.9, 135.2, 136.3, 141.5, 143.6, 151.5, 158.7. IR (CH_2Cl_2) ν 3521, 3051, 2924, 2852, 2359, 2340, 1605, 1510, 1476, 1457, 1346, 1305, 1245, 1173, 1156, 1103, 1090, 1040, 987, 937, 898,

868, 833, 816, 777, 743, 709 cm^{-1}. HRMS (ESI) calcd. for $C_{31}H_{35}N_2O_4S$ $(M+H)^+$: 531.2312, Found: 531.2303. Enantiomeric excess was determined by HPLC with a Chiralcel AD-H column [λ = 254 nm; eluent: Hexane/Isopropanol = 80/20; Flow rate: 0.50 mL/min; t_{minor} = 17.75 min, t_{major} = 27.81 min; ee% > 99%; $[\alpha]^{20}_D$ = +39.3 (c 1.00, CH_2Cl_2)].

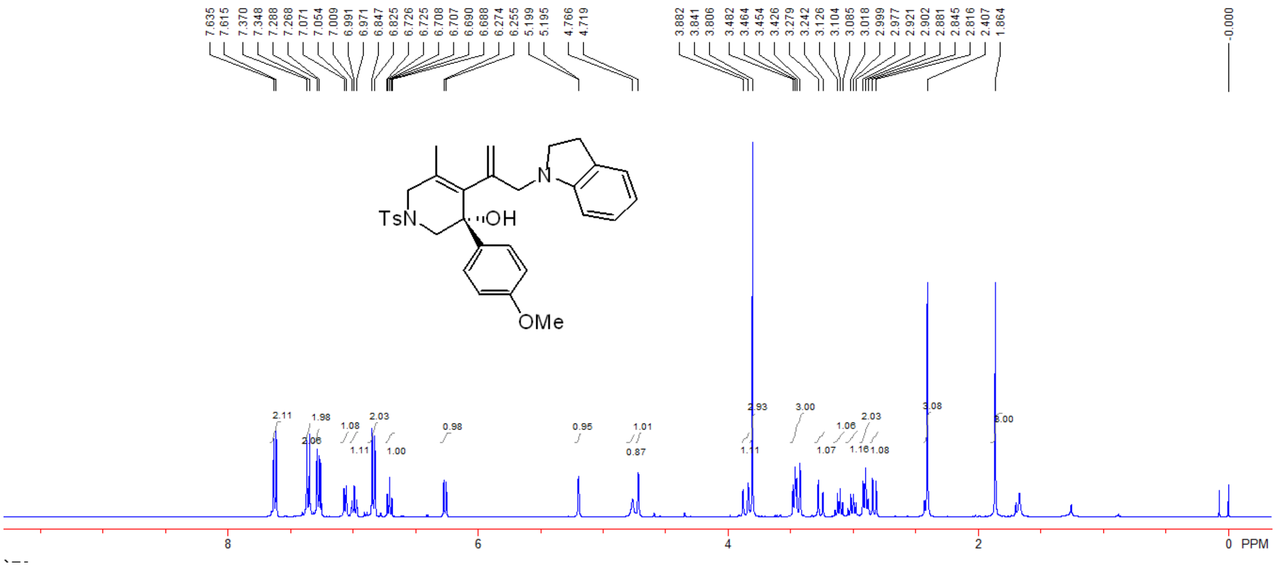

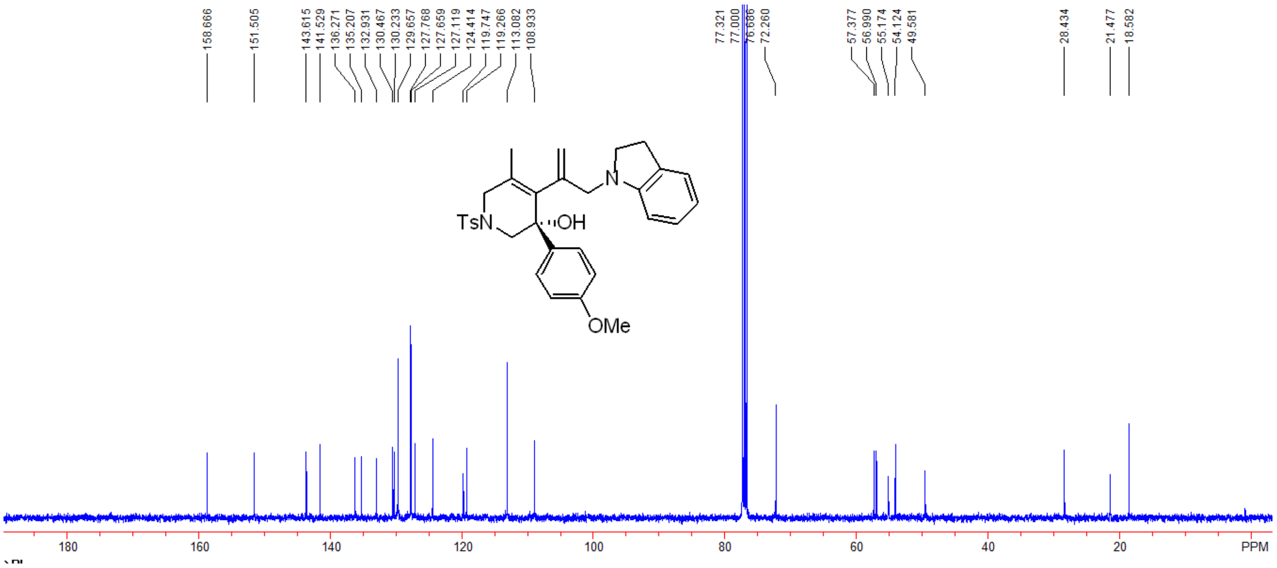

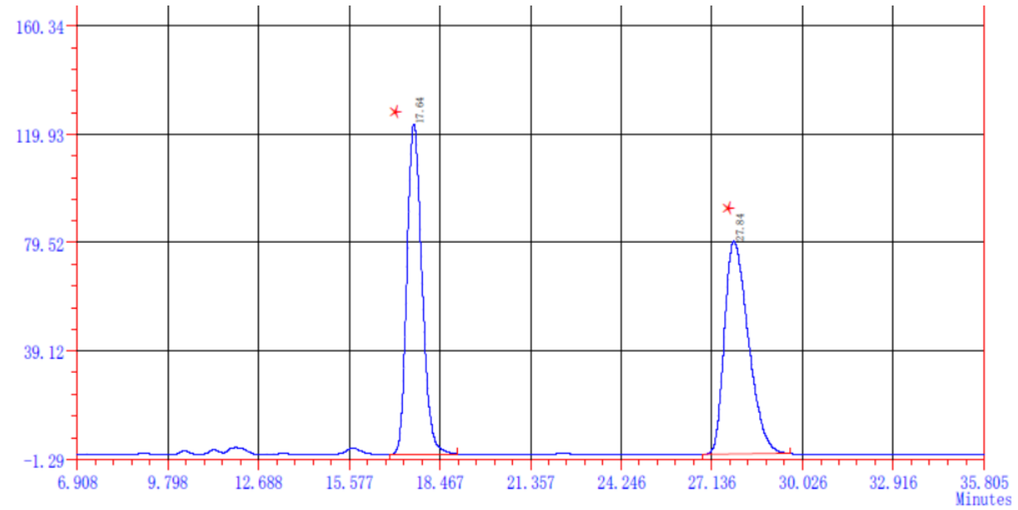

ID	组分名	保留时间	峰高	峰面积	浓度	拖尾因子	理论塔板
2	组份 2	17.637	123117	4089054.8	49.0985	1.21	5620
2		27.842	79398	4239209.1	50.9015	1.41	5420
	Σ:		202515	8328263.9	100.0000		

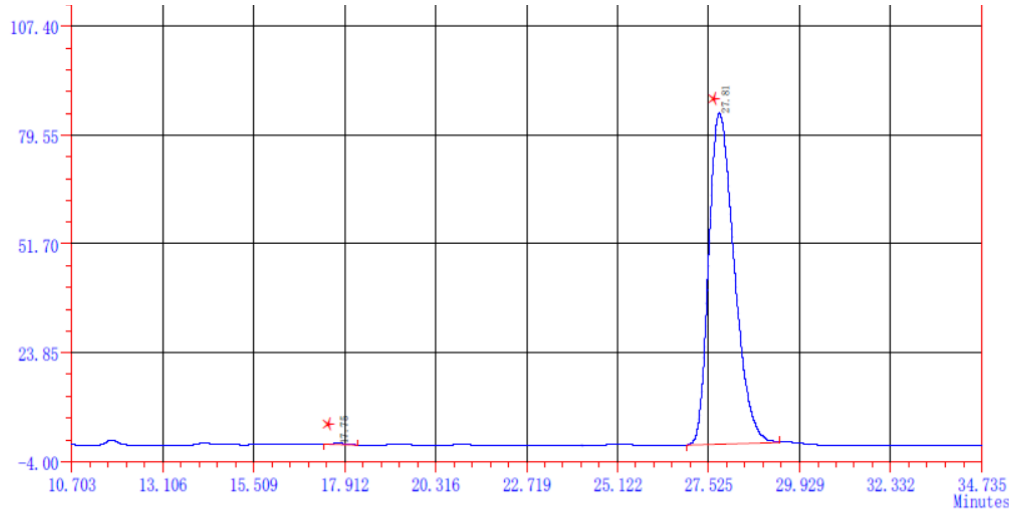

ID	组分名	保留时间	峰高	峰面积	浓度	拖尾因子	理论塔板
2	组份 2	17.750	258	7115.1	0.1785	0.92	8256
2		27.808	84817	3979988.8	99.8215	1.36	7000
	Σ:		85075	3987103.9	100.0000		

Translation: Chiralcel AD-H column [λ = 254 nm; eluent: Hexane/Isopropanol = 80/20; Flow rate: 0.5 mL/min; t_{minor} = 17.75 min, t_{major} = 27.81 min; ee% >99%].

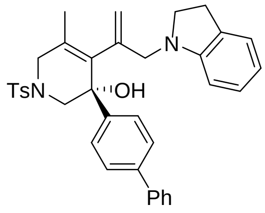

(S)-3-([1,1'-biphenyl]-4-yl)-4-(3-(indolin-1-yl)prop-1-en-2-yl)-5-methyl-1-tosyl-1,2,3,6-tetrahydropyridin-3-ol (3la)

A light yellow oil. 92% yield (53 mg). ^{1}H NMR (500 MHz, $CDCl_3$, TMS) δ 1.89 (s, 3H), 2.40 (s, 3H), 2.89-2.92 (m, 3H), 3.02 (dd, J_1 = 8.5 Hz, J_1 = 17.0 Hz, 1H), 3.08-3.15 (m, 1H), 3.29 (d, J = 15.0 Hz, 1H), 3.46-3.55 (m, 3H), 3.90 (d, J = 16.0 Hz, 1H), 4.77 (s, 1H), 4.89 (s, 1H), 5.22 (s, 1H), 6.24 (d, J = 7.5 Hz, 1H), 6.70 (dd, J_1 = 7.0 Hz, J_1 = 7.5 Hz, 1H), 6.96 (dd, J_1 = 7.5 Hz, J_1 = 7.5 Hz, 1H), 7.06 (d, J = 7.0 Hz, 1H), 7.28 (d, J = 8.0 Hz, 2H), 7.33 (dd, J_1 = 7.0 Hz, J_1 = 7.5 Hz, 1H), 7.44 (dd, J_1 = 7.5 Hz, J_1 = 7.5 Hz, 2H), 7.50-7.55 (m, 4H), 7.60-7.65 (m, 4H). ^{13}C NMR (125 MHz, $CDCl_3$, TMS) δ 18.6, 21.5, 28.5, 49.6, 54.2, 57.1, 57.3, 72.5, 108.9, 119.3, 120.0, 124.4, 126.4, 127.01, 127.17, 127.25, 127.79, 128.7, 129.7, 130.47, 130.50, 133.0, 136.1, 139.9, 140.7, 141.4, 142.2, 143.7, 151.5. IR (CH_2Cl_2): ν 2969, 2919, 2850, 2360, 2342, 1653, 1604, 1518, 1486, 1456, 1343, 1305, 1249, 1158, 1090, 988, 911, 873, 848, 810, 749, 705 cm^{-1}. HRMS (ESI) calcd. for $C_{36}H_{37}N_2O_3S$ $(M+H)^+$: 577.2510, Found: 577.2519. Enantiomeric excess was determined by HPLC with a Chiralcel AD-H column [λ = 211 nm; eluent: Hexane/Isopropanol = 70/30; Flow rate: 0.50 mL/min; t_{minor} = 20.49 min, t_{major} = 29.37 min; ee% > 99%; $[\alpha]^{20}_D$ = +50.1 (c 1.00, CH_2Cl_2)].

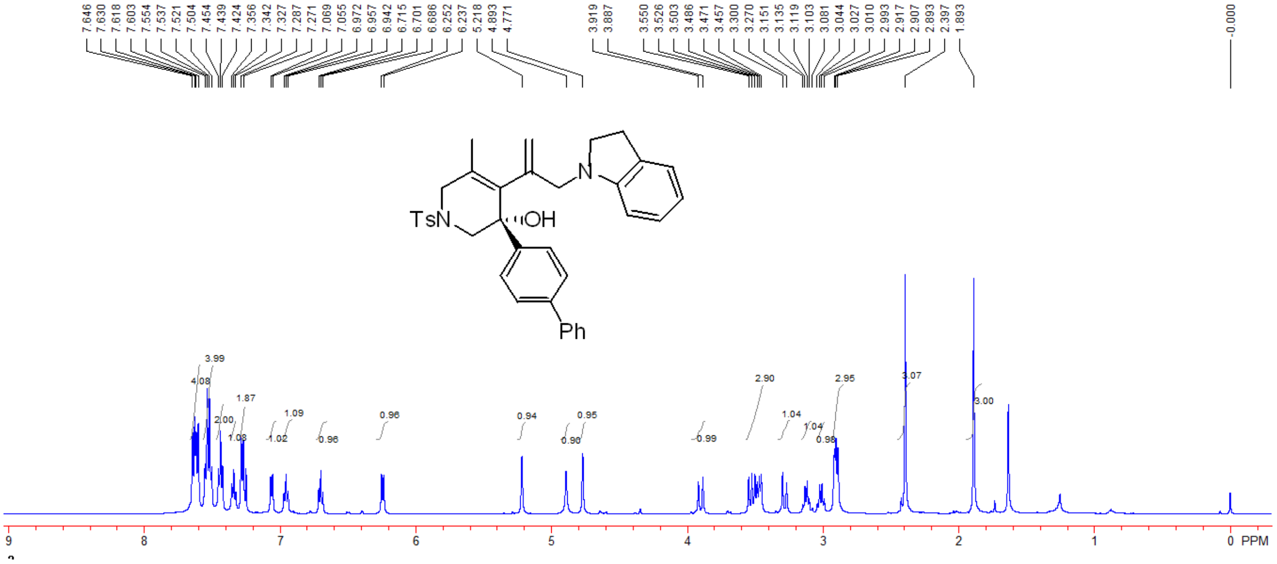

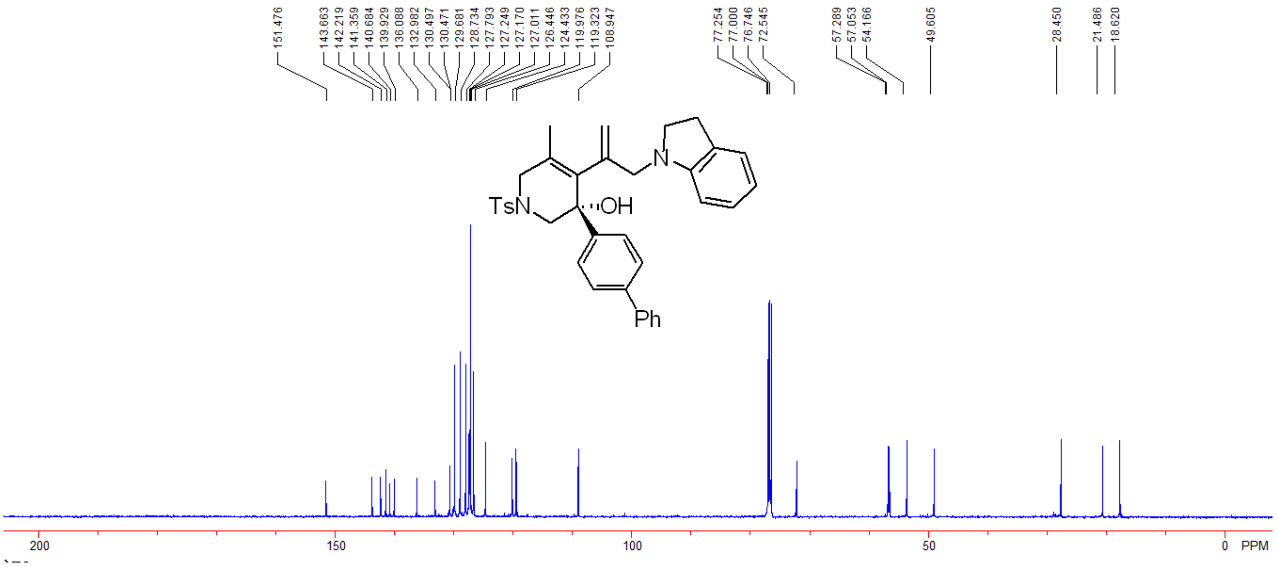

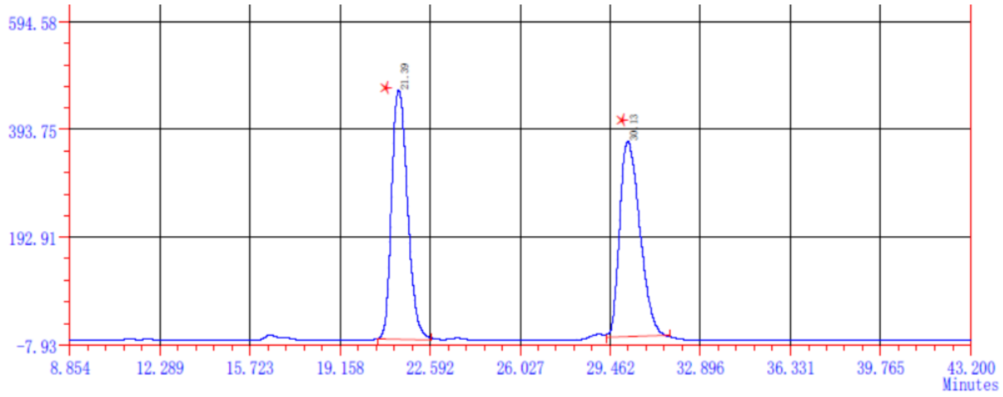

ID	组分名	保留时间	峰高	峰面积	浓度	拖尾因子	理论塔板
1		21.393	464869	19566812.4	50.0135	1.25	5149
2		30.132	365190	19556211.5	49.9865	1.38	6310
	Σ:		830059	39123023.9	100.0000		

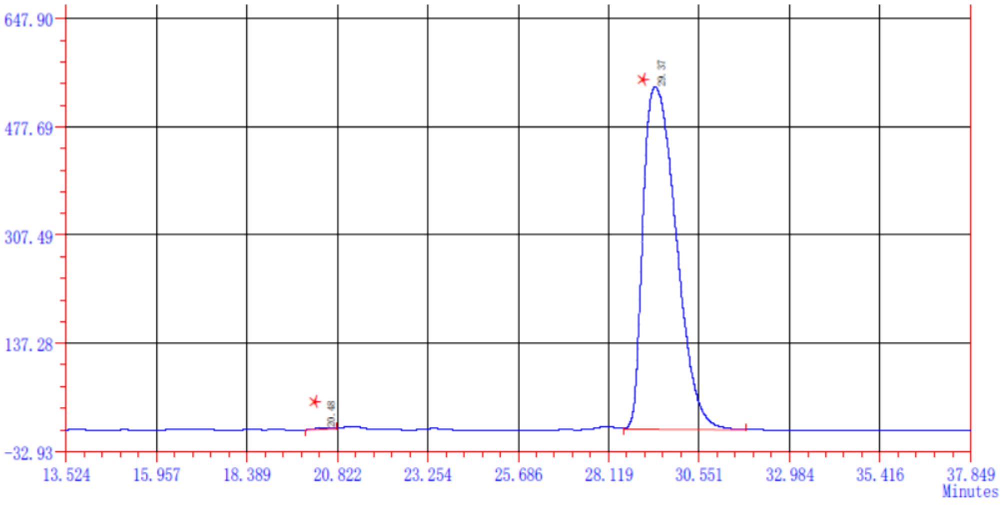

ID	组分名	保留时间	峰高	峰面积	浓度	拖尾因子	理论塔板
4	组份 4	20.485	2542	75697.2	0.2256	1.01	9432
2		29.372	539089	33473759.9	99.7744	1.56	4460
	Σ:		541631	33549457.1	100.0000		

Translation: Chiralcel AD-H column [λ = 211 nm; eluent: Hexane/Isopropanol = 70/30; Flow rate: 0.5 mL/min; t_{minor} = 20.49 min, t_{major} = 29.37 min; ee% >99%].

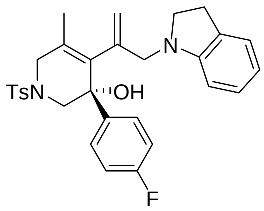

(S)-3-(4-fluorophenyl)-4-(3-(indolin-1-yl)prop-1-en-2-yl)-5-methyl-1-tosyl-1,2,3,6-tetrahydro pyridin-3-ol (3ma)

A white solid. 78% yield (40 mg). M. P. 89-91 °C. ^{1}H NMR (400 MHz, $CDCl_3$, TMS) δ 1.87 (s, 3H), 2.41 (s, 3H), 2.86-2.92 (m, 3H), 3.01 (dd, J_1 = 8.0 Hz, J_2 = 16.4 Hz 1H), 3.10 (dd, J_1 = 8.0 Hz, J_2 = 16.4 Hz 1H), 3.29 (d, J = 14.8 Hz, 1H), 3.40 (d, J = 11.6 Hz, 1H), 3.42 (d, J = 14.8 Hz, 1H), 3.51 (d, J = 16.4 Hz, 1H), 3.82 (d, J = 16.4 Hz, 1H), 4.71 (s, 1H), 5.11 (brs, 1H), 5.21 (s, 1H), 6.27-6.29 (m, 1H), 6.72-6.75 (m, 1H), 6.96-7.03 (m, 2H), 7.08 (d, J = 7.2 Hz, 1H), 7.25-7.29 (m, 3H), 7.39-7.45 (m, 2H), 7.60-7.63 (m, 2H). ^{13}C NMR (100 MHz, $CDCl_3$, TMS) δ 18.5, 21.5, 28.4, 49.5, 54.3, 57.1, 57.3, 72.3 (d, J = 2.9 Hz), 109.1, 114.5 (d, J = 21.2 Hz), 119.7, 120.5, 124.5, 127.2, 127.7, 127.9 (d, J = 5.1 Hz), 129.7, 130.6, 130.8, 132.9 (d, J = 2.4 Hz), 135.6, 141.2, 142.0, 143.7, 151.3, 162.0 (d, J = 244.2). ^{19}F NMR (376 MHz, $CDCl_3$) δ -115.8 (s, 1F). IR (CH_2Cl_2): ν 3072, 2976, 2918, 2847, 2359, 1604, 1519, 1486, 1450, 1400, 1344, 1305, 1220, 1155, 1090, 1014, 987, 909, 864, 812, 750, 707 cm^{-1}. HRMS (ESI) calcd. for $C_{30}H_{32}FN_2O_3S$ $(M+H)^+$: 519.2112, Found: 519.2105. Enantiomeric excess was determined by HPLC with a Chiralcel IC-H column [λ = 254 nm; eluent: Hexane/Isopropanol = 70/30; Flow rate: 0.50 mL/min; t_{minor} = 21.12 min, t_{major} = 27.12 min; ee% > 99%; $[\alpha]^{20}_D$ = +30.5 (c 1.00, CH_2Cl_2)].

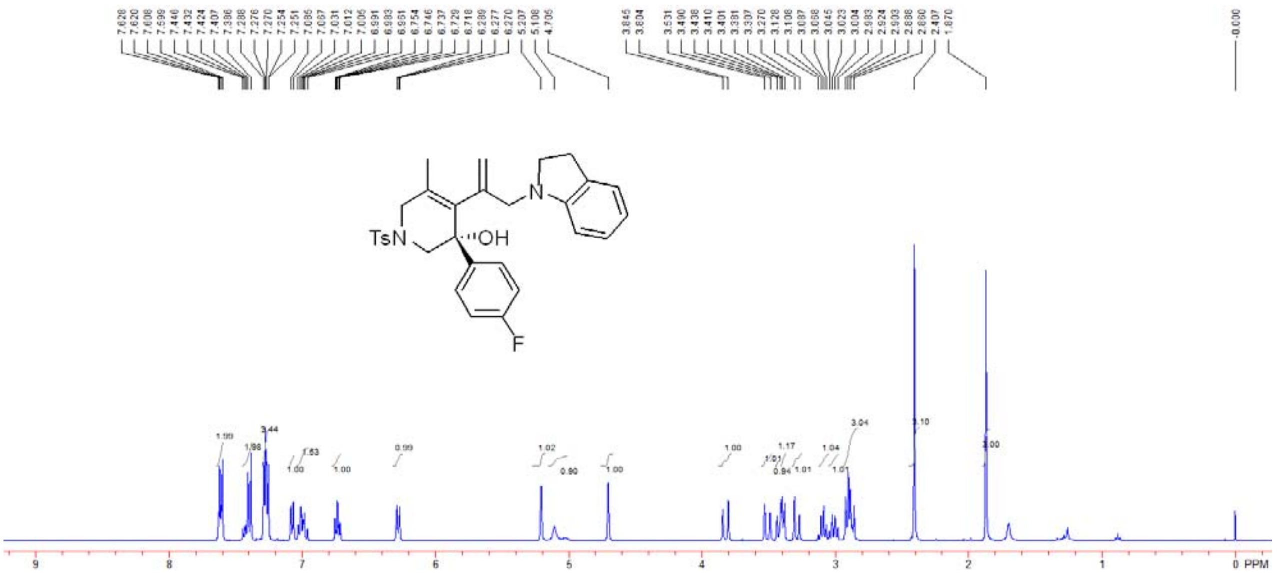
7.628 7.620 7.608 7.599 7.440 7.432 7.424 7.407 7.398 7.288 7.276 7.270 7.254 7.251 7.085 7.067 7.031 7.012 7.006 6.991 6.983 6.661 6.754 6.746 6.737 6.729 6.718 6.289 6.277 6.270 5.207 5.108 4.705
3.845 3.804 3.531 3.490 3.438 3.410 3.401 3.381 3.337 3.270 3.128 3.108 3.087 3.068 3.045 3.023 3.004 2.983 2.924 2.903 2.888 2.860 2.407 1.870
-0.000
TsN
OH
N
F
1.99 1.98 3.44 1.53 1.00 1.00 0.99 1.02 0.90 1.00 1.00 1.01 1.17 0.94 1.01 1.04 1.01 3.04 3.10 3.00
9 8 7 6 5 4 3 2 1 0 PPM

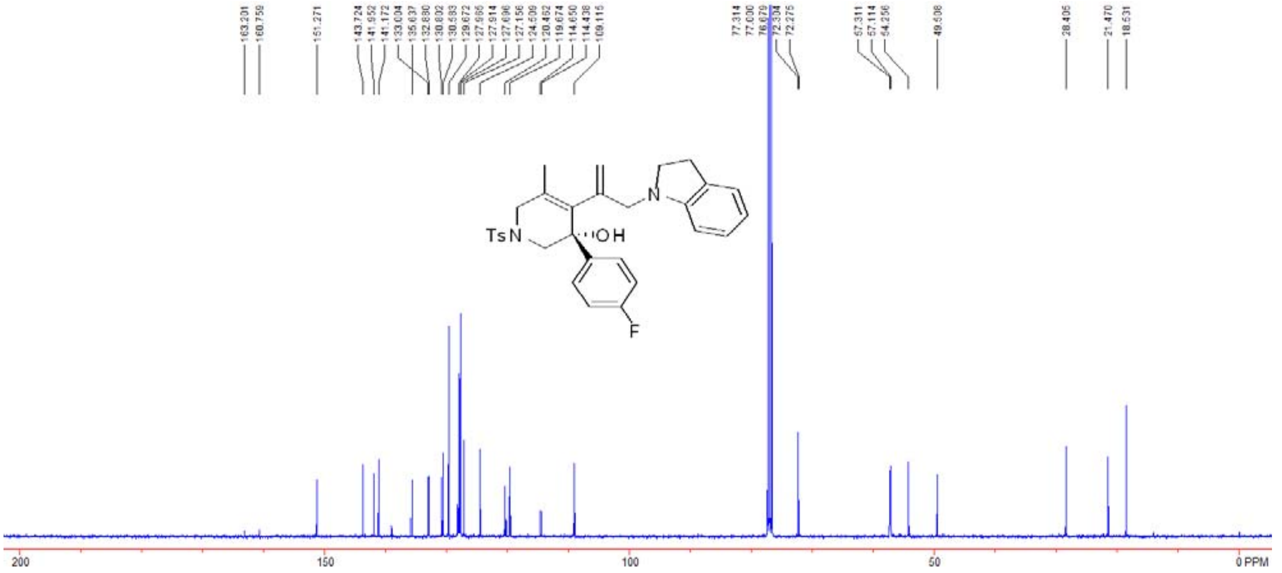
163.201 160.756 151.271 143.724 141.952 141.172 133.004 135.637 132.880 130.802 130.593 129.672 127.966 127.914 127.696 127.156 124.509 120.462 119.674 114.650 114.436 109.115
77.314 77.060 76.679 72.304 72.275 57.311 57.114 54.256 49.506 28.405 21.470 18.531
TsN
OH
N
F
200 150 100 50 0 PPM

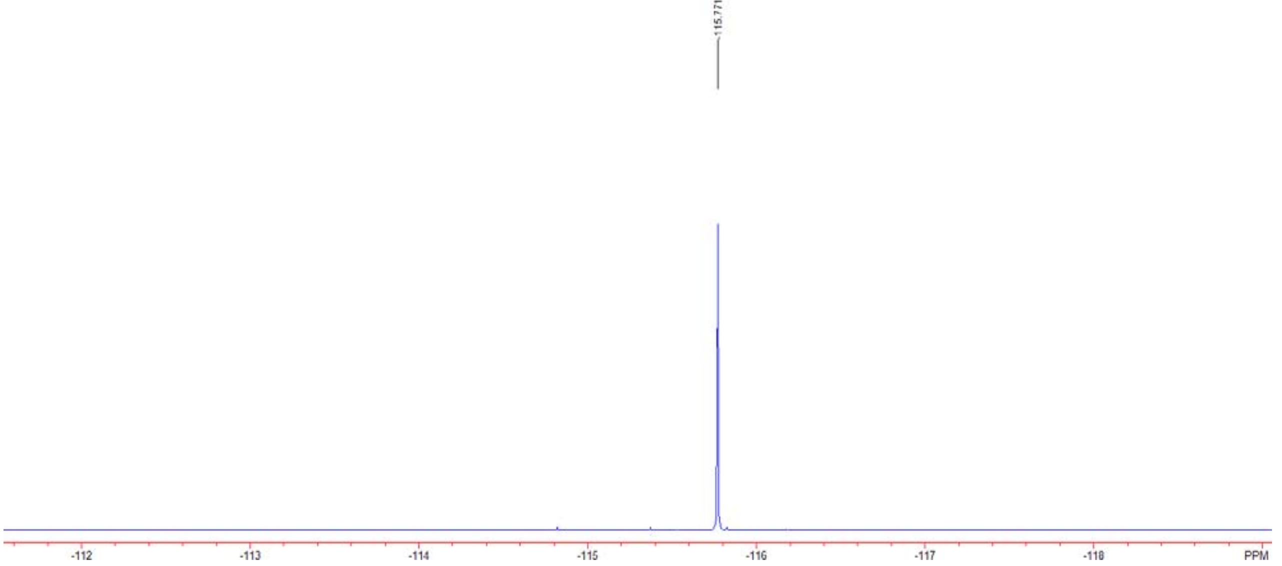
-115.771
-112 -113 -114 -115 -116 -117 -118 PPM

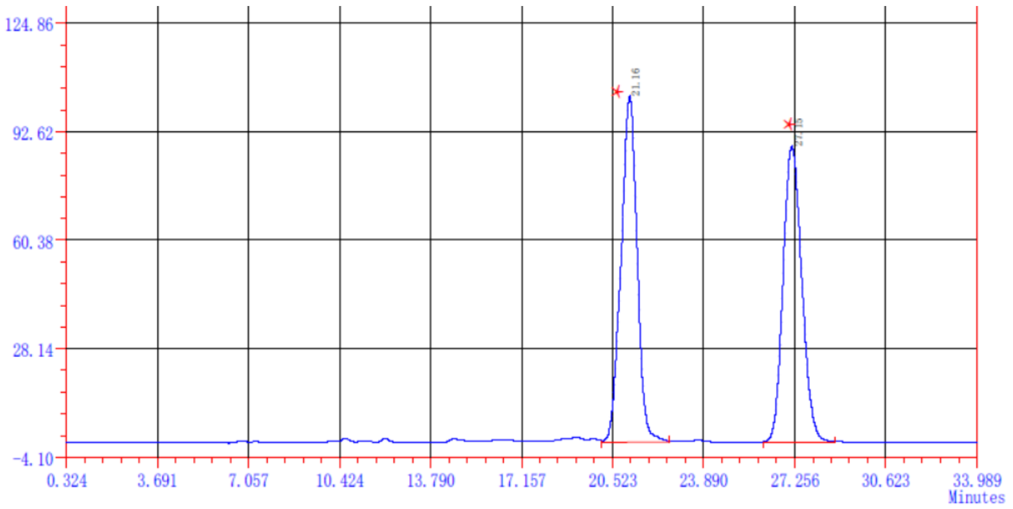

ID	组分名	保留时间	峰高	峰面积	浓度	拖尾因子	理论塔板
4	组份 4	21.155	102905	4274312.6	50.1284	0.96	5170
2		27.152	88162	4252419.0	49.8716	1.13	6316
	Σ:		191067	8526731.6	100.0000		

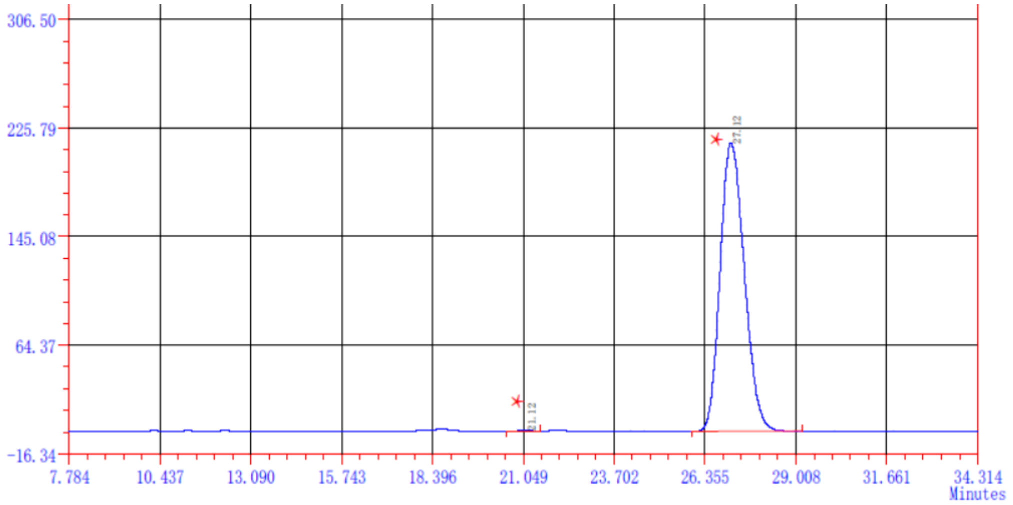

ID	组分名	保留时间	峰高	峰面积	浓度	拖尾因子	理论塔板
4	组份 4	21.115	423	15099.1	0.1424	0.88	6974
2		27.115	214380	10585221.6	99.8576	1.18	6011
	Σ:		214803	10600320.7	100.0000		

Translation: Chiralcel IC-H column [λ = 254 nm; eluent: Hexane/Isopropanol = 70/30; Flow rate: 0.5 mL/min; t_{minor} = 21.12 min, t_{major} = 27.12 min; ee% > 99%].

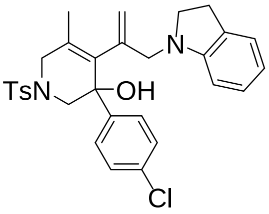

3-(4-chlorophenyl)-4-(3-(indolin-1-yl)prop-1-en-2-yl)-5-methyl-1-tosyl-1,2,3,6-tetrahydropyridin-3-ol (3na)

A white solid. 94% yield (50 mg). M. P. 103-105 °C. ^{1}H NMR (400 MHz, $CDCl_3$, TMS) δ 1.87 (s, 3H), 2.41 (s, 3H), 2.85-2.93 (m, 3H), 3.01 (dd, J_1 = 8.4 Hz, J_2 = 16.4 Hz, 1H), 3.09 (dd, J_1 = 8.4 Hz, J_2 = 16.4 Hz, 1H), 3.29 (d, J = 14.4 Hz, 1H), 3.38-3.44 (m, 2H), 3.51 (d, J = 16.0 Hz, 1H), 3.83 (d, J = 16.0 Hz, 1H), 4.71 (s, 1H), 5.14 (brs, 1H), 5.22 (s, 1H), 6.29 (d, J = 7.6 Hz, 1H), 6.75 (dd, J_1 = 7.2 Hz, J_2 = 7.6 Hz, 1H), 7.02 (dd, J_1 = 7.6 Hz, J_2 = 8.0 Hz, 1H), 7.08 (d, J = 7.2 Hz, 1H), 7.26-7.29 (m, 4H), 7.40 (d, J = 8.4 Hz, 2H), 7.61 (d, J = 8.4 Hz, 2H). ^{13}C NMR (100 MHz, $CDCl_3$, TMS) δ 18.6, 21.5, 28.4, 49.5, 54.3, 57.2, 57.4, 72.3, 109.2, 119.8, 120.6, 124.6, 127.20, 127.75, 127.96, 128.0, 129.7, 130.7, 130.9, 132.9, 133.1, 135.7, 141.2, 142.0, 143.8, 151.3. IR (CH_2Cl_2): ν 3031, 2969, 2913, 2849, 2360, 2342, 1602, 1518, 1489, 1450, 1343, 1305, 1289, 1249, 1157, 1090, 1044, 1022,988, 911, 873, 857, 811, 750, 701 cm^{-1}. HRMS (ESI) calcd. for $C_{30}H_{32}ClN_2O_3S$ $(M+H)^+$: 535.1817, Found: 535.1807. Enantiomeric excess was determined by HPLC with a Chiralcel AD-H column [λ = 254 nm; eluent: Hexane/Isopropanol = 70/30; Flow rate: 0.50 mL/min; t_{minor} = 16.70 min, t_{major} = 19.51 min; ee% > 99%; $[\alpha]^{20}_D$ = -8.0 (c 1.00, CH_2Cl_2)].

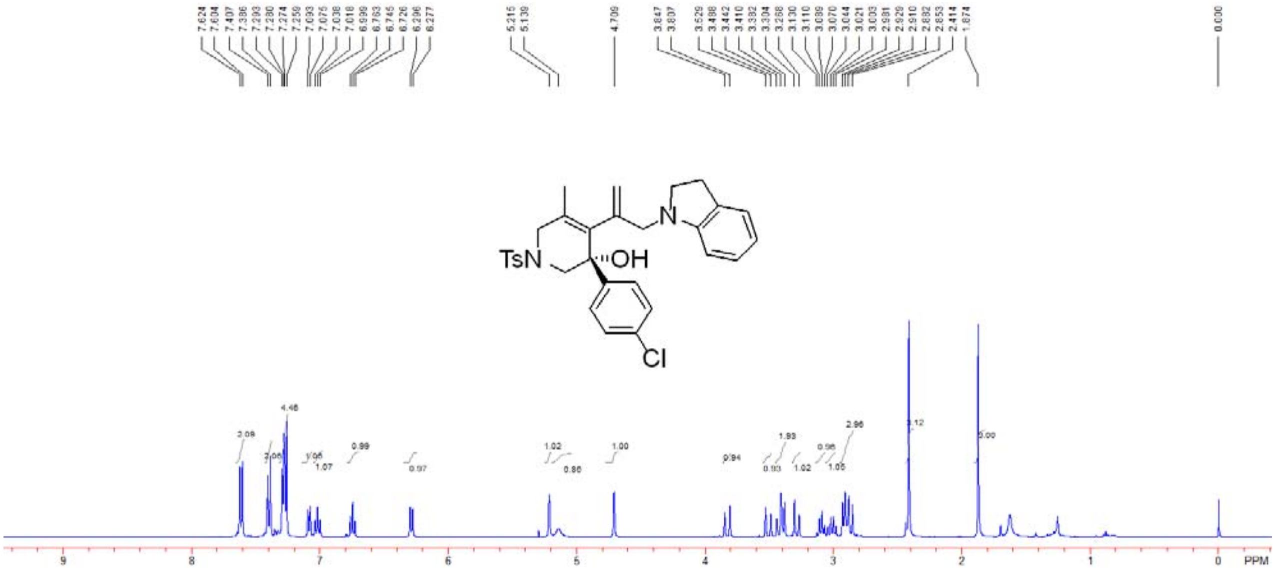

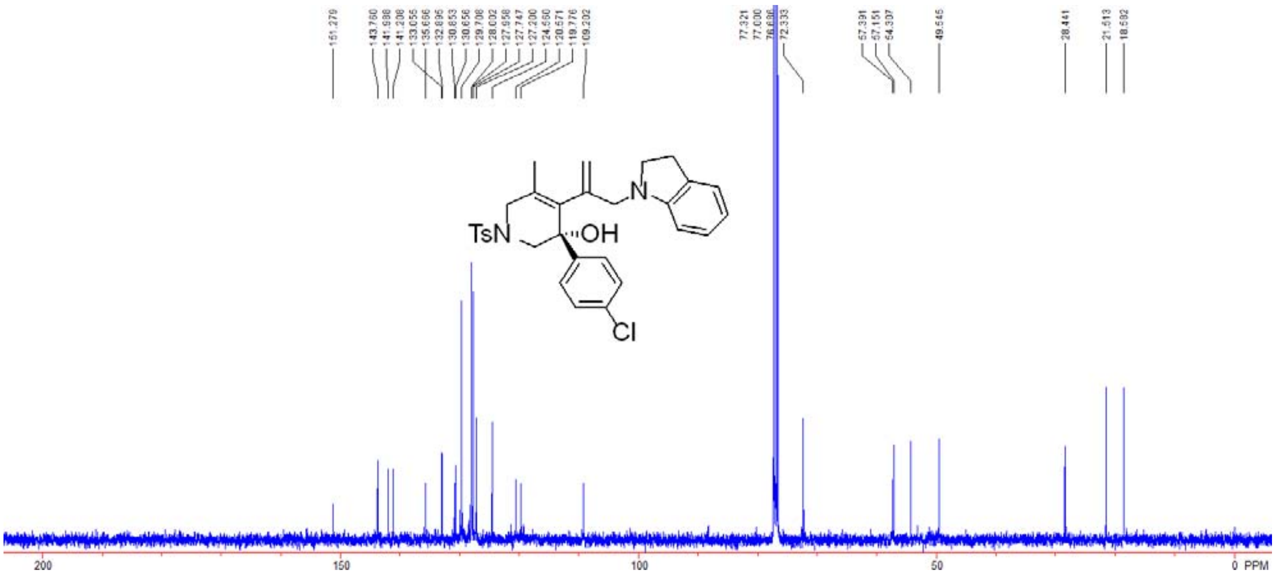

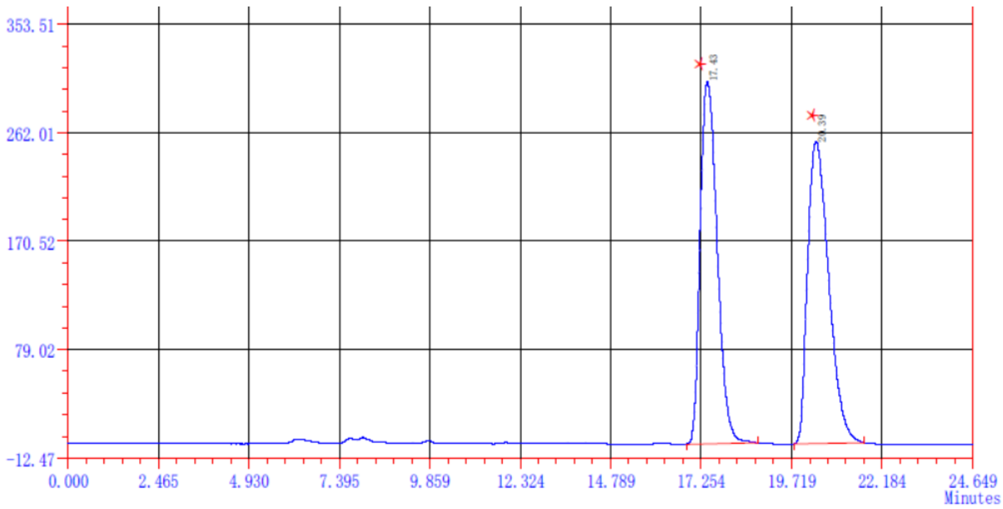

ID	组分名	保留时间	峰高	峰面积	浓度	拖尾因子	理论塔板
2	组份 2	17.425	305932	9842425.6	49.1254	1.32	5847
4	组份 4	20.388	254490	10192873.2	50.8746	1.47	5165
	Σ:		560422	20035298.8	100.0000		

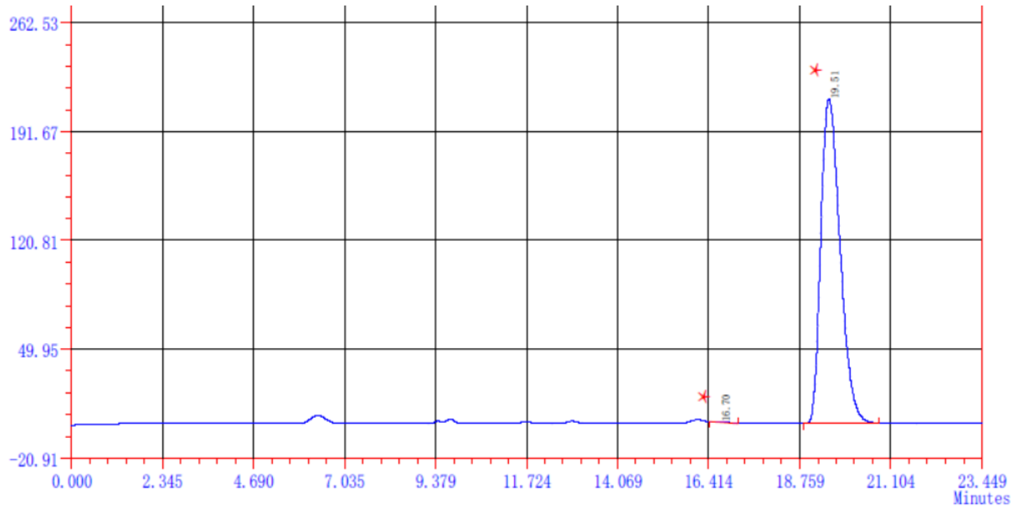

ID	组分名	保留时间	峰高	峰面积	浓度	拖尾因子	理论塔板
1		16.698	655	17082.9	0.2330	0.97	8170
3	组份 3	19.508	211821	7314484.2	99.7670	1.36	6361
	Σ:		212476	7331567.0	100.0000		

Translation: Chiralcel AD-H column [λ = 254 nm; eluent: Hexane/Isopropanol = 70/30; Flow rate: 0.5 mL/min; t_{minor} = 16.70 min, t_{major} = 19.51 min; ee% > 99%].

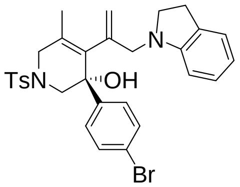

(S)-3-(4-bromophenyl)-4-(3-(indolin-1-yl)prop-1-en-2-yl)-5-methyl-1-tosyl-1,2,3,6-tetrahydro pyridin-3-ol (3oa)

A light yellow oil. 81% yield (47 mg). ^{1}H NMR (400 MHz, $CDCl_3$, TMS) δ 1.87 (s, 3H), 2.41 (s, 3H), 2.85-2.93 (m, 3H), 3.01 (dd, J_1 = 8.0 Hz, J_2 = 16.4 Hz, 1H), 3.10 (dd, J_1 = 8.0 Hz, J_2 = 16.4 Hz, 1H), 3.29 (d, J = 14.4 Hz, 1H), 3.38-3.44 (m, 2H), 3.51 (d, J = 16.0 Hz, 1H), 3.83 (d, J = 16.0 Hz, 1H), 4.71 (s, 1H), 5.13 (brs, 1H), 5.22 (d, J = 0.8 Hz, 1H), 6.28 (d, J = 8.0 Hz, 1H), 6.74 (dd, J_1 = 7.2 Hz, J_2 = 7.6 Hz, 1H), 7.02 (dd, J_1 = 7.2 Hz, J_2 = 7.6 Hz, 1H), 7.08 (d, J = 7.6 Hz, 1H), 7.28 (d, J = 8.0 Hz, 2H), 7.34 (d, J = 8.8 Hz, 2H), 7.42 (d, J = 8.8 Hz, 2H), 7.61 (d, J = 8.4 Hz, 2H). ^{13}C NMR (100 MHz, $CDCl_3$, TMS) δ 18.5, 21.5, 28.4, 49.5, 54.3, 57.0, 57.3, 72.3, 109.1, 119.7, 120.5, 121.2, 124.5, 127.2, 127.7, 128.3, 129.7, 130.6, 130.8, 132.8, 135.5, 141.1, 142.5, 143.7, 151.2. IR (CH_2Cl_2): ν 3488, 3067, 3020, 2923, 2851, 2360, 2342, 1662, 1599, 1574, 1506, 1494, 1447, 1380, 1342, 1306, 1249, 1182, 1157, 1122, 1090, 1029, 988, 968, 911, 805, 790, 764,

700 cm^{-1}. HRMS (ESI) calcd. for $C_{30}H_{32}BrN_2O_3S$ $(M+H)^+$: 579.1312, Found: 579.1299. Enantiomeric excess was determined by HPLC with a Chiralcel AD-H column [λ = 254 nm; eluent: Hexane/Isopropanol = 70/30; Flow rate: 0.50 mL/min; t_{minor} = 18.17 min, t_{major} = 22.51 min; ee% > 99%; $[\alpha]^{20}_D$ = 32.7 (c 1.00, CH_2Cl_2)].

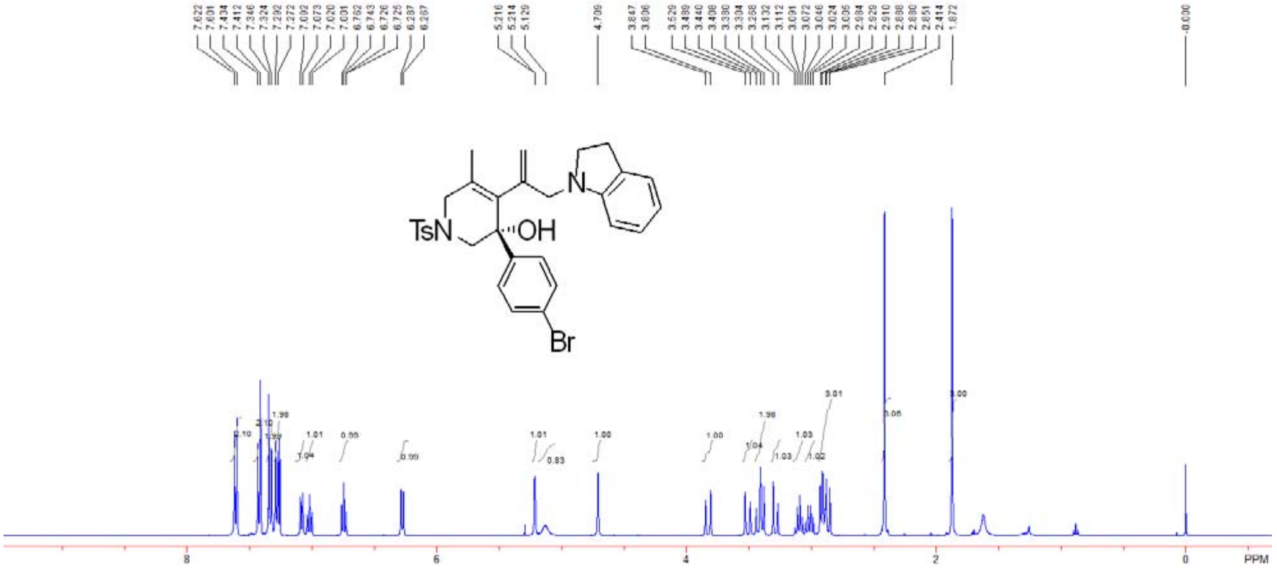

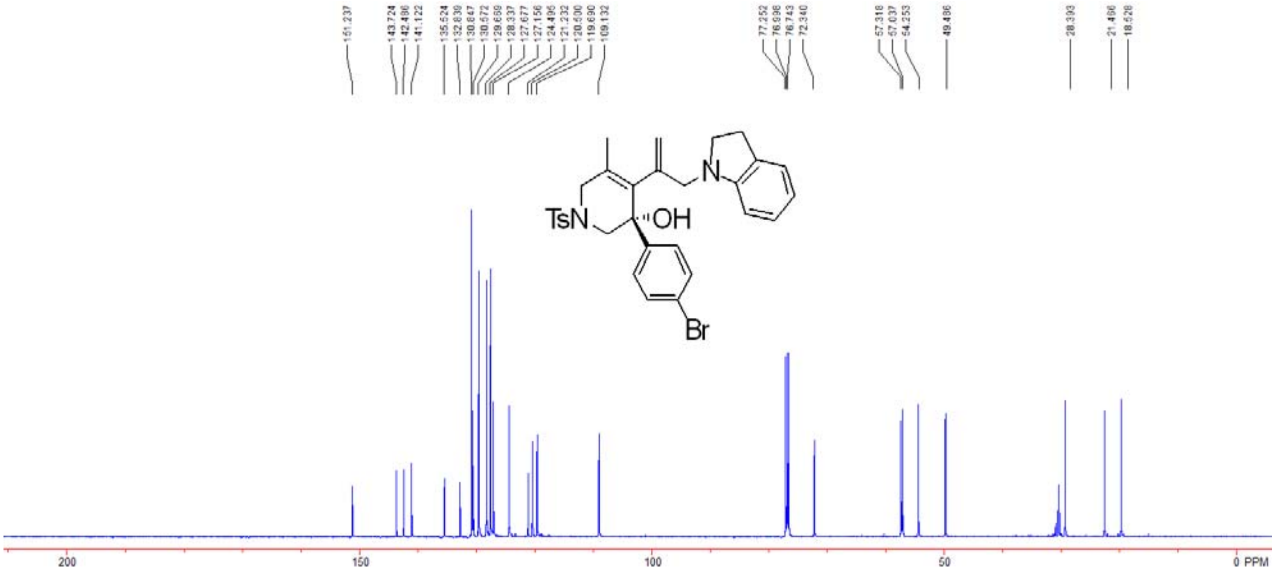

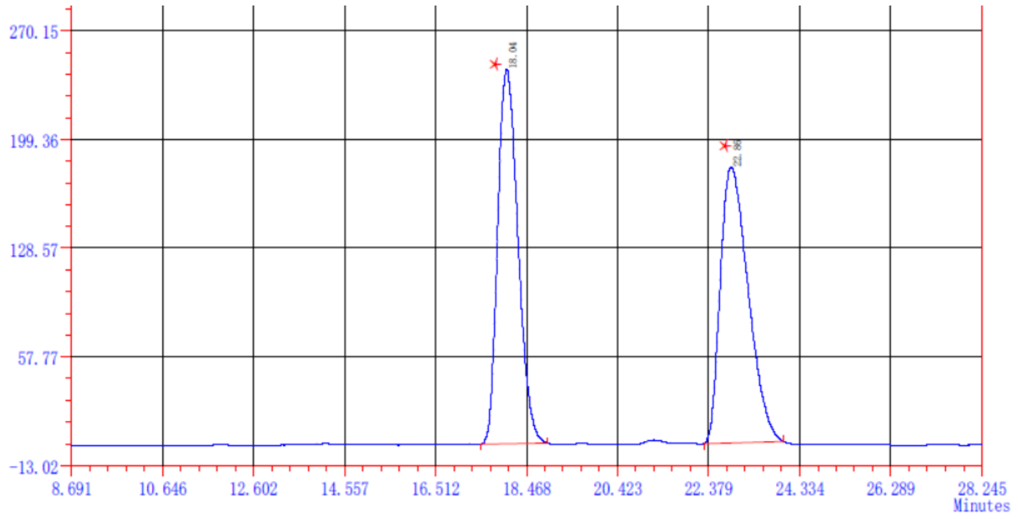

ID	组分名	保留时间	峰高	峰面积	浓度	拖尾因子	理论塔板
2	组份 2	18.037	244332	7373698.6	49.5520	1.27	7119
2		22.860	179760	7507020.8	50.4480	1.48	5972
	Σ:		424092	14880719.4	100.0000		

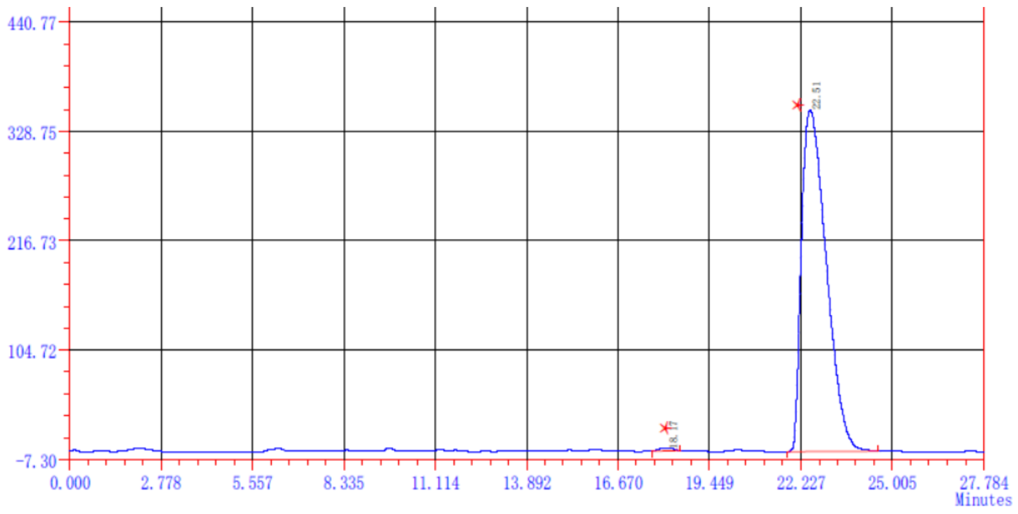

ID	组分名	保留时间	峰高	峰面积	浓度	拖尾因子	理论塔板
2	组份 2	18.172	2696	82728.3	0.4481	0.93	6989
2		22.510	350321	18381189.1	99.5519	1.71	3668
	Σ:		353017	18463917.4	100.0000		

Translation: Chiralcel AD-H column [λ = 254 nm; eluent: Hexane/Isopropanol = 70/30; Flow rate: 0.5 mL/min; t_{minor} = 18.17 min, t_{major} = 22.51 min; ee% > 99%].

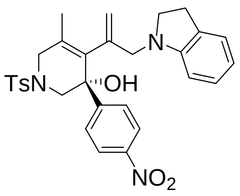

(S)-4-(3-(indolin-1-yl)prop-1-en-2-yl)-5-methyl-3-(4-nitrophenyl)-1-tosyl-1,2,3,6-tetrahydrop

yridin-3-ol (3pa)

A white solid. 83% yield (45 mg). M. P. 103-105 °C. ^{1}H NMR (400 MHz, $CDCl_3$, TMS) δ 1.90 (s, 3H), 2.41 (s, 3H), 2.90-2.94 (m, 3H), 3.01-3.09 (m, 3H), 3.30 (d, J = 12.0 Hz, 1H), 3.36 (d, J = 14.4 Hz, 1H), 3.40 (d, J = 14.4 Hz, 1H), 3.65 (d, J = 16.4 Hz, 1H), 3.76 (d, J = 16.4 Hz, 1H), 4.68 (s, 1H), 5.22 (d, J = 0.8 Hz, 1H), 5.69 (s, 1H), 6.36 (d, J = 7.6 Hz, 1H), 6.79 (dd, J_1 = 7.2 Hz, J_2 = 7.6 Hz, 1H), 7.02 (dd, J_1 = 7.2 Hz, J_2 = 7.6 Hz, 1H), 7.11 (d, J = 7.2 Hz, 1H), 7.29 (d, J = 8.4 Hz, 2H), 7.61 (d, J = 8.0 Hz, 2H), 7.68 (d, J = 8.8 Hz, 2H), 8.16 (d, J = 8.8 Hz, 2H). ^{13}C NMR (100 MHz, $CDCl_3$, TMS) δ 18.5, 21.5, 28.4, 49.5, 54.6, 56.8, 57.9, 72.6, 109.5, 120.4, 121.4, 123.1, 124.7, 127.2, 127.5, 127.7, 129.8, 130.8, 131.5, 132.8, 135.1, 141.0, 143.9, 147.1, 151.0, 151.3. IR (CH_2Cl_2): ν 3733, 3023, 2990, 2846, 2360, 2342, 1602, 1517, 1485, 1449, 1343, 1290, 1249, 1157, 1106, 1090, 1021, 988, 910, 872, 857, 811, 749, 705 cm^{-1}. HRMS (ESI) calcd. for $C_{30}H_{32}N_3O_5S$ $(M+H)^+$: 546.2057, Found: 546.2049. Enantiomeric excess was determined by HPLC with a Chiralcel IC-H column [λ = 254 nm; eluent: Hexane/Isopropanol = 70/30; Flow rate: 0.50 mL/min; t_{minor} = 33.90 min, t_{major} = 51.84 min; ee% > 99%; $[\alpha]^{20}_{D}$ = +2.1 (c 1.00, CH_2Cl_2)].

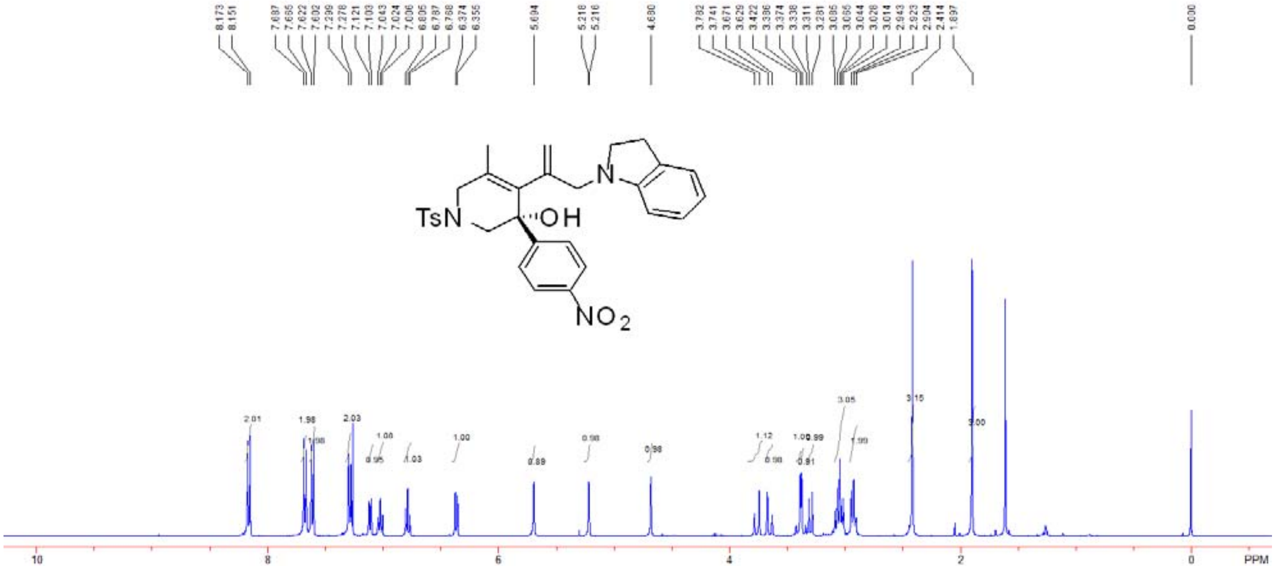

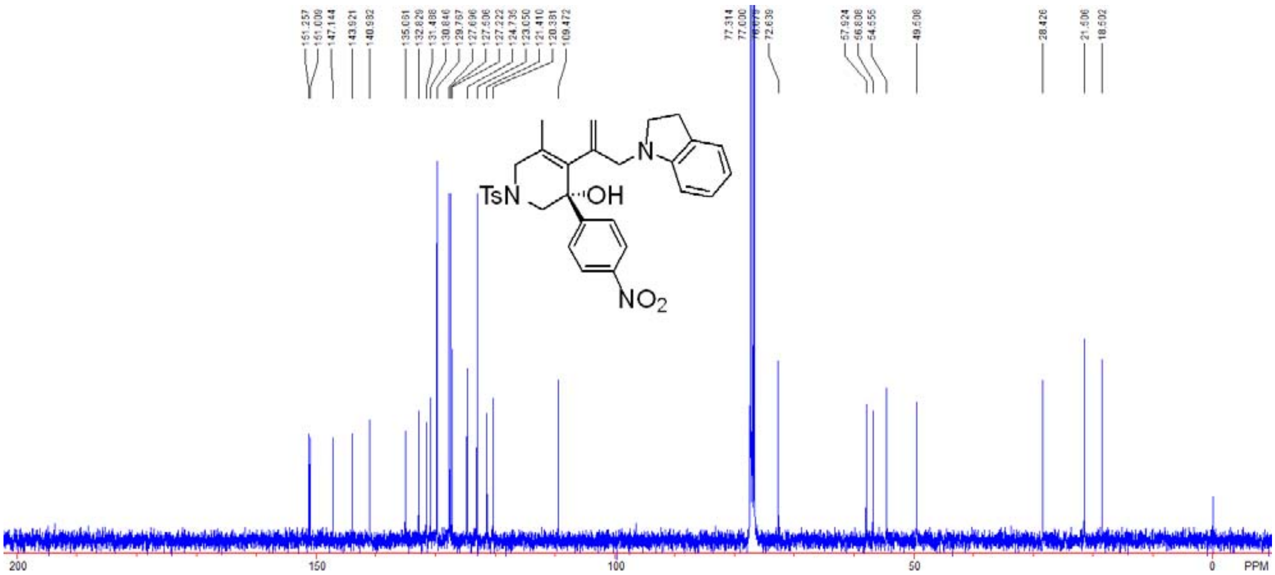

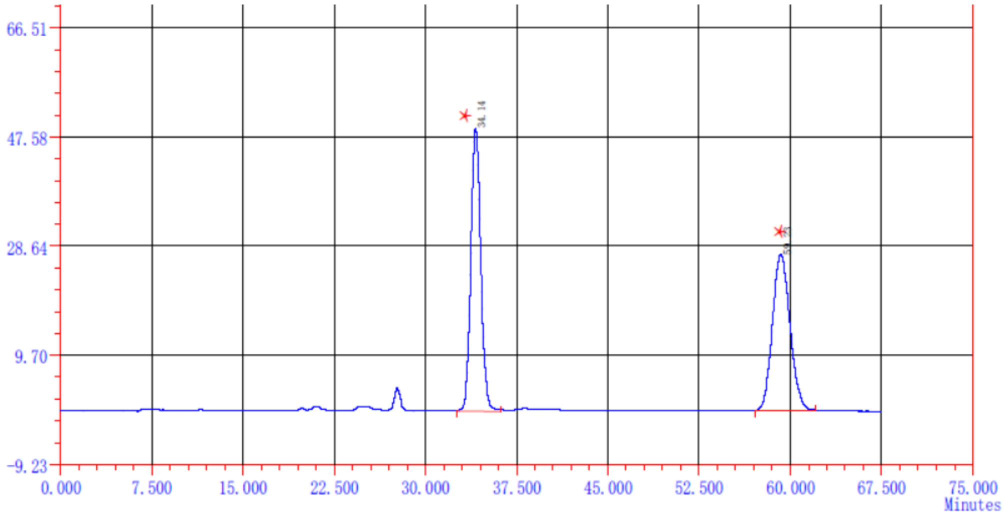

ID	组分名	保留时间	峰高	峰面积	浓度	拖尾因子	理论塔板
1		34.135	49106	2788270.5	50.4760	1.09	7203
2		59.230	27108	2735685.0	49.5240	1.10	6865
	Σ:		76214	5523955.5	100.0000		

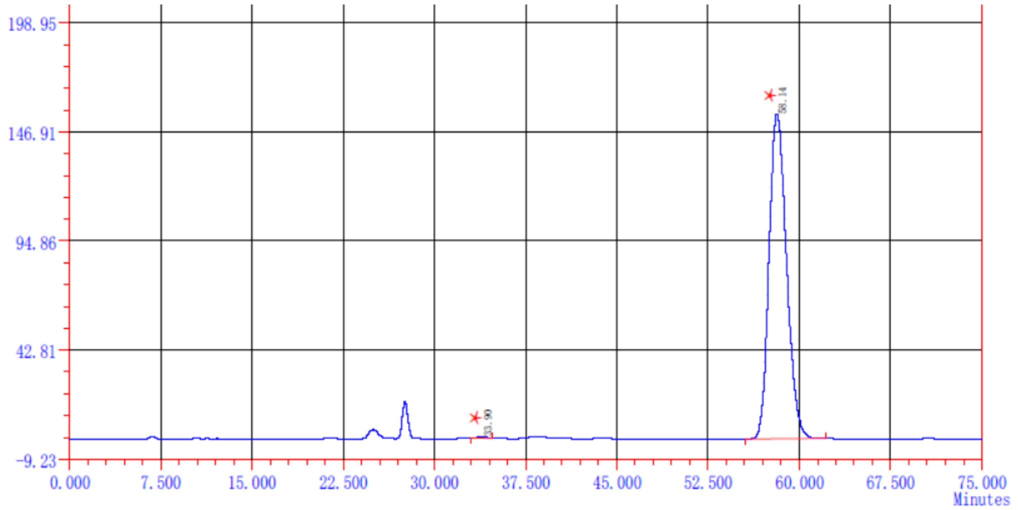

ID	组分名	保留时间	峰高	峰面积	浓度	拖尾因子	理论塔板
1		33.897	1048	55862.3	0.3575	0.90	8060
2		58.140	155181	15568798.0	99.6425	1.21	6693
	Σ:		156229	15624660.3	100.0000		

Translation: Chiralcel IC-H column [λ = 254 nm; eluent: Hexane/Isopropanol = 70/30; Flow rate: 0.5 mL/min; t_{minor} = 33.90 min, t_{major} = 58.14 min; ee% > 99%].

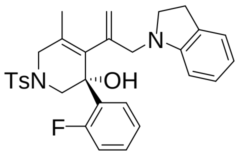

(R)-3-(2-fluorophenyl)-4-(3-(indolin-1-yl)prop-1-en-2-yl)-5-methyl-1-tosyl-1,2,3,6-tetrahydropyridin-3-ol (3qa)

A light yellow oil. 75% yield (39 mg). ^{1}H NMR (400 MHz, $CDCl_3$, TMS) δ 1.85 (s, 3H), 2.41 (s, 3H), 2.86 (d, J = 11.6 Hz, 1H), 2.92-3.00 (m, 3H), 3.12-3.17 (m, 1H), 3.24 (d, J = 14.8 Hz, 1H), 3.27 (d, J = 16.0 Hz, 1H), 3.58 (d, J = 14.8 Hz, 1H), 3.71 (d, J = 11.6 Hz, 1H), 4.06 (d, J = 16.0 Hz, 1H), 4.82 (s, 1H), 4.87 (brs, 1H), 5.10 (s, 1H), 6.34 (d, J = 8.0 Hz, 1H), 6.73 (dd, J_1 = 7.6 Hz, J_2 = 7.6 Hz, 1H), 6.89 (ddd, J_1 = 1.2 Hz, J_2 = 8.0 Hz, J_3 = 9.2 Hz, 1H), 7.01 (dd, J_1 = 7.6 Hz, J_2 = 7.6 Hz, 1H), 7.08 (d, J = 7.2 Hz, 1H), 7.22 (ddd, J_1 = 1.2 Hz, J_2 = 7.6 Hz, J_3 = 8.8 Hz, 1H), 7.26-7.30 (m, 3H), 7.66 (d, J = 8.0 Hz, 2H), 7.95 (ddd, J_1 = 1.6 Hz, J_2 = 8.0 Hz, J_3 = 9.2 Hz, 1H). ^{13}C NMR (100 MHz, $CDCl_3$, TMS) δ 18.4, 21.5, 28.4, 49.3, 54.1, 56.9, 71.2 (d, J = 3.7 Hz), 109.0, 115.5 (d, J = 23.6 Hz), 118.3, 119.3, 124.1 (d, J = 2.9 Hz), 124.4, 127.1, 127.7, 128.8 (d, J = 3.6 Hz), 129.46 (d, J = 5.8 Hz), 129.51, 129.7, 130.0 (d, J = 11.7 Hz), 130.5, 133.3, 134.9, 141.4,

143.6, 151.6, 159.0 (d, J = 245.2 Hz). ^{19}F NMR (376 MHz, $CDCl_3$) δ -111.7 (s, 1F). IR (CH_2Cl_2): ν 3483, 2987, 2969, 2923, 2360, 2342, 1605, 1487, 1450, 1347, 1265, 1211, 1186, 1169, 1152, 1122, 1088, 1038, 984, 947, 874, 861, 824, 812, 760, 746, 708 cm^{-1}. HRMS (ESI) calcd. for $C_{31}H_{35}N_2O_4S$ $(M+H)^+$: 531.2312, Found: 531.2313. Enantiomeric excess was determined by HPLC with a Chiralcel AD-H column [λ = 254 nm; eluent: Hexane/Isopropanol = 80/20; Flow rate: 0.50 mL/min; t_{minor} = 19.53 min, t_{major} = 17.13 min; ee% > 99%; $[\alpha]^{20}_D$ = +59.5 (c 1.00, CH_2Cl_2)].

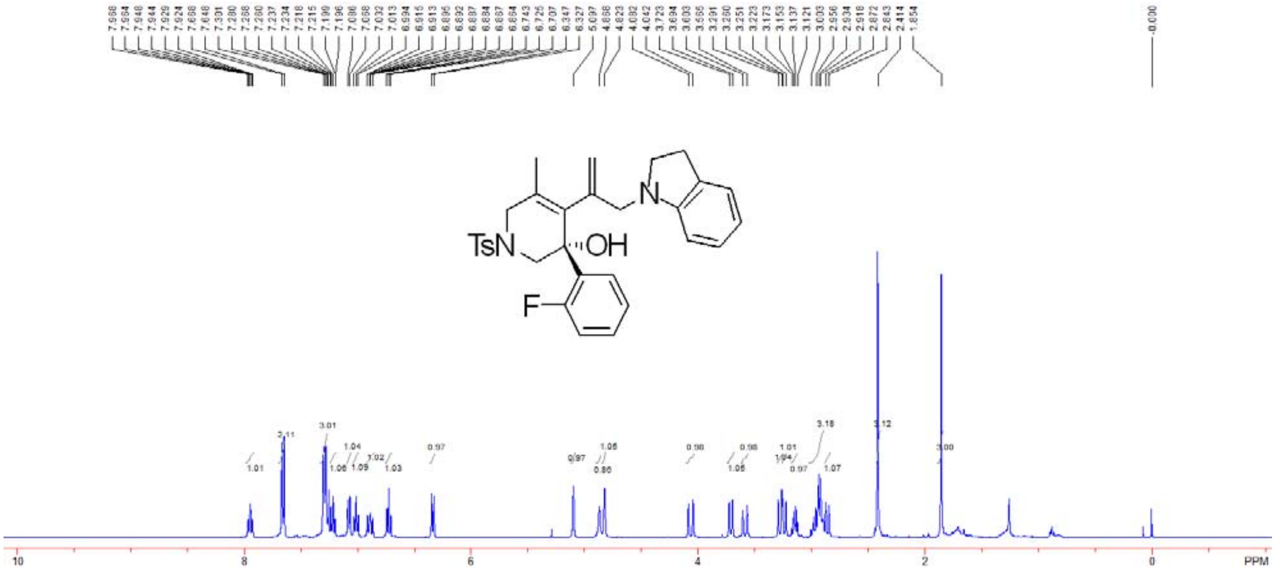

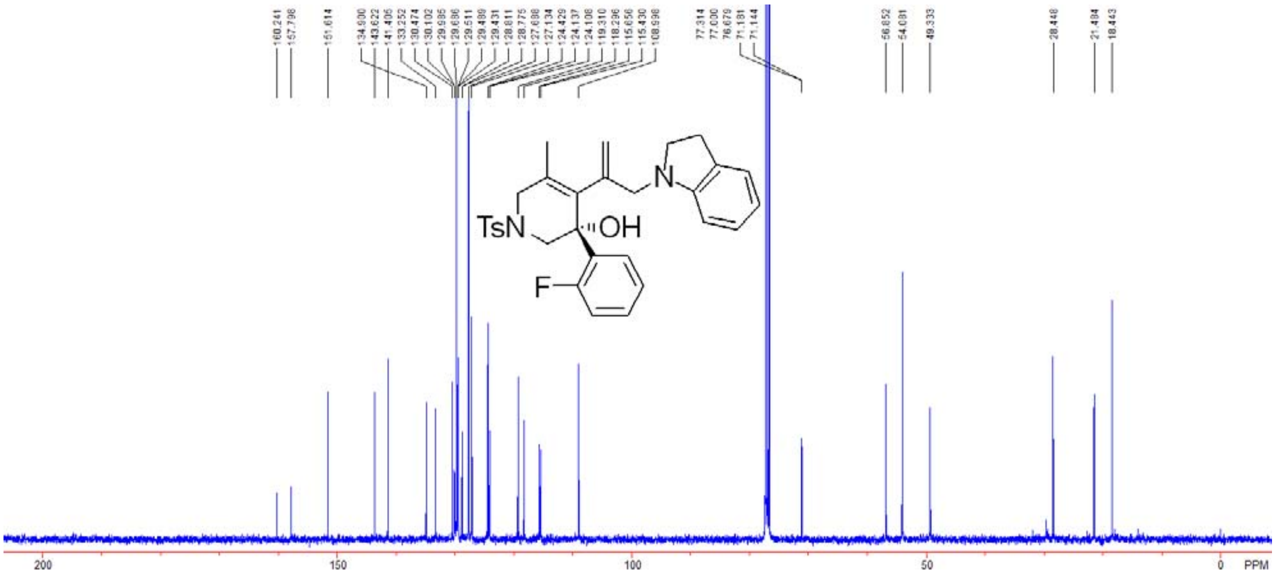

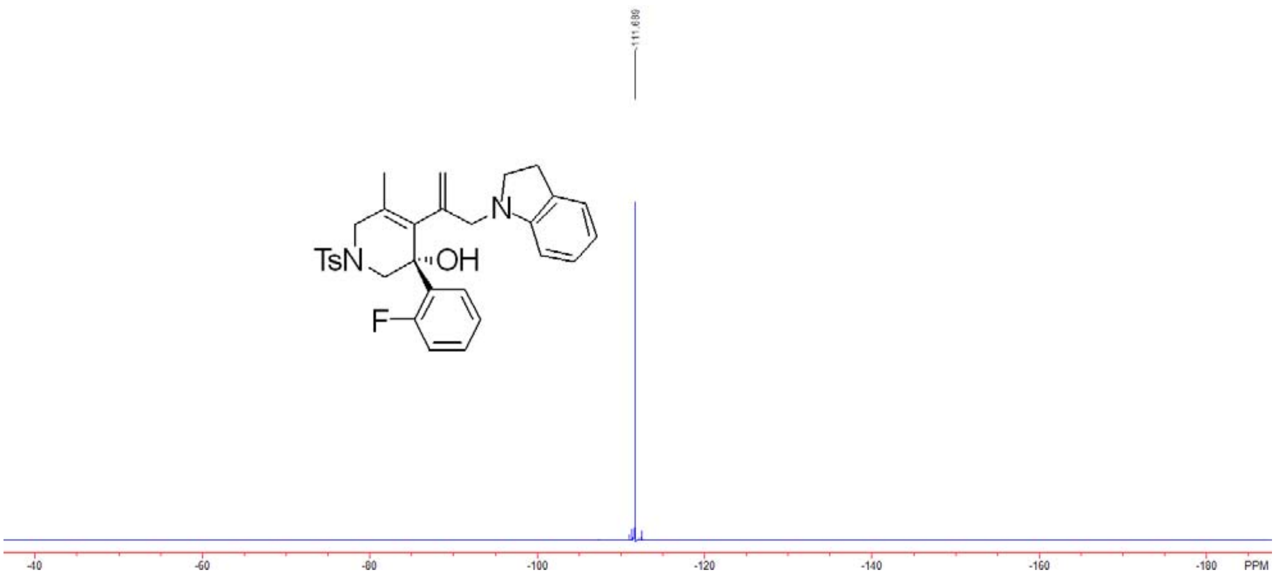

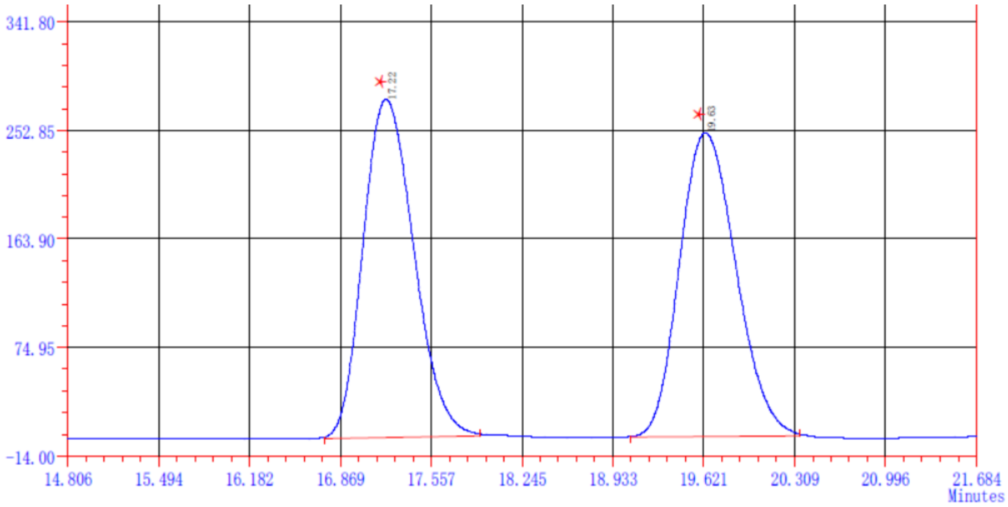

ID	组分名	保留时间	峰高	峰面积	浓度	拖尾因子	理论塔板
1		17.217	277085	7420410.1	50.0965	1.21	8237
4	组份 4	19.632	249003	7391819.1	49.9035	1.18	8717
	Σ:		526088	14812229.2	100.0000		

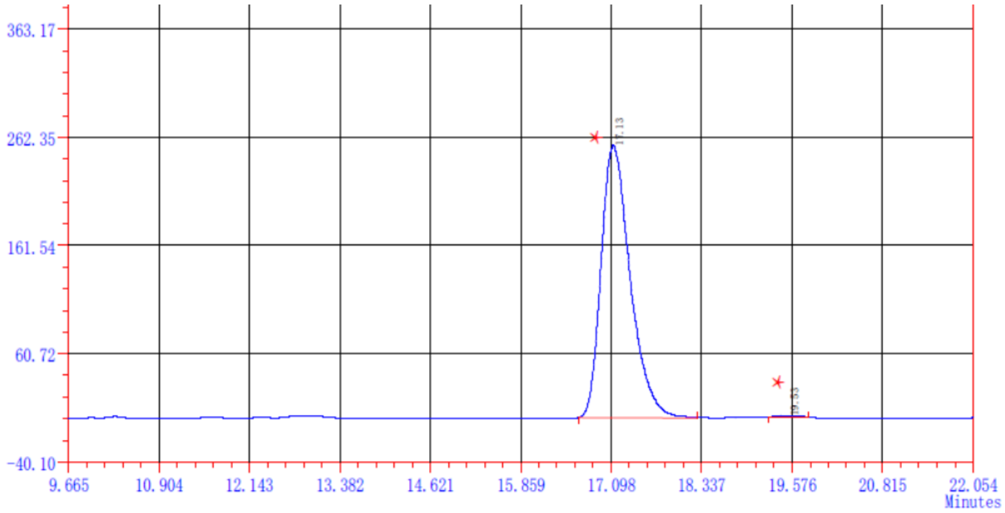

ID	组分名	保留时间	峰高	峰面积	浓度	拖尾因子	理论塔板
1		17.127	253706	7066603.2	99.5147	1.38	7535
3	组份 3	19.525	1792	34464.4	0.4853	0.99	20542
	Σ:		255498	7101067.6	100.0000		

Translation: Chiralcel AD-H column [λ = 254 nm; eluent: Hexane/Isopropanol = 80/20; Flow rate: 0.5 mL/min; t_{minor} = 19.53 min, t_{major} = 17.13 min; ee% > 99%].

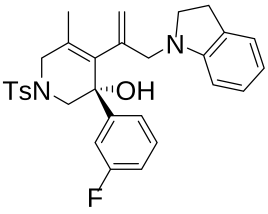

(S)-3-(3-fluorophenyl)-4-(3-(indolin-1-yl)prop-1-en-2-yl)-5-methyl-1-tosyl-1,2,3,6-tetrahydro pyridin-3-ol (3ra)

A light yellow oil. 74% yield (38 mg). ^{1}H NMR (400 MHz, $CDCl_3$, TMS) δ 1.87 (s, 3H), 2.41 (s, 3H), 2.89-2.93 (m, 3H), 3.02 (dd, J_1 = 8.8 Hz, J_2 = 16.4 Hz, 1H), 3.09 (dd, J_1 = 8.8 Hz, J_2 = 16.4 Hz, 1H), 3.31 (d, J = 14.8 Hz, 1H), 3.41-3.45 (m, 2H), 3.51 (d, J = 16.4 Hz, 1H), 3.83 (d, J = 16.4 Hz, 1H), 4.71 (s, 1H), 5.14 (brs, 1H), 5.21 (d, J = 1.2 Hz, 1H), 6.33 (d, J = 7.6 Hz, 1H), 6.74 (dd, J_1 = 7.2 Hz, J_2 = 7.6 Hz, 1H), 6.92-6.97 (m, 1H), 7.02 (dd, J_1 = 7.2 Hz, J_2 = 7.6 Hz, 1H), 7.08 (d, J = 7.2 Hz, 1H), 7.17-7.26 (m, 3H), 7.29 (d, J = 8.4 Hz, 2H), 7.63 (d, J = 8.4 Hz, 2H). ^{13}C NMR (100 MHz, $CDCl_3$, TMS) δ 18.5, 21.5, 28.4, 49.5, 54.3, 57.0, 57.4, 72.4 (d, J = 1.5 Hz), 109.1, 113.7 (d, J = 22.6 Hz), 114.0 (d, J = 21.1 Hz), 119.7, 120.5, 122.2 (d, J = 2.9 Hz), 124.5, 127.2, 127.7, 129.3 (d, J = 8.1 Hz), 129.7, 130.6, 130.8, 132.9, 135.6, 141.2, 143.7, 146.3 (d, J = 7.3 Hz),

151.3, 162.6 (d, J = 243.5 Hz). ^{19}F NMR (376 MHz, $CDCl_3$) δ -113.2 (s, 1F). IR (CH_2Cl_2): ν 3081, 2918, 2843, 2360, 2342, 1606, 1589, 1484, 1448, 1346, 1231, 1155, 1091, 843, 815, 788, 747, 705, 662 cm^{-1}. HRMS (ESI) calcd. for $C_{30}H_{32}FN_2O_3S$ $(M+H)^+$: 519.2112, Found: 519.2104. Enantiomeric excess was determined by HPLC with a Chiralcel IC-H column [λ = 254 nm; eluent: Hexane/Isopropanol = 70/30; Flow rate: 0.50 mL/min; t_{minor} = 23.35 min, t_{major} = 20.67 min; ee% > 99%; $[\alpha]^{20}_D$ = +46.2 (c 1.00, CH_2Cl_2)].

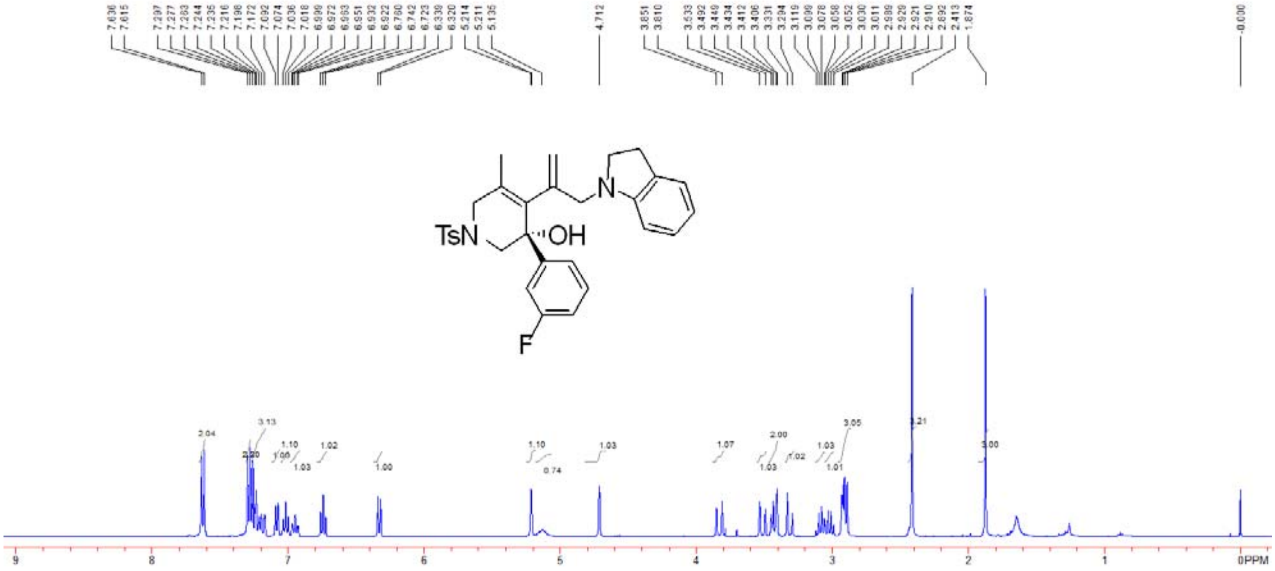

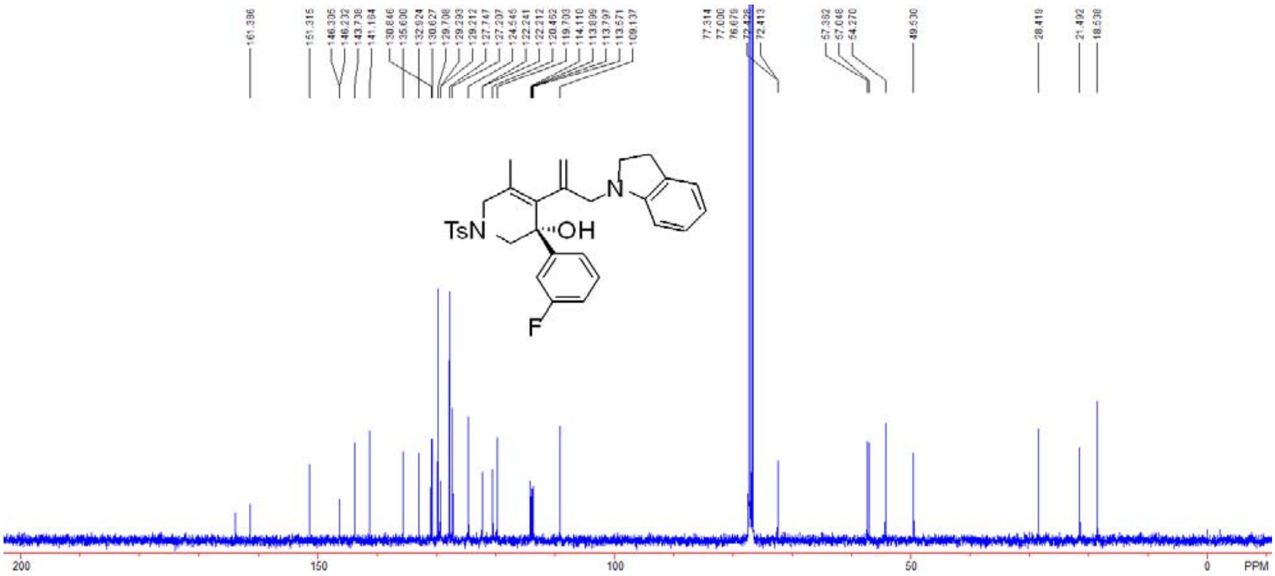

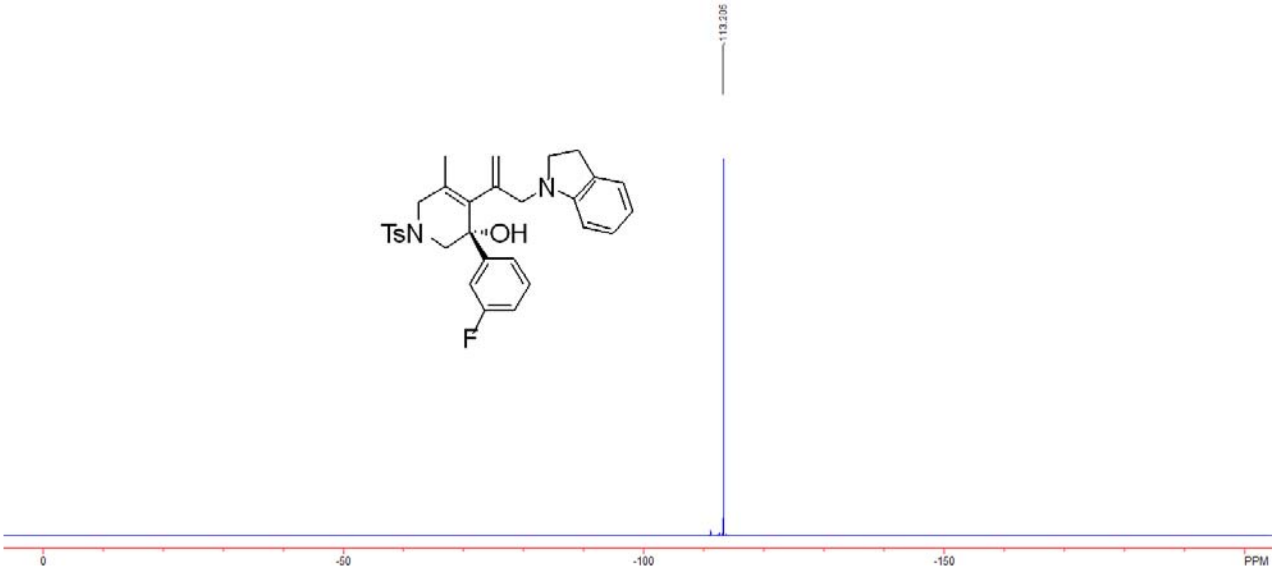

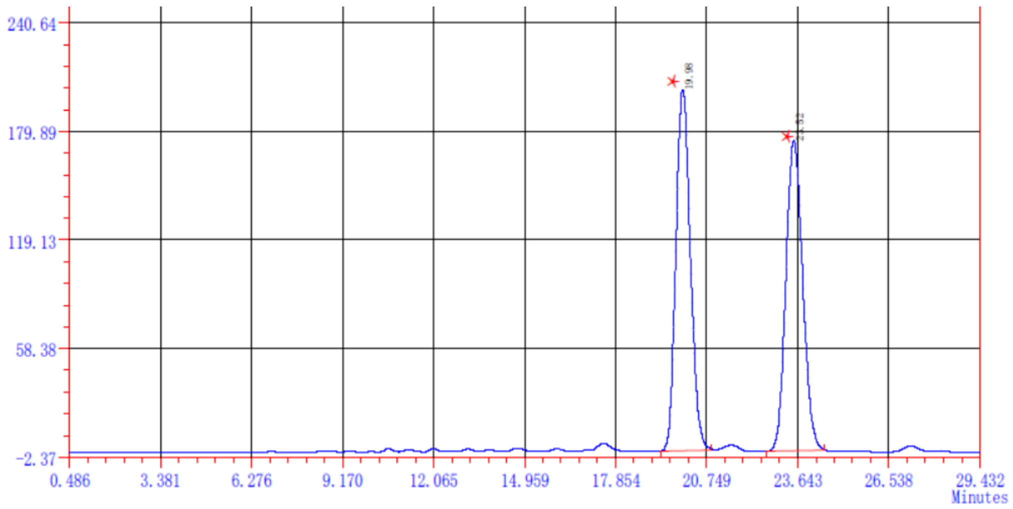

ID	组分名	保留时间	峰高	峰面积	浓度	拖尾因子	理论塔板
4	组份 4	19.983	202124	6453912.4	50.0323	1.15	7806
2		23.517	173398	6445574.4	49.9677	1.12	7977
	Σ:		375522	12899486.7	100.0000		

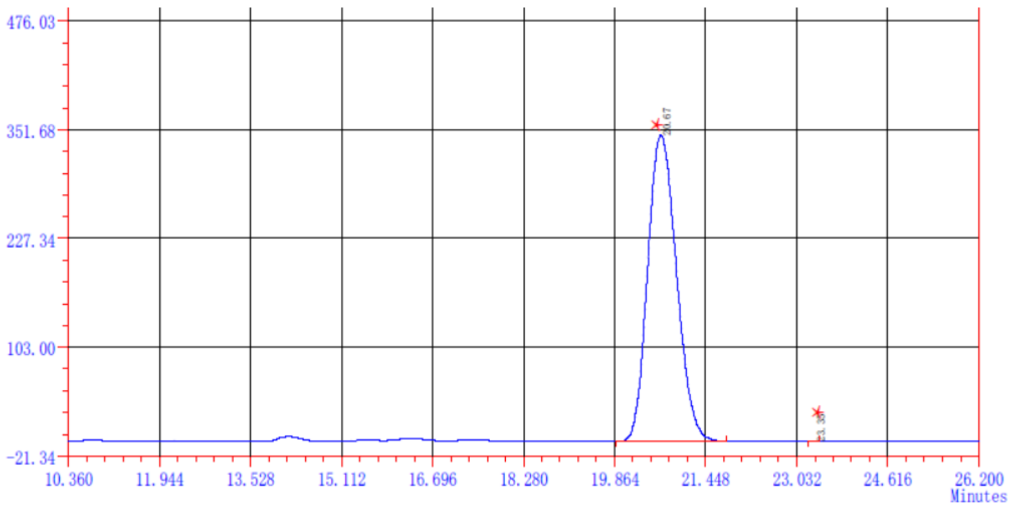

ID	组分名	保留时间	峰高	峰面积	浓度	拖尾因子	理论塔板
4	组份 4	20.672	350279	12260106.2	99.9957	1.16	6952
2		23.350	60	525.3	0.0043	0.83	141770
	Σ:		350339	12260631.5	100.0000		

Translation: Chiralcel IC-H column [λ = 254 nm; eluent: Hexane/Isopropanol = 70/30; Flow rate: 0.5 mL/min; t_{minor} = 23.35 min, t_{major} = 20.67 min; ee% > 99%].

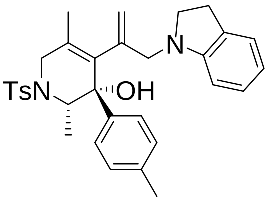

(2S,3S)-4-(3-(indolin-1-yl)prop-1-en-2-yl)-2,5-dimethyl-3-(p-tolyl)-1-tosyl-1,2,3,6-tetrahydropyridin-3-ol (*syn*-3sa)

0.2 mmol scale. A light yellow oil. 58% yield (61 mg). ^{1}H NMR (400 MHz, $CDCl_3$, TMS) δ 1.09 (d, *J* = 6.4 Hz, 3H), 1.90 (s, 3H), 2.33 (s, 3H), 2.38 (s, 3H), 2.91 (dd, J_1 = 8.0 Hz, J_2 = 8.0 Hz, 2H), 3.21-3.25 (m, 2H), 3.37 (d, *J* = 14.8 Hz, 1H), 3.47 (d, *J* = 14.8 Hz, 1H), 3.65 (d, *J* = 17.2 Hz, 1H), 3.83 (d, *J* = 17.2 Hz, 1H), 3.99 (q, *J* = 6.4 Hz, 1H), 4.34 (s, 1H), 4.86 (brs, 1H), 5.29 (d, *J* = 2.0 Hz, 1H), 6.05 (d, *J* = 7.6 Hz, 1H), 6.69 (dd, J_1 = 7.2 Hz, J_2 = 7.6 Hz, 1H), 6.91-6.97 (m, 3H), 7.04-7.09 (m, 3H), 7.19 (d, *J* = 8.0 Hz, 2H), 7.28 (d, *J* = 8.0 Hz, 2H). ^{13}C NMR (125 MHz, $CDCl_3$, TMS) δ 13.0, 17.7, 21,1 21.5, 28.5, 44.9, 54.8, 57.2, 58.7, 75.8, 108.5, 119.17, 119.23, 124.4, 126.5, 127.1, 127.2, 127.4, 128.5, 129.2, 129.9, 130.3, 134.2, 136.5, 141.3, 142.2, 142.7, 151.4. IR (CH_2Cl_2): ν 3029, 2962, 2920, 2849, 1605, 1485, 1458, 1378, 1330, 1304, 1288, 1264, 1240, 1222, 1163, 935, 915, 899, 857, 812, 735, 705 cm^{-1}. HRMS (ESI) calcd. for $C_{32}H_{37}N_2O_3S$ $(M+H)^+$: 529.2519,

Found: 529.2508. Enantiomeric excess was determined by HPLC with a Chiralcel AD-H column [λ = 254 nm; eluent: Hexane/Isopropanol = 80/20; Flow rate: 0.50 mL/min; t_{minor} = 18.24 min, t_{major} = 21.67 min; ee% = 99%; $[\alpha]^{20}_{D}$ = -10.6 (c 1.00, CH_2Cl_2)].

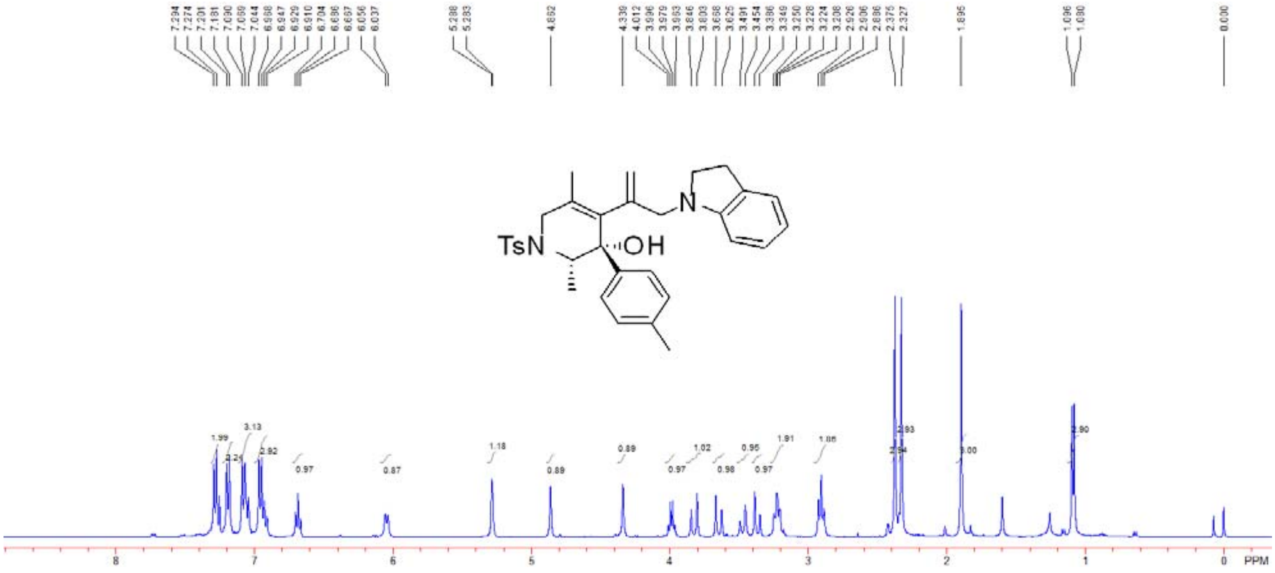

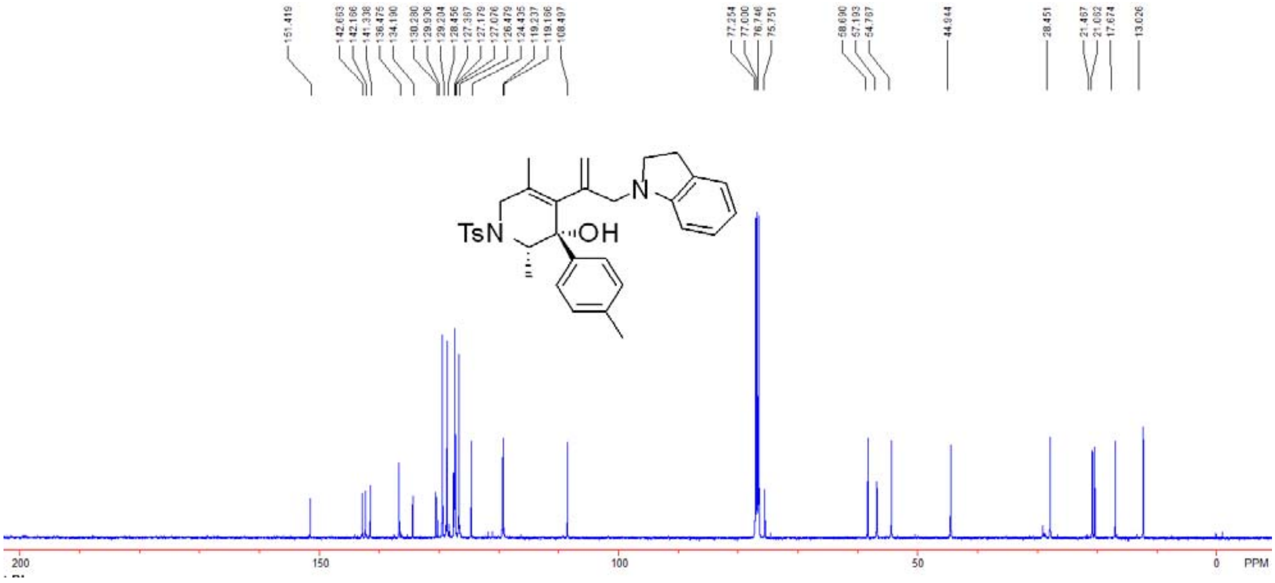

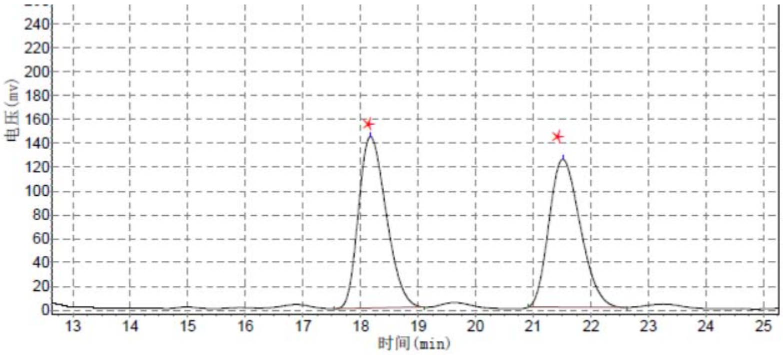

分析结果表

峰号	峰名	保留时间	峰高	峰面积	含量
1		18. 170	143981. 766	4807147. 500	50. 6963
2		21. 510	123990. 281	4675089. 500	49. 3037
总计			267972. 047	9482237. 000	100. 0000

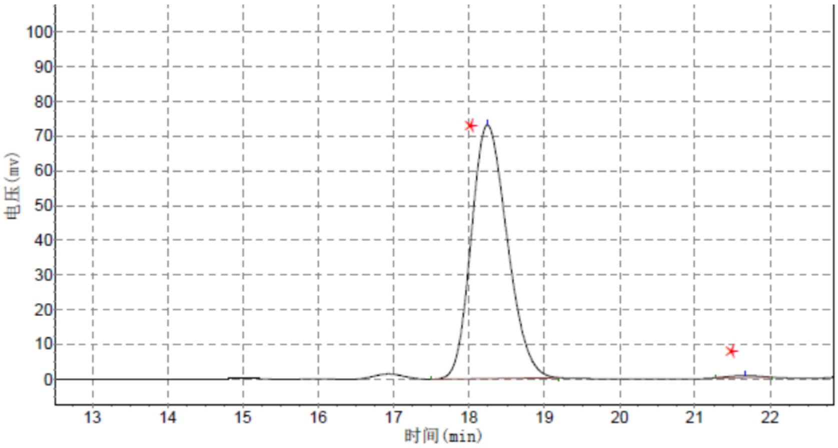

分析结果表

峰号	峰名	保留时间	峰高	峰面积	含量
1		18. 243	73092. 391	2424701. 750	99. 3524
2		21. 665	588. 969	15805. 798	0. 6476
总计			73681. 360	2440507. 548	100. 0000

Translation: Chiralcel AD-H column [λ = 254 nm; eluent: Hexane/Isopropanol = 80/20; Flow rate: 0.5 mL/min; t_{minor} = 18.24 min, t_{major} = 21.67 min; ee% = 99%].

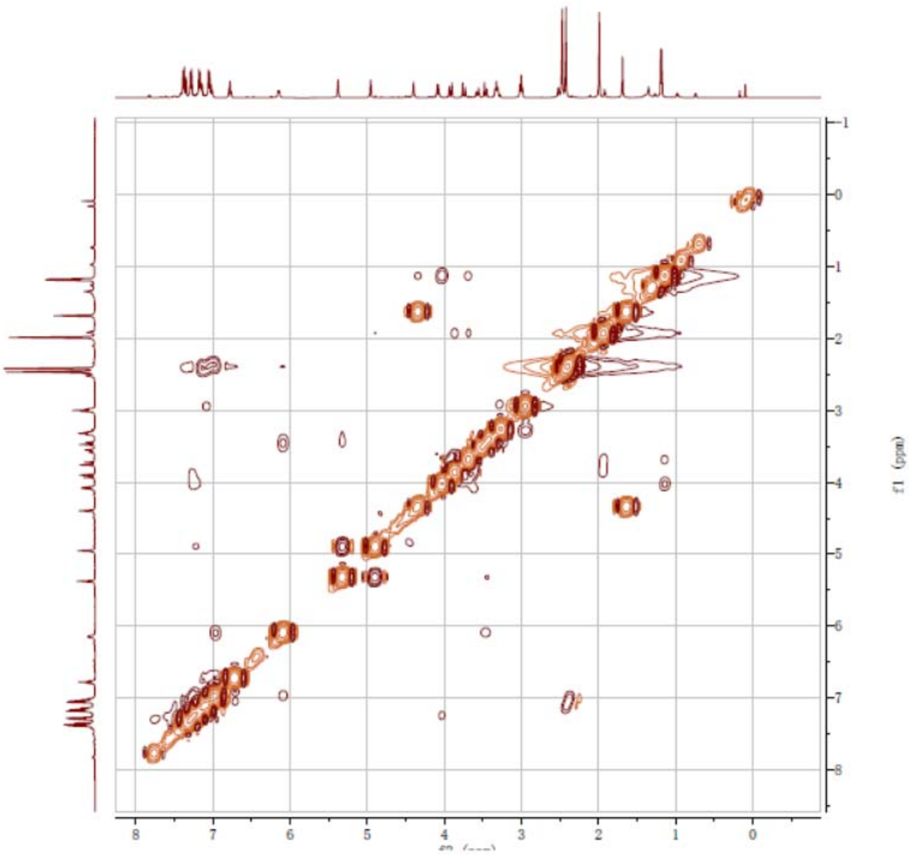

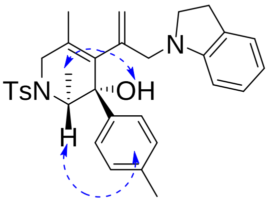

TsN
OH
H
N

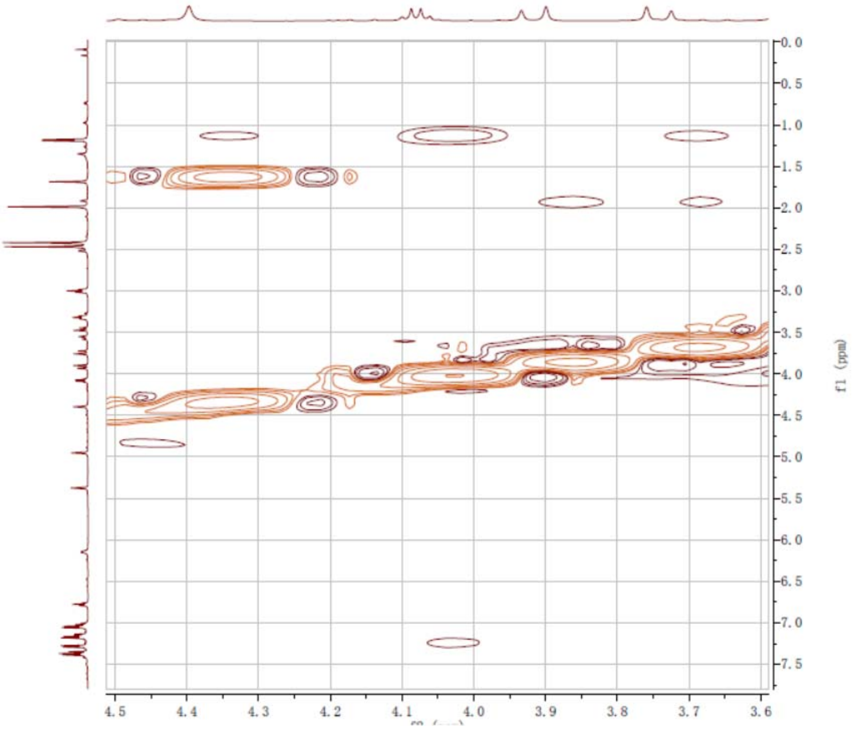

Nuclear Overhauser Effect Spectroscopy (NOESY) of 3

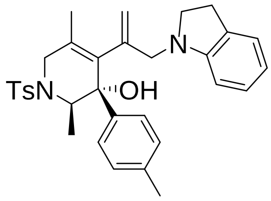

(2R,3S)-4-(3-(indolin-1-yl)prop-1-en-2-yl)-2,5-dimethyl-3-(p-tolyl)-1-tosyl-1,2,3,6-tetrahydro pyridin-3-ol (*anti*-3sa)

0.2 mmol scale. A light yellow oil. 15% yield (12 mg). ^{1}H NMR (400 MHz, $CDCl_3$, TMS) δ 0.70 (d, J = 6.8 Hz, 3H), 1.94 (s, 3H), 2.35 (s, 3H), 2.37 (s, 3H), 2.77-2.87 (m, 3H), 3.01-3.09 (m, 2H), 3.46 (d, J = 15.2 Hz, 1H), 3.69 (d, J = 17.6 Hz, 1H), 3.98-4.02 (m, 2H), 4.65 (s, 1H), 4.96 (s, 1H), 5.37 (s, 1H), 6.08 (d, J = 7.6 Hz, 1H), 6.69 (dd, J_1 = 7.2, J_1 = 7.6, Hz, 1H), 6.95 (dd, J_1 = 7.6 Hz, J_2 = 7.6 Hz, 1H), 7.04 (d, J = 7.2 Hz, 1H), 7.09 (d, J = 8.0 Hz, 2H), 7.21 (d, J = 8.0 Hz, 2H), 7.38 (d, J = 7.2 Hz, 2H) 7.75 (d, J = 8.0 Hz, 2H). ^{13}C NMR (125 MHz, $CDCl_3$, TMS) δ 13.5, 19.1, 21.1, 21.5, 28.4, 45.2, 53.7, 56.7, 59.3, 74.5, 109.0, 119.0, 119.5, 124.2, 127.1, 127.4, 127.8, 128.0, 129.4, 130.3, 131.9, 134.8, 136.8, 137.1, 138.8, 142.5, 143.1, 151.6. IR (CH_2Cl_2): ν 3499, 3086, 3057, 3029, 2975, 2917, 2845, 1749, 1598, 1554, 1494, 1479, 1448, 1399, 1348, 1310, 1251, 1154, 1120, 1091, 1039, 988, 918, 863, 814, 766, 748, 704 cm^{-1}. HRMS (ESI) calcd. for $C_{32}H_{37}N_2O_3S$ $(M+H)^+$: 529.2519, Found: 529.2509. Enantiomeric excess was determined by HPLC with a Chiralcel AD-H column [λ = 254 nm; eluent: Hexane/Isopropanol = 80/20; Flow rate: 0.50 mL/min; t_{minor} = 12.62 min, t_{major} = 22.86 min; ee% = 95%; $[\alpha]^{20}_{D}$ = +13.8 (c 1.00, CH_2Cl_2)].

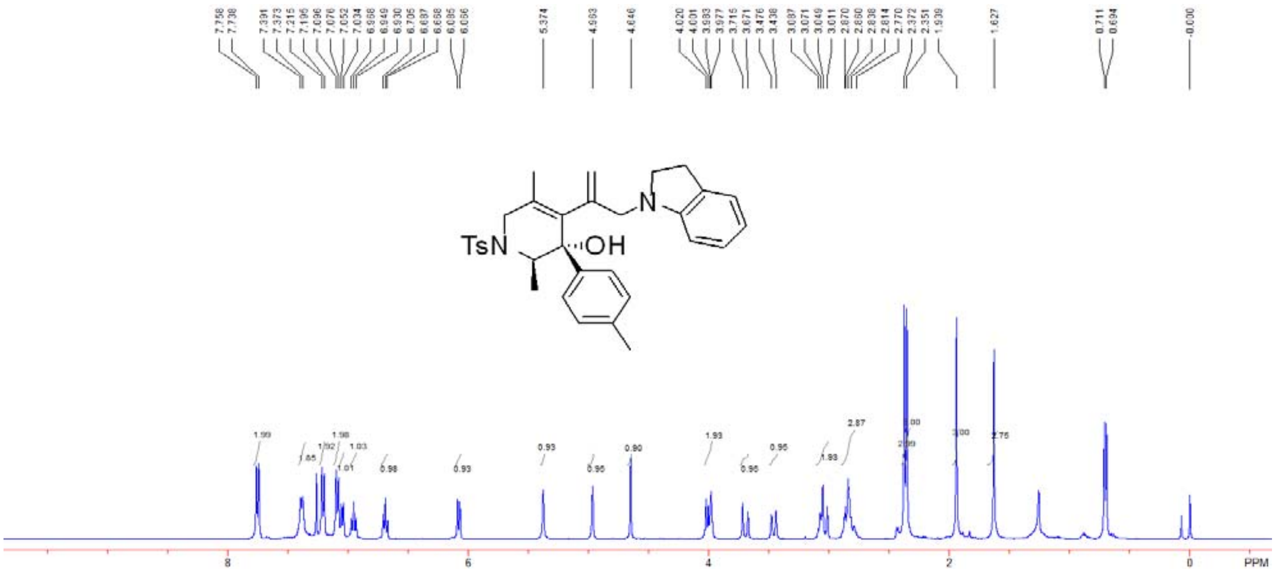

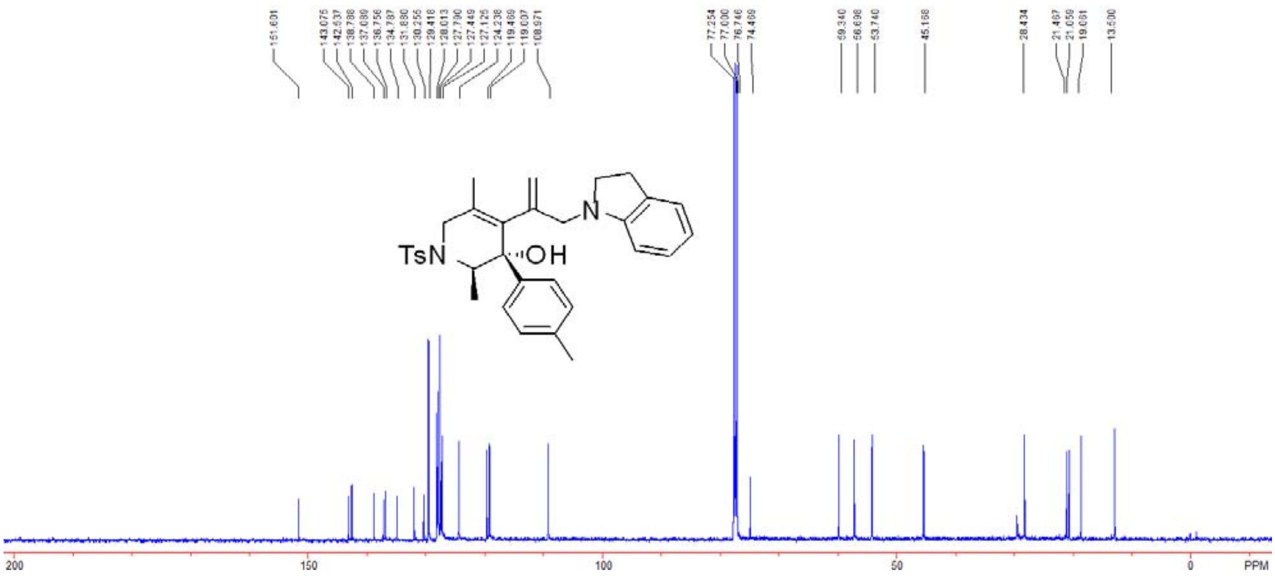

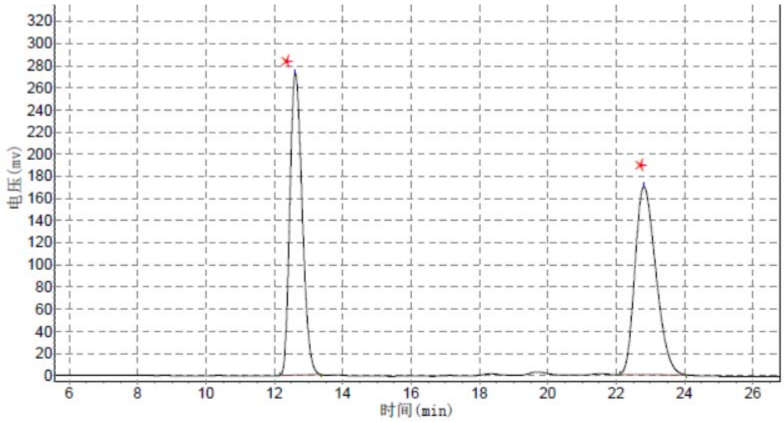

分析结果表

峰号	峰名	保留时间	峰高	峰面积	含量
1		12.617	272696.313	6826212.000	48.6209
2		22.812	169817.609	7213457.500	51.3791
总计			442513.922	14039669.500	100.0000

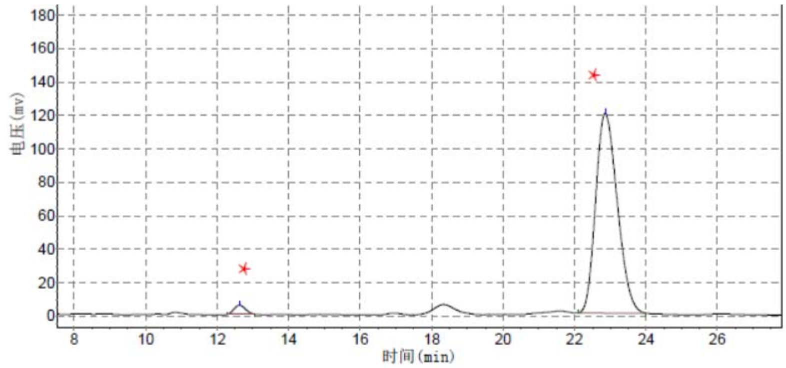

分析结果表

峰号	峰名	保留时间	峰高	峰面积	含量
1		12.617	5410.445	119951.695	2.3303
2		22.855	119497.422	5027567.500	97.6697
总计			124907.867	5147519.195	100.0000

Translation: Chiralcel AD-H column [λ = 254 nm; eluent: Hexane/Isopropanol = 80/20; Flow rate: 0.5 mL/min; t_{minor} = 12.62 min, t_{major} = 22.86 min; ee% = 95%].

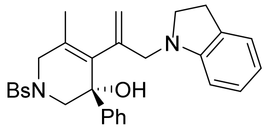

(S)-1-((4-bromophenyl)sulfonyl)-4-(3-(indolin-1-yl)prop-1-en-2-yl)-5-methyl-3-phenyl-1,2,3,6-tetrahydropyridin-3-ol (3ta)

A white solid. 83% yield (47 mg). M. P. 153-155 ^{o}C. ^{1}H NMR (400 MHz, $CDCl_3$, TMS) δ 1.89 (s, 3H), 2.88-2.91 (m, 3H), 2.93-3.00 (m, 1H), 3.04-3.10 (m, 1H), 3.24 (d, J = 14.8 Hz, 1H), 3.43 (d, J = 14.8 Hz, 1H), 3.51 (d, J = 16.4 Hz, 1H), 3.53 (d, J = 11.6 Hz, 1H), 3.90 (d, J = 16.4 Hz, 1H), 4.74 (s, 1H), 5.09 (brs, 1H), 5.20 (d, J = 1.2 Hz, 1H), 6.31 (d, J = 7.6 Hz, 1H), 6.74 (ddd, J_1 = 0.8 Hz, J_2 = 7.6 Hz, J_3 = 8.0 Hz, 1H), 7.01 (dd, J_1 = 7.6 Hz, J_2 = 8.0 Hz, 1H), 7.08 (d, J = 7.2 Hz, 1H), 7.25-7.33 (m, 3H), 7.45 (d, J = 8.4 Hz, 2H), 7.58-7.62 (m, 4H). ^{13}C NMR (100 MHz, $CDCl_3$, TMS) δ 18.5, 28.4, 49.3, 54.1, 57.1, 57.3, 72.5, 109.2, 119.6, 120.3, 124.5, 126.4, 127.16, 127.20, 127.79, 129.2, 130.3, 130.6, 132.3, 135.4, 136.1, 141.3, 143.1, 151.3. IR (CH_2Cl_2): ν 3225, 3048, 2987, 2919, 2851, 2360, 2342, 1605, 1575, 1483, 1471, 1460, 1446, 1388, 1231, 1206, 1170, 1153, 1115, 1091, 1066, 1053, 1024, 1011, 994, 929, 869, 829, 809, 784, 765, 749, 737, 709 cm^{-1}. HRMS (ESI) calcd. for $C_{29}H_{30}BrN_2O_3S$ $(M+H)^+$: 565.1155, Found: 565.1146. Enantiomeric excess was determined by HPLC with a Chiralcel IC-H column [λ = 230 nm; eluent: Hexane/Isopropanol = 80/20; Flow rate: 0.50 mL/min; t_{minor} = 27.86 min, t_{major} = 33.21 min; ee% > 99%; $[\alpha]^{20}_{D}$ = 50.6 (c 1.00, CH_2Cl_2)].

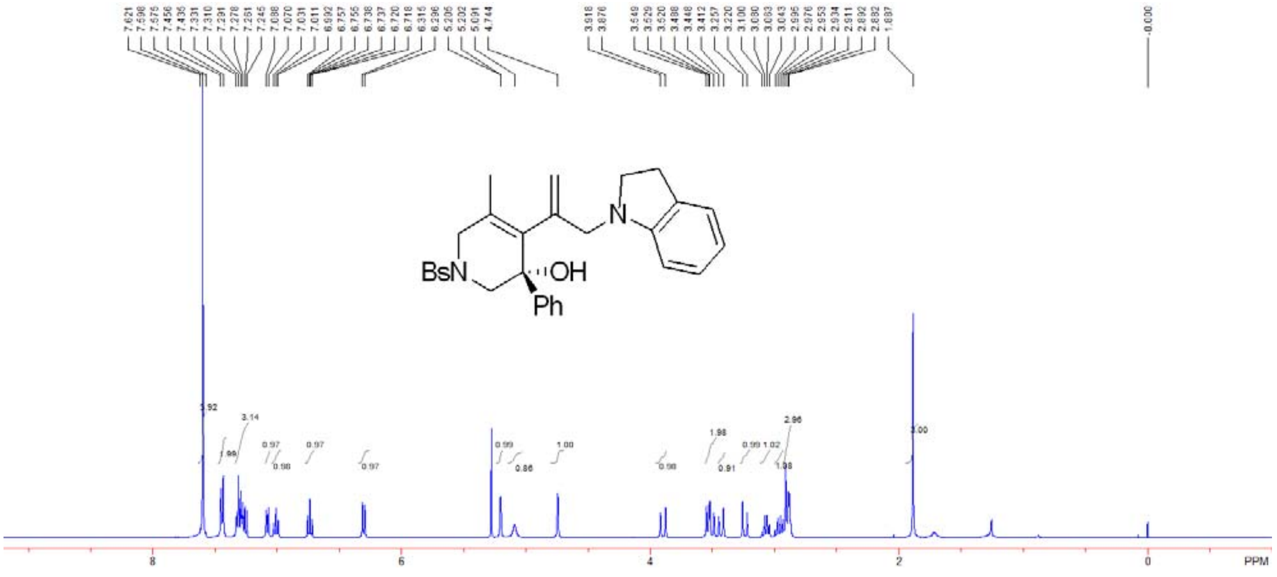

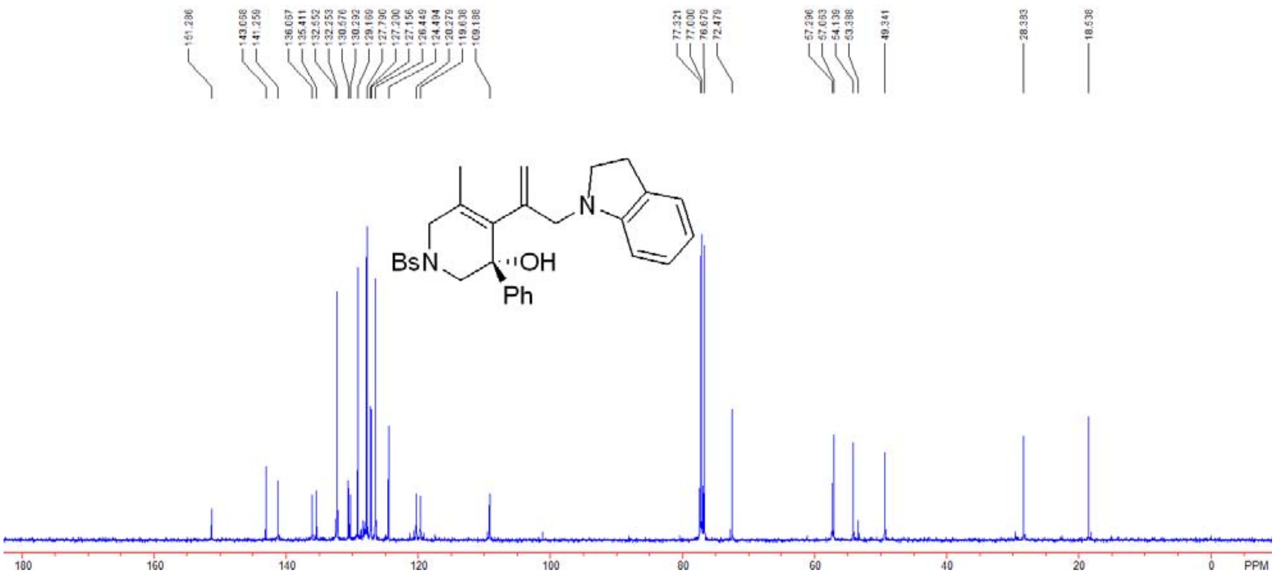

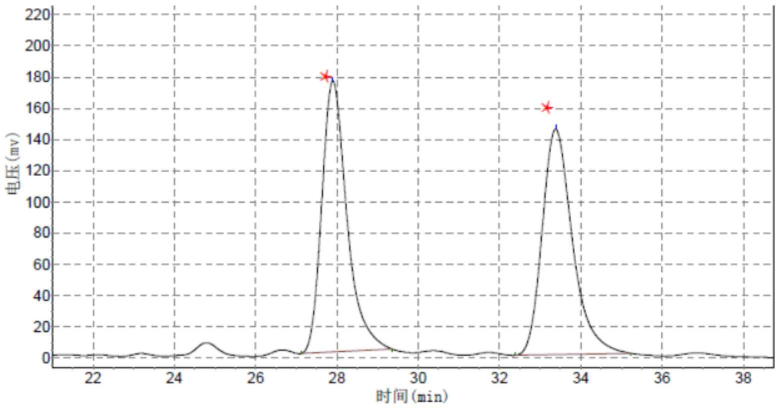

分析结果表

峰号	峰名	保留时间	峰高	峰面积	含量
1		27.900	173625.359	7440871.000	49.9061
2		33.380	144383.297	7468864.000	50.0939
总计			318008.656	14909735.000	100.0000

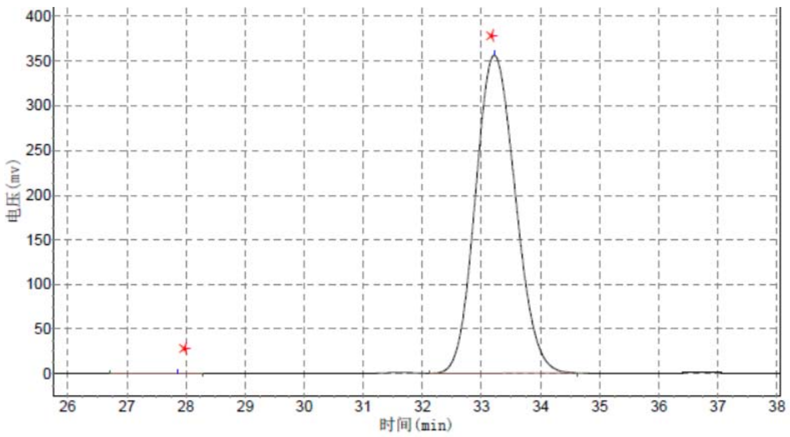

分析结果表

峰号	峰名	保留时间	峰高	峰面积	含量
1		27.858	606.896	25421.248	0.1521
2		33.213	355284.250	16684778.000	99.8479
总计			355891.146	16710199.248	100.0000

Translation: Chiralcel IC-H column [λ = 230 nm; eluent: Hexane/Isopropanol = 80/20; Flow rate:

0.5 mL/min; t_{minor} = 27.86 min, t_{major} = 33.21 min; ee% > 99%].

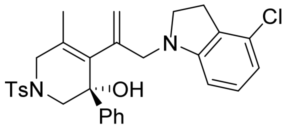

(S)-4-(3-(4-chloroindolin-1-yl)prop-1-en-2-yl)-5-methyl-3-phenyl-1-tosyl-1,2,3,6-tetrahydropyridin-3-ol (3ab)

A white solid. 79% yield (42 mg). M. P. 92-94 ^{o}C. ^{1}H NMR (400 MHz, $CDCl_3$, TMS) δ 1.85 (s, 3H), 2.42 (s, 3H), 2.83 (d, *J* = 11.6 Hz, 1H), 2.91-2.96 (m, 2H), 3.05-3.21 (m, 2H), 3.30 (d, *J* = 16.0 Hz, 1H), 3.40 (d, *J* = 16.4 Hz, 1H), 3.41 (d, *J* = 16.0 Hz, 1H), 3.56 (d, *J* = 11.6 Hz, 1H), 3.92 (d, *J* = 16.4 Hz, 1H), 3.97 (s, 1H), 4.71 (s, 1H), 5.13 (d, *J* = 1.2 Hz, 1H), 5.95 (d, *J* = 8.0 Hz, 1H), 6.61 (d, *J* = 8.0 Hz, 1H), 6.87 (dd, J_1 = 8.0 Hz, J_2 = 8.0 Hz, 1H), 7.27-7.33 (m, 5H), 7.43 (d, *J* = 6.8 Hz, 2H), 7.62 (d, *J* = 8.0 Hz, 2H). ^{13}C NMR (100 MHz, $CDCl_3$, TMS) δ 18.5, 21.5, 27.7, 49.7, 53.1, 55.7, 57.5, 72.7, 106.0, 118.3, 119.0, 126.5, 127.27, 127.81, 127.84, 127.98, 128.0, 128.7, 129.8, 130.3, 130.6, 132.8, 136.0, 140.8, 142.3, 143.8, 153.0. IR (CH_2Cl_2): ν 3500, 3057, 3029, 2951, 2917, 2832, 1635, 1599, 1480, 1446, 1430, 1383, 1350, 1307, 1293, 1251, 1188, 1174, 1155, 1121, 1091, 1054, 1039, 1019, 986, 920, 901, 865, 809, 794, 765, 704, 683, 661 cm^{-1}. HRMS (ESI) calcd. for $C_{30}H_{32}ClN_2O_3S$ $(M+H)^+$: 535.1817, Found: 535.1810. Enantiomeric excess was determined by HPLC with a Chiralcel IC-H column [λ = 254 nm; eluent: Hexane/Isopropanol = 70/30; Flow rate: 0.50 mL/min; t_{minor} = 19.64 min, t_{major} = 22.98 min; ee% > 99%; $[\alpha]^{20}_{D}$ = +40.2 (c 1.00, CH_2Cl_2)].

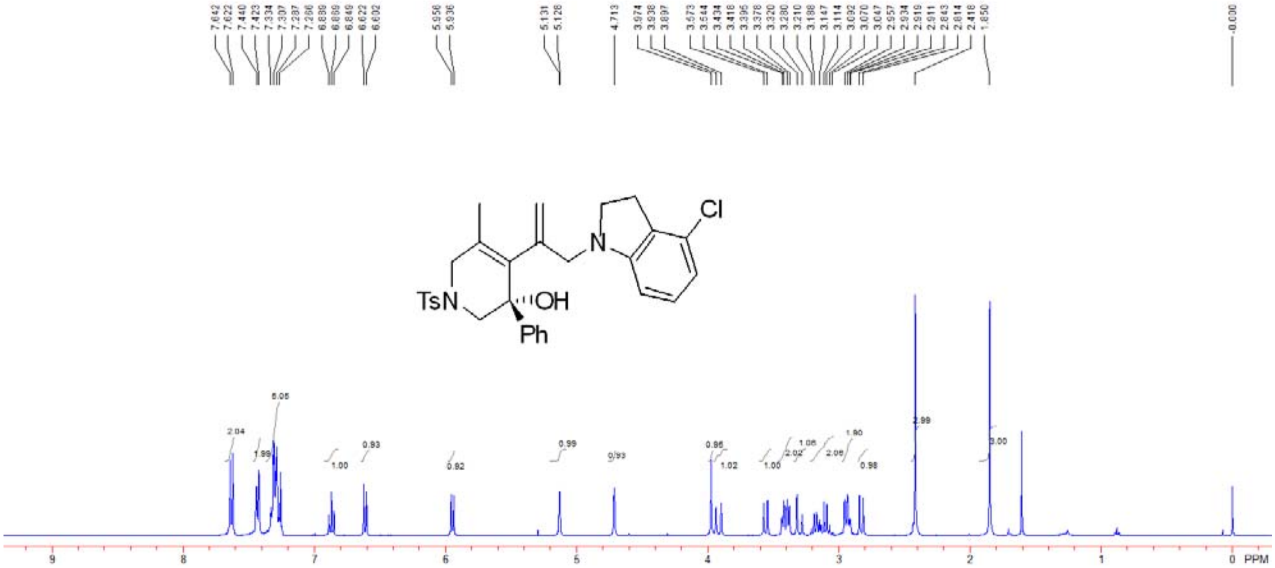

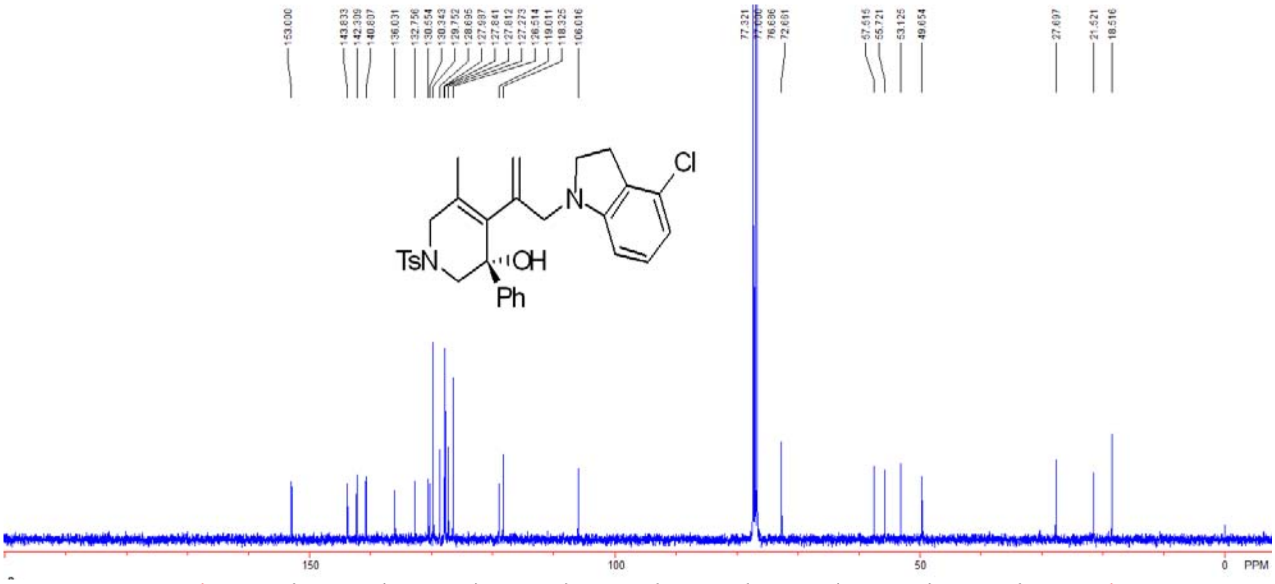

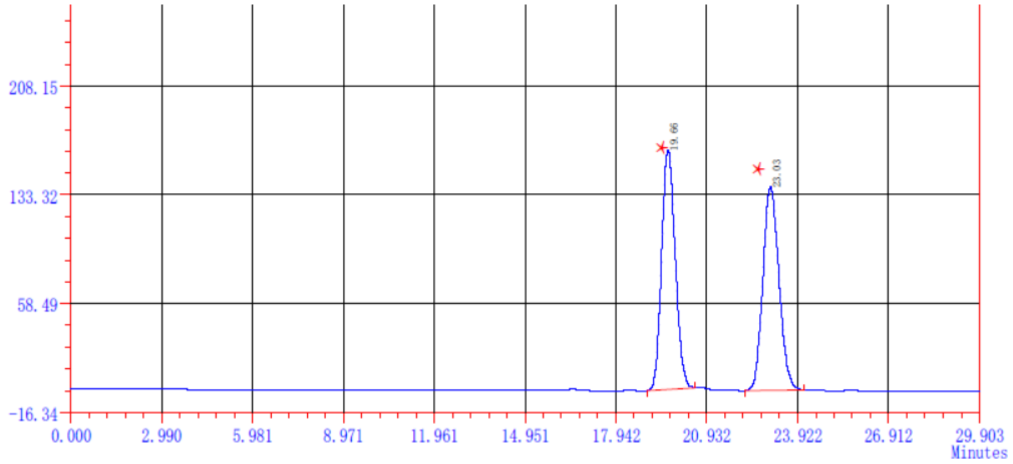

ID	组分名	保留时间	峰高	峰面积	浓度	拖尾因子	理论塔板
4	组份 4	19.660	164348	5150061.2	49.7880	1.13	7845
2		23.028	139849	5193926.2	50.2120	1.12	7663
	Σ:		304197	10343987.4	100.0000		

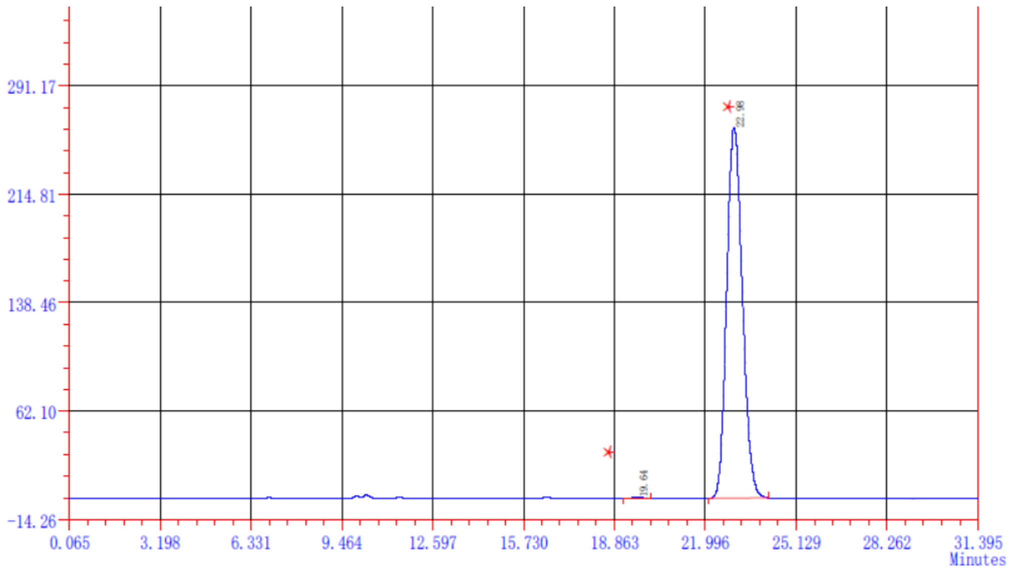

ID	组分名	保留时间	峰高	峰面积	浓度	拖尾因子	理论塔板
4	组份 4	19.642	959	27438.5	0.2774	1.05	9393
2		22.983	261012	9865382.0	99.7226	1.14	7370
	Σ:		261971	9892820.5	100.0000		

Translation: Chiralcel IC-H column [λ = 254 nm; eluent: Hexane/Isopropanol = 70/30; Flow rate: 0.5 mL/min; t_{minor} = 19.64 min, t_{major} = 22.98 min; ee% > 99%].

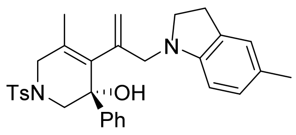

(S)-5-methyl-4-(3-(5-methylindolin-1-yl)prop-1-en-2-yl)-3-phenyl-1-tosyl-1,2,3,6-tetrahydropyridin-3-ol (3ac)

A light yellow oil. 67% yield (33 mg). ^{1}H NMR (500 MHz, $CDCl_3$, TMS) δ 1.88 (s, 3H), 2.23 (s, 3H), 2.40 (s, 3H), 2.84-2.87 (m, 3H), 2.90-2.95 (m, 1H), 3.03-3.07 (m, 1H), 3.20 (d, J = 14.5 Hz, 1H), 3.39 (d, J = 14.5 Hz, 1H), 3.46-3.50 (m, 2H), 3.88 (d, J = 16.5 Hz, 1H), 4.72 (s, 1H), 5.12 (s, 1H), 5.30 (brs, 1H), 6.23 (d, J = 7.5 Hz, 1H), 6.81 (d, J = 8.0 Hz, 1H), 6.91 (s, 1H), 7.25-7.32 (m, 5H), 7.45 (d, J = 8.0 Hz, 2H), 7.63 (d, J = 8.0 Hz, 2H). ^{13}C NMR (100 MHz, $CDCl_3$, TMS) δ 18.6, 20.7, 21.5, 28.4, 49.6, 54.5, 57.3, 57.9, 72.5, 109.3, 120.3, 125.3, 126.5, 127.1, 127.4, 127.7, 127.8, 129.1, 129.6, 130.5, 131.0, 133.0, 136.0, 141.6, 143.5, 143.6, 149.1. IR (CH_2Cl_2): ν 3497, 3056, 3031, 2970, 2924, 2851, 2342, 1604, 1472, 1457, 1447, 1348, 1306, 1249, 1168, 1152, 1090, 1051, 977, 909, 848, 815, 757, 706, 668, 661 cm^{-1}. HRMS (ESI) calcd. for $C_{31}H_{35}N_2O_3S$ $(M+H)^+$: 515.2363, Found: 515.2355. Enantiomeric excess was determined by HPLC with a Chiralcel

AD-H column [λ = 254 nm; eluent: Hexane/Isopropanol = 80/20; Flow rate: 0.50 mL/min; t_{minor} = 13.48 min, t_{major} = 14.61 min; ee% > 99%; $[\alpha]^{20}_{D}$ = +46.2 (c 1.00, CH_2Cl_2)].

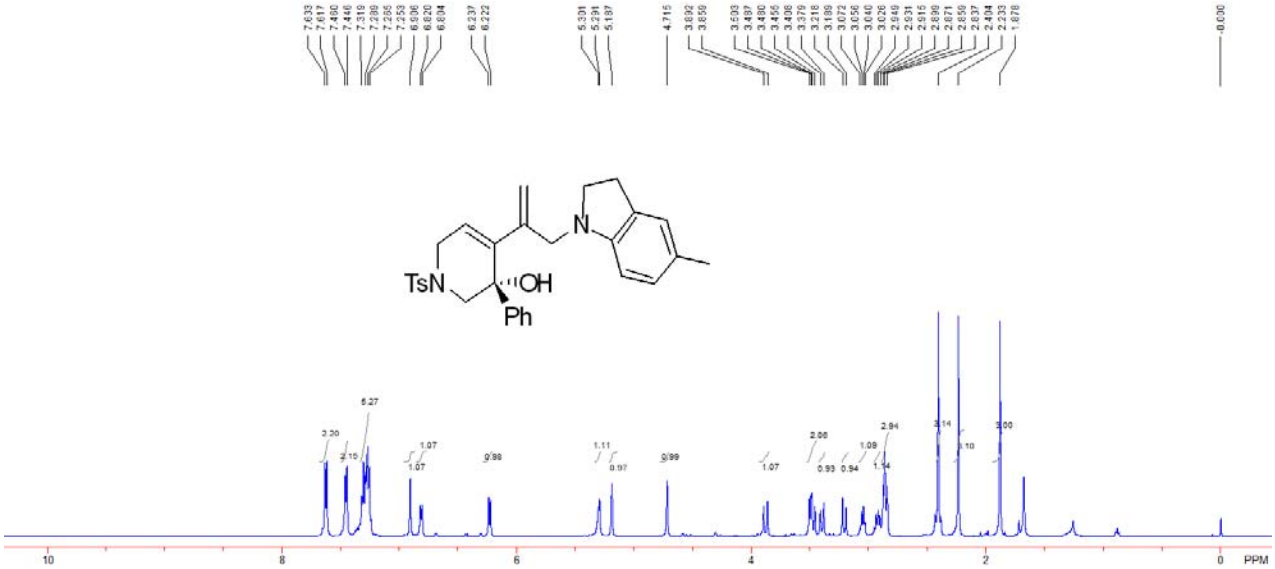

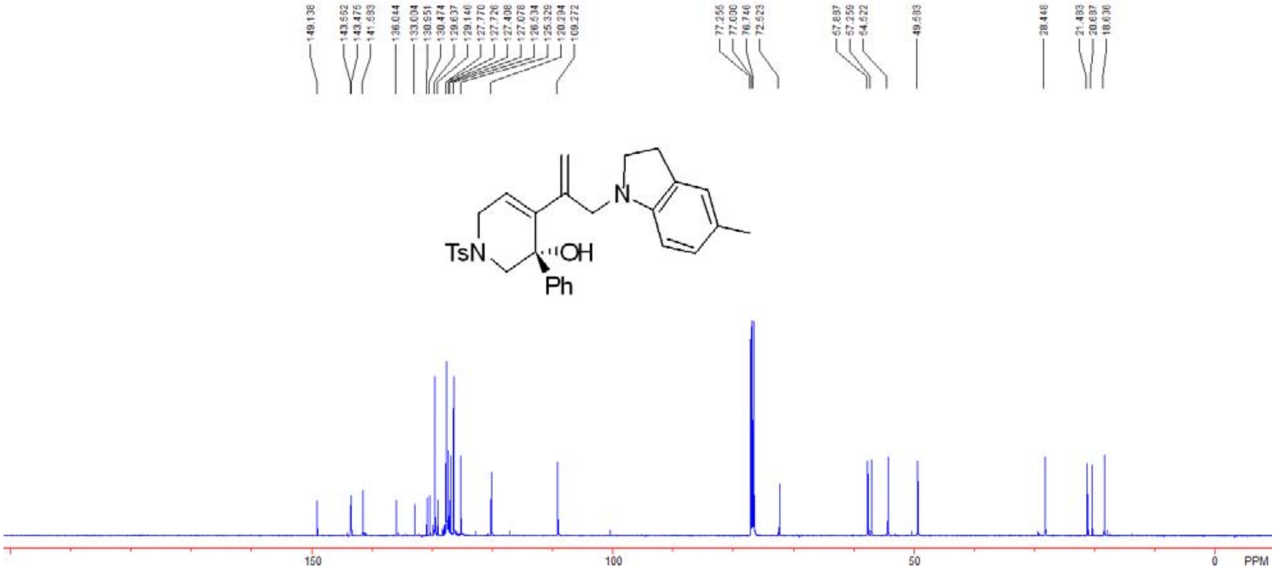

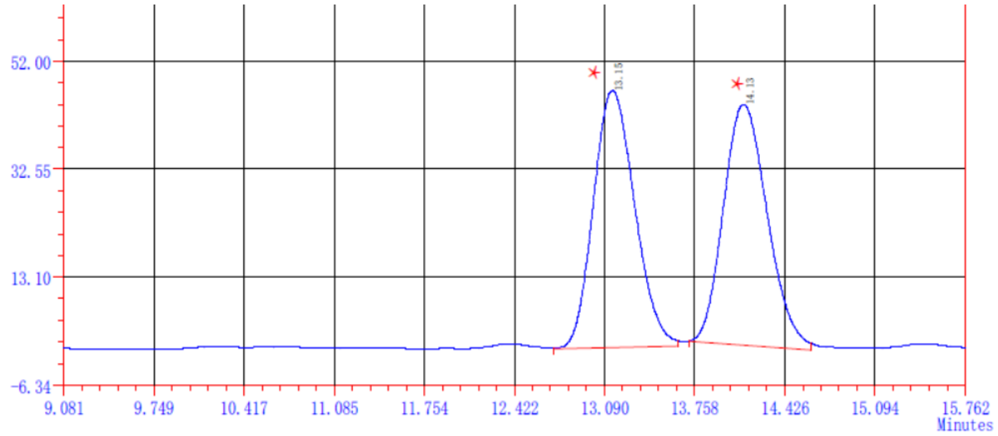

ID	组分名	保留时间	峰高	峰面积	浓度	拖尾因子	理论塔板
1		13.150	46334	987980.2	50.1279	1.18	7580
1	组份 1	14.125	43455	982940.3	49.8721	1.10	7772
	Σ:		89789	1970920.5	100.0000		

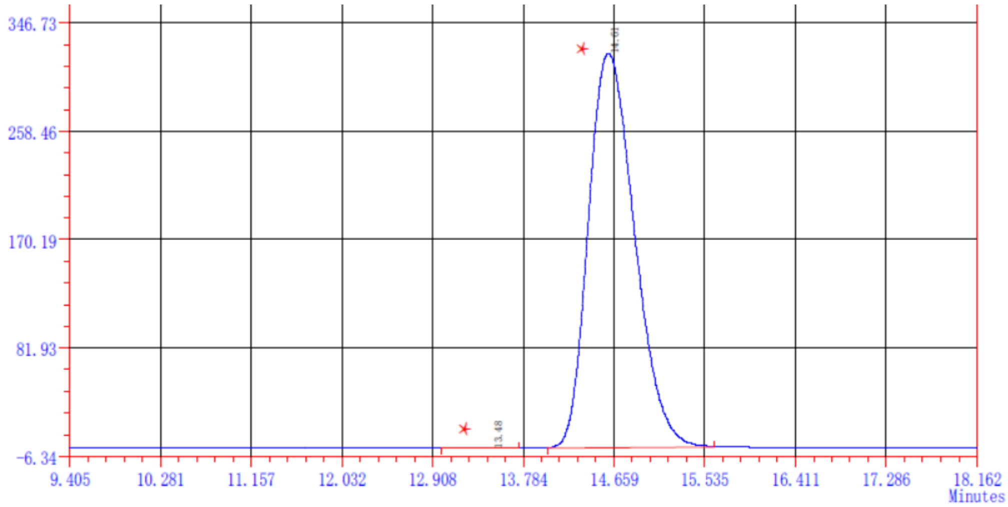

ID	组分名	保留时间	峰高	峰面积	浓度	拖尾因子	理论塔板
1		13.483	52	955.1	0.0096	1.14	10740
1	组份 1	14.607	321402	9944585.9	99.9904	1.26	4442
	Σ:		321454	9945541.0	100.0000		

Translation: Chiralcel AD-H column [λ = 254 nm; eluent: Hexane/Isopropanol = 80/20; Flow rate: 0.5 mL/min; t_{minor} = 13.48 min, t_{major} = 14.61 min; ee% > 99%].

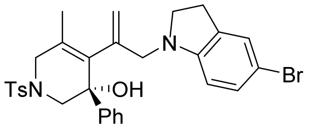

(S)-4-(3-(5-bromoindolin-1-yl)prop-1-en-2-yl)-5-methyl-3-phenyl-1-tosyl-1,2,3,6-tetrahydropyridin-3-ol (3ad)

A white solid. 73% yield (42 mg). M. P. 191-193 °C ^{1}H NMR (400 MHz, $CDCl_3$, TMS) δ 1.85 (s,

3H), 2.42 (s, 3H), 2.81 (d, J = 11.6 Hz, 1H), 2.86-2.91 (m, 2H), 3.06 (dd, J_1 = 8.8 Hz, J_2 = 16.8 Hz, 1H), 3.14 (dd, J_1 = 8.0 Hz, J_2 = 16.8 Hz, 1H), 3.26 (d, J = 15.2 Hz, 1H), 3.39 (d, J = 16.8 Hz, 2H), 3.55 (d, J = 11.6 Hz, 1H), 3.92 (d, J = 16.8 Hz, 1H), 4.17 (s, 1H), 4.72 (s, 1H), 5.14 (d, J = 0.8 Hz, 1H), 5.91 (d, J = 8.4 Hz, 1H), 7.02 (dd, J_1 = 2.0 Hz, J_2 = 8.4 Hz, 1H), 7.12 (s, 1H), 7.27-7.33 (m, 5H), 7.43 (d, J = 8.4 Hz, 2H), 7.63 (d, J = 8.4 Hz, 2H). ^{13}C NMR (100 MHz, $CDCl_3$, TMS) δ 18.5, 21.5, 28.2, 49.6, 53.9, 55.9, 57.5, 72.6, 109.4, 110.2, 119.2, 126.5, 127.24, 127.77, 127.82, 128.1, 129.7, 129.9, 130.6, 132.4, 132.7, 136.0, 140.9, 142.4, 143.8, 150.8. IR (CH_2Cl_2): ν 3498, 2964, 2920, 2849, 2360, 2342, 1647, 1598, 1488, 1466, 1448, 1348, 1329, 1305, 1288, 1251, 1171, 1153, 1091, 1054, 1038, 1018, 986, 945, 904, 882, 862, 809, 766, 706 cm^{-1}. HRMS (ESI) calcd. for $C_{30}H_{32}BrN_2O_3S$ $(M+H)^+$: 579.1312, Found: 579.1304. Enantiomeric excess was determined by HPLC with a Chiralcel IC-H column [λ = 254 nm; eluent: Hexane/Isopropanol = 70/30; Flow rate: 0.50 mL/min; t_{minor} = 30.33 min, t_{major} = 27.26 min; ee% > 99%; $[\alpha]^{20}_D$ = +32.0 (c 1.00, CH_2Cl_2)].

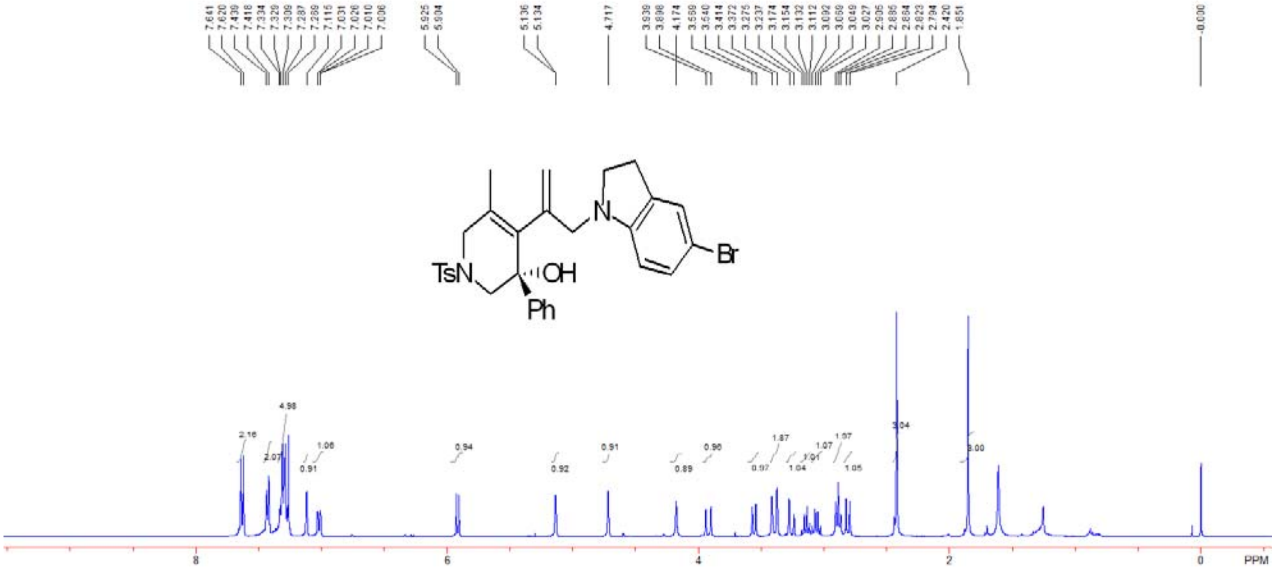

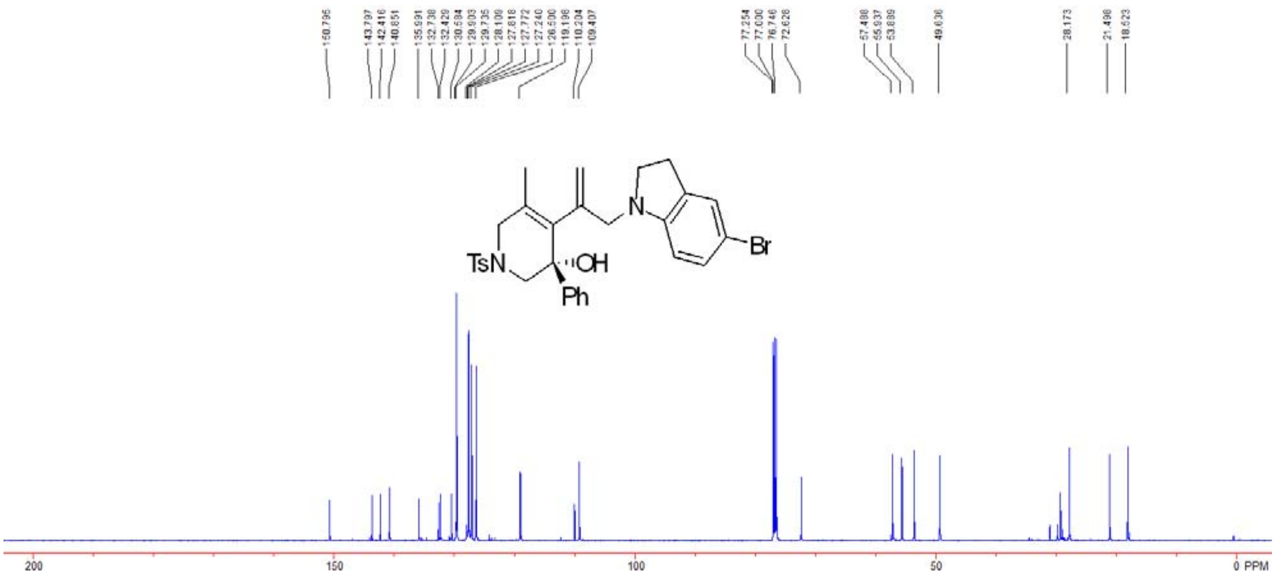

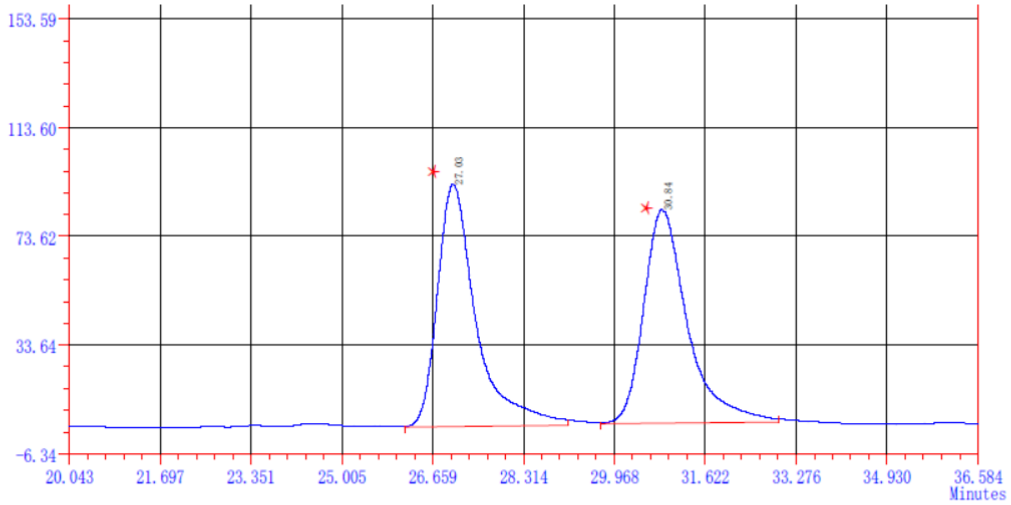

ID	组分名	保留时间	峰高	峰面积	浓度	拖尾因子	理论塔板
1		27.027	89297	4396854.0	50.1070	1.84	6005
2		30.835	78565	4378069.8	49.8930	1.56	6102
	Σ:		167862	8774923.9	100.0000		

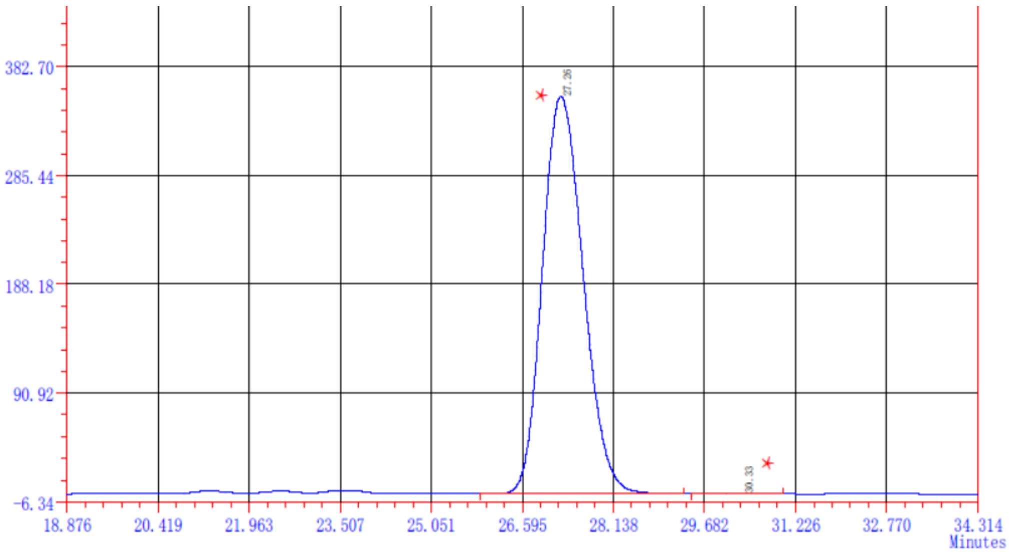

ID	组分名	保留时间	峰高	峰面积	浓度	拖尾因子	理论塔板
1		27.255	355810	17110879.2	99.8320	1.16	6402
2		30.332	580	28801.1	0.1680	0.88	7436
	Σ:		356390	17139680.3	100.0000		

Translation: Chiralcel IC-H column [λ = 254 nm; eluent: Hexane/Isopropanol = 70/30; Flow rate: 0.5 mL/min; t_{minor} = 30.33 min, t_{major} = 27.26 min; ee% > 99%].

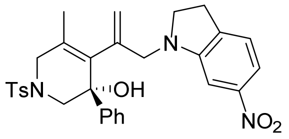

(S)-5-methyl-4-(3-(6-nitroindolin-1-yl)prop-1-en-2-yl)-3-phenyl-1-tosyl-1,2,3,6-tetrahydropyridin-3-ol (3ae)

A white solid. 87% yield (47 mg). M. P. 181-183 °C. ^{1}H NMR (400 MHz, $CDCl_3$, TMS) δ 1.85 (s, 3H), 2.43 (s, 3H), 2.85 (d, J = 11.6 Hz, 1H), 2.98 (dd, J_1 = 8.4 Hz, J_2 = 8.4 Hz, 2H), .3.26 (dd, J_1 = 8.4 Hz, J_2 = 8.4 Hz, 2H), 3.37 (d, J = 16.0 Hz, 1H), 3.43 (s, 2H), 3.51 (s, 1H), 3.59 (d, J = 11.6 Hz, 1H), 3.94 (d, J = 16.0 Hz, 1H), 4.73 (d, J = 1.2 Hz, 1H), 5.11 (d, J = 1.2 Hz, 1H), 6.85 (d, J = 1.6 Hz, 1H), 7.05 (d, J = 8.0 Hz, 1H), 7.28-7.36 (m, 5H), 7.44 (d, J = 8.0 Hz, 2H), 7.48 (dd, J_1 = 2.0 Hz, J_2 = 8.0 Hz, 1H), 7.64 (d, J = 8.0 Hz, 2H). ^{13}C NMR (100 MHz, $CDCl_3$, TMS) δ 18.4, 21.5, 28.2, 49.7, 53.4, 54.3, 57.5, 72.7, 100.9, 113.6, 118.3, 123.8, 126.3, 127.5, 127.8, 127.9, 129.8, 130.5, 132.5, 135.9, 137.4, 140.2, 141.6, 144.0, 148.5, 152.7. IR (CH_2Cl_2): ν 2969, 2920, 2844, 2360, 2342, 1604, 1558, 1487, 1456, 1399, 1249, 1156, 1090, 1045, 988, 911, 857, 848, 811, 748, 705 cm^{-1}. HRMS (ESI) calcd. for $C_{30}H_{32}N_3O_5S$ $(M+H)^+$: 546.2057, Found: 546.2048.

Enantiomeric excess was determined by HPLC with a Chiralcel AD-H column [λ = 254 nm; eluent: Hexane/Isopropanol = 70/30; Flow rate: 0.50 mL/min; t_{minor} = 52.87 min, t_{major} = 25.6 min; ee% > 99%; $[\alpha]^{20}_{D}$ = +43.52 (c 1.00, CH_2Cl_2)].

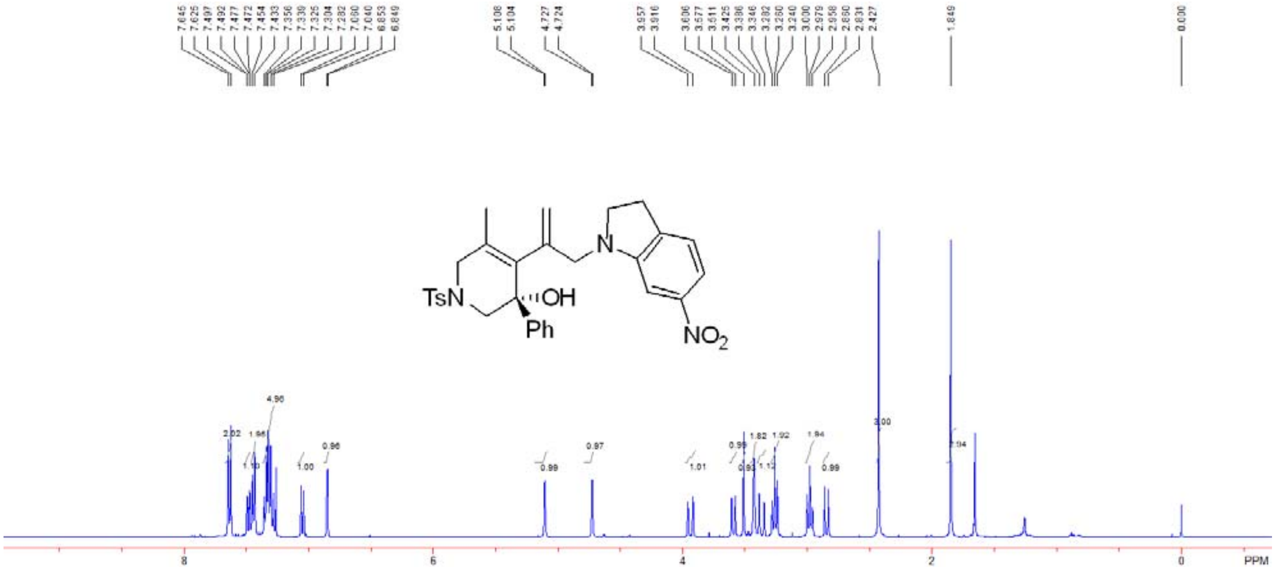

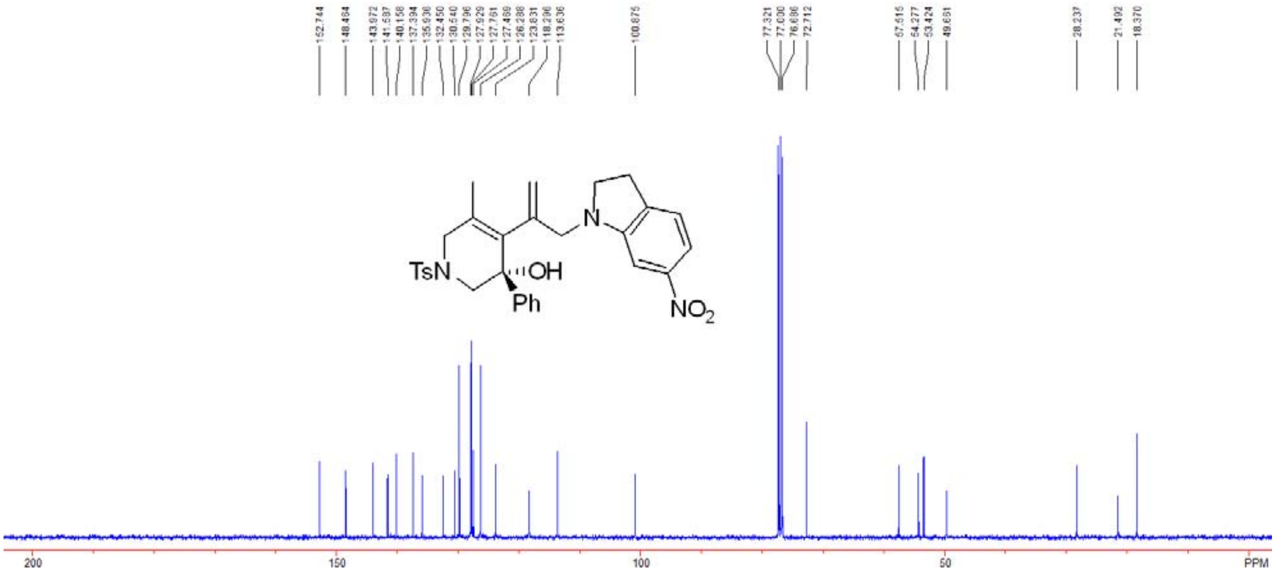

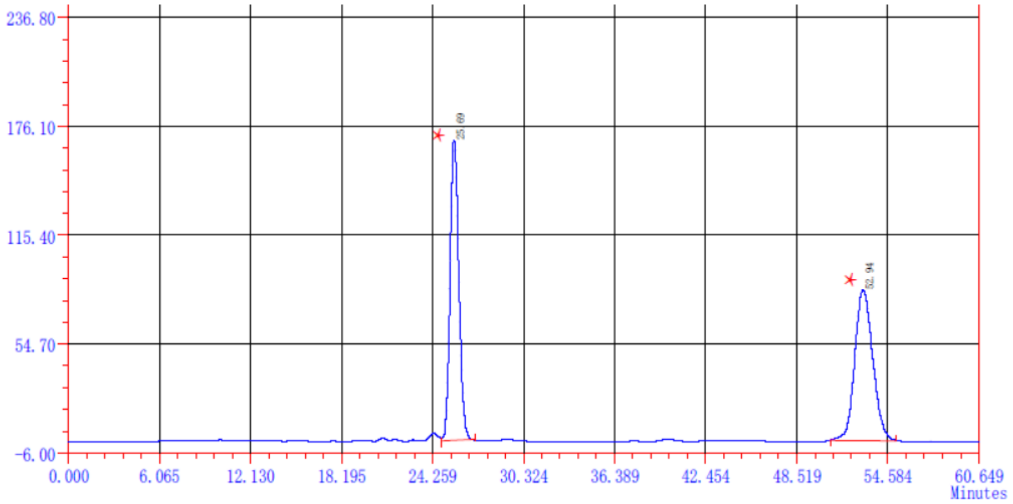

ID	组分名	保留时间	峰高	峰面积	浓度	拖尾因子	理论塔板
1		25. 688	167511	6814806. 1	49. 2332	1. 17	7947
2		52. 943	84146	7027074. 8	50. 7668	1. 11	8011
	Σ:		251657	13841880. 9	100. 0000		

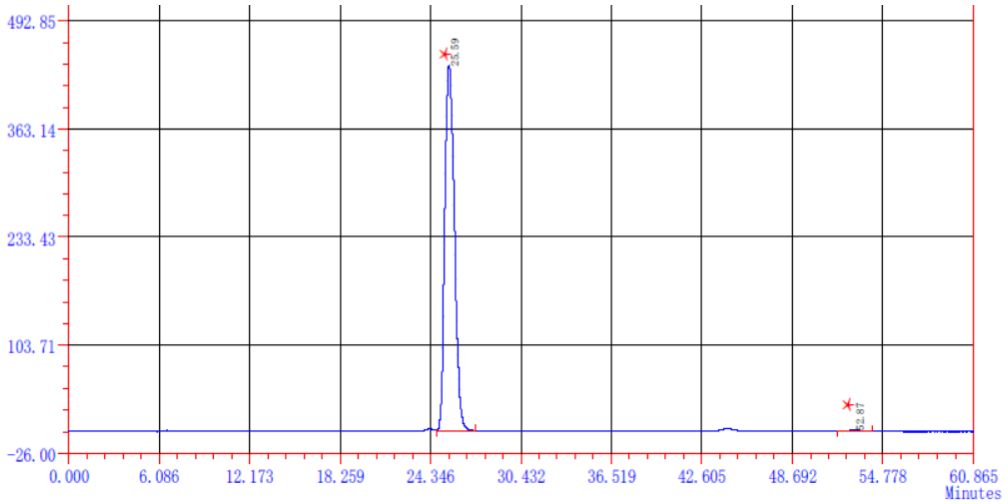

ID	组分名	保留时间	峰高	峰面积	浓度	拖尾因子	理论塔板
1		25. 585	437850	19801217. 6	99. 5097	1. 24	6379
2		52. 865	1273	97558. 5	0. 4903	0. 00	9484
	Σ:		439123	19898776. 1	100. 0000		

Translation: Chiralcel AD-H column [λ = 254 nm; eluent: Hexane/Isopropanol = 70/30; Flow rate: 0.5 mL/min; t_{minor} = 52.87 min, t_{major} = 25.59 min; ee% > 99%].

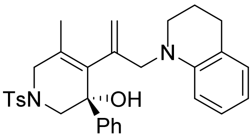

(S)-4-(3-(3,4-dihydroquinolin-1(2H)-yl)prop-1-en-2-yl)-5-methyl-3-phenyl-1-tosyl-1,2,3,6-tetr

ahydropyridin-3-ol (3af)

A light yellow oil. 91% yield (47 mg). ^{1}H NMR (400 MHz, $CDCl_3$, TMS) δ 1.78-1.85 (m, 5H), 2.43 (s, 3H), 2.67 (t, *J* = 6.4 Hz, 2H), 2.90-2.96 (m, 2H), 2.99-3.05 (m, 1H), 3.22 (s, 1H), 3.32 (d, *J* = 18.0 Hz, 1H), 3.39 (d, *J* = 16.4 Hz, 1H), 3.56 (d, *J* = 11.6 Hz, 1H), 3.67 (d, *J* = 18.0 Hz, 1H), 3.90 (d, *J* = 16.0 Hz, 1H), 4.64 (d, *J* = 2.0 Hz, 1H), 4.97 (d, *J* = 2.0 Hz, 1H), 5.61 (d, *J* = 8.4 Hz, 1H), 6.46 (dd, J_1 = 7.2 Hz, J_1 = 7.2 Hz, 1H), 6.73-6.77 (m, 1H), 6.84 (d, *J* = 7.2 Hz, 1H), 7.29-7.35 (m, 5H), 7.46 (d, *J* = 8.0 Hz, 2H), 7.65 (d, *J* = 8.0 Hz, 2H). ^{13}C NMR (100 MHz, $CDCl_3$, TMS) δ 18.2, 21.5, 22.1, 28.0, 49.1, 49.6, 56.8, 57.5, 72.7, 110.9, 115.5, 116.0, 121.8, 126.5, 126.9, 127.4, 128.82, 128.84, 128.51, 129.4, 129.8, 132.5, 136.6, 139.7, 141.5, 144.0, 145.0. IR (CH_2Cl_2): ν 3483, 3062, 3023, 2922, 2850, 2360, 2341, 1647, 1599, 1574, 1506, 1493, 1447, 1342, 1307, 1249, 1210, 1182, 1157, 1090, 1045, 1029, 989, 968, 863, 804, 790, 741, 701 cm^{-1}. HRMS (ESI) calcd. for $C_{31}H_{35}N_2O_3S$ $(M+H)^+$: 515.2363, Found: 515.2354. Enantiomeric excess was determined by HPLC with a Chiralcel AD-H column [λ = 254 nm; eluent: Hexane/Isopropanol = 80/20; Flow rate: 0.50 mL/min; t_{minor} = 29.49 min, t_{major} = 24.90 min; ee% > 99%; $[\alpha]^{20}_D$ = +34.9 (c 1.00, CH_2Cl_2)].

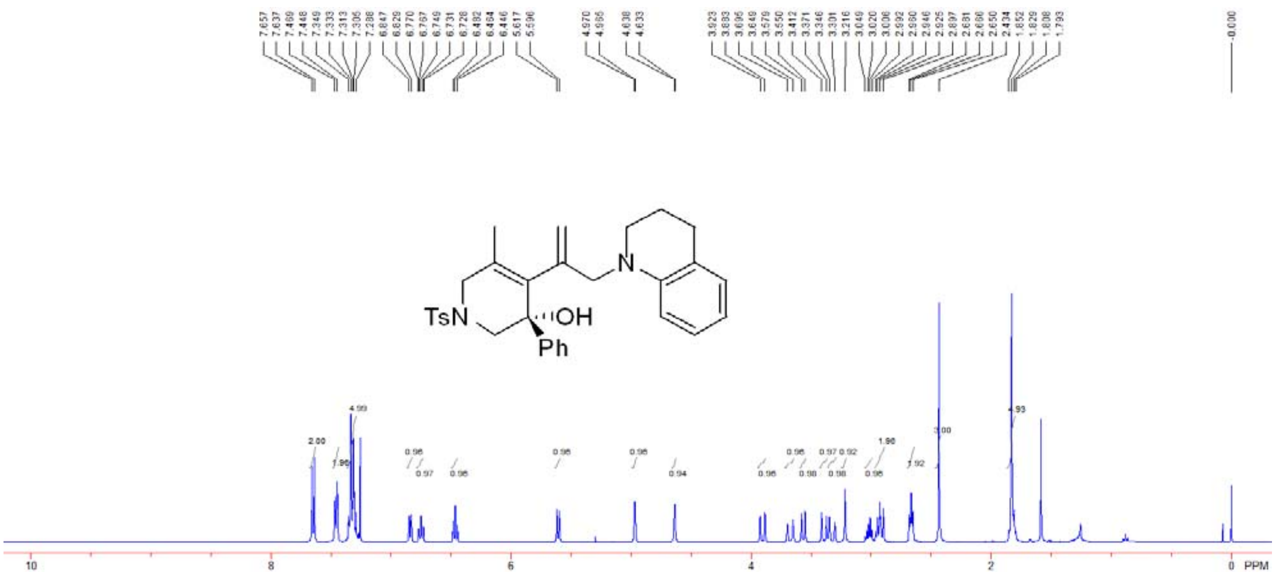

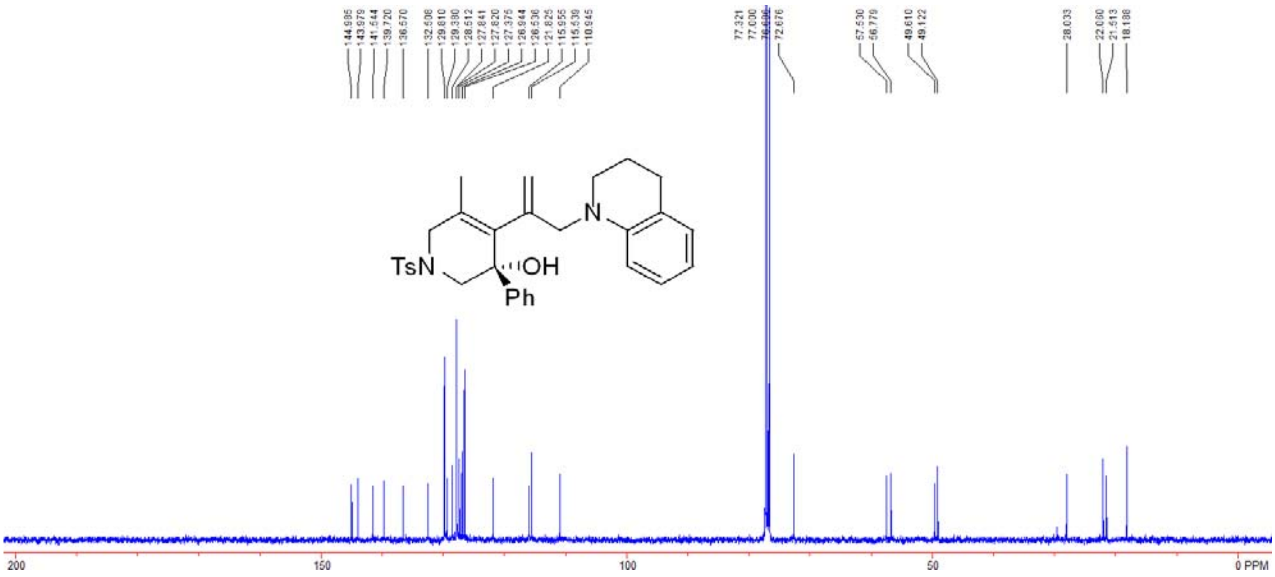

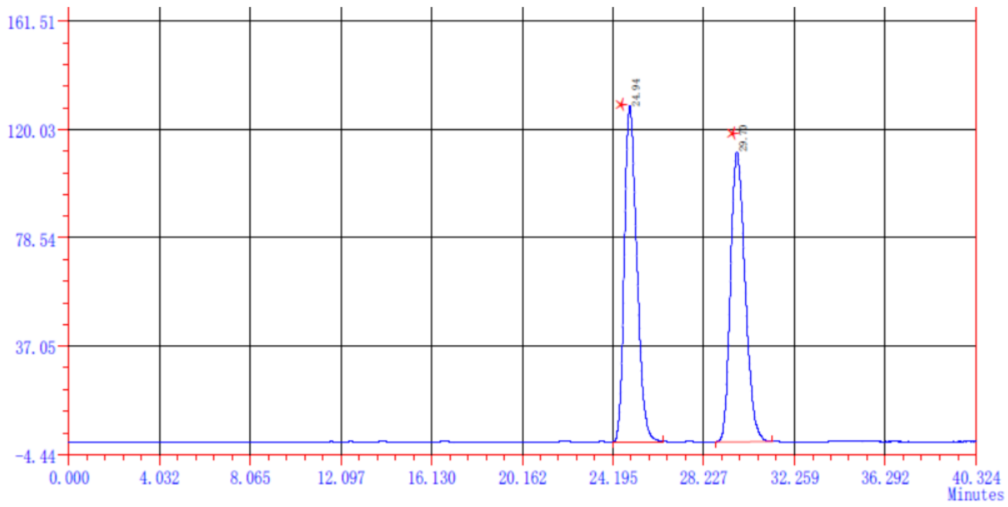

ID	组分名	保留时间	峰高	峰面积	浓度	拖尾因子	理论塔板
1		24.937	128550	4973985.0	49.6943	1.27	8278
2		29.703	111175	5035186.3	50.3057	1.23	8573
	Σ:		239725	10009171.3	100.0000		

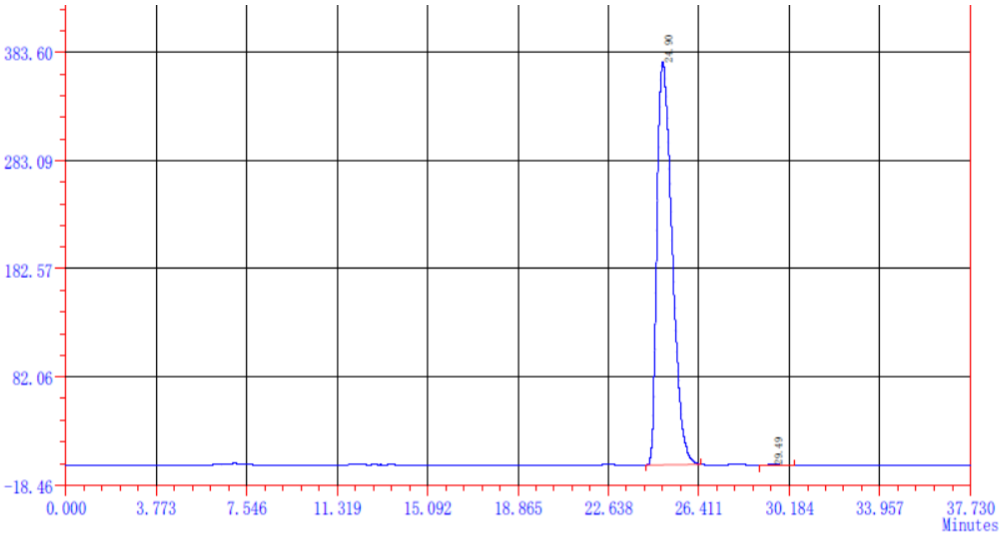

ID	组分名	保留时间	峰高	峰面积	浓度	拖尾因子	理论塔板
1		24.900	374531	16455715.0	99.7579	1.54	6401
2		29.493	798	39942.5	0.2421	1.30	6920
	Σ:		375329	16495657.5	100.0000		

Translation: Chiralcel AD-H column [λ = 254 nm; eluent: Hexane/Isopropanol = 80/20; Flow rate: 0.5 mL/min; t_{minor} = 29.49 min, t_{major} = 24.90 min; ee% > 99%].

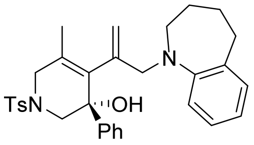

(S)-5-methyl-3-phenyl-4-(3-(2,3,4,5-tetrahydro-1H-benzo[b]azepin-1-yl)prop-1-en-2-yl)-1-tosyl-1,2,3,6-tetrahydropyridin-3-ol (3ag)

A light yellow oil. 67% yield (35 mg). ^{1}H NMR (400 MHz, $CDCl_3$, TMS) δ 1.52 (s, 3H), 1.55-1.62 (m, 3H), 1.75-1.81 (m, 1H), 2.41 (s, 3H), 2.67-2.79 (m, 4H), 2.85-2.91 (m, 1H), 3.38 (d, J = 16.0 Hz, 2H), 3.58-3.62 (m, 2H), 3.80 (d, J = 16.0 Hz, 1H), 4.74 (s, 1H), 5.20 (s, 1H), 5.93 (brs, 1H), 6.74 (d, J = 7.6 Hz, 1H), 6.91 (ddd, J_1 = 1.2 Hz, J_2 = 7.6 Hz, J_3 = 8.4 Hz, 1H), 7.03-7.11 (m, 2H), 7.20-7.29 (m, 5H), 7.37 (dd, J_1 = 1.2 Hz, J_2 = 8.4 Hz,, 2H), 7.66 (d, J = 8.4 Hz, 2H). ^{13}C NMR (100 MHz, $CDCl_3$, TMS) δ 18.0, 21.5, 25.3, 29.1, 34.3, 49.3, 54.1, 57.3, 60.1, 72.6, 118.4, 120.1, 122.9, 126.5, 126.6, 126.9, 127.6, 127.8, 129.6, 130.1, 131.6, 133.7, 135.1, 137.0, 142.4, 143.4, 143.5, 150.2. IR (CH_2Cl_2): ν 3064, 2924, 2853, 2360, 2342, 1670, 1597, 1541, 1493, 1449, 1348, 1251, 1167, 1091, 1055, 990, 918, 865, 814, 791, 764, 749, 704 cm^{-1}. HRMS (ESI) calcd. for $C_{32}H_{37}N_2O_3S$ $(M+H)^+$: 529.2519, Found: 529.2511. Enantiomeric excess was determined by HPLC with a Chiralcel AD-H column [λ = 254 nm; eluent: Hexane/Isopropanol = 90/10; Flow

rate: 0.50 mL/min; t_{minor} = 24.05 min, t_{major} = 22.00 min; ee% > 99%; $[\alpha]^{20}_{D}$ = +3.7 (c 1.00, CH_2Cl_2)].

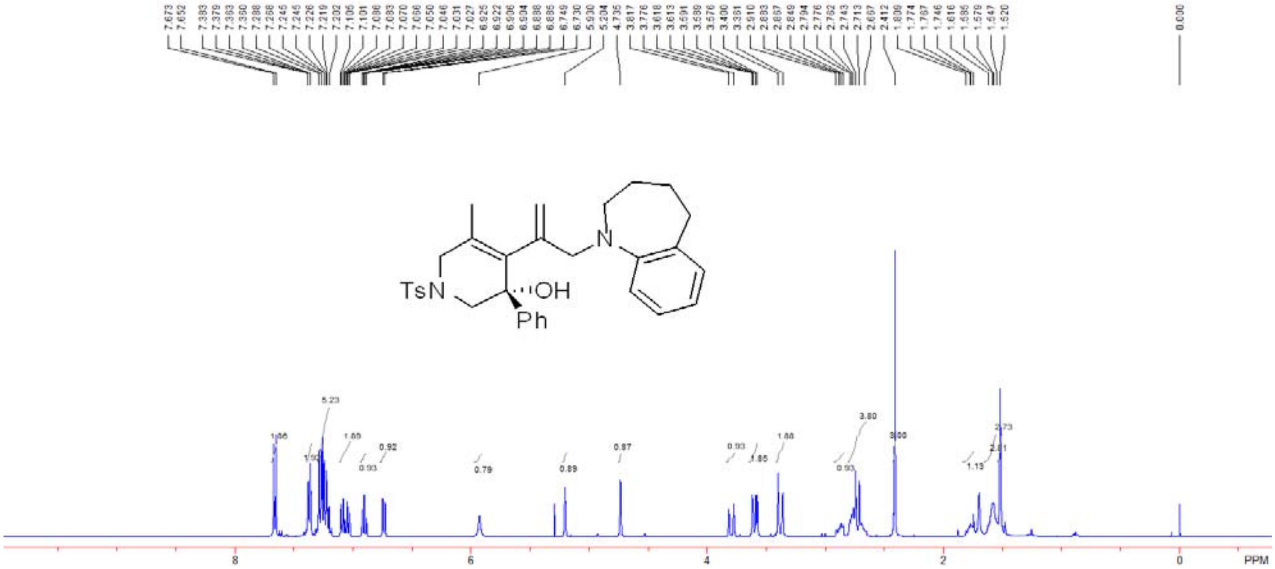

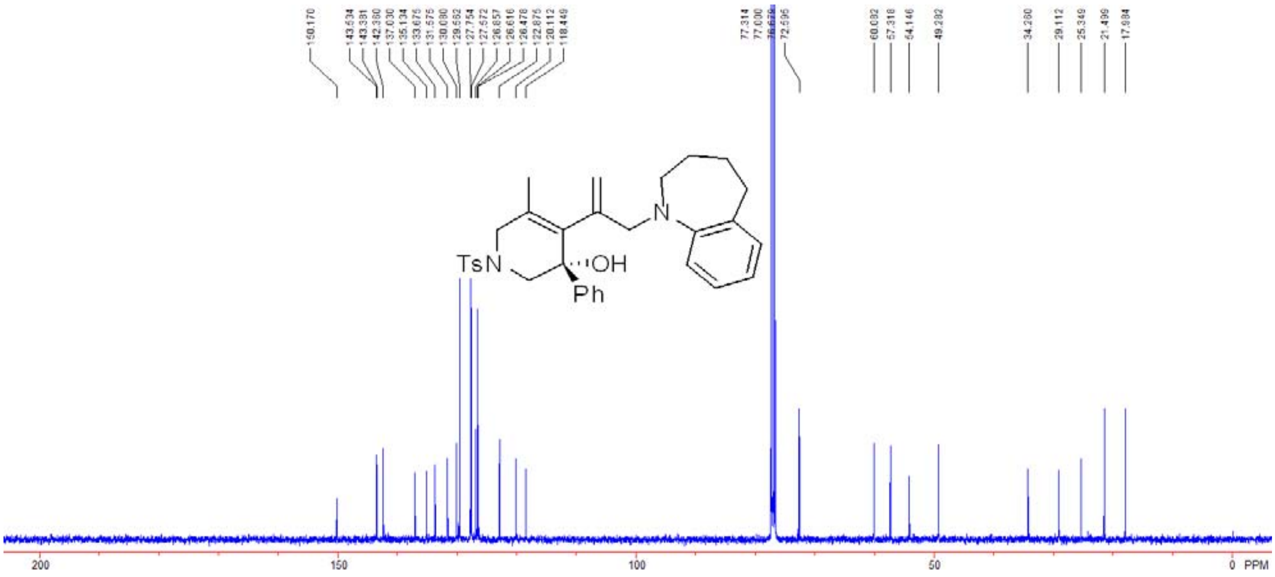

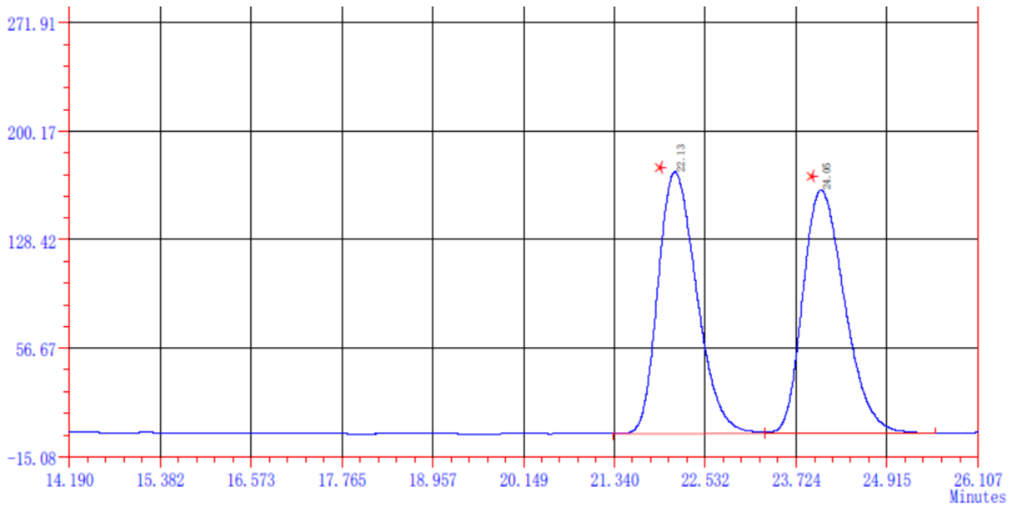

ID	组分名	保留时间	峰高	峰面积	浓度	拖尾因子	理论塔板
1		22.133	172987	6111698.1	49.7283	1.27	7822
2		24.048	161225	6178478.7	50.2717	1.28	7849
	Σ:		334212	12290176.8	100.0000		

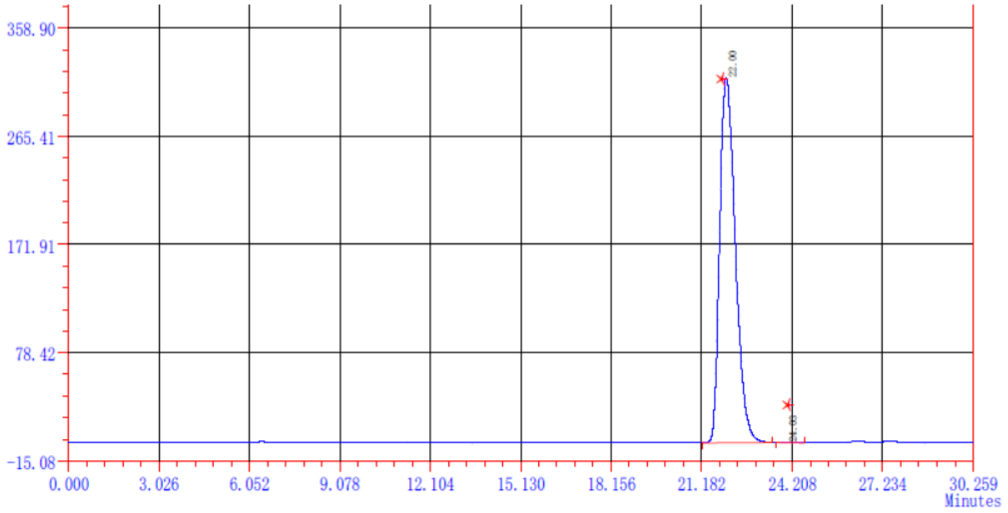

ID	组分名	保留时间	峰高	峰面积	浓度	拖尾因子	理论塔板
1		22.003	315572	11532191.8	99.8938	1.36	7226
2		24.025	401	12260.4	0.1062	0.99	12306
	Σ:		315973	11544452.2	100.0000		

Translation: Chiralcel AD-H column [λ = 254 nm; eluent: Hexane/Isopropanol = 90/10; Flow rate: 0.5 mL/min; t_{minor} = 24.03 min, t_{major} = 22.00 min; ee% > 99%].

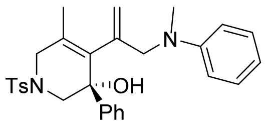

(S)-5-methyl-4-(3-(methyl(phenyl)amino)prop-1-en-2-yl)-3-phenyl-1-tosyl-1,2,3,6-tetrahydropyridin-3-ol (3ah)

A white solid. 96% yield (47 mg). M. P. 138-140 °C ^{1}H NMR (400 MHz, $CDCl_3$, TMS) δ 1.80 (s, 3H), 2.43 (s, 3H), 2.67 (s, 3H), 2.89 (d, J = 8.0 Hz, 1H), 3.39 (d, J = 16.8 Hz, 2H), 3.51 (brs, 1H), 3.57 (d, J = 11.6 Hz, 1H), 3.71 (d, J = 18.4 Hz, 1H), 3.90 (d, J = 16.0 Hz, 1H), 4.65 (d, J = 1.6 Hz, 1H), 4.90 (d, J = 1.6 Hz, 1H), 6.26 (d, J = 8.0 Hz, 2H), 6.63 (dd, J_1 = 7.2 Hz, J_2 = 7.6 Hz, 1H), 7.06 (dd, J_1 = 7.2 Hz, J_2 = 8.8 Hz, 2H), 7.29-7.35 (m, 5H), 7.46 (d, J = 8.0 Hz, 2H), 7.65 (d, J = 8.0 Hz, 2H). ^{13}C NMR (100 MHz, $CDCl_3$, TMS) δ 18.2, 21.5, 25.3, 38.3, 49.6, 57.5, 58.3, 72.6, 112.5, 116.67, 116.74, 126.6, 127.3, 127.8, 128.8, 129.6, 129.8, 132.5, 136.4, 140.4, 141.7, 143.9, 149.3. IR (CH_2Cl_2): ν 3482 3059, 2023, 2992, 2922, 2851, 2360, 2342, 1597, 1573, 1505, 1448, 1428, 1373, 1340, 1258, 1245, 1215, 1191, 1175, 1154, 1118, 1091, 1052, 949, 916, 862, 805, 789, 774, 762, 750, 703 cm^{-1}. HRMS (ESI) calcd. for $C_{29}H_{33}N_2O_3S$ $(M+H)^+$: 489.2206, Found: 489.2200. Enantiomeric excess was determined by HPLC with a Chiralcel AD-H column [λ = 254 nm; eluent: Hexane/Isopropanol = 80/20; Flow rate: 0.50 mL/min; t_{minor} = 32.55 min, t_{major} = 28.41 min; ee% > 99%; $[\alpha]^{20}_D$ = +29.8 (c 1.00, CH_2Cl_2)].

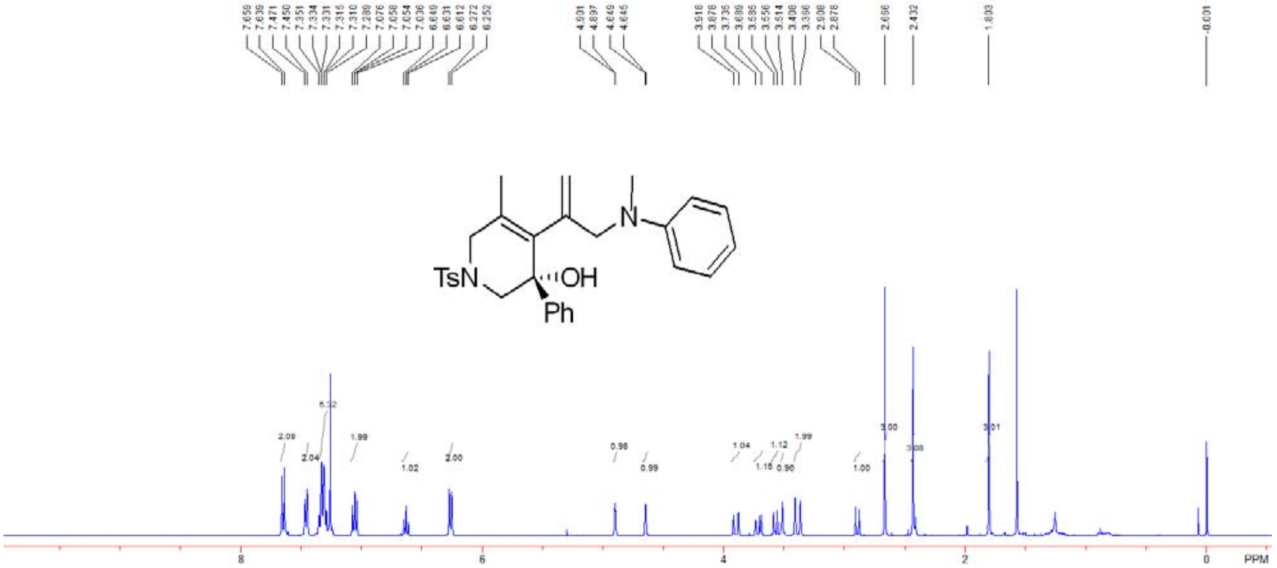

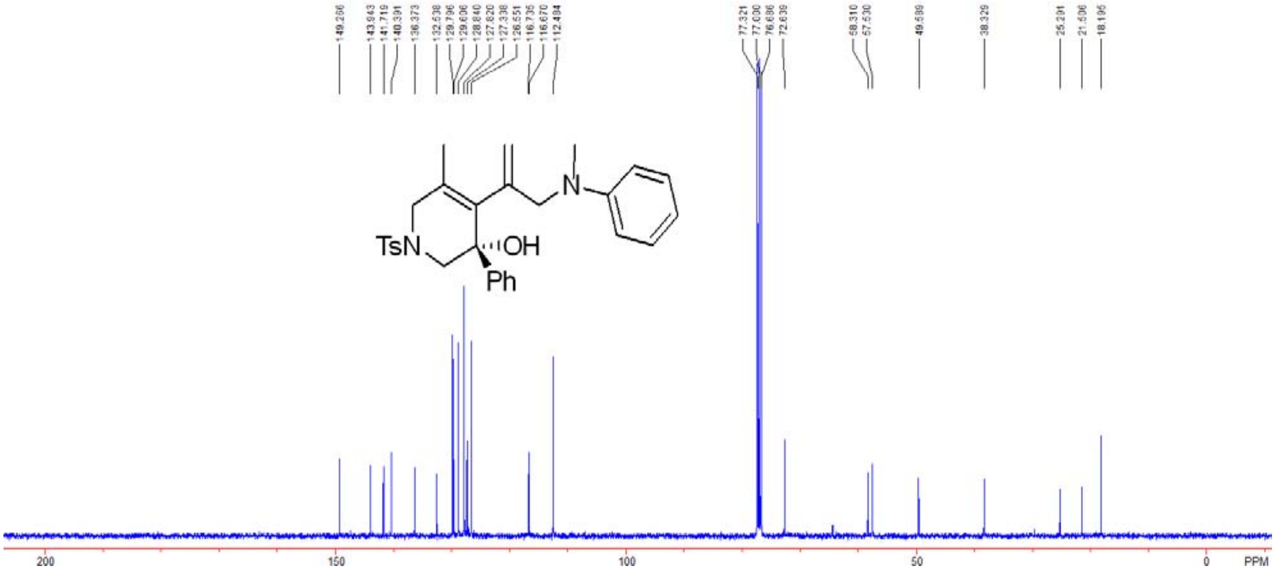

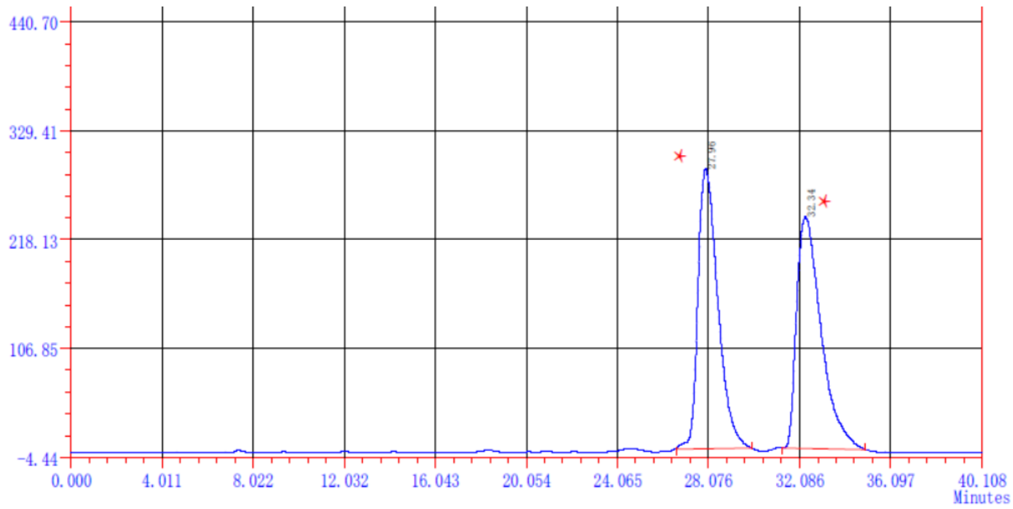

ID	组分名	保留时间	峰高	峰面积	浓度	拖尾因子	理论塔板
1		27.957	286458	17488648.6	49.5164	1.52	4179
2		32.340	236911	17830264.6	50.4836	1.82	3680
	Σ:		523369	35318913.2	100.0000		

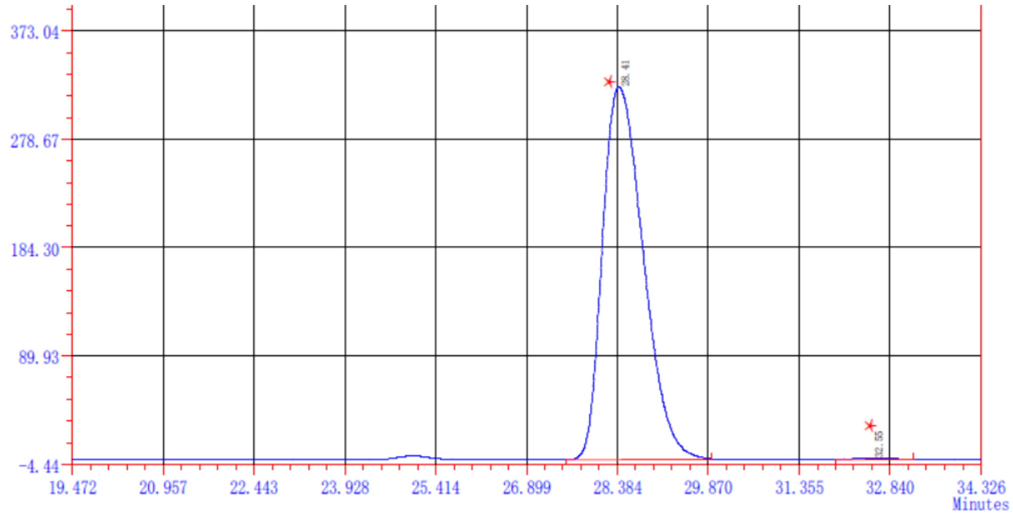

ID	组分名	保留时间	峰高	峰面积	浓度	拖尾因子	理论塔板
1		28.410	324910	15038891.1	99.5206	1.38	7509
2		32.553	1600	72448.2	0.4794	1.10	10301
	Σ:		326510	15111339.3	100.0000		

Translation: Chiralcel AD-H column [λ = 254 nm; eluent: Hexane/Isopropanol = 80/20; Flow rate: 0.5 mL/min; t_{minor} = 32.55 min, t_{major} = 28.41 min; ee% > 99%].

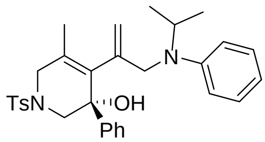

(S)-4-(3-(isopropyl(phenyl)amino)prop-1-en-2-yl)-5-methyl-3-phenyl-1-tosyl-1,2,3,6-tetrahydropyridin-3-ol (3ai)

A light yellow oil. 73% yield (38 mg). ^{1}H NMR (400 MHz, $CDCl_3$, TMS) δ 0.89 (d, J = 6.4 Hz, 3H), 1.01 (d, J = 6.4 Hz, 3H), 1.63 (s, 3H), 2.42 (s, 3H), 2.78 (d, J = 11.6 Hz, 1H), 3.32 (d, J = 18.0 Hz, 1H), 3.35 (d, J = 16.0 Hz, 1H), 3.56 (d, J = 18.0 Hz, 1H), 3.62 (d, J = 11.6 Hz, 1H), 3.73-3.81 (m, 1H), 3.87 (d, J = 16.0 Hz, 1H), 4.17 (brs, 1H), 4.64 (d, J = 2.0 Hz, 1H), 5.08 (d, J = 2.0 Hz, 1H), 6.32 (d, J = 8.0 Hz, 2H), 6.67 (dd, J_1 = 7.2 Hz, J_2 = 7.2 Hz, 1H), 7.02 (dd, J_1 = 7.2 Hz, J_2 = 8.8 Hz, 2H), 7.27-7.32 (m, 5H), 7.45 (d, J = 8.4 Hz, 2H), 7.66 (d, J = 8.4 Hz, 2H). ^{13}C NMR (100 MHz, $CDCl_3$, TMS) δ 18.2, 18.9, 19.5, 21.5, 49.2, 49.5, 51.3, 57.8, 72.6, 116.1, 117.86, 117.91, 126.7, 127.2, 127.8, 127.9, 128.6, 129.7, 130.2, 132.9, 136.6, 142.11, 142.14, 143.8, 148.0. IR (CH_2Cl_2): ν 3026, 2972, 2920, 2849, 2360, 2342, 1604, 1518, 1486, 1456, 1343, 1306, 1249, 1157, 1107, 1090, 1022, 988, 910, 873, 848, 810, 749 cm^{-1}. HRMS (ESI) calcd. for $C_{31}H_{37}N_2O_3S$ $(M+H)^+$: 517.2519, Found: 517.2513. Enantiomeric excess was determined by HPLC with a

Chiralcel AD-H column [λ = 254 nm; eluent: Hexane/Isopropanol = 80/20; Flow rate: 0.50 mL/min; t_{minor} = 20.30 min, t_{major} = 23.02 min; ee% = 97%; $[\alpha]^{20}_{D}$ = +34.8 (c 1.00, CH_2Cl_2)].

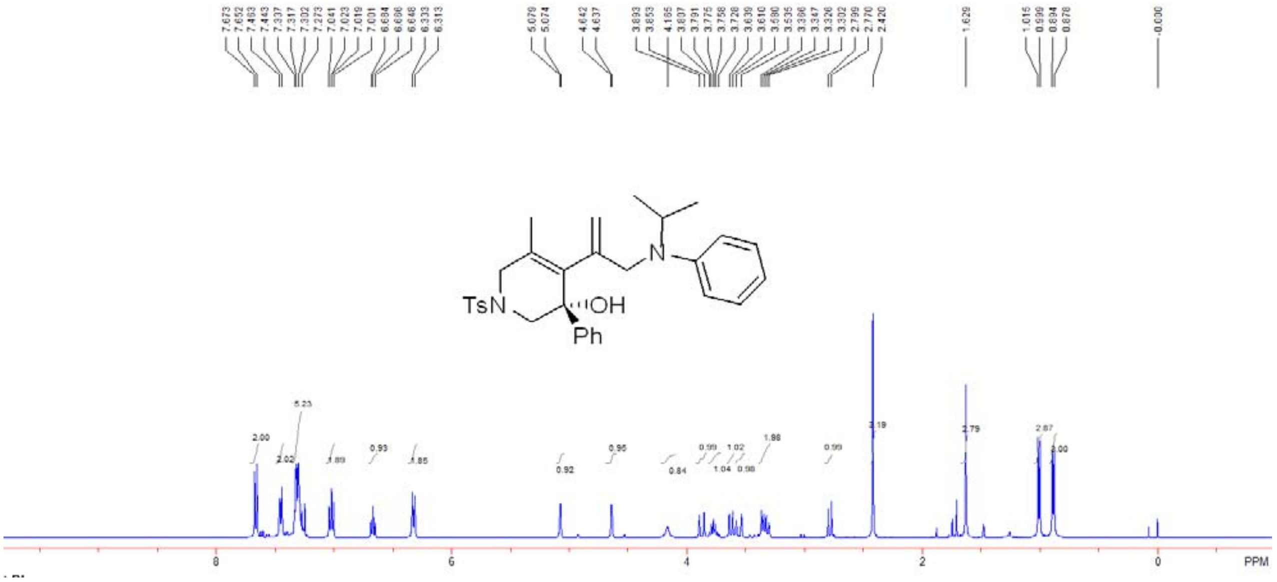

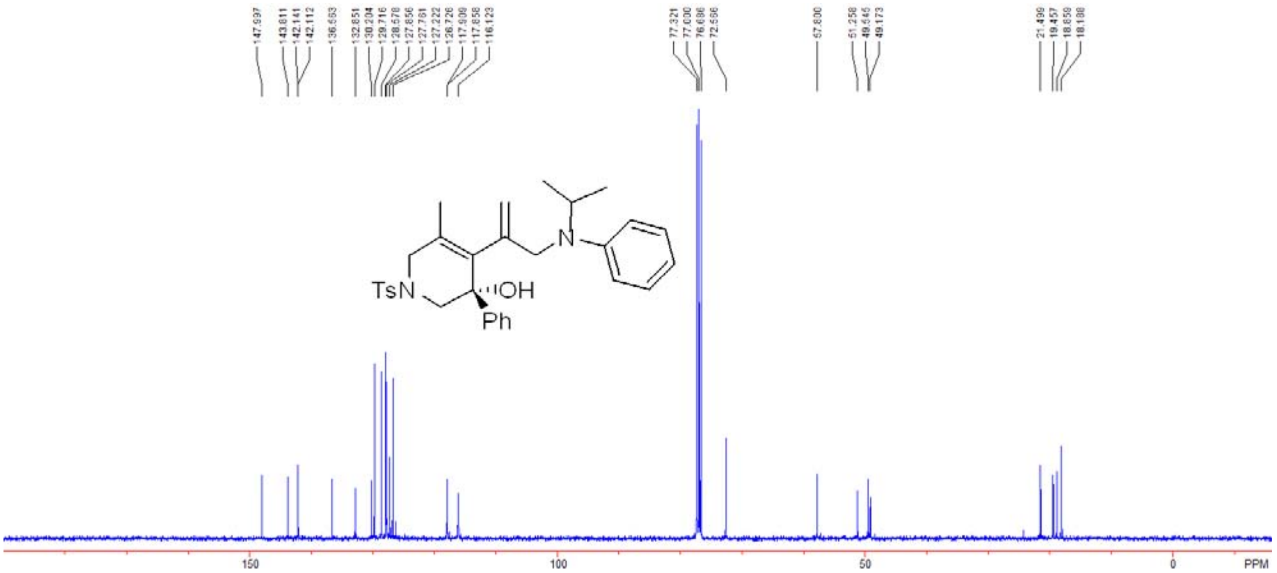

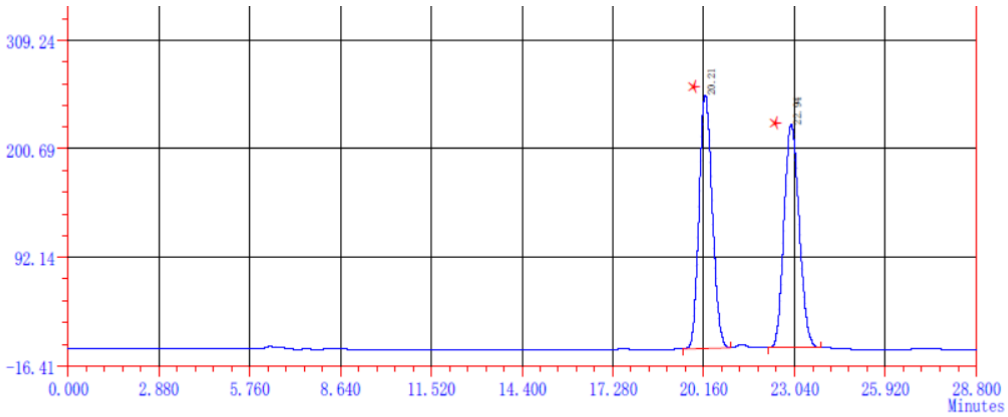

ID	组分名	保留时间	峰高	峰面积	浓度	拖尾因子	理论塔板
4	组份 4	20.210	253582	7425906.5	49.7627	1.12	9493
2		22.935	223575	7496719.8	50.2373	1.12	9325
	Σ:		477157	14922626.3	100.0000		

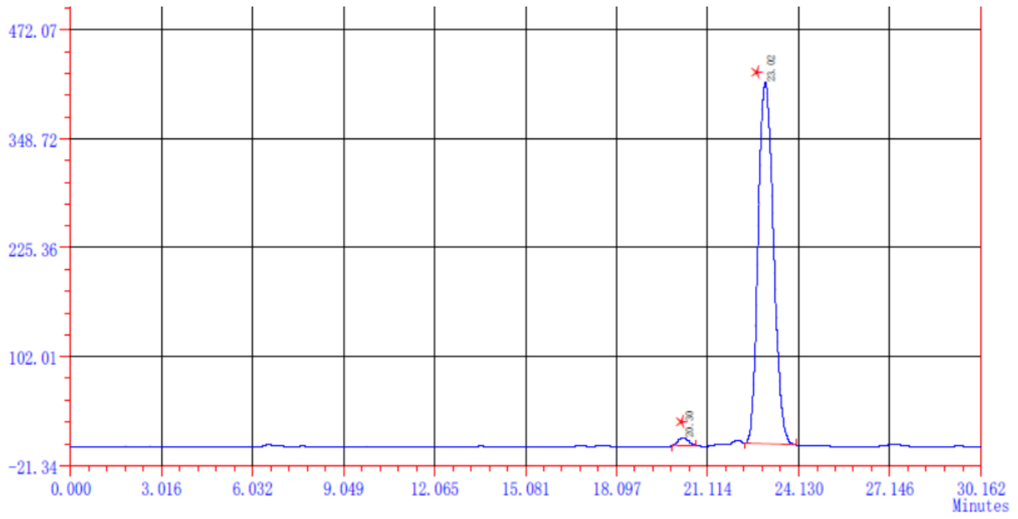

ID	组分名	保留时间	峰高	峰面积	浓度	拖尾因子	理论塔板
4	组份 4	20.295	9036	227519.9	1.5506	1.12	12948
2		23.018	409646	14445243.2	98.4494	1.15	8493
	Σ:		418682	14672763.1	100.0000		

Translation: Chiralcel AD-H column [λ = 254 nm; eluent: Hexane/Isopropanol = 80/20; Flow rate: 0.5 mL/min; t_{minor} = 20.30 min, t_{major} = 23.02 min; ee% = 97%].

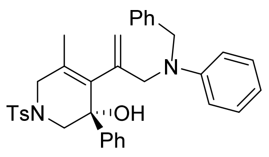

(S)-4-(3-(benzyl(phenyl)amino)prop-1-en-2-yl)-5-methyl-3-phenyl-1-tosyl-1,2,3,6-tetrahydropyridin-3-ol (3aj)

A white solid. 85% yield (48 mg). M. P. 143-145 °C. ^{1}H NMR (400 MHz, $CDCl_3$, TMS) δ 1.75 (s, 3H), 2.42 (s, 3H), 2.82 (d, J = 11.6 Hz, 1H), 3.10 (s, 1H), 3.30 (d, J = 16.0 Hz, 1H), 3.51 (d, J = 18.4 Hz, 1H), 3.61 (d, J = 11.6 Hz, 1H), 3.87-3.93 (m, 2H), 4.20 (d, J = 17.6 Hz, 1H), 4.27 (d, J = 17.6 Hz, 1H), 4.71 (d, J = 1.6 Hz, 1H), 4.95 (d, J = 1.6 Hz, 1H), 6.12 (d, J = 8.0 Hz, 2H), 6.57 (dd, J_1 = 7.2 Hz, J_2 = 7.2 Hz, 1H), 6.96 (dd, J_1 = 7.6 Hz, J_2 = 8.8 Hz, 2H), 7.09 (d, J = 7.2 Hz, 1H), 7.19-7.23 (m, 1H), 7.26 (d, J = 7.6 Hz, 2H), 7.29-7.33 (m, 5H), 7.41-7.43 (m, 2H), 7.64 (d, J = 8.0 Hz, 2H). ^{13}C NMR (100 MHz, $CDCl_3$, TMS) δ 18.2, 21.5, 49.6, 54.0, 55.5, 57.6, 72.7, 112.1, 116.3, 126.56, 126.58, 126.73, 127.4, 127.8, 127.9, 128.5, 128.8, 129.7, 129.8, 132.4, 136.4, 138.5, 139.8, 141.2, 144.0, 148.6. IR (CH_2Cl_2): ν 3734, 3087, 3067, 3029, 2990, 2918, 2854, 2806, 2360, 2342, 1597, 1559, 1506, 1449, 1394, 1346, 1305, 1234, 1191, 1154, 1091, 1046, 1028, 988, 958,

919, 861, 808, 770, 747, 732, 694, 662 cm^{-1}. HRMS (ESI) calcd. for $C_{35}H_{37}N_2O_3S$ $(M+H)^+$: 565.2519, Found: 565.2511. Enantiomeric excess was determined by HPLC with a Chiralcel IC-H column [λ = 230 nm; eluent: Hexane/Isopropanol = 80/20; Flow rate: 0.50 mL/min; t_{minor} = 23.42 min, t_{major} = 25.19 min; ee% > 99%; $[\alpha]^{20}_D$ = +80.0 (c 1.00, CH_2Cl_2)].

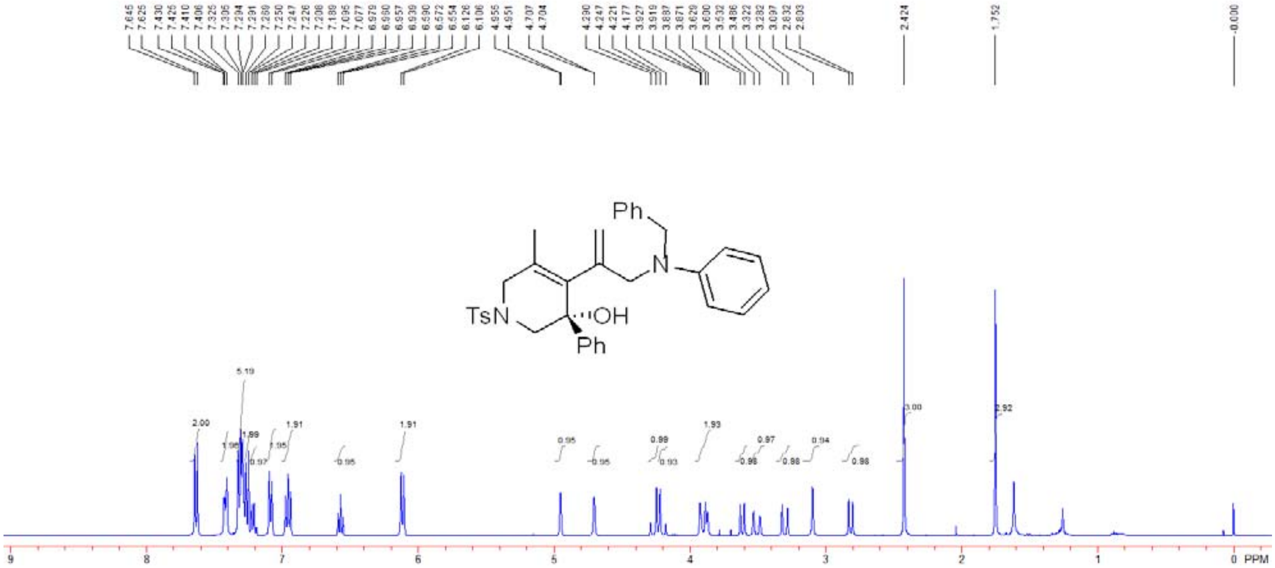

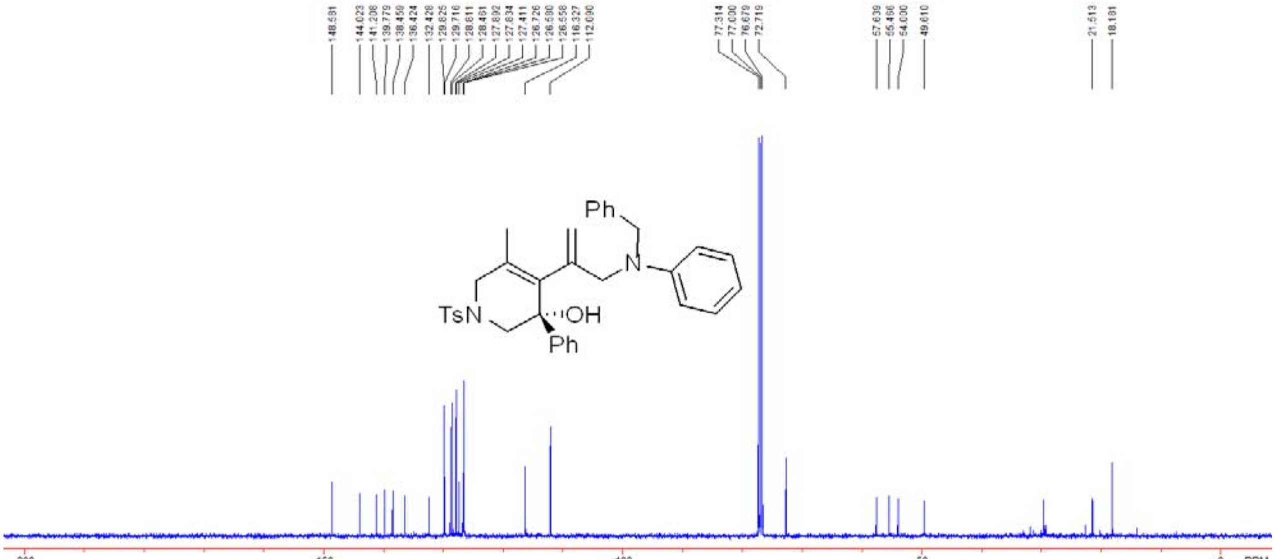

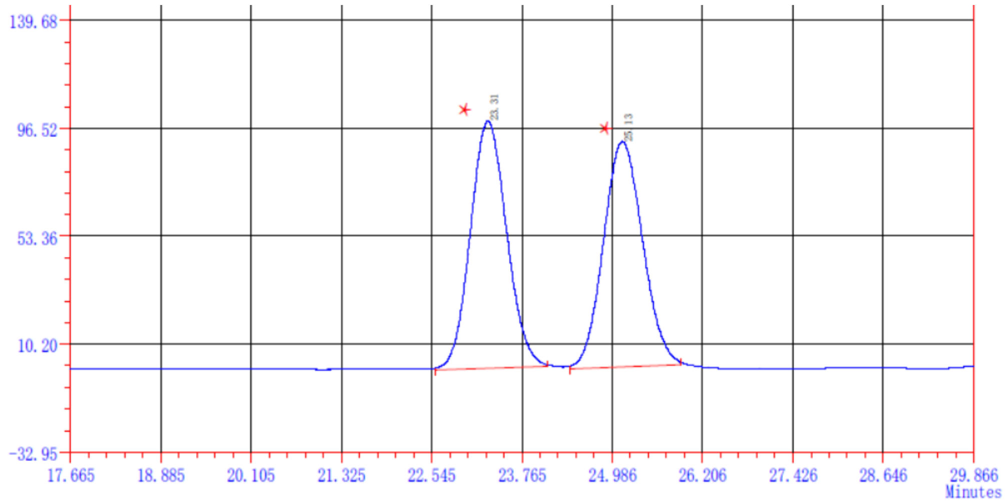

ID	组分名	保留时间	峰高	峰面积	浓度	拖尾因子	理论塔板
1		23.308	98951	3371638.3	50.3564	1.10	9326
2		25.125	90185	3323914.9	49.6436	1.10	9262
	Σ:		189136	6695553.2	100.0000		

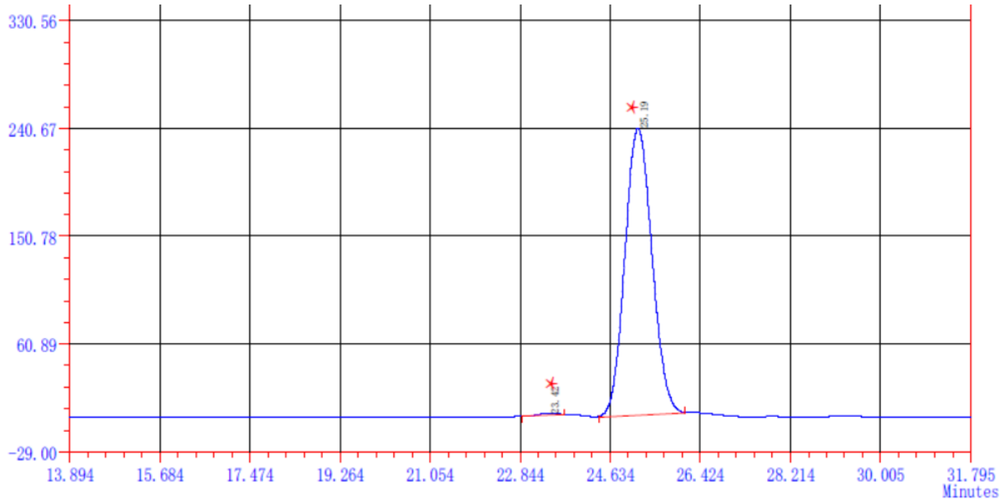

ID	组分名	保留时间	峰高	峰面积	浓度	拖尾因子	理论塔板
1		23.417	1365	33620.9	0.3721	1.06	18014
2		25.187	239861	9002899.9	99.6279	1.10	8975
	Σ:		241226	9036520.8	100.0000		

Translation: Chiralcel IC-H column [λ = 230 nm; eluent: Hexane/Isopropanol = 80/20; Flow rate: 0.5 mL/min; t_{minor} = 23.42 min, t_{major} = 25.19 min; ee% > 99%].

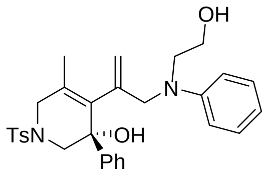

(S)-4-(3-((2-hydroxyethyl)(phenyl)amino)prop-1-en-2-yl)-5-methyl-3-phenyl-1-tosyl-1,2,3,6-tetrahydropyridin-3-ol (3ak)

A light yellow oil. 77% yield (40 mg). ^{1}H NMR (400 MHz, $CDCl_3$, TMS) δ 1.80 (s, 3H), 2.43 (s, 3H), 2.83 (d, J = 11.6 Hz, 1H), 3.11-3.26 (m, 2H), 3.34 (d, J = 16.0 Hz, 1H), 3.39 (brs, 1H), 3.52 (d, J = 18.8 Hz, 1H), 3.60-3.63 (m, 3H), 3.85 (d, J = 18.8 Hz, 1H), 3.93 (d, J = 16.0 Hz, 1H), 4.67 (s, 1H), 4.84 (s, 1H), 6.23 (d, J = 8.0 Hz, 2H), 6.60 (dd, J_1 = 7.2 Hz, J_2 = 7.2 Hz, 1H), 7.01 (dd, J_1 = 8.0 Hz, J_2 = 8.0 Hz, 2H), 7.31-7.36 (m, 5H), 7.45 (d, J = 7.2 Hz, 2H), 7.65 (d, J = 8.0 Hz, 2H). ^{13}C NMR (100 MHz, $CDCl_3$, TMS) δ 18.3, 21.5, 49.6, 53.3, 56.0, 57.7, 60.3, 72.8, 112.3, 116.6, 116.7, 126.6, 127.4, 127.9, 129.0, 129.8, 129.9, 132.5, 136.3, 140.2, 141.4, 144.1, 148.3. IR (CH_2Cl_2): ν 3537, 3054, 2962, 2924, 2845, 2806, 2359, 2341, 1597, 1540, 1506, 1449, 1338, 1160, 1090, 1048, 988, 863, 815, 748, 705 cm^{-1}. HRMS (ESI) calcd. for $C_{30}H_{35}N_2O_4S$ $(M+H)^+$: 519.2312, Found: 519.2304. Enantiomeric excess was determined by HPLC with a Chiralcel AD-H column [λ = 254 nm; eluent: Hexane/Isopropanol = 70/30; Flow rate: 0.50 mL/min; t_{minor} = 33.82 min, t_{major} = 26.16 min; ee% > 99%; $[\alpha]^{20}_{D}$ = -10.5 (c 1.00, CH_2Cl_2)].

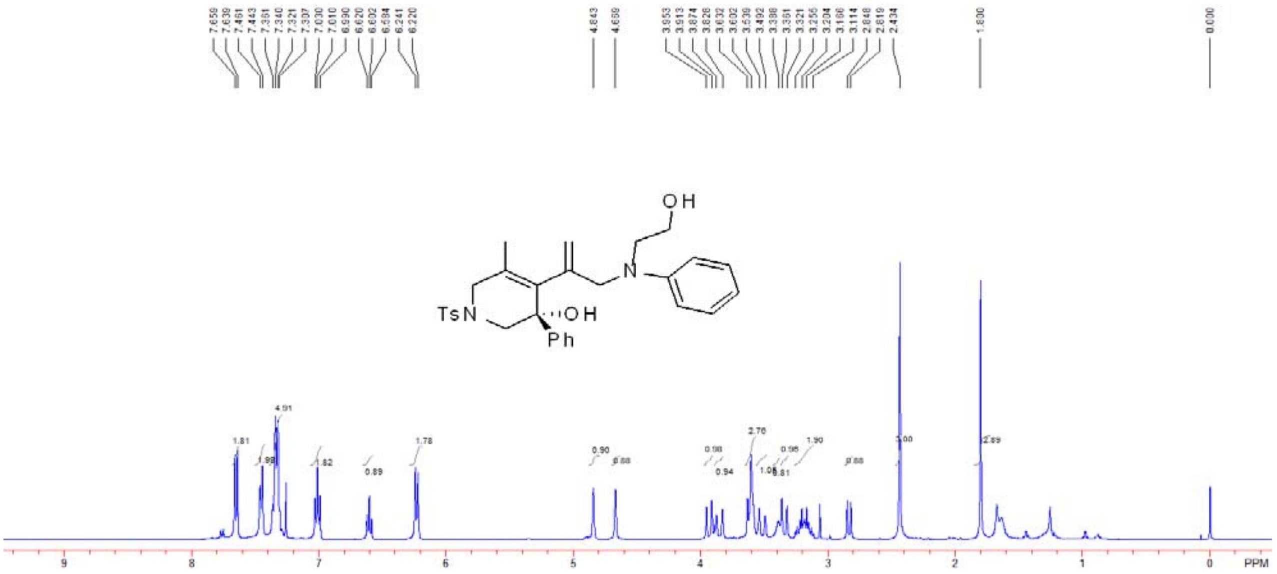

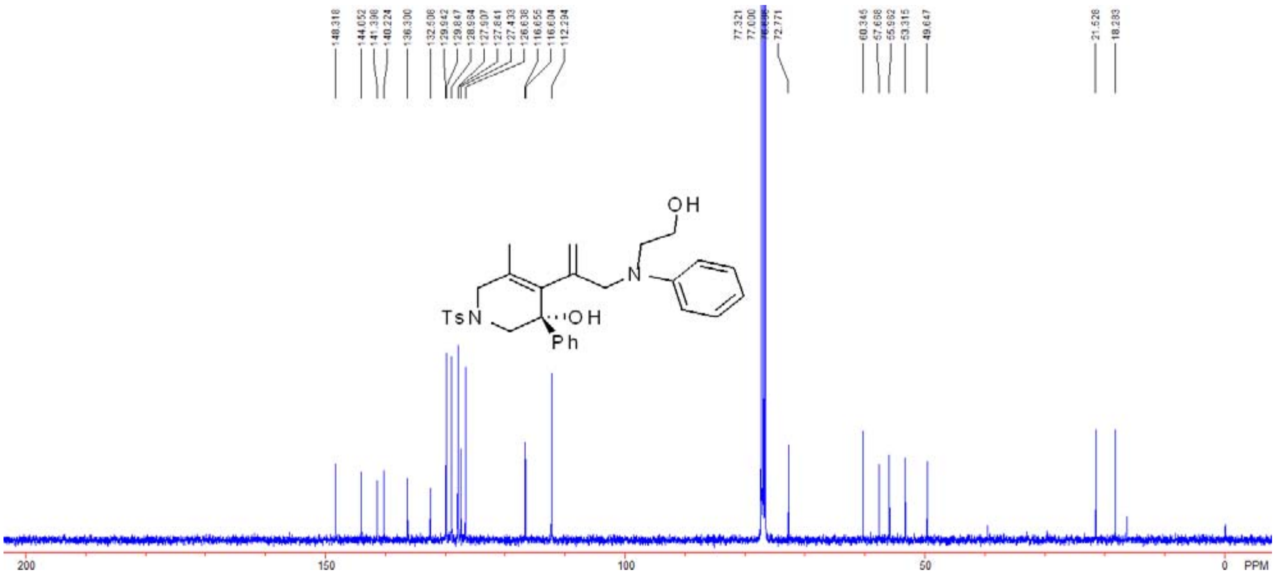

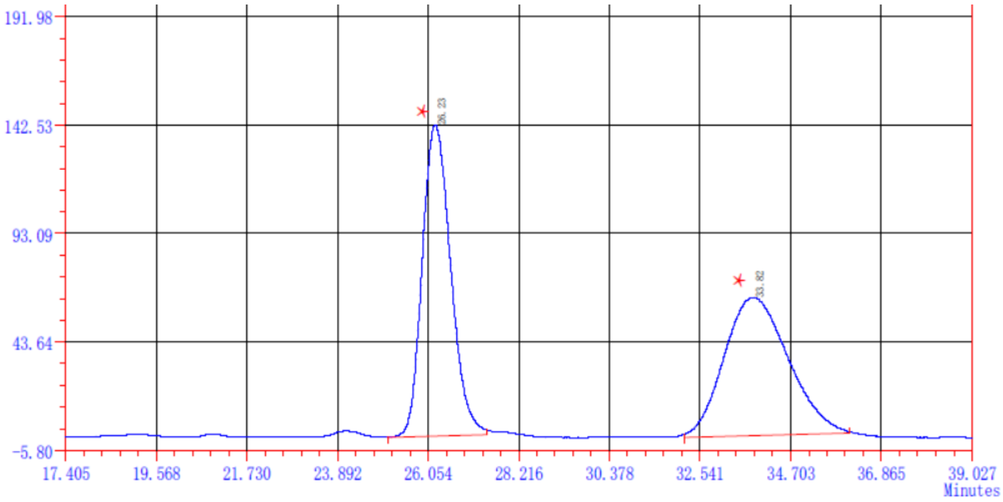

ID	组分名	保留时间	峰高	峰面积	浓度	拖尾因子	理论塔板
1		26.228	141905	6693165.5	49.9218	1.22	6163
2		33.818	63071	6714128.2	50.0782	1.22	2011
	Σ:		204976	13407293.7	100.0000		

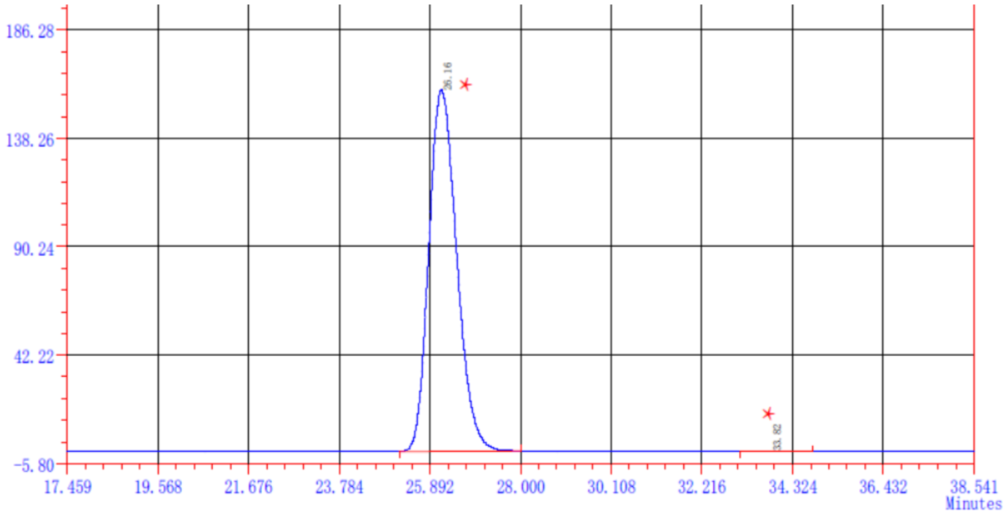

ID	组分名	保留时间	峰高	峰面积	浓度	拖尾因子	理论塔板
1		26.162	160041	7232018.0	99.8990	1.21	6680
2		33.820	136	7308.7	0.1010	1.10	7894
	Σ:		160177	7239326.7	100.0000		

Translation: Chiralcel AD-H column [λ = 254 nm; eluent: Hexane/Isopropanol = 70/30; Flow rate: 0.5 mL/min; t_{minor} = 33.82 min, t_{major} = 26.16 min; ee% > 99%].

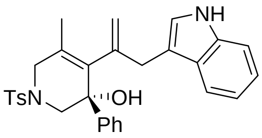

(S)-4-(3-(1H-indol-3-yl)prop-1-en-2-yl)-5-methyl-3-phenyl-1-tosyl-1,2,3,6-tetrahydropyridin-3-ol (5aa)

A white solid. 55% yield (27 mg). M. P. 183-185 °C. ^{1}H NMR (400 MHz, $CDCl_3$, TMS) δ 1.66 (s, 3H), 2.42 (s, 3H), 2.87 (s, 1H), 2.95 (d, J = 11.6 Hz, 1H), 3.17 (d, J = 17.2 Hz, 1H), 3.29 (d, J = 17.2 Hz, 1H), 3.38 (d, J = 16.0 Hz, 1H), 3.48 (d, J = 11.6 Hz, 1H), 3.78 (d, J = 16.0 Hz, 1H), 4.60 (s, 1H), 4.82 (d, J = 1.6 Hz, 1H), 6.79 (d, J = 3.0 Hz, 1H), 6.94-6.98 (m, 1H), 7.05 (d, J = 8.0 Hz, 1H), 7.09-7.13 (m, 1H), 7.28-7.36 (m, 6H), 7.50 (d, J = 8.0 Hz, 2H), 7.62 (d, J = 8.0 Hz, 2H), 7.92 (s, 1H). ^{13}C NMR (100 MHz, $CDCl_3$, TMS) δ 18.3, 21.5, 32.5, 49.5, 57.6, 72.8, 110.9, 112.8, 117.7, 119.0, 119.3, 121.7, 122.7, 126.6, 127.24, 127.78, 127.83, 128.6, 129.8, 132.5, 136.2, 137.9, 141.9, 143.9, 144.5. IR (CH_2Cl_2): ν 3455, 3328, 3062, 3031, 2970, 2921, 2848, 2820, 2360, 2342, 1598, 1491, 1447, 1393, 1346, 1184, 1169, 1153, 1107, 1090, 1051, 1039, 1018, 982, 944, 918, 900, 860, 809, 742, 703, 677, 661 cm^{-1}. HRMS (ESI) calcd. for $C_{30}H_{34}N_3O_3S$ $(M+NH_4)^+$: 516.2315, Found: 516.2310. Enantiomeric excess was determined by HPLC with a Chiralcel IC-H

column [λ = 254 nm; eluent: Hexane/Isopropanol = 80/20; Flow rate: 0.50 mL/min; t_{minor} = 13.68 min, t_{major} = 18.18 min; ee% > 99%; $[\alpha]^{20}_{D}$ = +38.2 (c 1.00, CH_2Cl_2)].

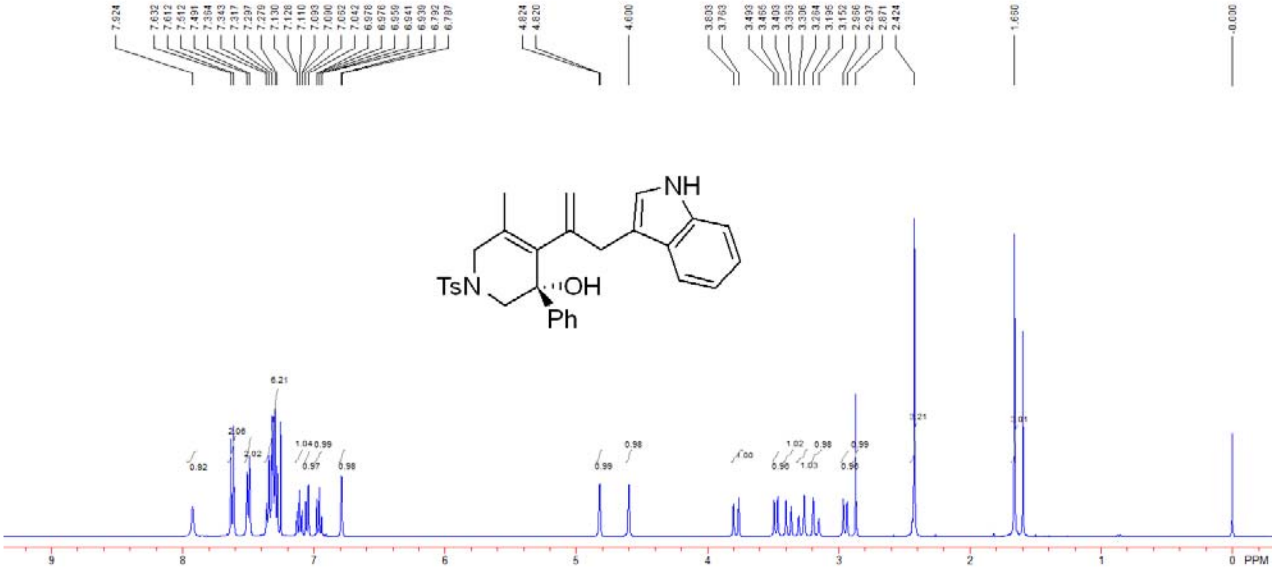

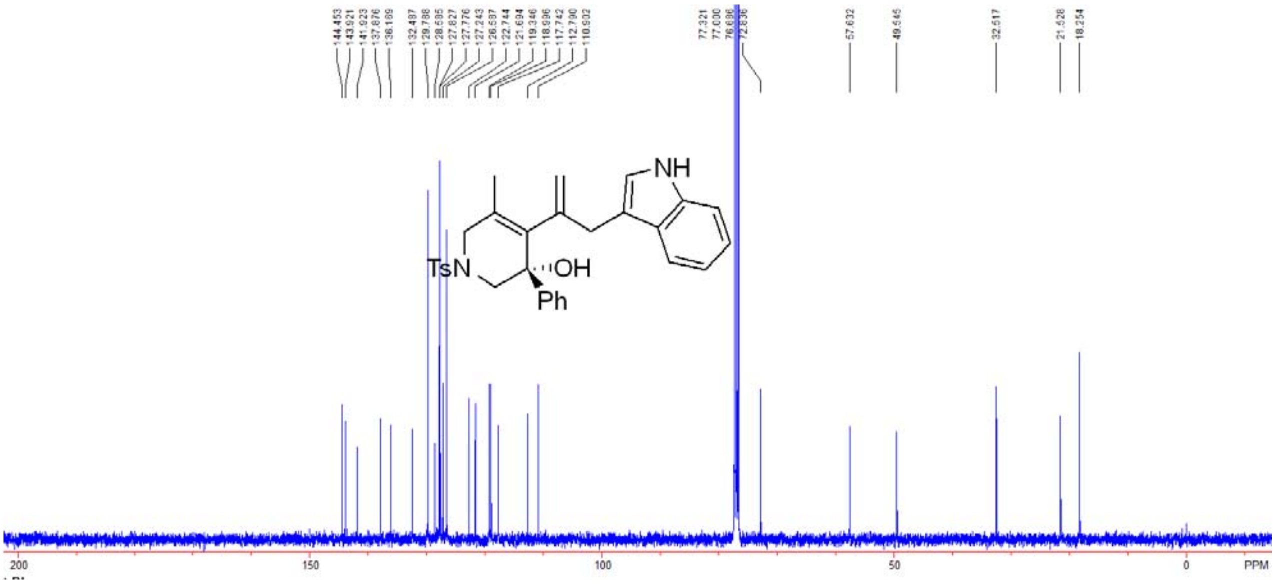

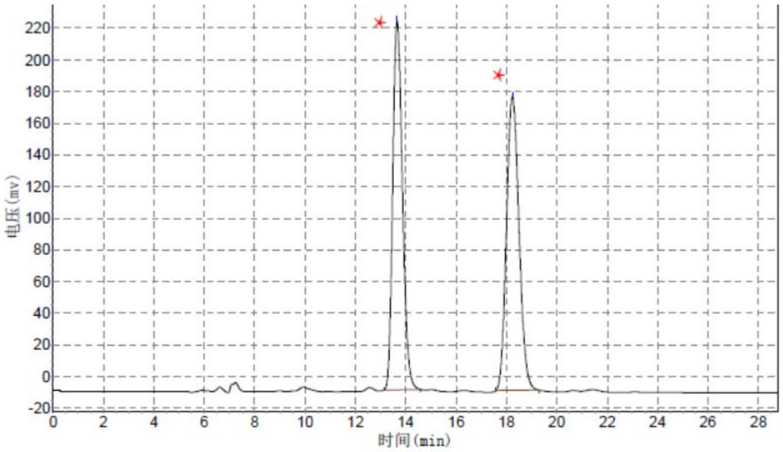

分析结果表

峰号	峰名	保留时间	峰高	峰面积	含量
1		13.655	233315.938	6251380.000	49.0075
2		18.232	185743.156	6416457.000	50.3016
总计			424228.453	12755965.969	100.0000

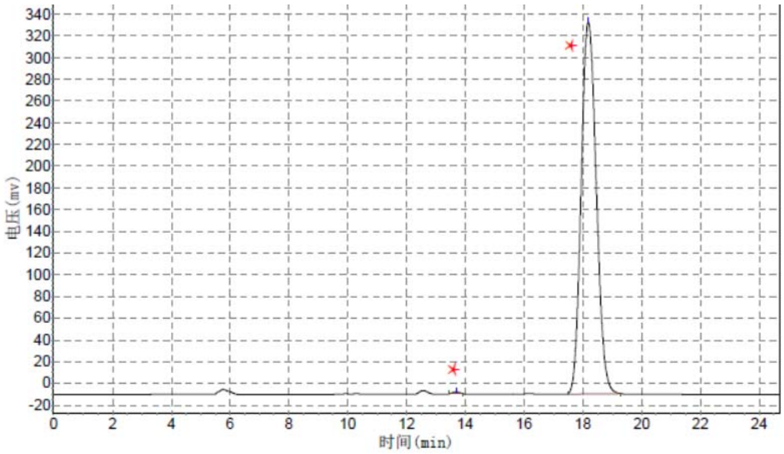

分析结果表

峰号	峰名	保留时间	峰高	峰面积	含量
1		13.678	1649.880	27886.191	0.2272
2		18.183	342628.938	12244637.000	99.7728
总计			344278.817	12272523.191	100.0000

Translation: Chiralcel IC-H column [λ = 230 nm; eluent: Hexane/Isopropanol = 70/30; Flow rate: 0.5 mL/min; t_{minor} = 13.68 min, t_{major} = 18.18 min; ee% > 99%].

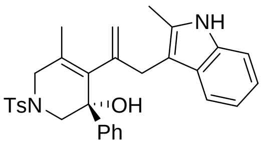

(S)-5-methyl-4-(3-(2-methyl-1H-indol-3-yl)prop-1-en-2-yl)-3-phenyl-1-tosyl-1,2,3,6-tetrahydropyridin-3-ol (5ab)

A light yellow oil. 63% yield (32 mg). ^{1}H NMR (400 MHz, $CDCl_3$, TMS) δ 1.81 (s, 3H), 1.99 (s,

3H), 2.43 (s, 3H), 2.91 (s, 1H), 2.94 (d, *J* = 18.4 Hz, 1H), 3.02 (d, *J* = 11.6 Hz, 1H), 3.25 (d, *J* = 18.4 Hz, 1H), 3.46 (d, *J* = 16.0 Hz, 1H), 3.49 (d, *J* = 11.6 Hz, 1H), 3.84 (d, *J* = 16.0 Hz, 1H), 4.51 (d, *J* = 2.0 Hz, 1H), 4.54 (d, *J* = 1.2 Hz, 1H), 6.89-6.94 (m, 2H), 7.00-7.04 (m, 1H), 7.18 (d, *J* = 8.0 Hz, 1H), 7.25-7.36 (m, 5H), 7.52 (d, *J* = 8.4 Hz, 2H), 7.64 (d, *J* = 8.0 Hz, 2H), 7.72 (s, 1H). ^{13}C NMR (100 MHz, $CDCl_3$, TMS) δ 11.2, 18.2, 21.5, 31.1, 49.5, 57.4, 72.8, 108.1, 110.0, 116.9, 118.2, 118.9, 120.7, 126.6, 127.26, 127.78, 127.83, 127.86, 128.8, 129.8, 132.1, 132.5, 135.1, 138.6, 142.0, 143.8, 143.9. IR (CH_2Cl_2): ν 3397, 3059, 3031, 2971, 2848, 2360, 2342, 1734, 1697, 1684, 1653, 1598, 1559, 1540, 1493, 1507, 1493, 1449, 1342, 1307, 1247, 1091, 1043, 1019, 986, 972, 929, 900, 873, 806, 744, 724, 703 cm^{-1}. HRMS (ESI) calcd. for $C_{31}H_{36}N_3O_3S$ $(M+NH_4)^+$: 530.2472, Found: 530.2448. Enantiomeric excess was determined by HPLC with a Chiralcel AD-H column [λ = 254 nm; eluent: Hexane/Isopropanol = 70/30; Flow rate: 0.50 mL/min; t_{minor} = 28.03 min, t_{major} = 24.78 min; ee% > 99%; $[\alpha]^{20}_{D}$ = +41.4 (c 1.00, CH_2Cl_2)].

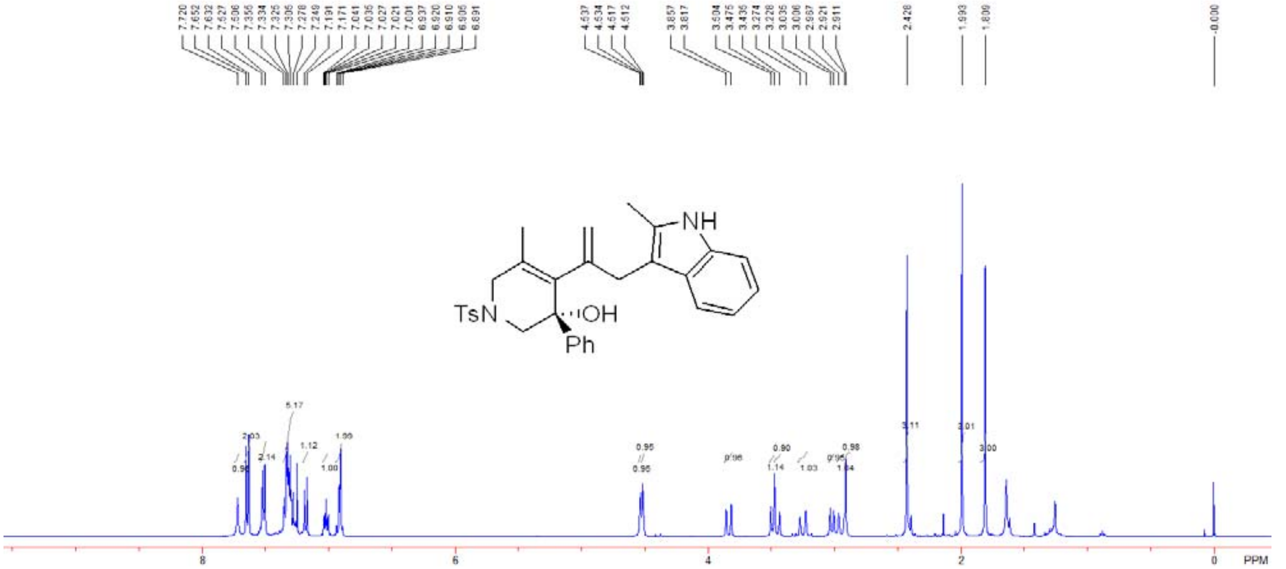

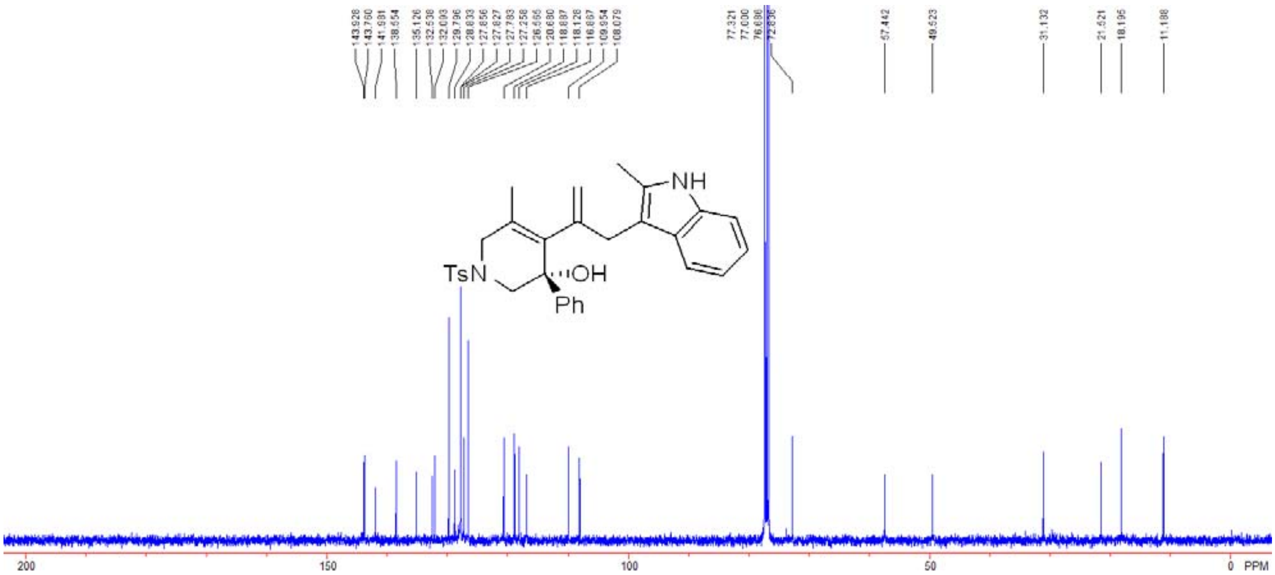

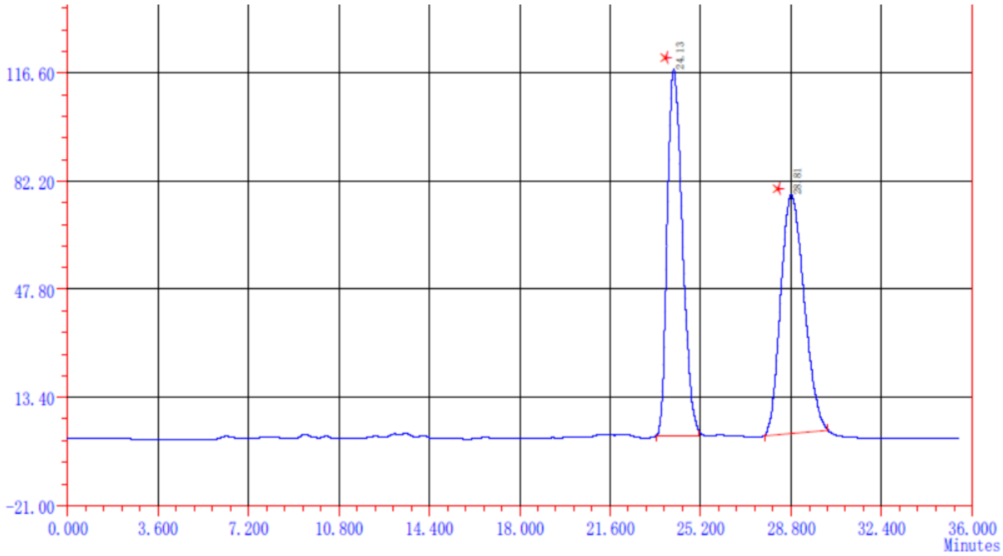

ID	组分名	保留时间	峰高	峰面积	浓度	拖尾因子	理论塔板
1		24.132	116638	4882377.4	49.7046	1.29	6624
2		28.812	75950	4940403.2	50.2954	1.22	3910
	Σ:		192588	9822780.6	100.0000		

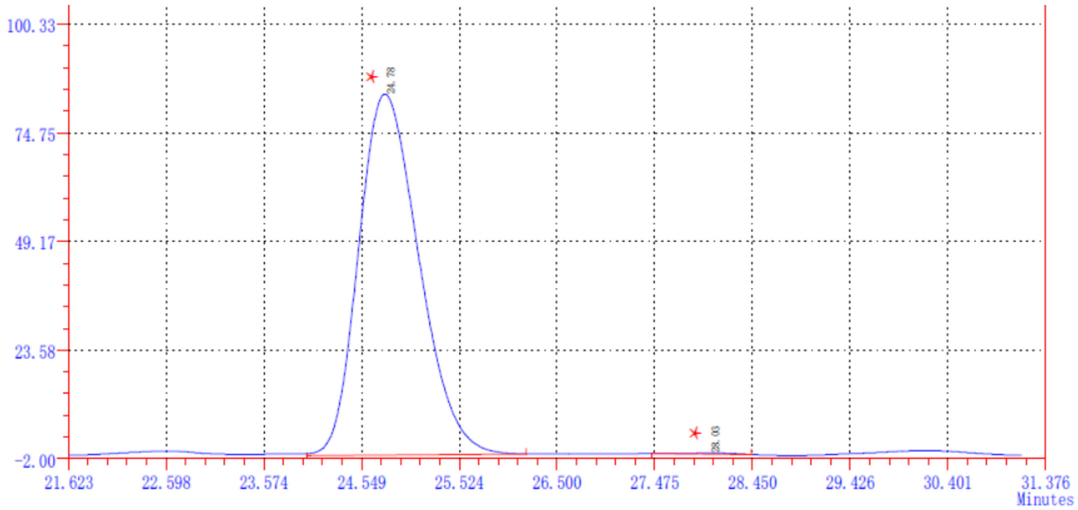

ID	组分名	保留时间	峰高	峰面积	浓度	拖尾因子	理论塔板
1		24.783	84982	3558650.7	99.7313	1.25	6981
2		28.025	313	9586.3	0.2687	0.80	16688
	Σ:		85295	3568237.0	100.0000		

Translation: Chiralcel AD-H column [λ = 254 nm; eluent: Hexane/Isopropanol = 70/30; Flow rate: 0.5 mL/min; t_{minor} = 28.03 min, t_{major} = 24.78 min; ee% > 99%].

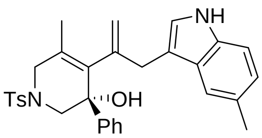

(S)-5-methyl-4-(3-(5-methyl-1H-indol-3-yl)prop-1-en-2-yl)-3-phenyl-1-tosyl-1,2,3,6-tetrahydropyridin-3-ol (5ac)

A white solid. 61% yield (31 mg). M. P. 109-111 °C. ^{1}H NMR (400 MHz, $CDCl_3$, TMS) δ 1.69 (s, 3H), 2.35 (s, 3H), 2.42 (s, 3H), 2.89 (s, 1H), 2.96 (d, *J* = 11.2 Hz, 1H), 3.12 (d, *J* = 17.2 Hz, 1H), 3.27 (d, *J* = 17.2 Hz, 1H), 3.40 (d, *J* = 16.0 Hz, 1H), 3.48 (d, *J* = 11.2 Hz, 1H), 3.80 (d, *J* = 16.0 Hz, 1H), 4.59 (s, 1H), 4.78 (d, *J* = 1.6 Hz, 1H), 6.74 (d, *J* = 2.0 Hz, 1H), 6.76 (s, 1H), 6.92 (dd, J_1 = 1.2 Hz, J_2 = 8.4 Hz, 1H), 7.16 (d, *J* = 8.4 Hz, 1H), 7.29-7.37 (m, 5H), 7.51 (d, *J* = 8.4 Hz, 2H), 7.62 (d, *J* = 8.4 Hz, 2H), 7.84 (brs, 1H). ^{13}C NMR (100 MHz, $CDCl_3$, TMS) δ 18.2, 21.3, 21.5, 32.5, 49.5, 57.6, 72.9, 110.5, 112.2, 117.6, 118.8, 122.9, 123.3, 126.6, 127.32, 127.76, 127.81, 127.83, 129.8, 132.6, 134.5, 138.0, 142.0, 143.9, 144.5. IR (CH_2Cl_2): ν 3402, 3029, 2987, 2912, 2851, 2361, 1598, 1492, 1448, 1379, 1344, 1249, 1155, 1090, 1040, 986, 916, 862, 793, 766, 703 cm^{-1}. HRMS (ESI) calcd. for $C_{31}H_{36}N_3O_3S$ $(M+NH_4)^+$: 530.2472, Found: 530.2465. Enantiomeric excess was determined by HPLC with a Chiralcel IC-H column [λ = 230 nm; eluent:

Hexane/Isopropanol = 70/30; Flow rate: 0.50 mL/min; t_{minor} = 19.29 min, t_{major} = 39.12 min; ee% = 99%; $[\alpha]^{20}_{D}$ = +36.2 (c 1.00, CH_2Cl_2)].

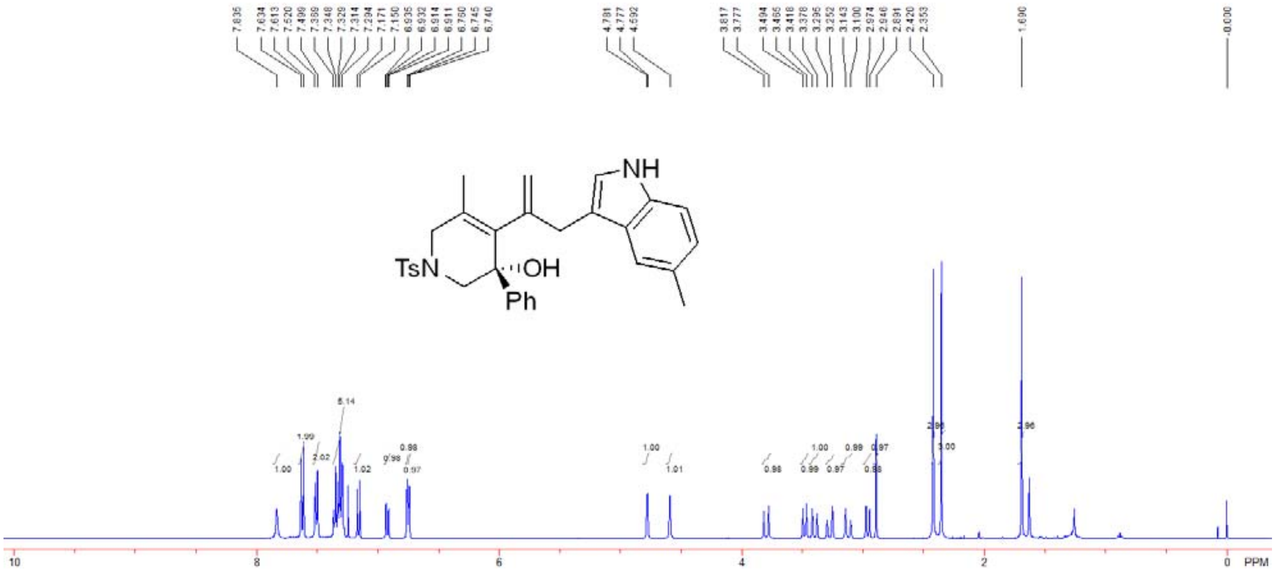

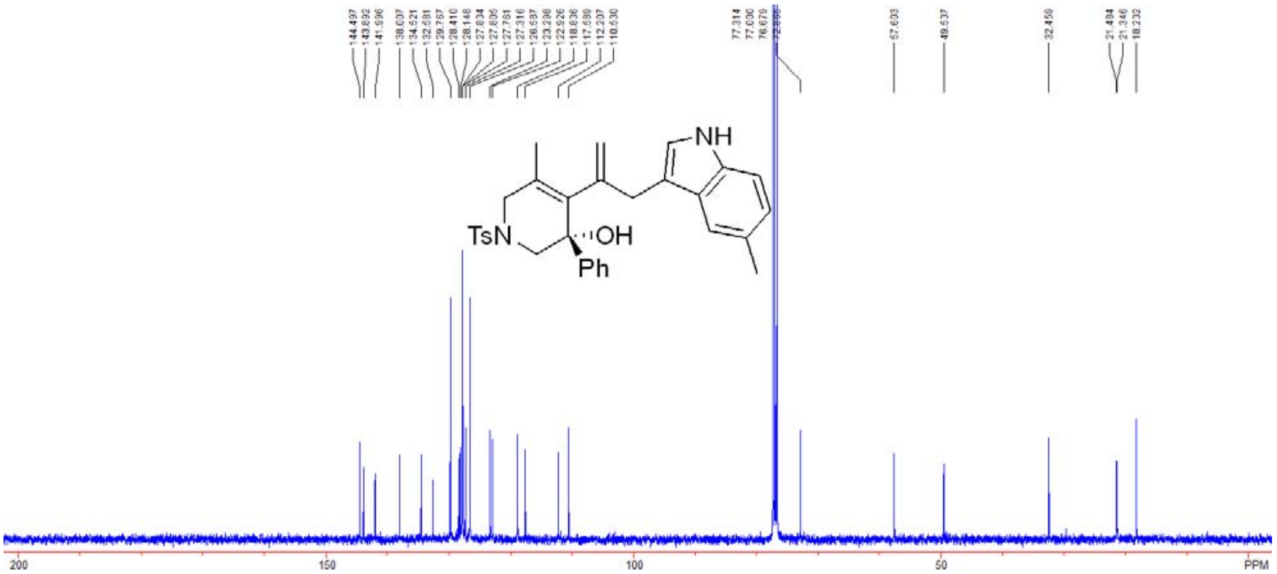

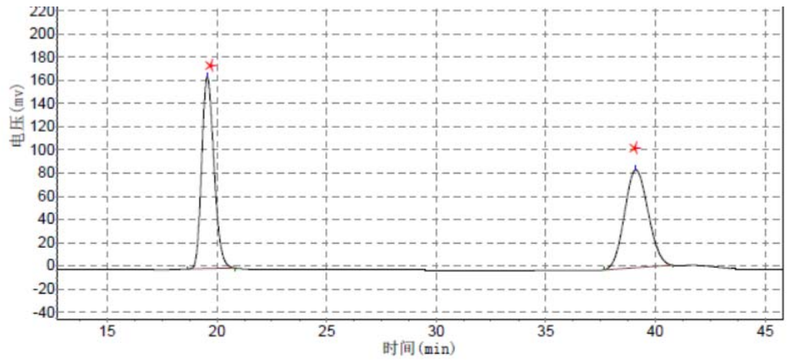

分析结果表

峰号	峰名	保留时间	峰高	峰面积	含量
1		19.550	165355.641	6410473.000	50.3970
2		39.095	84646.508	6309470.000	49.6030
总计			250002.148	12719943.000	100.0000

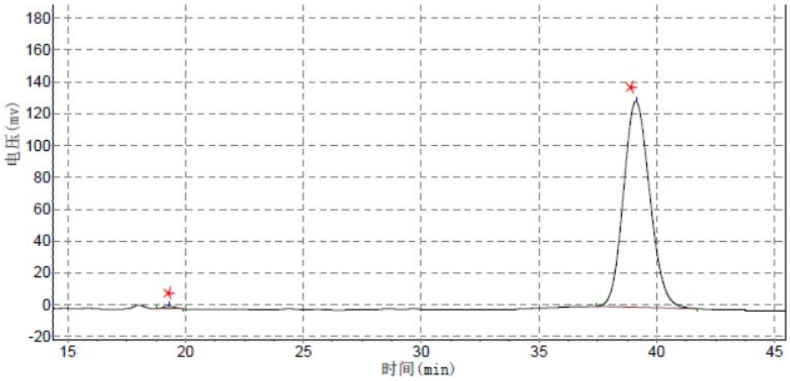

分析结果表

峰号	峰名	保留时间	峰高	峰面积	含量
1		19. 290	1756. 268	58617. 695	0. 5807
2		39. 120	129339. 914	10035588. 000	99. 4193
总计			131096. 182	10094205. 695	100. 0000

Translation: Chiralcel IC-H column [λ = 230 nm; eluent: Hexane/Isopropanol = 70/30; Flow rate: 0.5 mL/min; t_{minor} = 19.29 min, t_{major} = 39.12 min; ee% = 99%].

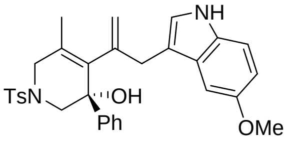

(S)-4-(3-(5-methoxy-1H-indol-3-yl)prop-1-en-2-yl)-5-methyl-3-phenyl-1-tosyl-1,2,3,6-tetrahydropyridin-3-ol (5ad)

A light yellow oil. 60% yield (31 mg). ^{1}H NMR (400 MHz, $CDCl_3$, TMS) δ 1.67 (s, 3H), 2.42 (s, 3H), 2.86 (s, 1H), 2.96 (d, J = 11.2 Hz, 1H), 3.14 (d, J = 16.8 Hz, 1H), 3.22 (d, J = 16.8 Hz, 1H), 3.40 (d, J = 16.0 Hz, 1H), 3.45 (d, J = 11.2 Hz, 1H), 3.767 (s, 3H), 3.770 (d, J = 16.0 Hz, 1H), 4.60 (s, 1H), 4.84 (d, J = 2.0 Hz, 1H), 6.64 (d, J = 2.0 Hz, 1H), 6.78 (dd, J_1 = 2.0 Hz, J_2 = 9.2 Hz, 2H), 7.18 (d, J = 8.8 Hz, 1H), 7.27-7.34 (m, 5H), 7.50 (d, J = 8.4 Hz, 2H), 7.62 (d, J = 8.4 Hz, 2H), 7.85 (brs, 1H). ^{13}C NMR (100 MHz, $CDCl_3$, TMS) δ 18.2, 21.5, 32.6, 49.5, 56.0, 57.6, 72.9, 101.2, 111.6, 111.9, 112.5, 117.7, 123.6, 126.5, 127.3, 127.7, 127.8, 128.1, 128.5, 129.8, 131.4, 132.6, 137.9, 142.0, 143.9, 144.4, 153.7. IR (CH_2Cl_2): ν 3735, 3397, 3059, 3026, 2979, 2917, 2851, 2360, 2342, 1625, 1596, 1490, 1448, 1379, 1342, 1305, 1248, 1155, 1091, 1043, 986, 917, 863, 792, 767, 703 cm^{-1}. HRMS (ESI) calcd. for $C_{31}H_{36}N_3O_4S$ $(M+NH_4)^+$: 546.2421, Found: 546.2414. Enantiomeric excess was determined by HPLC with a Chiralcel IC-H column [λ = 230 nm; eluent: Hexane/Isopropanol = 70/30; Flow rate: 0.50 mL/min; t_{minor} = 24.65 min, t_{major} = 36.97 min; ee% > 99%; $[\alpha]^{20}_D$ = +32.0 (c 1.00, CH_2Cl_2)].

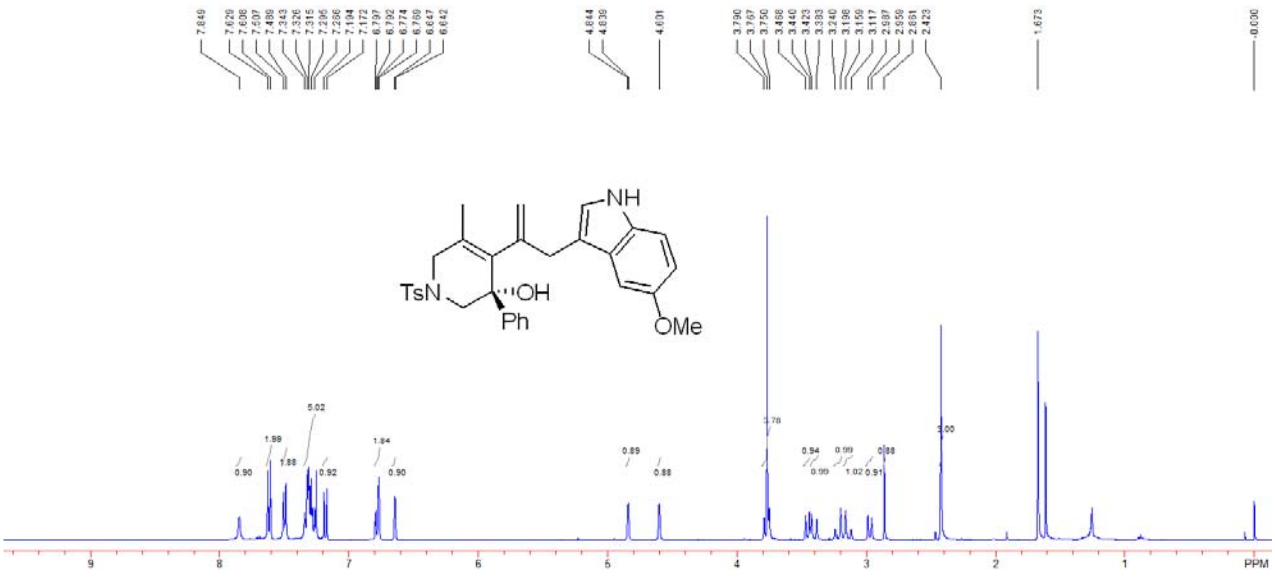

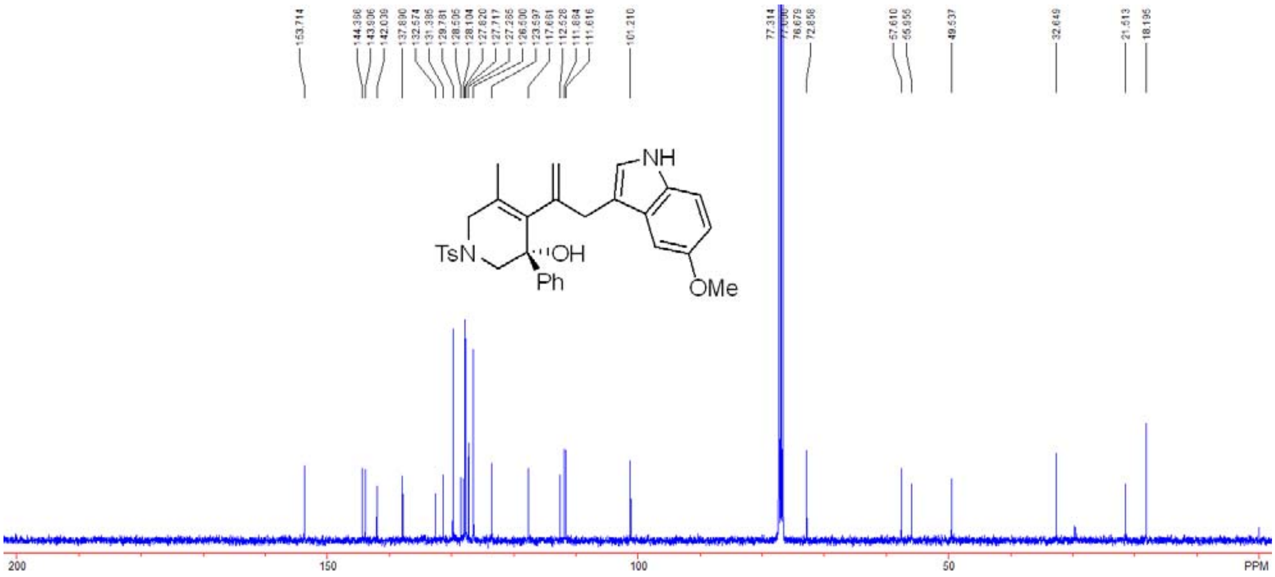

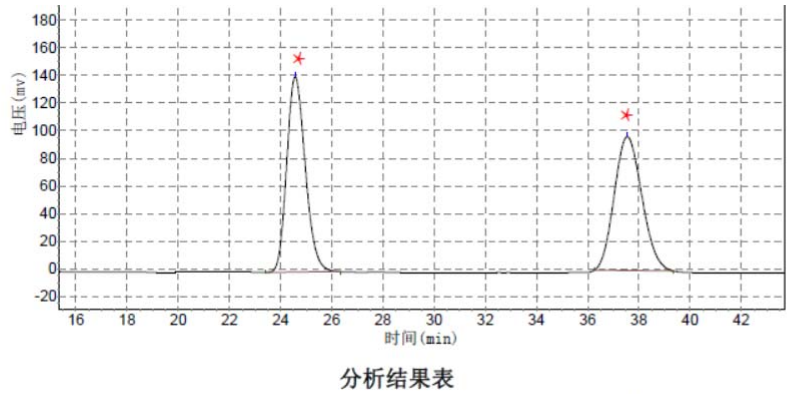

分析结果表

峰号	峰名	保留时间	峰高	峰面积	含量
1		24.565	141465.172	7141699.000	49.5385
2		37.548	96986.609	7274751.500	50.4615
总计			238451.781	14416450.500	100.0000

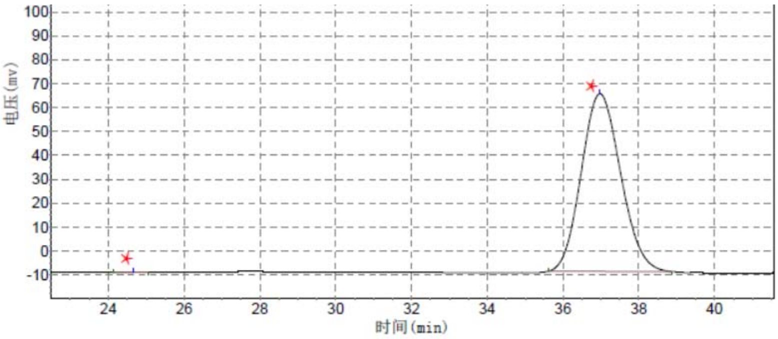

分析结果表

峰号	峰名	保留时间	峰高	峰面积	含量
1		24.653	154.932	5115.632	0.0953
2		36.972	74336.914	5364168.000	99.9047
总计			74491.846	5369283.632	100.0000

Translation: Chiralcel IC-H column [λ = 230 nm; eluent: Hexane/Isopropanol = 70/30; Flow rate: 0.5 mL/min; t_{minor} = 24.65 min, t_{major} = 36.97 min; ee% > 99%].

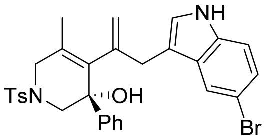

(S)-4-(3-(5-bromo-1H-indol-3-yl)prop-1-en-2-yl)-5-methyl-3-phenyl-1-tosyl-1,2,3,6-tetrahydropyridin-3-ol (5ae)

A light yellow oil. 54% yield (31 mg). ^{1}H NMR (400 MHz, $CDCl_3$, TMS) δ 1.66 (s, 3H), 2.43 (s, 3H), 2.88-2.91 (m, 2H), 3.12 (d, *J* = 17.2 Hz, 1H), 3.26 (d, *J* = 17.2 Hz, 1H), 3.34 (d, *J* = 16.0 Hz, 1H), 3.54 (d, *J* = 11.6 Hz, 1H), 3.83 (d, *J* = 16.0 Hz, 1H), 4.60 (s, 1H), 4.75 (d, *J* = 2.0 Hz, 1H), 6.81 (s, 1H), 7.08 (s, 1H), 7.13-7.19 (m, 2H), 7.30-7.38 (m, 5H), 7.49 (d, *J* = 6.4 Hz, 2H), 7.63 (d, *J* = 8.4 Hz, 2H), 804 (brs, 1H). ^{13}C NMR (100 MHz, $CDCl_3$, TMS) δ 18.3, 21.5, 32.3, 49.6, 57.7, 72.8, 112.36, 112.42, 112.54, 117.8, 121.8, 124.6, 126.5, 127.6, 127.8, 127.9, 128.8, 129.4, 129.8, 132.4, 134.8, 137.8, 141.6, 144.0, 144.2. IR (CH_2Cl_2): ν 3456, 3051, 3026, 2967, 2922, 2852, 2360, 2342, 1653, 1605, 1492, 1448, 1395, 1346, 1289, 1249, 1091, 1044, 985, 909, 861, 882, 810, 790, 764, 665 cm^{-1}. HRMS (ESI) calcd. for $C_{30}H_{33}N_3BrO_3S$ $(M+NH_4)^+$: 594.1421, Found: 594.1416. Enantiomeric excess was determined by HPLC with a Chiralcel IC-H column [λ = 230 nm; eluent: Hexane/Isopropanol = 70/30; Flow rate: 0.50 mL/min; t_{minor} = 13.76 min, t_{major} = 16.95 min; ee% = 97%; $[\alpha]^{20}_{D}$ = +22.6 (c 1.00, CH_2Cl_2)].

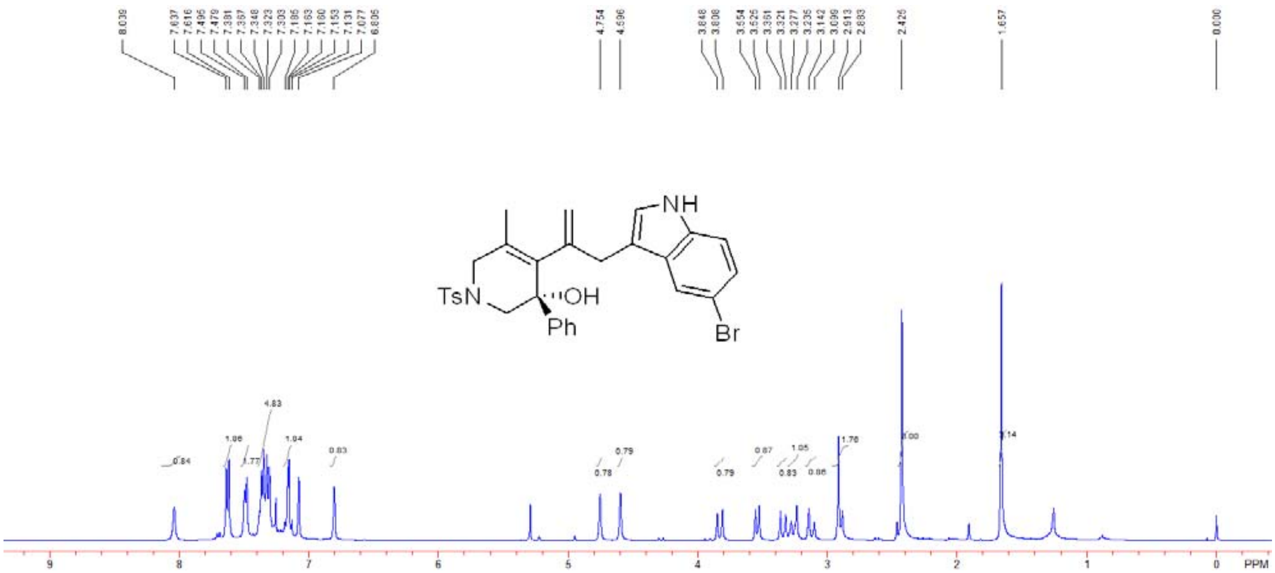

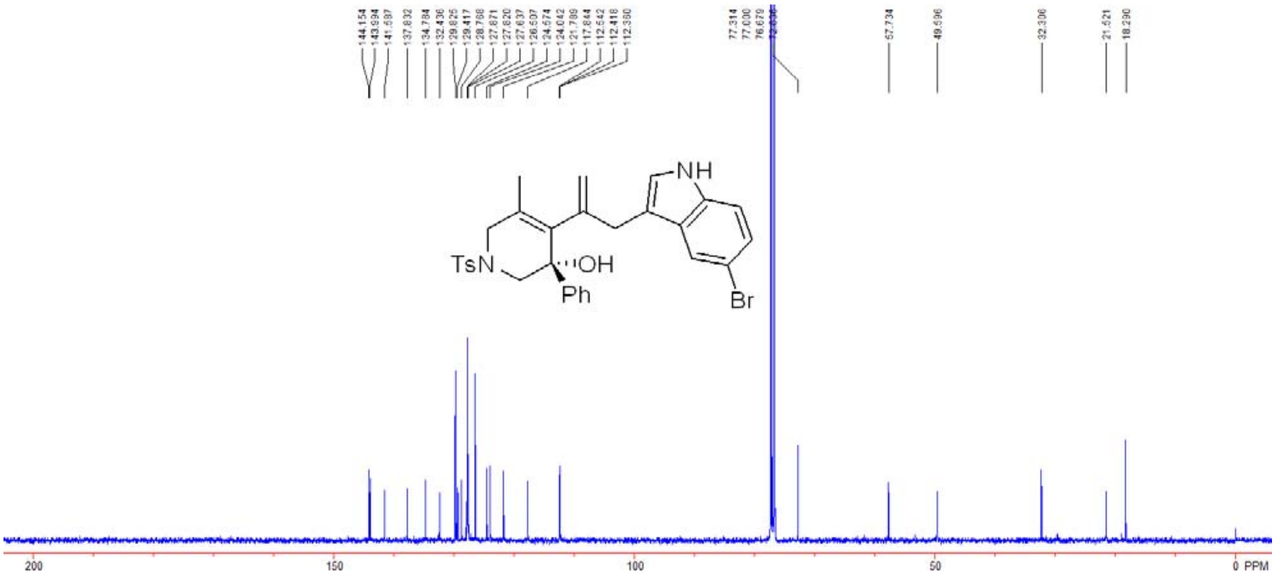

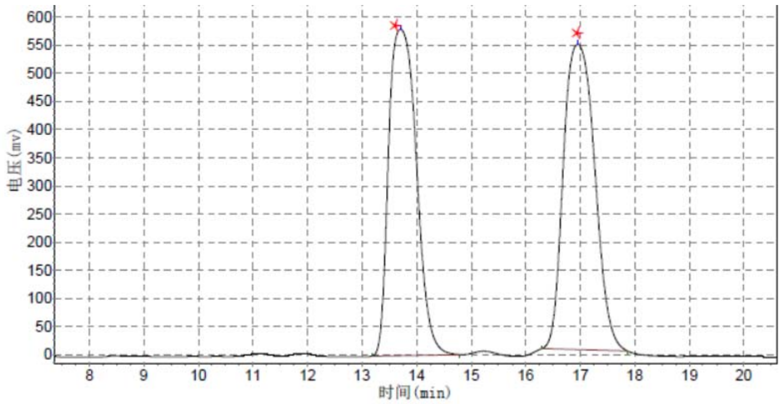

分析结果表

峰号	峰名	保留时间	峰高	峰面积	含量
1		13.705	580307.313	20481512.000	48.6309
2		16.957	542767.625	21634740.000	51.3691
总计			1123074.938	42116252.000	100.0000

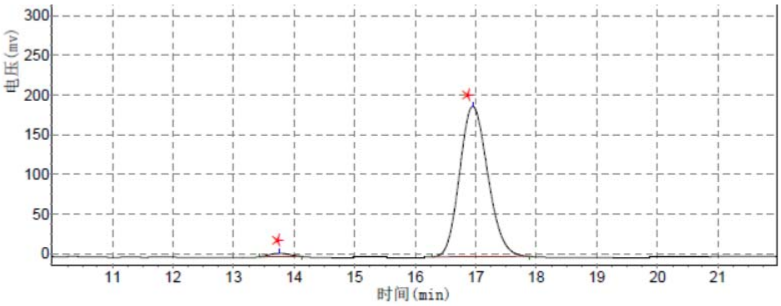

分析结果表

峰号	峰名	保留时间	峰高	峰面积	含量
1		13. 755	4237. 969	96335. 094	1. 5486
2		16. 952	188939. 391	6124596. 500	98. 4514
总计			193177. 359	6220931. 594	100. 0000

Translation: Chiralcel IC-H column [λ = 230 nm; eluent: Hexane/Isopropanol = 70/30; Flow rate: 0.5 mL/min; t_{minor} = 13.76 min, t_{major} = 16.95 min; ee% = 97%].

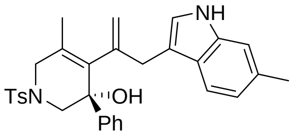

(S)-5-methyl-4-(3-(6-methyl-1H-indol-3-yl)prop-1-en-2-yl)-3-phenyl-1-tosyl-1,2,3,6-tetrahydropyridin-3-ol (5af)

A light yellow oil. 67% yield (34 mg). ^{1}H NMR (400 MHz, $CDCl_3$, TMS) δ 1.67 (s, 3H), 2.40 (s, 3H), 2.42 (s, 3H), 2.86 (s, 1H), 2.97 (d, J = 11.2 Hz, 1H), 3.13 (d, J = 16.8 Hz, 1H), 3.25 (d, J = 16.8 Hz, 1H), 3.40 (d, J = 16.0 Hz, 1H), 3.46 (d, J = 11.2 Hz, 1H), 3.78 (d, J = 16.0 Hz, 1H), 4.59 (s, 1H), 4.81 (d, J = 1.6 Hz, 1H), 6.70 (d, J = 2.0 Hz, 1H), 6.79 (d, J = 8.0 Hz, 1H), 6.90 (d, J = 8.4 Hz, 1H), 7.07 (s, 1H), 7.27-7.35 (m, 5H), 7.49 (d, J = 6.8 Hz, 2H), 7.62 (d, J = 8.4 Hz, 2H), 7.82 (brs, 1H). ^{13}C NMR (100 MHz, $CDCl_3$, TMS) δ 18.2, 21.5, 21.6, 32.6, 49.5, 57.6, 72.8, 110.9, 112.6, 117.6, 119.0, 120.8, 122.1, 125.5, 126.6, 127.22, 127.75, 127.82, 128.5, 129.8, 131.4, 132.6, 136.7, 137.9, 142.0, 143.9, 144.5. IR (CH_2Cl_2): ν 2954, 2923, 2854, 2360, 2342, 2366, 2298, 1507, 1496, 1457, 1159, 1081, 967, 937, 668 cm^{-1}. HRMS (ESI) calcd. for $C_{31}H_{36}N_3O_3S$ $(M+NH_4)^+$: 530.2472, Found: 530.2466. Enantiomeric excess was determined by HPLC with a Chiralcel IC-H column [λ = 230 nm; eluent: Hexane/Isopropanol = 70/30; Flow rate: 0.50 mL/min; t_{minor} = 14.81 min, t_{major} = 21.12 min; ee% > 99%; $[\alpha]^{20}_D$ = +50.6 (c 1.00, CH_2Cl_2)].

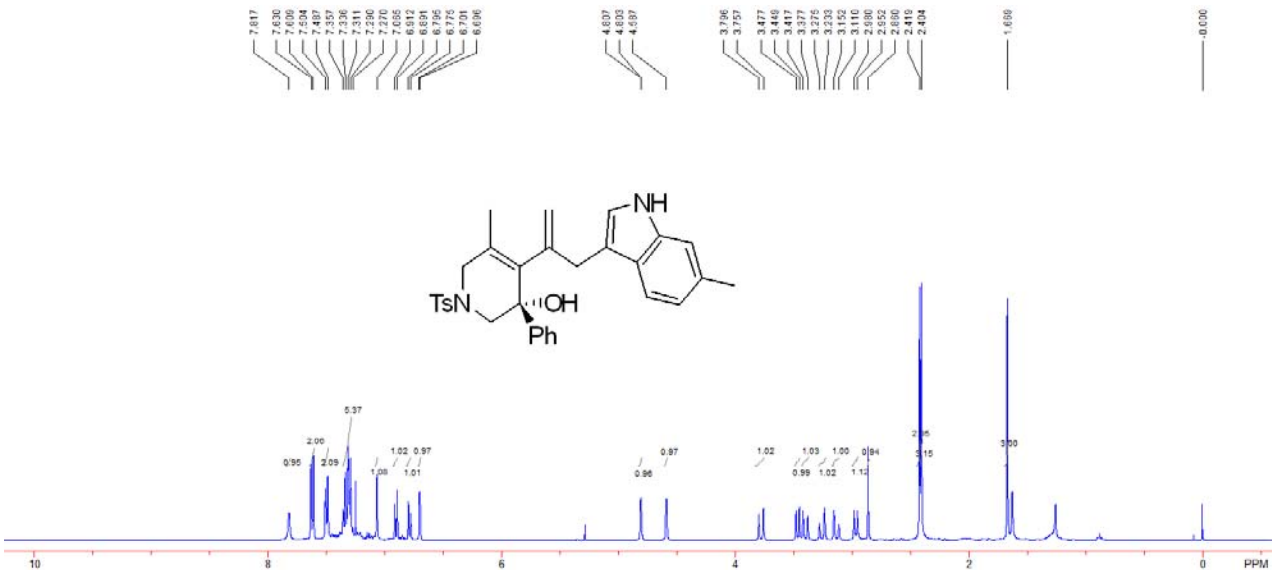

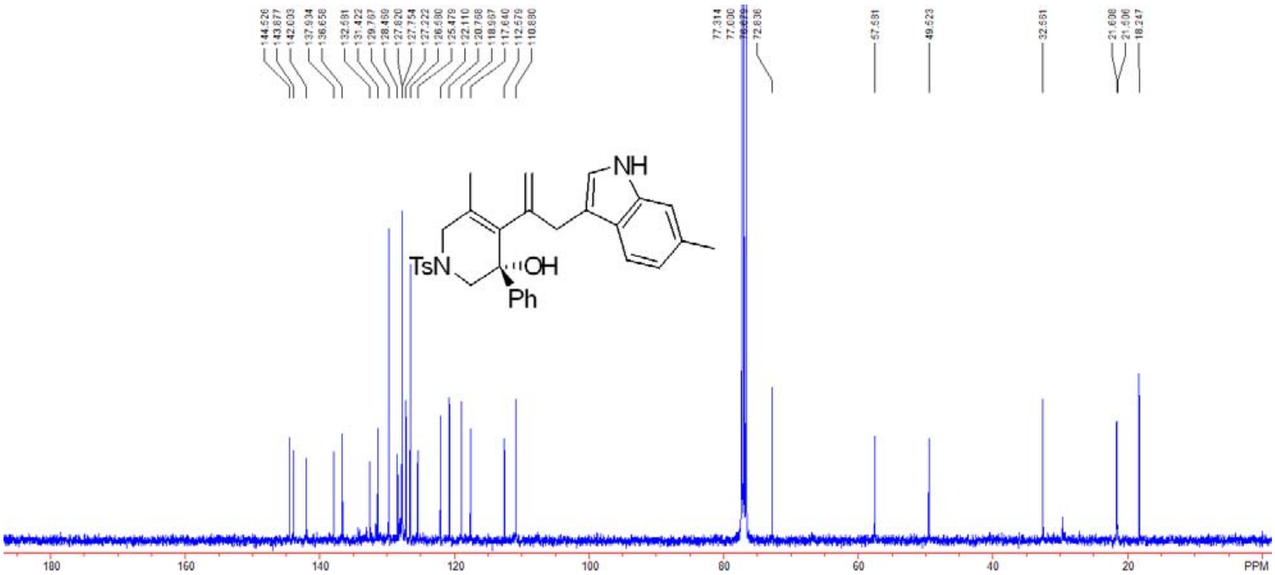

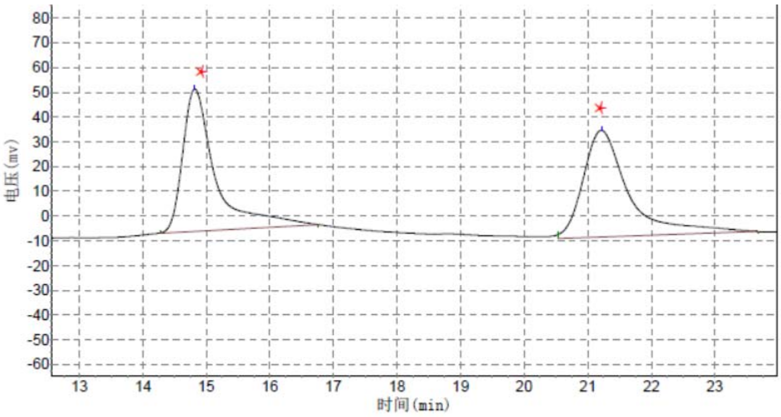

分析结果表

峰号	峰名	保留时间	峰高	峰面积	含量
1		14.815	57463.051	2061609.375	49.0155
2		21.215	43103.563	2144426.000	50.9845
总计			100566.613	4206035.375	100.0000

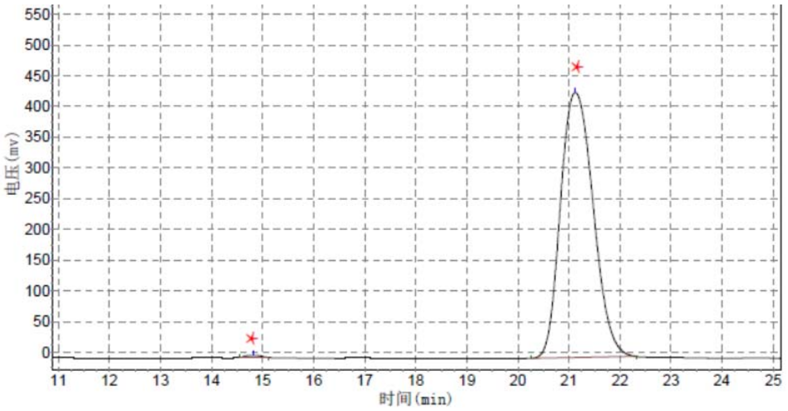

分析结果表

峰号	峰名	保留时间	峰高	峰面积	含量
1		14.805	3466.641	68885.703	0.3579
2		21.123	430875.281	19176144.000	99.6421
总计			434341.922	19245029.703	100.0000

Translation: Chiralcel IC-H column [λ = 230 nm; eluent: Hexane/Isopropanol = 70/30; Flow rate: 0.5 mL/min; t_{minor} = 14.81 min, t_{major} = 21.12 min; ee% > 99%].

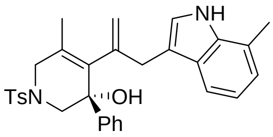

(S)-5-methyl-4-(3-(7-methyl-1H-indol-3-yl)prop-1-en-2-yl)-3-phenyl-1-tosyl-1,2,3,6-tetrahydropyridin-3-ol (5ag)

A light yellow oil. 66% yield (34 mg). ^{1}H NMR (400 MHz, $CDCl_3$, TMS) δ 1.67 (s, 3H), 2.42 (s, 3H), 2.43 (s, 3H), 2.87 (s, 1H), 2.96 (d, J = 11.2 Hz, 1H), 3.17 (d, J = 16.8 Hz, 1H), 3.27 (d, J = 16.8 Hz, 1H), 3.39 (d, J = 16.0 Hz, 1H), 3.47 (d, J = 11.2 Hz, 1H), 3.77 (d, J = 16.0 Hz, 1H), 4.60 (s, 1H), 4.83 (s, 1H), 6.78 (s, 1H), 6.87-6.95 (m, 3H), 7.27-7.35 (m, 5H), 7.50 (d, J = 7.2 Hz, 2H), 7.62 (d, J = 8.0 Hz, 2H), 7.89 (brs, 1H). ^{13}C NMR (100 MHz, $CDCl_3$, TMS) δ 16.5, 18.2, 21.5, 32.7, 49.5, 57.6, 72.9, 113.3, 117.1, 117.7, 119.2, 120.0, 122.2, 122.5, 126.6, 127.22, 127.75, 127.81, 128.1, 128.5, 129.8, 132.7, 135.8, 137.9, 142.0, 143.9, 144.5. IR (CH_2Cl_2): ν 3536, 3375, 3054, 2920, 2848, 2820, 1600, 1492, 1446, 1381, 1339, 1307, 1188, 1153, 1091, 1053, 1038, 984, 935, 899, 861, 816, 785, 767, 747, 703, 665 cm^{-1}. HRMS (ESI) calcd. for $C_{31}H_{36}N_3O_3S$ $(M+NH_4)^+$: 530.2472, Found: 530.2465. Enantiomeric excess was determined by HPLC with a Chiralcel IC-H column [λ = 230 nm; eluent: Hexane/Isopropanol = 70/30; Flow rate: 0.50 mL/min; t_{minor} = 14.19 min, t_{major} = 28.27 min; ee% = 99%; $[\alpha]^{20}_{D}$ = +48.0 (c 1.00, CH_2Cl_2)].

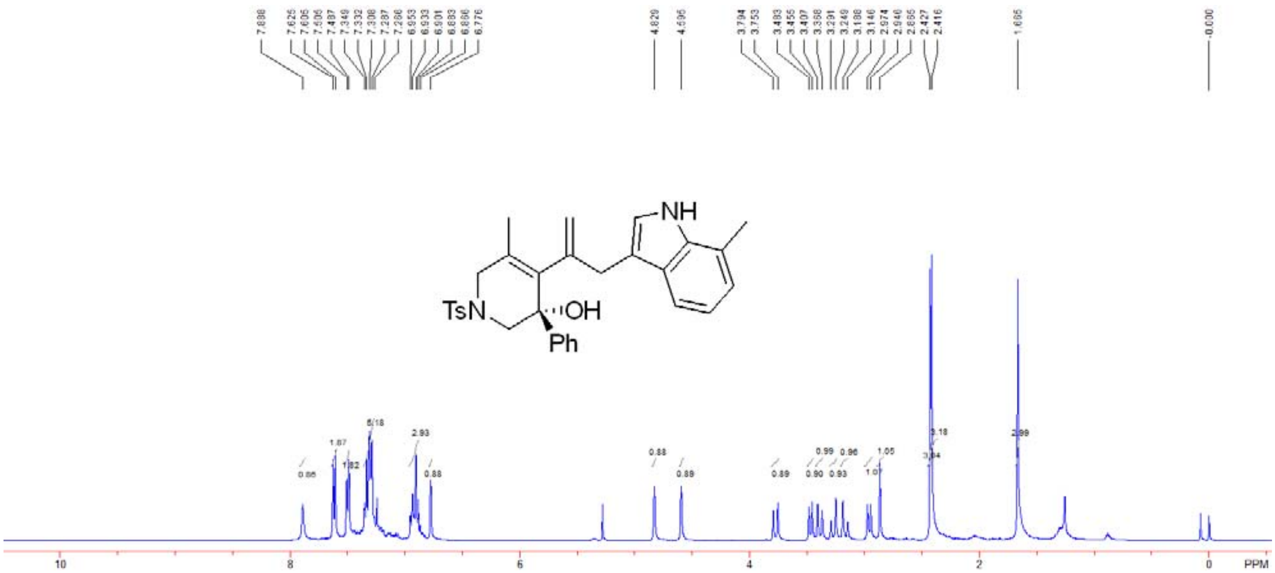

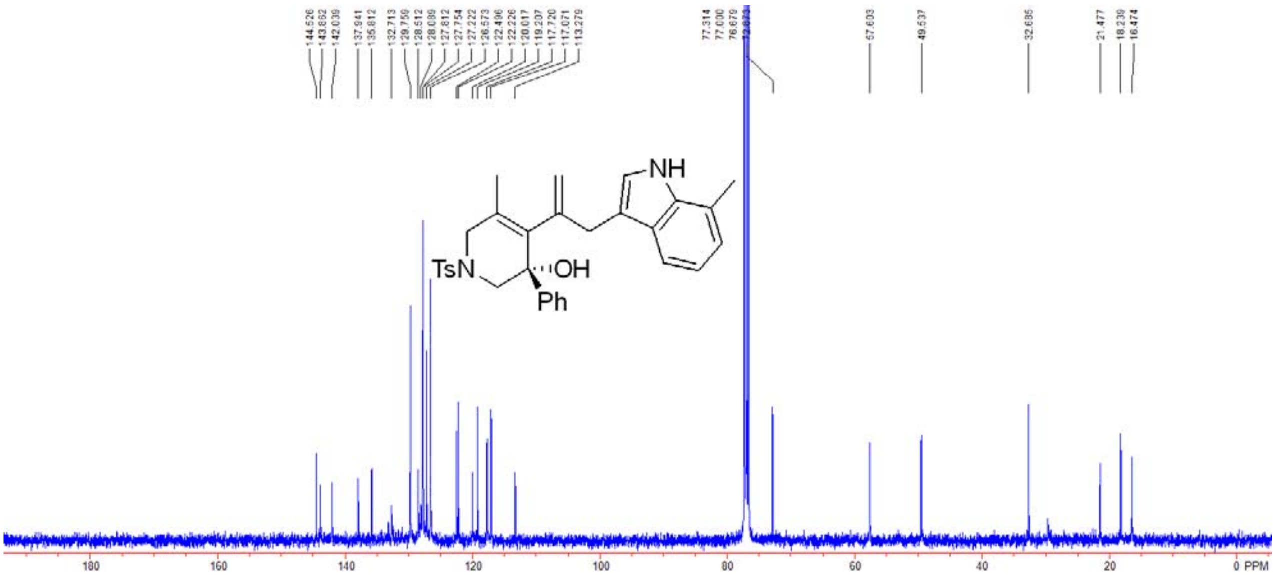

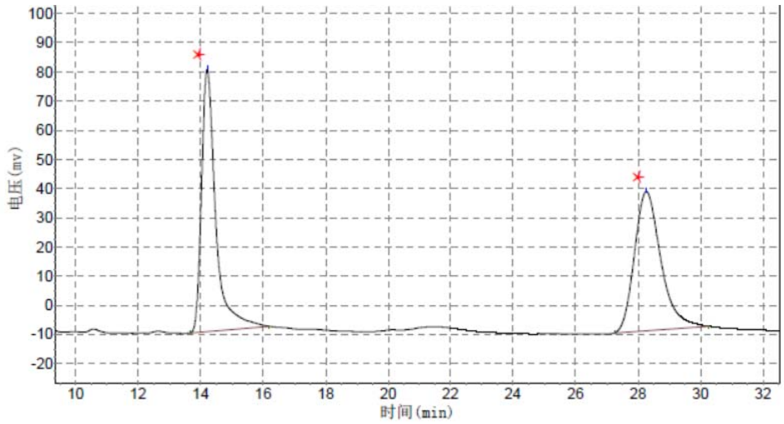

分析结果表

峰号	峰名	保留时间	峰高	峰面积	含量
1		14.213	89861.344	2836172.250	50.3772
2		28.260	47668.949	2793695.750	49.6228
总计			137530.293	5629868.000	100.0000

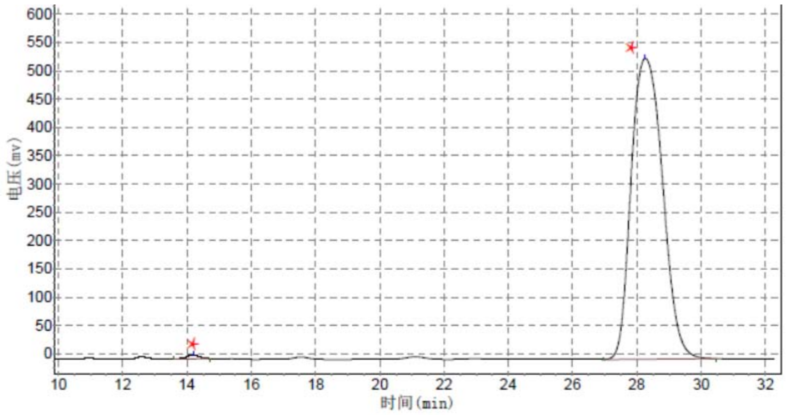

分析结果表

峰号	峰名	保留时间	峰高	峰面积	含量
1		14.190	6718.438	199551.344	0.5489
2		28.267	531147.625	36152904.000	99.4511
总计			537866.063	36352455.344	100.0000

Translation: Chiralcel IC-H column [λ = 230 nm; eluent: Hexane/Isopropanol = 80/20; Flow rate: 0.5 mL/min; t_{minor} = 14.19 min, t_{major} = 28.27 min; ee% = 99%].

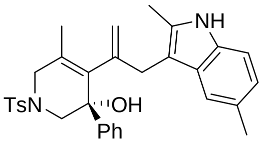

(S)-4-(3-(2,5-dimethyl-1H-indol-3-yl)prop-1-en-2-yl)-5-methyl-3-phenyl-1-tosyl-1,2,3,6-tetrahydropyridin-3-ol (5ah)

A light yellow oil. 82% yield (42 mg). ^{1}H NMR (400 MHz, $CDCl_3$, TMS) δ 1.82 (s, 3H), 1.99 (s, 3H), 2.33 (s, 3H), 2.43 (s, 3H), 2.89-2.94 (m, 2H), 3.02 (d, J = 11.6 Hz, 1H), 3.23 (d, J = 18.4 Hz, 1H), 3.46 (d, J = 16.0 Hz, 1H), 3.49 (d, J = 11.6 Hz, 1H), 3.84 (d, J = 16.0 Hz, 1H), 4.50 (d, J = 2.0 Hz, 1H), 4.54 (d, J = 1.2 Hz, 1H), 6.61 (s, 1H), 6.84 (dd, J_1 = 0.8 Hz, J_1 = 8.0 Hz, 1H), 7.06 (d, J = 8.0 Hz, 1H), 7.27-7.37 (m, 5H), 7.53 (d, J = 8.4 Hz, 2H), 7.60 (brs, 1H), 7.64 (d, J = 8.4 Hz, 2H). ^{13}C NMR (100 MHz, $CDCl_3$, TMS) δ 11.2, 18.2, 21.4, 21.5, 31.1, 49.5, 57.5, 72.8, 107.6, 109.6, 116.8, 117.8, 122.2, 126.6, 127.35, 127.80, 127.83, 128.0, 129.1, 129.8, 132.2, 132.6, 133.4, 138.6, 142.0, 143.8, 143.9. IR (CH_2Cl_2): ν 3397, 3023, 2917, 2851, 2360, 2342, 1628, 1597, 1448, 1378, 1345, 1305, 1154, 1091, 1040, 985, 862, 792, 766, 703 cm^{-1}. HRMS (ESI) calcd. for $C_{32}H_{38}N_3O_3S$ $(M+NH_4)^+$: 544.2628, Found: 544.2623. Enantiomeric excess was determined by HPLC with a Chiralcel AD-H column [λ = 230 nm; eluent: Hexane/Isopropanol = 80/20; Flow rate: 0.50 mL/min; t_{minor} = 69.24 min, t_{major} = 46.84 min; ee% = 97%; $[\alpha]^{20}_D$ = +40.8 (c 1.00, CH_2Cl_2)].

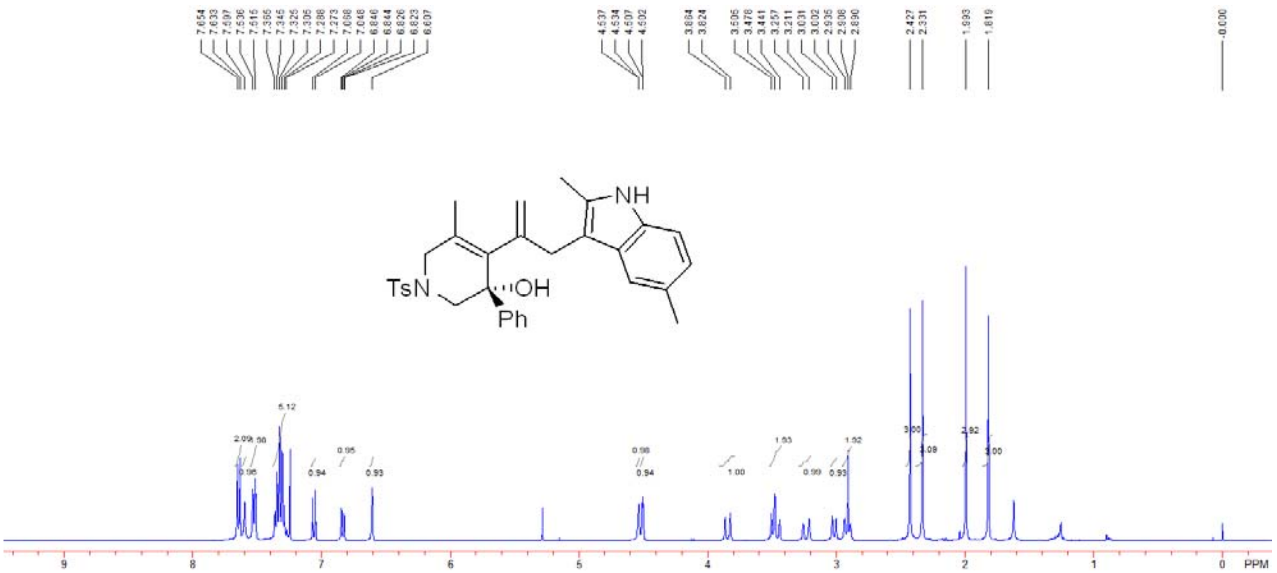

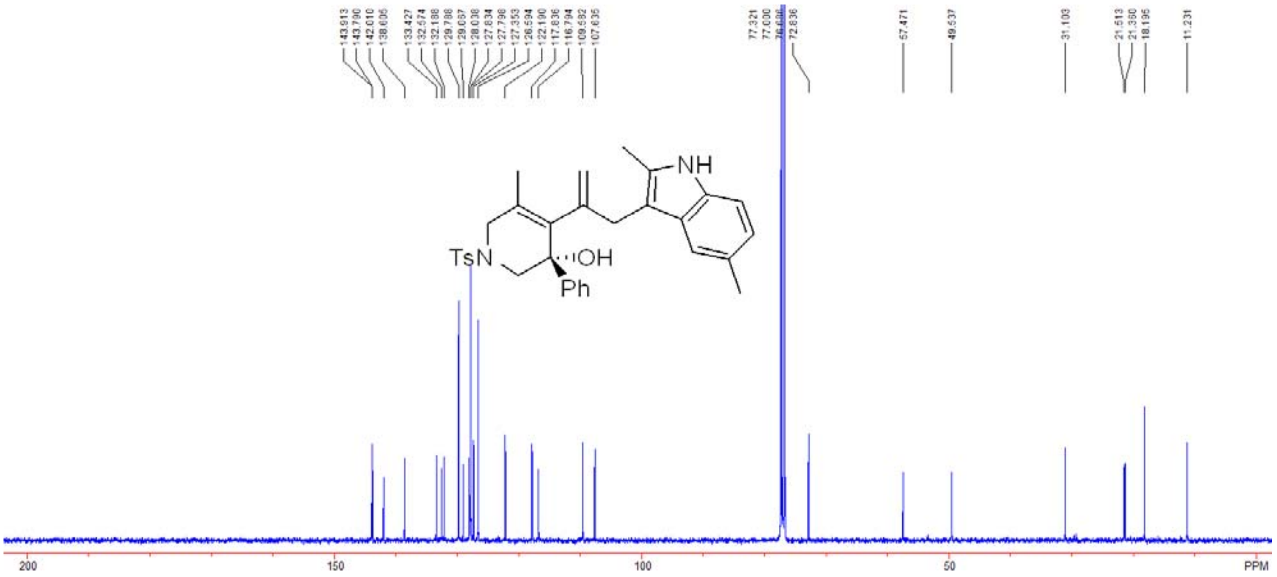

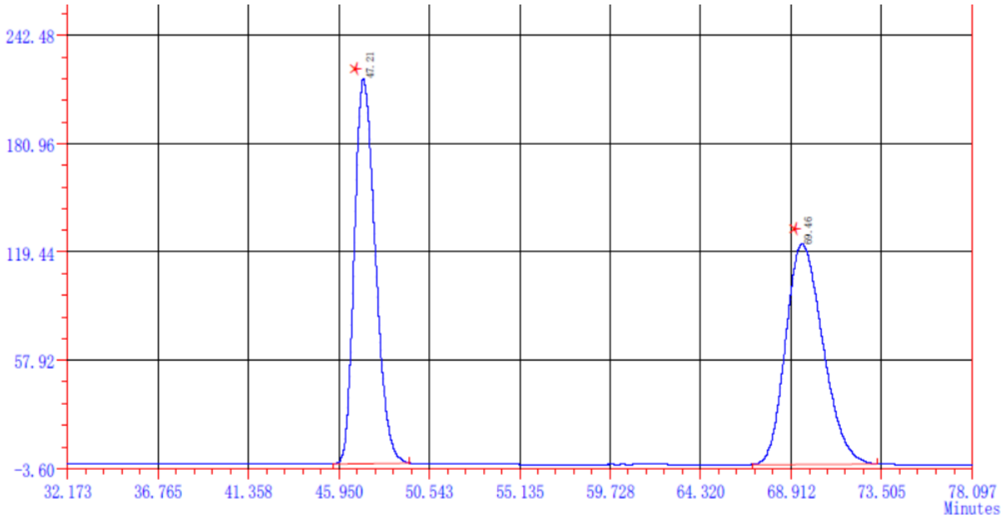

ID	组分名	保留时间	峰高	峰面积	浓度	拖尾因子	理论塔板
1		47.208	218686	16204267.1	49.5384	1.25	8090
2		69.463	125051	16506240.3	50.4616	1.23	5520
	Σ:		343737	32710507.4	100.0000		

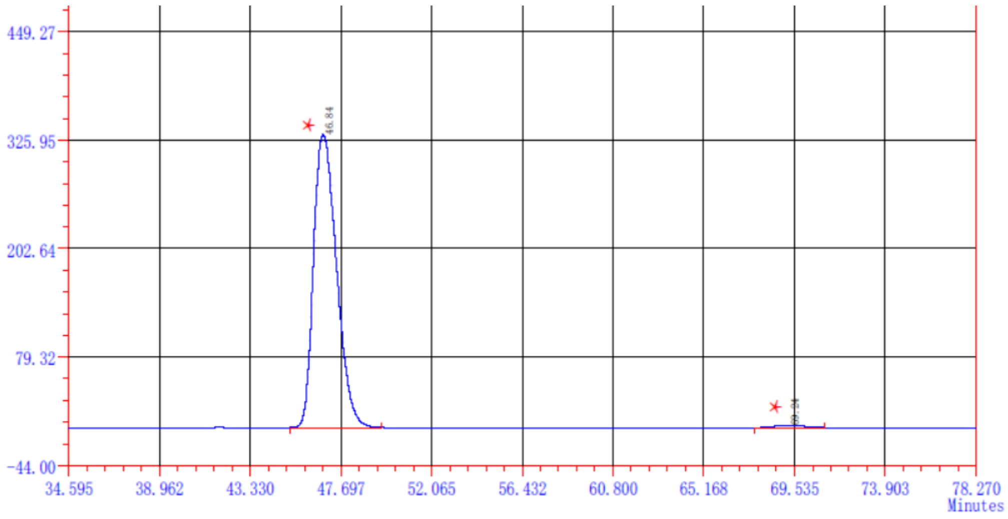

ID	组分名	保留时间	峰高	峰面积	浓度	拖尾因子	理论塔板
1		46.842	334059	25785297.7	98.6029	1.34	7340
2		69.243	3358	365353.8	1.3971	1.12	8073
	Σ:		337417	26150651.4	100.0000		

Translation: Chiralcel AD-H column [λ = 230 nm; eluent: Hexane/Isopropanol = 80/20; Flow rate: 0.5 mL/min; t_{minor} = 69.24 min, t_{major} = 46.84 min; ee% = 97%].

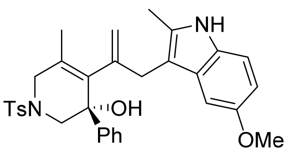

(S)-4-(3-(5-methoxy-2-methyl-1H-indol-3-yl)prop-1-en-2-yl)-5-methyl-3-phenyl-1-tosyl-1,2,3,6-tetrahydropyridin-3-ol (5ai)

A light yellow oil. 74% yield (40 mg). ^{1}H NMR (400 MHz, $CDCl_3$, TMS) δ 1.82 (s, 3H), 1.99 (s, 3H), 2.43 (s, 3H), 2.91-2.94 (m, 2H), 3.03 (d, J = 11.6 Hz, 1H), 3.20 (d, J = 18.4 Hz, 1H), 3.45-3.49 (m, 2H), 3.75 (s, 3H), 3.83 (d, J = 16.0 Hz, 1H), 4.54 (s, 2H), 6.48 (d, J = 2.0 Hz, 1H), 6.69 (dd, J_1 = 2.4 Hz, J_1 = 8.8 Hz, 1H), 7.08 (d, J = 8.8 Hz, 1H), 7.24-7.28 (m, 1H), 7.31-7.34 (m, 4H), 7.52 (d, J = 8.4 Hz, 2H), 7.61(brs, 1H), 7.64 (d, J = 8.4 Hz, 2H). ^{13}C NMR (100 MHz, $CDCl_3$, TMS) δ 11.3, 18.1, 21.5, 31.3, 49.5, 55.9, 57.5, 72.9, 100.6, 108.0, 110.3, 110.6, 116.9, 126.5, 127.29, 127.75, 127.79, 127.83, 129.4, 129.8, 130.3, 132.6, 133.0, 138.6, 142.1, 143.7, 143.9, 153.7. IR (CH_2Cl_2): ν 3398, 2990, 2956, 2920, 2845, 2829, 2360, 2342, 1595, 1484, 1449, 1341, 1305, 1248, 1219, 1156, 1104, 1091, 1034, 985, 917, 862, 805, 792, 767, 704 cm^{-1}. HRMS (ESI) calcd. for $C_{32}H_{38}N_3O_4S$ $(M+NH_4)^+$: 560.2578, Found: 560.2567. Enantiomeric excess was

determined by HPLC with a Chiralcel IC-H column [λ = 230 nm; eluent: Hexane/Isopropanol = 70/30; Flow rate: 0.50 mL/min; t_{minor} = 35.44 min, t_{major} = 31.72 min; ee% = 98%; $[\alpha]^{20}_{D}$ = +23.4 (c 1.00, CH_2Cl_2)].

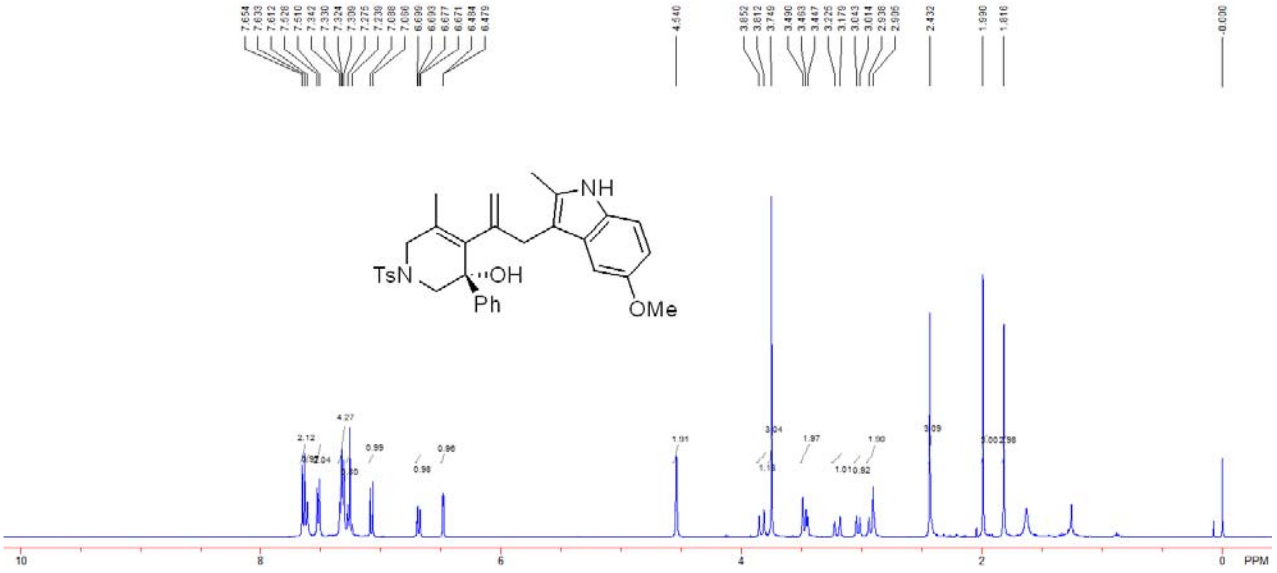

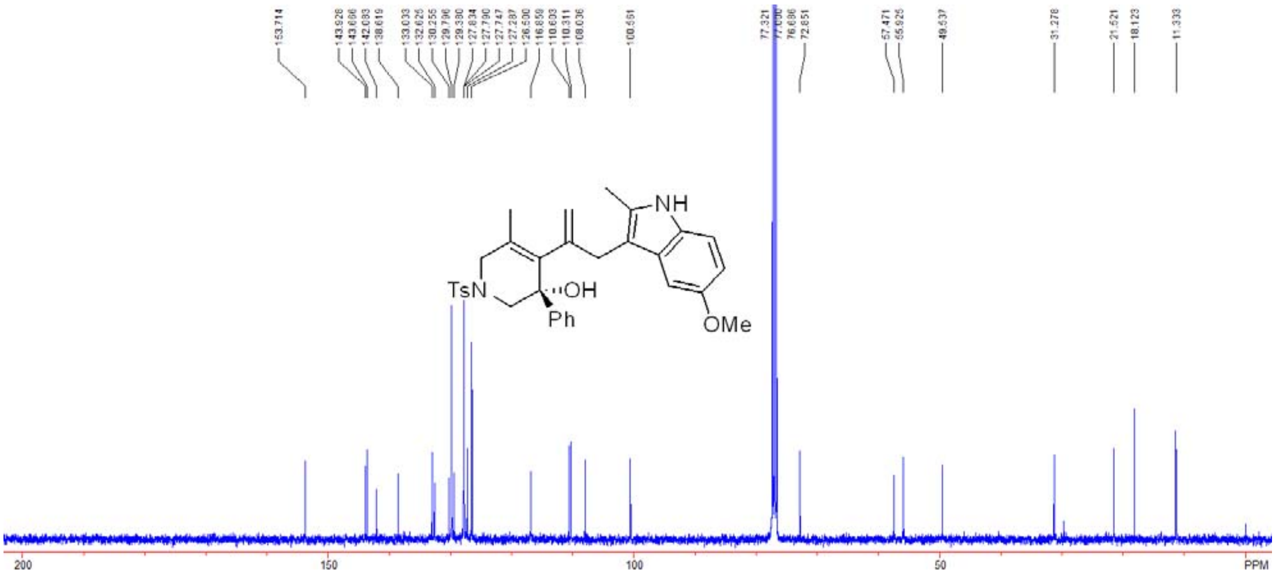

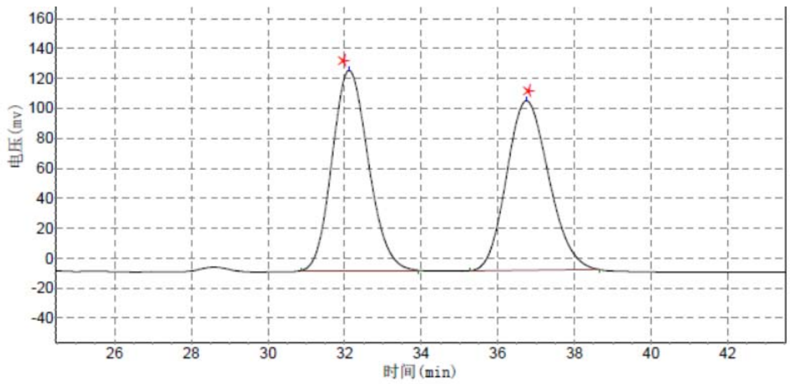

分析结果表

峰号	峰名	保留时间	峰高	峰面积	含量
1		32.123	133445.250	8743512.000	50.2604
2		36.737	113264.164	8652921.000	49.7396
总计			246709.414	17396433.000	100.0000

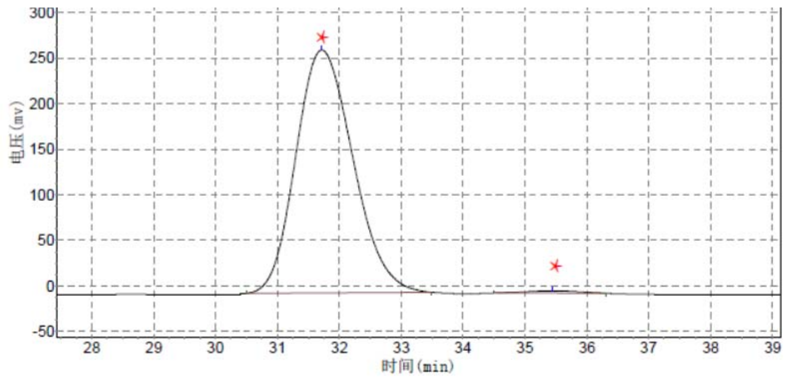

分析结果表

峰号	峰名	保留时间	峰高	峰面积	含量
1		31.717	267036.063	17134054.000	99.0909
2		35.442	2656.490	157201.734	0.9091
总计			269692.552	17291255.734	100.0000

Translation: Chiralcel IC-H column [λ = 230 nm; eluent: Hexane/Isopropanol = 70/30; Flow rate: 0.5 mL/min; t_{minor} = 35.44 min, t_{major} = 31.72 min; ee% = 98%].

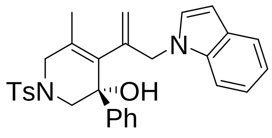

(S)-4-(3-(1H-indol-1-yl)prop-1-en-2-yl)-5-methyl-3-phenyl-1-tosyl-1,2,3,6-tetrahydropyridin-3-ol (6aa)

0.1 mmol scale. A white solid, 72% yield (36 mg). M. P. 100-102 °C. ^{1}H NMR ($CDCl_3$, 400 MHz, TMS) δ 1.71 (s, 3H), 2.43 (s, 3H), 2.85 (d, *J* = 11.2 Hz, 1H), 3.07 (s, 1H), 3.31 (d, *J* = 16.0 Hz, 1H), 3.66 (d, *J* = 11.2 Hz, 1H), 3.94 (d, *J* = 16.0 Hz, 1H), 4.29-4.33 (m, 2H), 4.54-4.59 (m, 2H),

6.39 (d, J = 2.8 Hz, 1H), 6.56-6.58 (m, 1H), 6.72 (d, J = 2.8 Hz, 1H) 6.99-7.01 (m, 2H), 7.31-7.39 (m, 5H), 7.48 (d, J = 8.4 Hz, 2H), 7.52-7.54 (m, 1H), 7.64 (d, J = 8.4 Hz, 2H). ^{13}C NMR ($CDCl_3$, 100 MHz, TMS) δ 18.2, 21.5, 49.7, 50.6, 57.7, 72.8, 101.2, 109.6, 117.3, 119.1, 120.6, 121.2, 126.5, 127.6, 127.8, 128.1, 128.2, 128.4, 129.9, 130.6, 132.3, 135.7, 136.1, 141.1, 141.2, 144.1. IR (CH_2Cl_2) ν 3363, 2973, 2923, 2900, 1646, 1598, 1510, 1484, 1449, 1397, 1345, 1316, 1191, 1170, 1091, 1046, 988, 918, 880, 806, 765, 742, 704, 665 cm^{-1}. HRMS (ESI) calcd. for $C_{30}H_{31}N_2O_3S$ $(M+H)^+$: 499.2050, Found: 499.2043. Enantiomeric excess was determined by HPLC with a Chiralcel IC-H column [λ = 230 nm; eluent: Hexane/Isopropanol = 80/20; Flow rate: 0.50 mL/min; t_{minor} = 40.57 min, t_{major} = 44.43 min; ee% > 99%; $[\alpha]^{20}_D$ = +44.2 (c 1.00, CH_2Cl_2)].

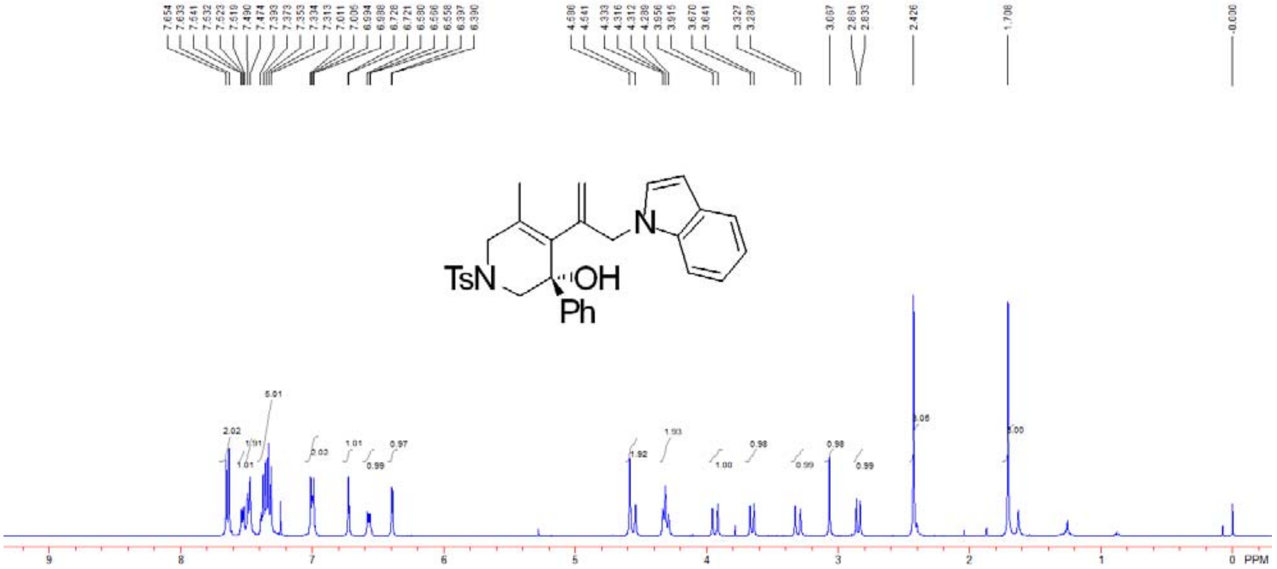

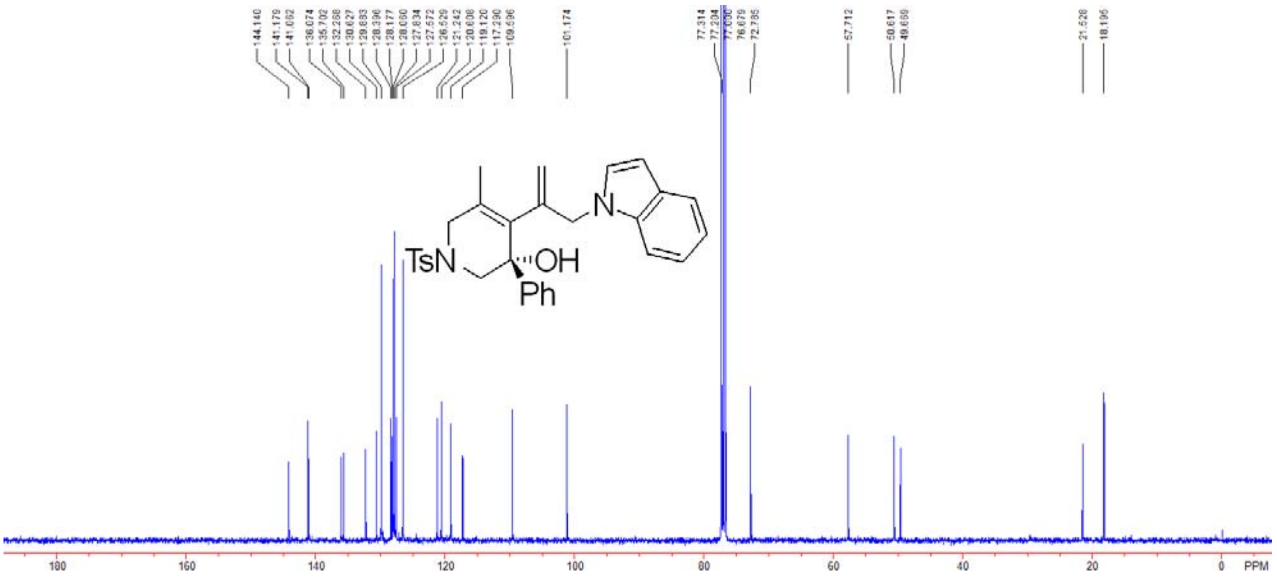

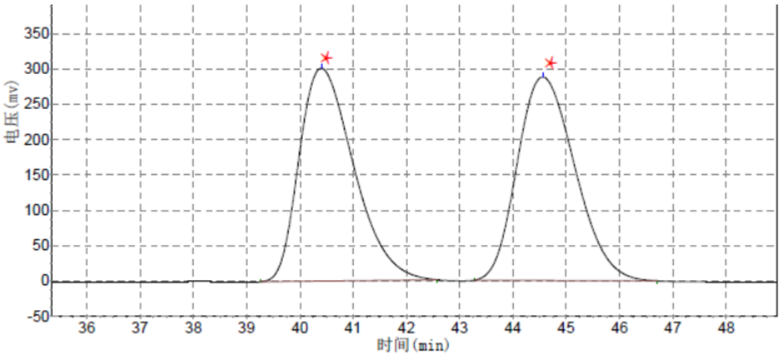

分析结果表

峰号	峰名	保留时间	峰高	峰面积	含量
1		40. 405	301359. 094	21451964. 000	49. 7204
2		44. 560	287995. 750	21693232. 000	50. 2796
总计			589354. 844	43145196. 000	100. 0000

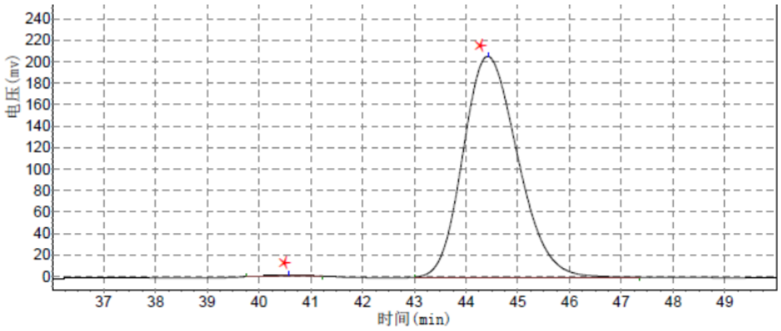

分析结果表

峰号	峰名	保留时间	峰高	峰面积	含量
1		40. 567	1338. 166	73021. 000	0. 4805
2		44. 425	205837. 141	15123479. 000	99. 5195
总计			207175. 307	15196500. 000	100. 0000

Translation: Chiralcel IC-H column [λ = 230 nm; eluent: Hexane/Isopropanol = 80/20; Flow rate: 0.5 mL/min; t_{minor} = 40.57 min, t_{major} = 44.43 min; ee% > 99%].

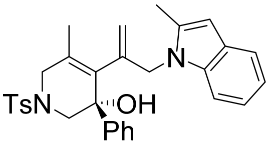

(S)-5-methyl-4-(3-(2-methyl-1H-indol-1-yl)prop-1-en-2-yl)-3-phenyl-1-tosyl-1,2,3,6-tetrahydropyridin-3-ol (6ab)

0.1 mmol scale. A white solid, 76% yield (39 mg). M. P. 124-126 °C. ^{1}H NMR ($CDCl_3$, 400 MHz, TMS) δ 1.87 (s, 3H), 1.91 (s, 3H), 2.42 (s, 3H), 2.81 (d, *J* = 11.6 Hz, 1H), 3.15 (s, 1H), 3.35 (d, *J* = 16.0 Hz, 1H), 3.68 (d, *J* = 11.6 Hz, 1H), 3.95 (s, 1H), 4.02 (d, *J* = 16.0 Hz, 1H), 4.21 (d, *J* = 18.8 Hz, 1H), 4.51 (s, 1H), 4.56 (d, *J* = 18.8 Hz, 1H), 6.13 (s, 1H), 6.47 (brs, 1H), 6.90-6.98 (m, 2H), 7.29-7.37 (m, 5H), 7.42 (d, *J* = 7.6 Hz, 1H), 7.48 (d, *J* = 7.2 Hz, 2H), 7.66 (d, *J* = 8.4 Hz, 2H). ^{13}C

NMR ($CDCl_3$, 125 MHz, TMS) δ 11.8, 18.4, 21.5, 47.2, 49.8, 57.9, 72.8, 99.7, 108.9, 116.2, 119.1, 119.3, 120.2, 126.6, 127.6, 127.8, 128.0, 129.9, 130.4, 132.3, 136.08, 136.60, 136.63, 140.9, 141.1, 144.1. IR (CH_2Cl_2) ν 3498, 3088, 3057, 3021, 2917, 2848, 1598, 1554, 1494, 1479, 1448, 1398, 1349, 1309, 1251, 1188, 1169, 1154, 1120, ,1092, 1039, 987, 919, 863, 839, 808, 748, 766, 704, 663 cm^{-1}. HRMS (ESI) calcd. for $C_{31}H_{33}N_2O_3S$ $(M+NH_4)^+$: 513.2206, Found: 513.2199. Enantiomeric excess was determined by HPLC with a Chiralcel IC-H column [λ = 230 nm; eluent: Hexane/Isopropanol = 80/20; Flow rate: 0.50 mL/min; t_{minor} = 54.08 min, t_{major} = 40.36 min; ee% > 99%; $[\alpha]^{20}_D$ = +40.4 (c 1.00, CH_2Cl_2)].

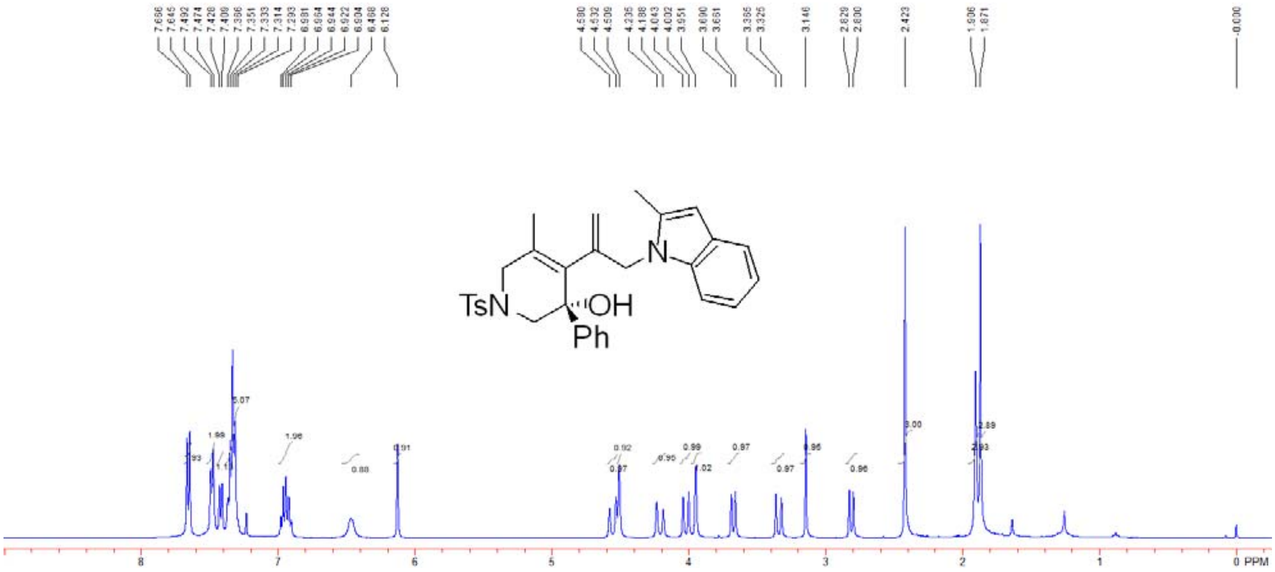

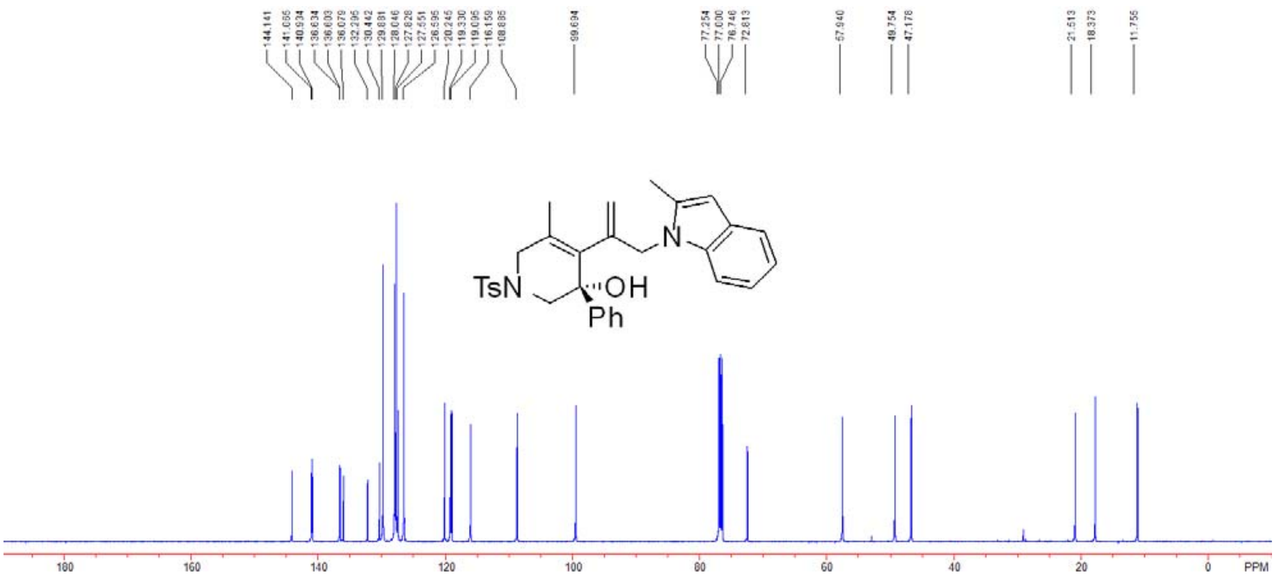

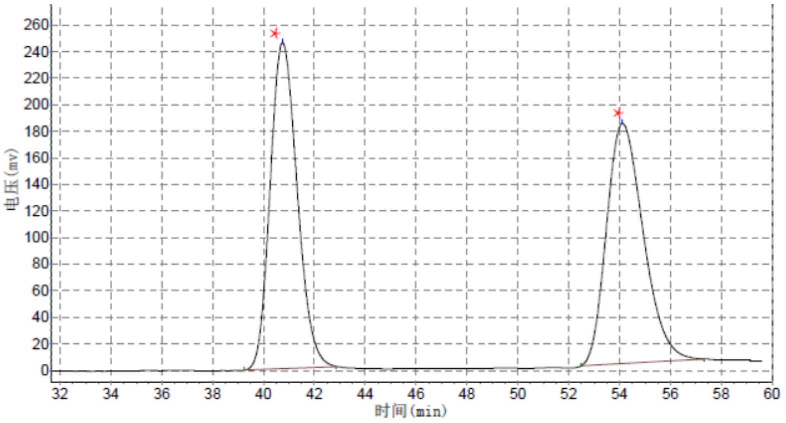

分析结果表

峰号	峰名	保留时间	峰高	峰面积	含量
1		40.752	245739.188	18119912.000	49.9582
2		54.115	180768.953	18150198.000	50.0417
总计			426508.141	36270110.000	100.0000

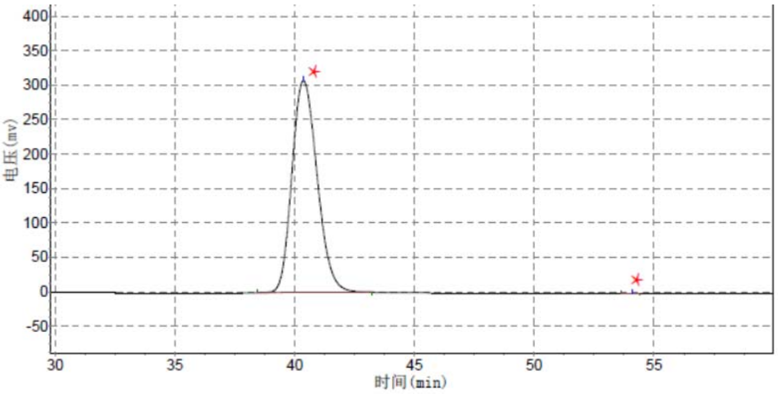

分析结果表

峰号	峰名	保留时间	峰高	峰面积	含量
1		40.360	307327.906	22530566.000	99.9927
2		54.083	59.813	1656.100	0.0073
总计			307387.720	22532222.100	100.0000

Translation: Chiralcel IC-H column [λ = 230 nm; eluent: Hexane/Isopropanol = 80/20; Flow rate: 0.5 mL/min; t_{minor} = 54.08 min, t_{major} = 40.36 min; ee% > 99%].

11. Transformations of products 3aa and 6aa

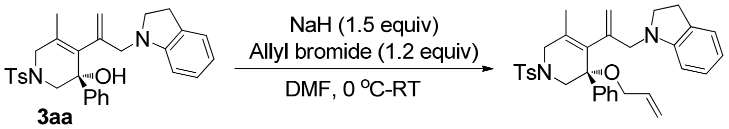

7aa, 91% yield

To a flame dried Schlenk tube was added compounds **3aa** (0.2 mmol), NaH (60% dispersion in mineral oil, 1.5 equiv) and DMF (2.0 mL). The reaction mixture was stirred at 0 °C for 0.5 h before allyl bromide (1.2 equiv) was added. The reaction mixture was stirred at 0 °C for another 4

h. Then, the reaction mixture was diluted with cold water and was extracted with ether (4 mL x 3) and the combined organics was dried over anhydrous Na_2SO_4. The solvent was removed under reduced pressure and the residue was purified by a flash column chromatography (SiO_2) to give the corresponding product **7aa**.

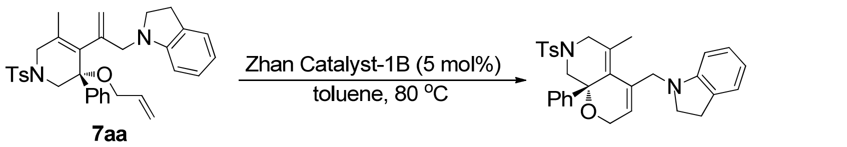

Under argon atmosphere, compound **7aa** (0.1 mmo, 1.0 equiv), Zhan-catalyst-1B (0.10 equiv) toluene (10 mL) were added and then the mixture was heated at 80 °C for 12 h. Then, the solvent was removed under reduced pressure and the residue was purified by a flash column chromatography (SiO_2) to give the corresponding product **8aa**.

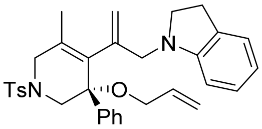

(S)-1-(2-(3-(allyloxy)-5-methyl-3-phenyl-1-tosyl-1,2,3,6-tetrahydropyridin-4-yl)allyl)indoline (7aa)

0.2 mmol scale. A colorless oil, 91% yield (98 mg). ^{1}H NMR ($CDCl_3$, 400 MHz, TMS) δ 1.85 (s, 3H), 2.44 (s, 3H), 2.88 (dd, J_1 = 8.4 Hz, J_1 = 8.4 Hz, 2H), 3.01 (d, J = 18.0 Hz, 1H), 3.08 (d, J = 12.4 Hz, 1H), 3.13 (dd, J_1 = 8.8 Hz, J_1 = 17.2 Hz, 1H), 3.22 (dd, J_1 = 8.8 Hz, J_1 = 17.2 Hz, 1H), 3.34 (d, J = 16.0 Hz, 1H), 3.42 (d, J = 18.0 Hz, 1H), 3.84 (d, J = 12.4 Hz, 1H), 3.90 (d, J = 16.0 Hz, 1H), 4.24-4.29 (m, 1H), 4.41-4.45 (m, 1H), 4.56 (d, J = 1.2 Hz, 1H), 5.10 (d, J = 2.0 Hz, 1H), 5.17 (dd, J_1 = 1.6 Hz, J_2 = 10.8 Hz, 1H), 5.40 (dd, J_1 = 1.6 Hz, J_2 = 17.2 Hz, 1H), 5.85 (s, 1H), 5.87 (s, 1H), 5.95-6.04 (m, 1H), 6.53 (dd, J_1 = 7.2 Hz, J_2 = 7.6 Hz, 1H), 6.88 (dd, J_1 = 7.6 Hz, J_2 = 7.6 Hz, 1H), 6.97 (d, J = 7.6 Hz, 1H), 7.22-7.31 (m, 3H), 7.33 (d, J = 8.0 Hz, 2H), 7.45 (d, J = 6.8 Hz, 2H), 7.64 (d, J = 8.4 Hz, 2H). ^{13}C NMR ($CDCl_3$, 125 MHz, TMS) δ 18.5, 21.5, 28.4, 49.3, 53.2, 53.7, 54.3, 65.1, 77.8, 106.1, 115.5, 116.1, 116.6, 124.0, 127.0, 127.1, 127.3, 127.8, 127.9, 129.0, 129.8, 131.2, 132.0, 133.4, 135.2, 140.4, 141.9, 143.9, 152.1. IR (CH_2Cl_2) ν 2972, 2923, 2897, 1606, 1487, 1447, 1405, 1380, 1346, 1257, 1208, 1167, 1090, 1047, 981, 957, 920, 880, 809, 768, 746, 7802, 660 cm^{-1}. HRMS (ESI) calcd. for $C_{33}H_{37}N_2O_3S$ $(M+H)^+$: 541.2519, Found:

541.2508. $[\alpha]^{20}_{D}$ = +30.2 (c 1.00, CH_2Cl_2)].

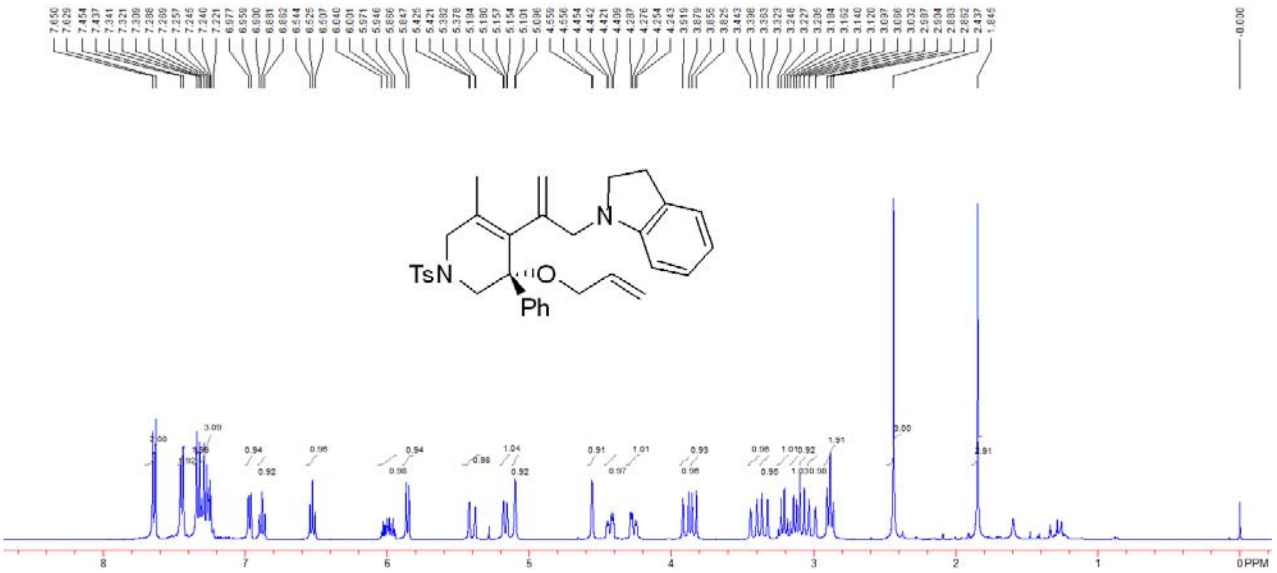

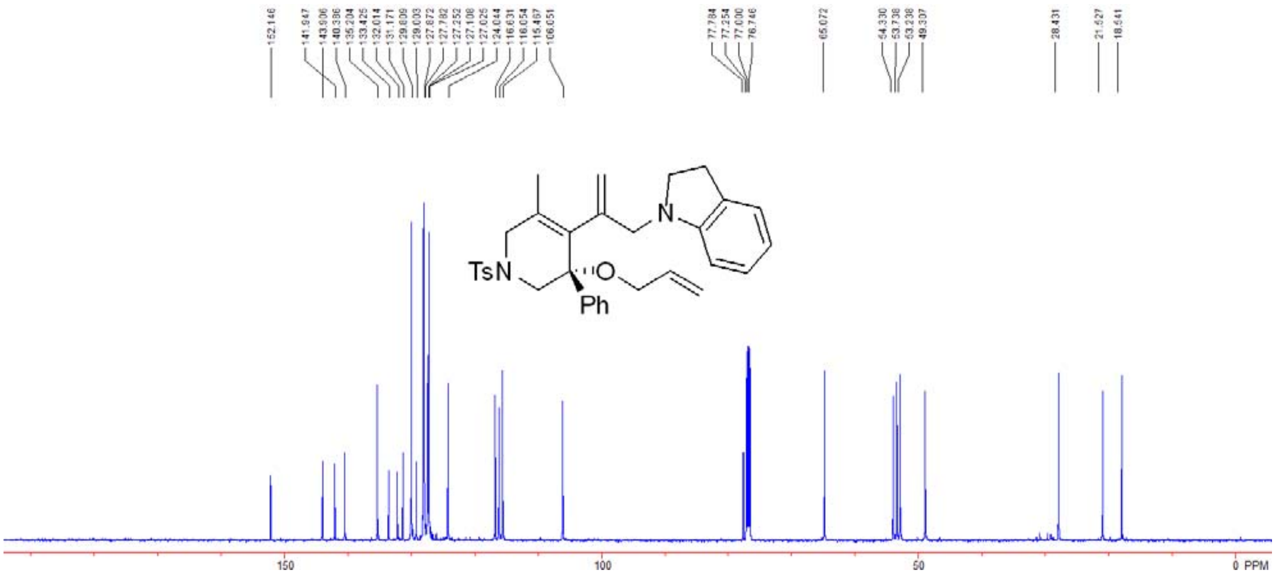

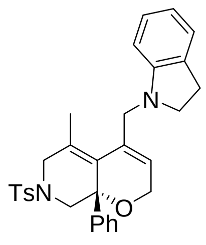

(S)-4-(indolin-1-ylmethyl)-5-methyl-8a-phenyl-7-tosyl-6,7,8,8a-tetrahydro-2H-pyrano[2,3-c]pyridine (8aa)

0.1 mmol scale. A white solid, 76% yield (39 mg). M. P. 90-92 °C. ^{1}H NMR ($CDCl_3$, 400 MHz, TMS) δ 1.98 (s, 3H), 2.39 (s, 3H), 2.76 (d, *J* = 10.8 Hz, 1H), 2.82-2.95 (m, 2H), 3.06-3.17 (m, 2H),

3.27 (d, J = 17.2 Hz, 1H), 3.79-3.85 (m, 2H), 4.00 (d, J = 16.0 Hz, 2H), 4.07 (d, J = 18.0 Hz, 1H), 4.20 (d, J = 18.0 Hz, 1H), 5.65 (s, 1H), 6.30 (d, J = 8.0 Hz, 1H), 6.63 (dd, J_1 = 6.8 Hz, J_2 = 7.2 Hz, 1H), 6.96 (dd, J_1 = 7.2 Hz, J_2 = 7.6 Hz, 1H), 7.05 (d, J = 7.2 Hz, 1H), 7.20 (d, J = 8.0 Hz, 2H), 7.28-7.33 (m, 5H), 7.44 (d, J = 8.4 Hz, 2H). ^{13}C NMR ($CDCl_3$, 125 MHz, TMS) δ 18.7, 21.5, 28.4, 51.3, 53.9, 54.8, 55.5, 61.9, 76.0, 106.8, 117.7, 124.4, 126.4, 127.16, 127.22, 127.38, 127.49, 127.63, 127.74, 127.95, 129.5, 129.9, 130.0, 133.2, 140.5, 143.5, 152.0. IR (CH_2Cl_2) ν 3060, 3021, 2920, 2828, 2161, 1980, 1606, 1489, 1448, 1351, 1304, 1253, 1159, 1131, 1089, 1056, 1010, 958, 902, 813, 747, 704 cm^{-1}. HRMS (ESI) calcd. for $C_{31}H_{33}N_2O_3S$ $(M+H)^+$: 513.2206, Found: 513.2211. $[\alpha]^{20}_D$ = +37.0 (c 1.00, CH_2Cl_2)]. Enantiomeric excess was determined by HPLC with a Chiralcel IC-H column [λ = 230 nm; eluent: Hexane/Isopropanol = 80/20; Flow rate: 0.50 mL/min; t_{minor} = 37.50 min, t_{major} = 52.10 min; ee% > 99%; $[\alpha]^{20}_D$ = -105.1 (c 1.00, CH_2Cl_2)].

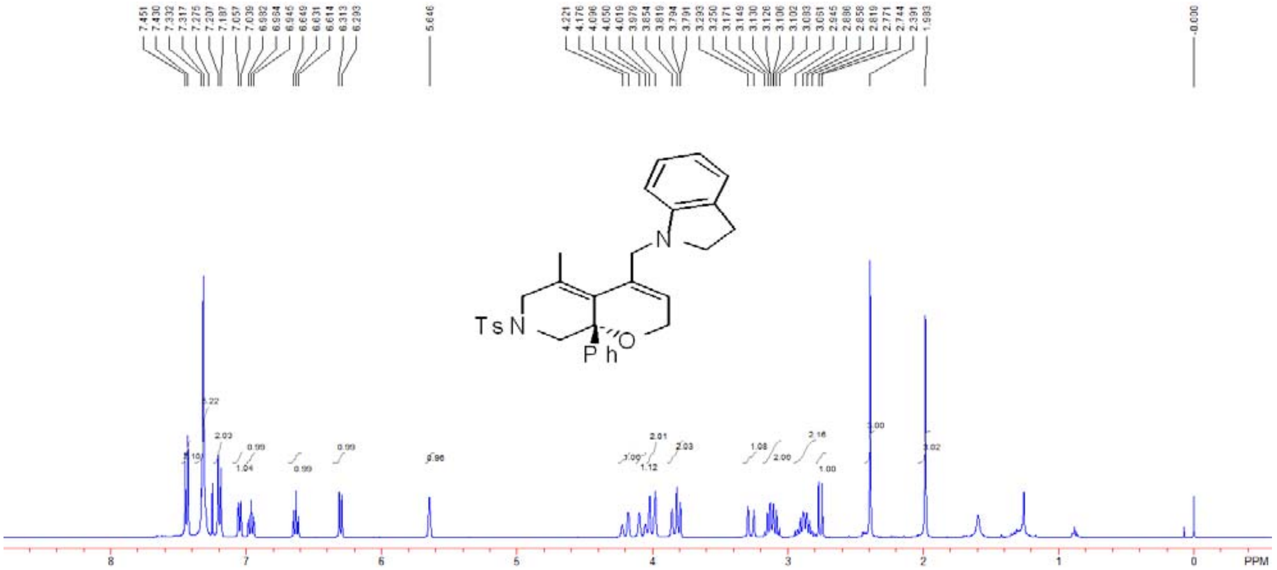

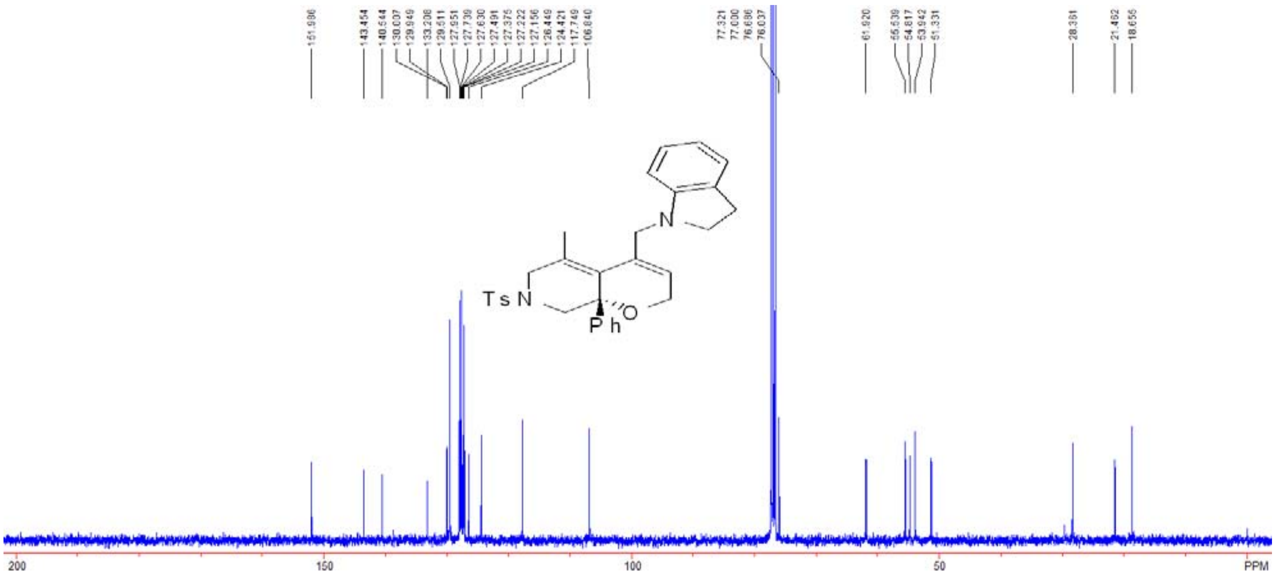

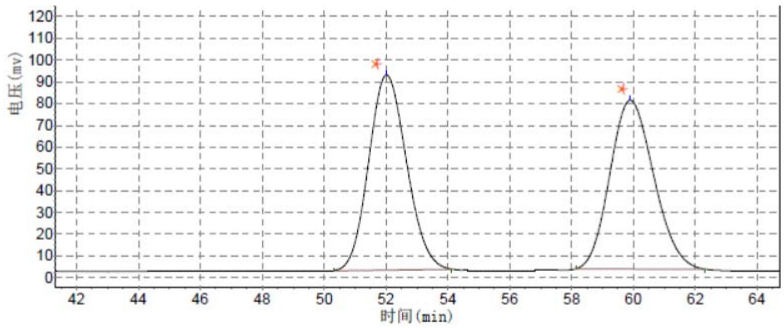

分析结果表

峰号	峰名	保留时间	峰高	峰面积	含量
1		52.022	89694.672	7611979.000	49.9770
2		59.907	77580.680	7618980.000	50.0230
总计			167275.352	15230959.000	100.0000

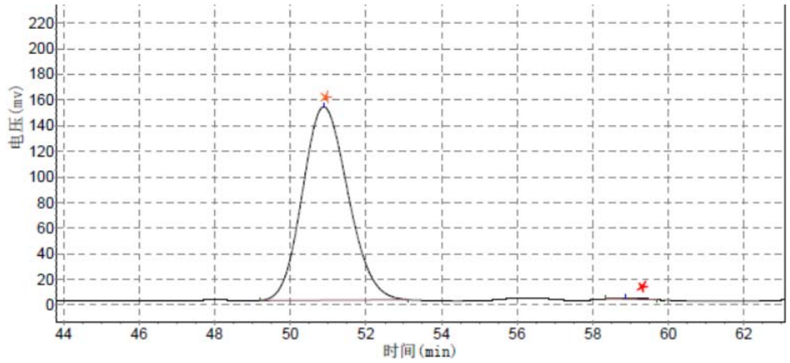

分析结果表

峰号	峰名	保留时间	峰高	峰面积	含量
1		50.882	150816.031	12597842.000	99.6049
2		58.878	968.400	49966.570	0.3951
总计			151784.431	12647808.570	100.0000

Translation: Chiralcel IC-H column [λ = 254 nm; eluent: Hexane/Isopropanol = 80/20; Flow rate: 0.5 mL/min; t_{minor} = 37.50 min, t_{major} = 52.10 min; ee% > 99%].

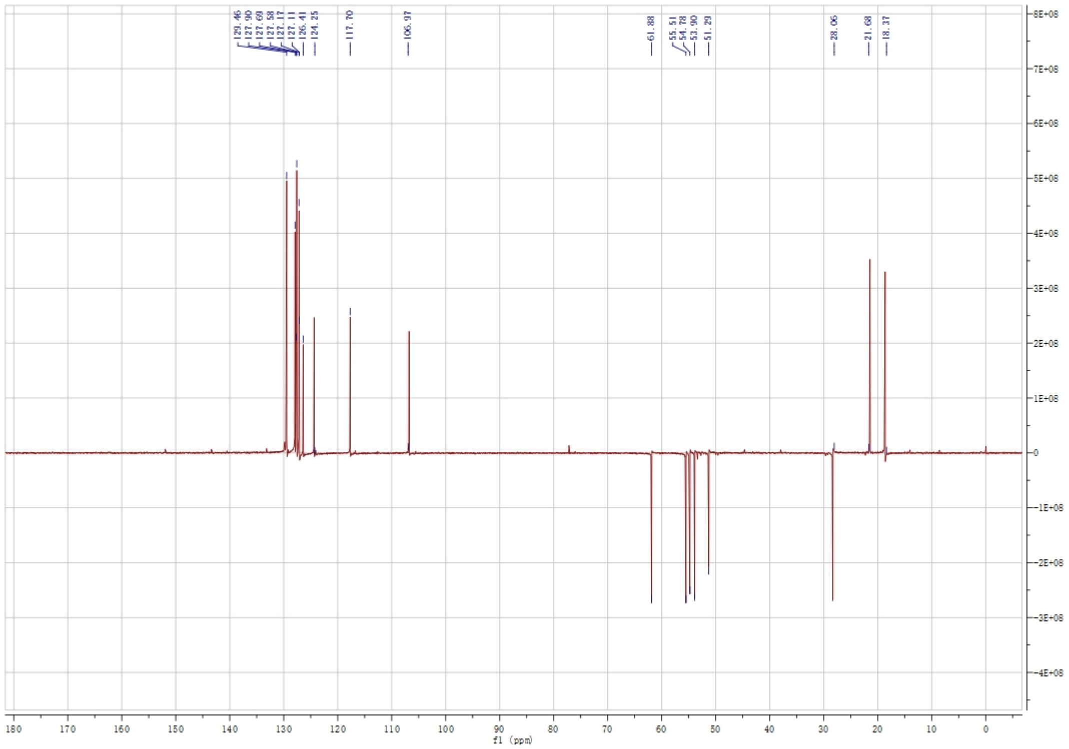

Distortionless Enhancement by Polarization Transfer (DEPT)

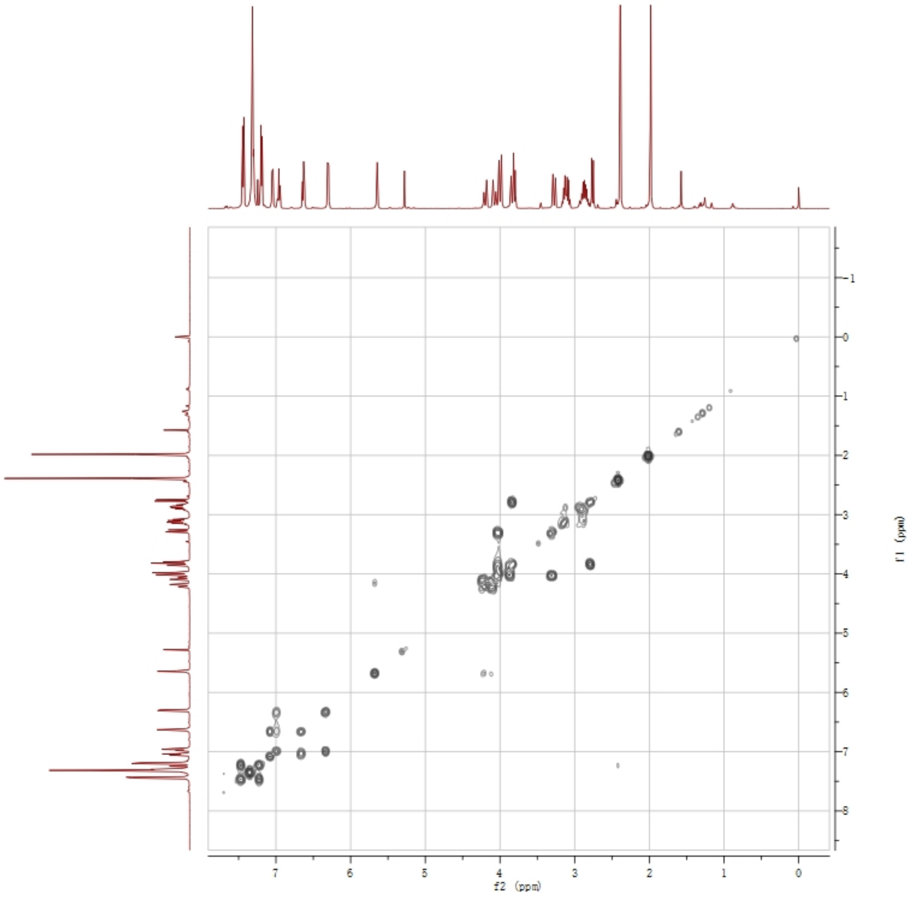

Correlation Spectroscopy (COSY)

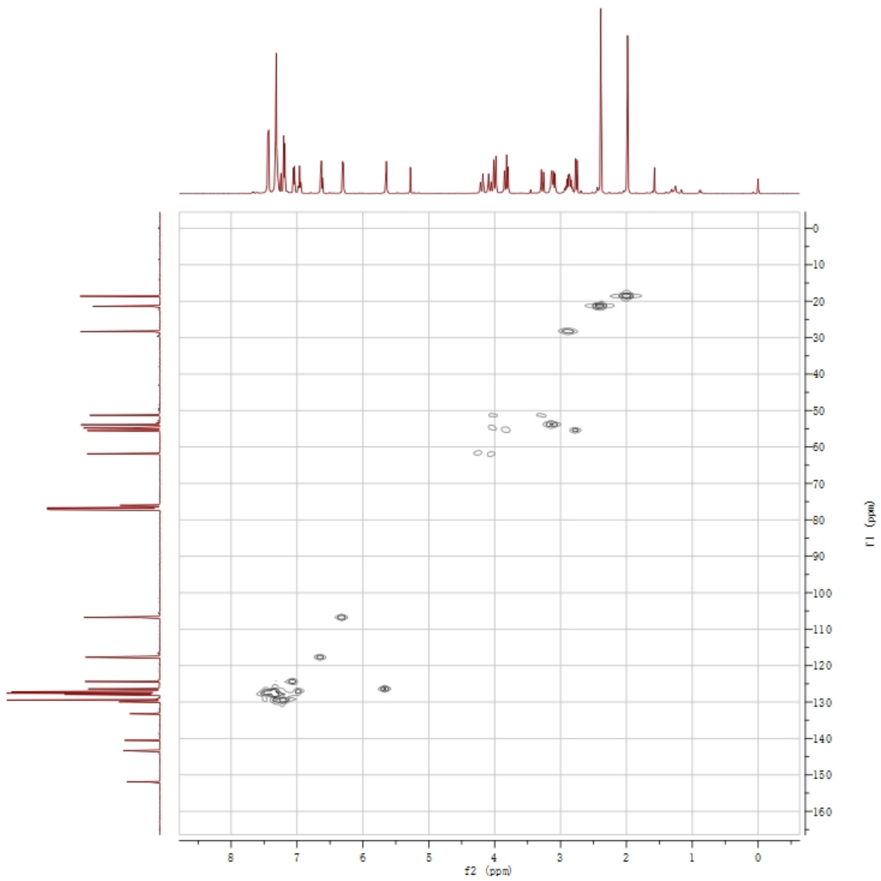

Heteronuclear Multiple-Quantum Correlation (HMQC)

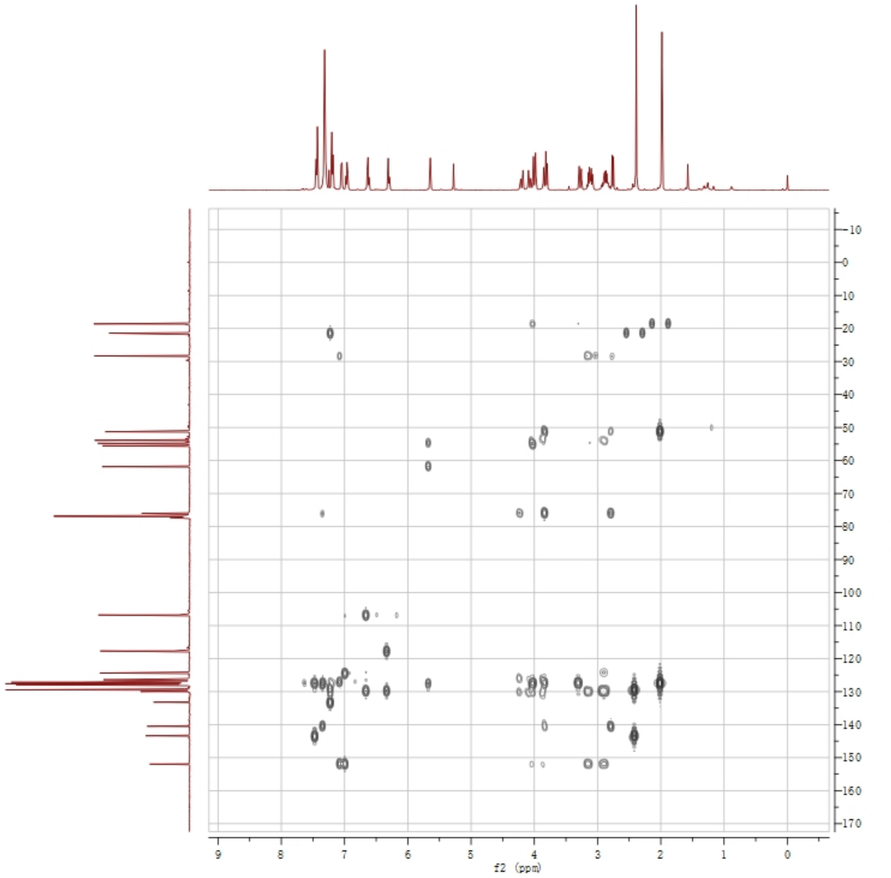

Heteronuclear Multiple Bond Correlation (HMBC)

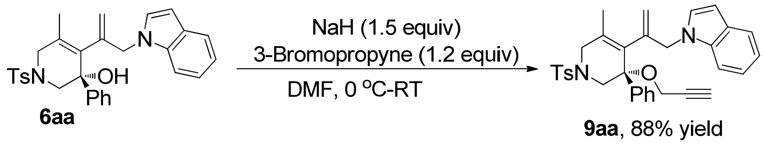

To a flame dried Schlenk tube was added compunds **6aa** (0.2 mmol), NaH (60% dispersion in mineral oil, 1.5 equiv) and DMF (2.0 mL). The reaction mixture was stirred at 0 °C for 0.5 h before 3-bromopropyne bromide (1.2 equiv) was added. The reaction mixture was stirred at 0 °C for another 4 h. Then, the reaction mixture was diluted with cold water and was extracted with ether (4 mL x 3) and the combined organics was dried over anhydrous Na_2SO_4. The solvent was removed under reduced pressure and the residue was purified by a flash column chromatography (SiO_2) to give the corresponding product **9aa**.

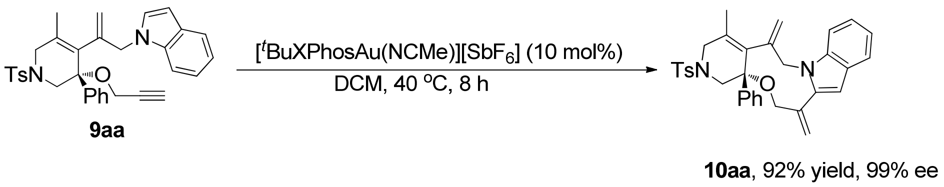

To a flame dried Schlenk tube was added **9aa** (0.1 mmol, 1.0 equiv), [Au(*t*BuXPhos)(NCMe)][SbF_6] (10 mol %) and DCM (2.0 mL). The reaction mixture was stirred at 10 °C for 8 h. The solvent was removed under reduced pressure and the residue was purified by a flash column chromatography (SiO_2) to give the corresponding product **10aa**.

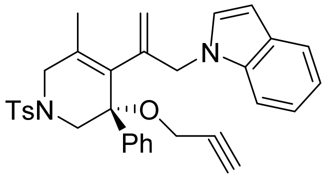

(S)-1-(2-(5-methyl-3-phenyl-3-(prop-2-yn-1-yloxy)-1-tosyl-1,2,3,6-tetrahydropyridin-4-yl)allyl)-1H-indole (9aa)

0.2 mmol scale. A white solid, 88% yield (94 mg). M. P. 94-96 °C. ^{1}H NMR ($CDCl_3$, 400 MHz, TMS) δ 1.78 (s, 3H), 2.43 (t, J = 2.4 Hz, 1H), 2.45 (s, 3H), 3.13 (d, J = 12.4 Hz, 1H), 3.36 (d, J = 16.1 Hz, 1H), 3.82-3.90 (m, 2H), 4.21-4.26 (m, 2H), 4.45 (s, 1H), 4.51 (dd, J_1 = 2.4 Hz, J_2 = 15.2 Hz, 1H), 4.57-4.64 (m, 2H), 6.41 (d, J = 3.2 Hz, 1H), 8.79-8.82 (m, 1H), 7.00-7.07 (m, 2H), 7.30-7.37 (m, 5H), 7.50 (d, J = 7.2 Hz, 2H), 7.54 (dd, J_1 = 2.0 Hz, J_2 = 6.4 Hz, 1H), 7.65 (d, J = 8.1 Hz, 2H). ^{13}C NMR ($CDCl_3$, 125 MHz, TMS) δ 18.5, 21.6, 49.3, 50.5, 53.2, 54.3, 73.8, 80.4, 101.2, 109.7, 117.2, 119.2, 120.6, 121.3, 127.0, 127.7, 127.9, 128.08, 128.15, 128.49, 129.9, 131.8, 133.0, 136.1, 140.7, 141.0, 144.1. IR (CH_2Cl_2) ν 3281, 3089, 3052, 3022, 2920, 2856, 2808, 1729, 1598, 1511, 1485, 1463, 1447, 1398, 1342, 1315, 1259, 1162, 1091, 1066, 1020, 986, 917, 870, 811, 764, 741, 704, 662 cm^{-1}. HRMS (ESI) calcd. for $C_{33}H_{33}N_2O_3S$ $(M+H)^+$: 537.2206, Found: 537.2200. $[\alpha]^{20}_D$ = +35.3 (c 1.00, CH_2Cl_2)].

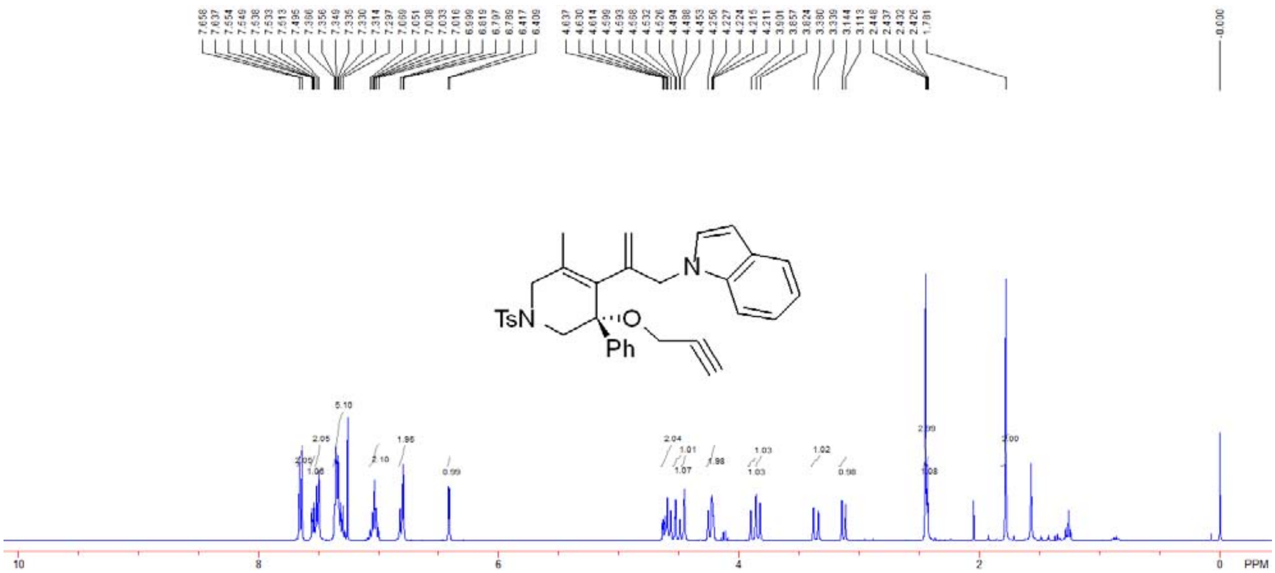

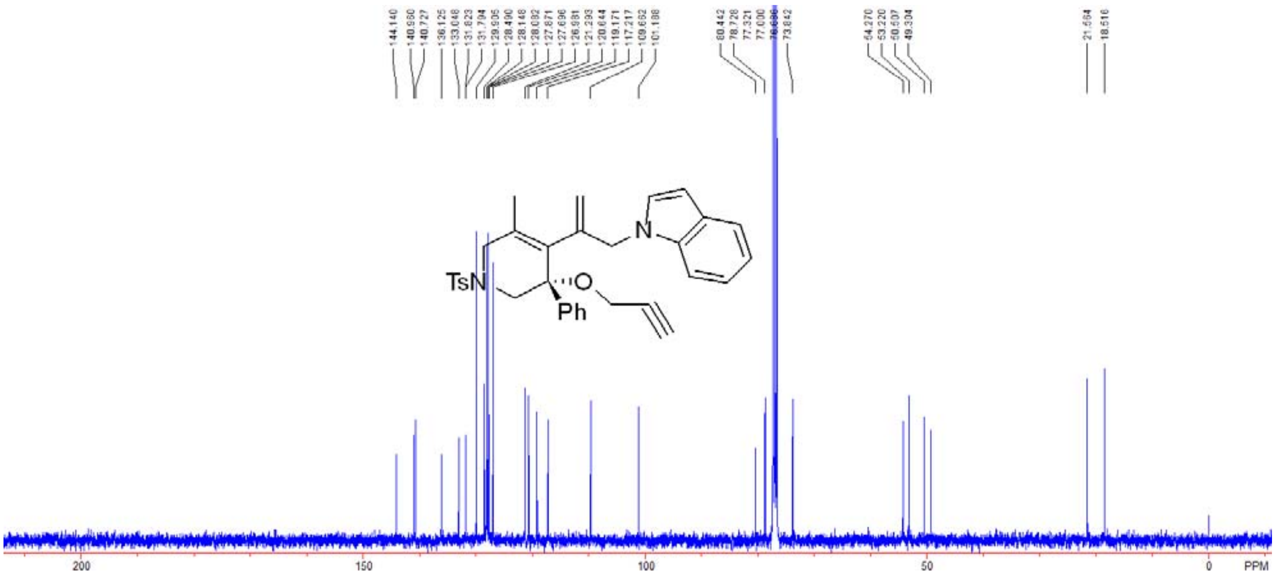

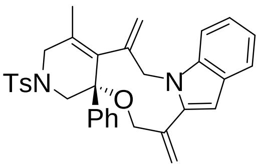

(S)-4-methyl-5,13-dimethylene-15a-phenyl-2-tosyl-1,2,3,5,6,13,14,15a-octahydropyrido[4',3':8,9][1,5]oxazonino[5,4-a]indole (10aa)

0.1 mmol scale. A white solid, 92% yield (49 mg). M. P. 190-192 ^{o}C. ^{1}H NMR ($CDCl_3$, 400 MHz, TMS) δ 1.41 (s, 3H), 2.39 (s, 3H), 2.75 (d, *J* = 12.4 Hz, 1H), 3.41 (d, *J* = 16.0 Hz, 1H), 3.85 (d, *J* = 16.0 Hz, 1H), 3.96 (d, *J* = 12.4 Hz, 1H), 4.25 (d, *J* = 14.8 Hz, 1H), 4.67-4.72 (s, 2H), 4.95 (d, *J* = 11.6 Hz, 1H), 5.04 (d, *J* = 14.8 Hz, 1H), 5.41 (s, 1H), 5.57 (s, 1H), 5.61 (d, *J* = 2.0 Hz, 1H), 6.29

(s, 1H), 7.03-7.13 (m, 2H), 7.20-7.23 (m, 1H), 7.26-7.29 (m, 5H), 7.40 (d, *J* = 7.6 Hz, 2H), 7.55 (m, 3H). ^{13}C NMR ($CDCl_3$, 125 MHz, TMS) δ 18.2, 21.6, 49.4, 52.5, 57.0, 68.2, 78.4, 101.6, 110.1, 119.6, 120.5, 121.5, 122.0, 123.4, 126.8, 127.2, 127.8, 128.1, 129.9, 130.7, 132.1, 135.2, 138.9, 139.1, 141.1, 142.5, 143.7, 144.0 cm^{-1}. IR (CH_2Cl_2) ν 3027, 2920, 2856, 2804, 2360, 2340, 1631, 1600, 1533, 1492, 1458, 1449, 1403, 1386, 1344, 1331, 1309, 1252, 1224, 1170, 1160, 1108, 1093, 1055, 1018, 977, 958, 935, 895, 860, 807, 788, 764, 757, 743, 706, 664 cm^{-1}. HRMS (ESI) calcd. for $C_{33}H_{33}N_2O_3S$ $(M+H)^+$: 537.2206, Found: 537.2200. $[\alpha]^{20}_D$ = +37.7 (c 1.00, CH_2Cl_2)]. Enantiomeric excess was determined by HPLC with a Chiralcel IC-H column [λ = 254 nm; eluent: Hexane/Isopropanol = 80/20; Flow rate: 0.50 mL/min; t_{minor} = 15.70 min, t_{major} = 18.88 min; ee% = 99%; $[\alpha]^{20}_D$ = 37.7 (c 1.00, CH_2Cl_2)].

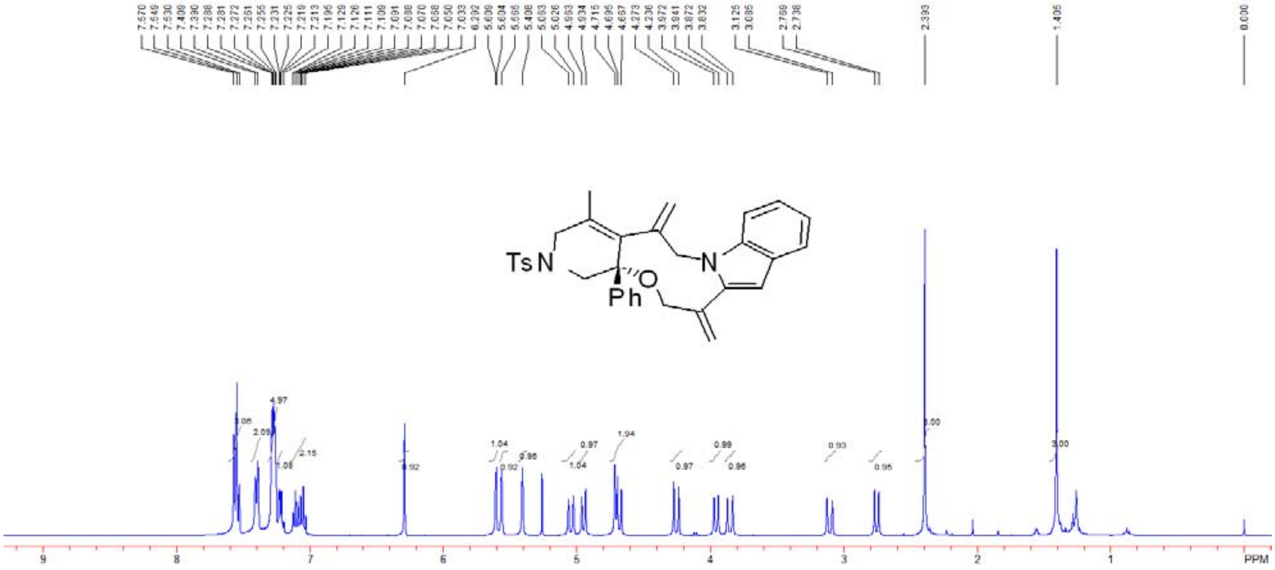

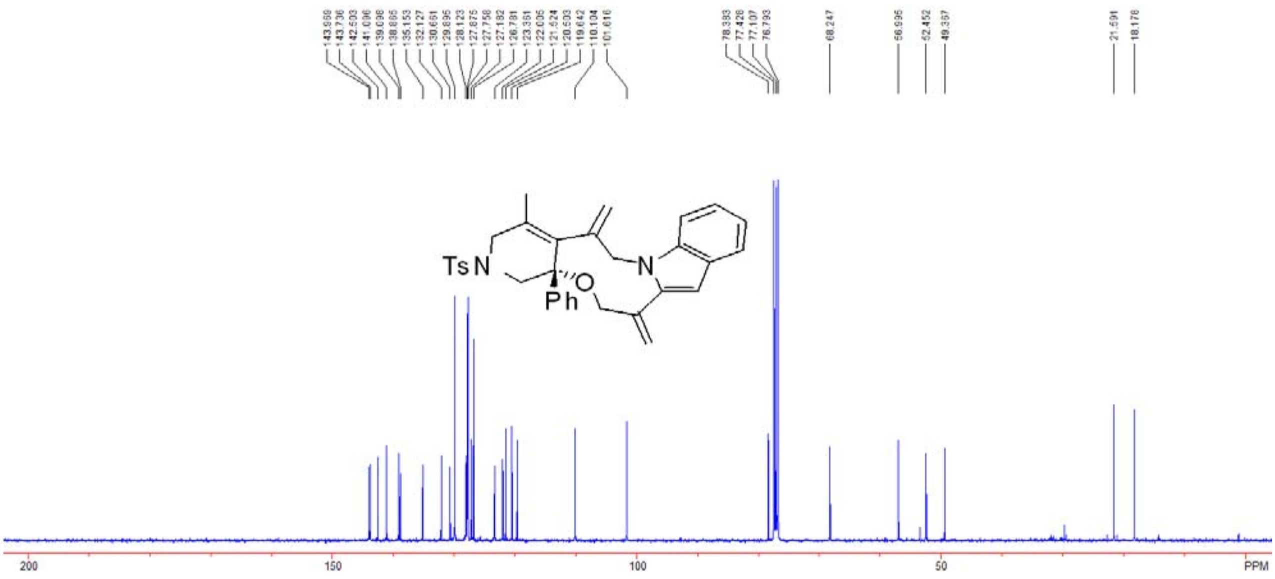

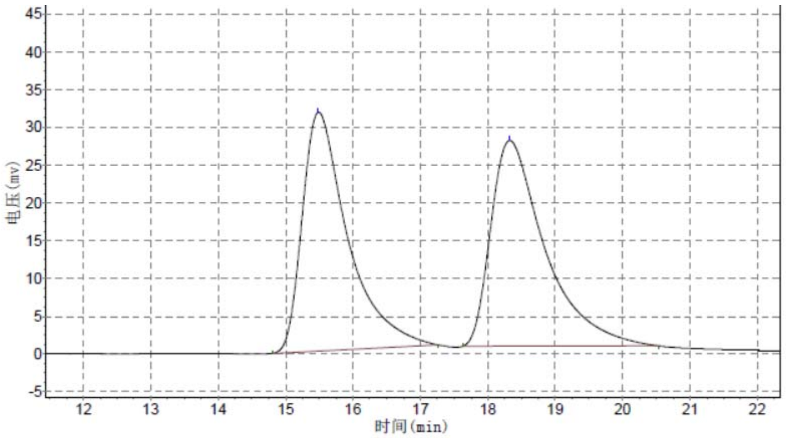

分析结果表

峰号	峰名	保留时间	峰高	峰面积	含量
1		15.488	31622.135	1481702.875	49.3646
2		18.322	27229.125	1519844.500	50.6354
总计			58851.260	3001547.375	100.0000

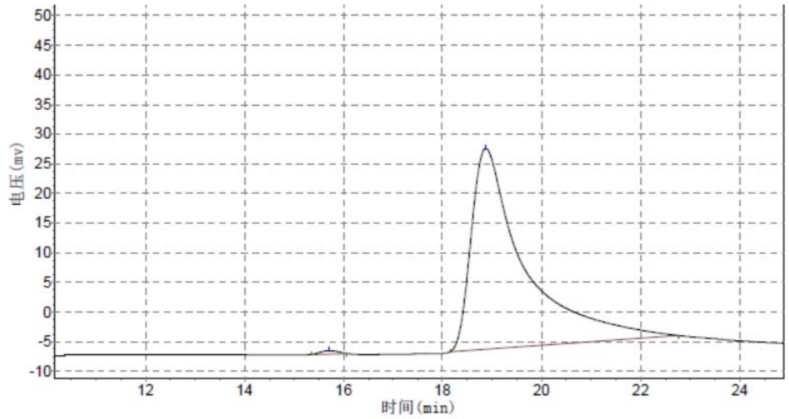

分析结果表

峰号	峰名	保留时间	峰高	峰面积	含量
1		15.698	569.161	13586.066	0.5330
2		18.877	33884.359	2535465.250	99.4670
总计			34453.521	2549051.316	100.0000

Translation: Chiralcel IC-H column [λ = 254 nm; eluent: Hexane/Isopropanol = 80/20; Flow rate: 0.5 mL/min; t_{minor} = 15.70 min, t_{major} = 18.88 min; ee% = 99%].

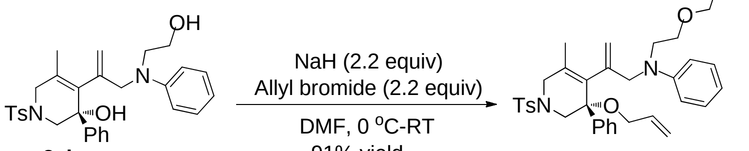

3ak

11ak, 73% yield

To a flame dried Schlenk tube was added compounds **3ak** (0.2 mmol), NaH (60% dispersion in mineral oil, 2.2 equiv) and DMF (2.0 mL). The reaction mixture was stirred at 0 °C for 0.5 h

before allyl bromide (2.2 equiv) was added. The reaction mixture was stirred at 0 °C for another 4 h. Then, the reaction mixture was diluted with cold water and was extracted with ether (4 mL x 3) and the combined organics was dried over anhydrous Na_2SO_4. The solvent was removed under reduced pressure and the residue was purified by a flash column chromatography (SiO_2) to give the corresponding product **11ak**.

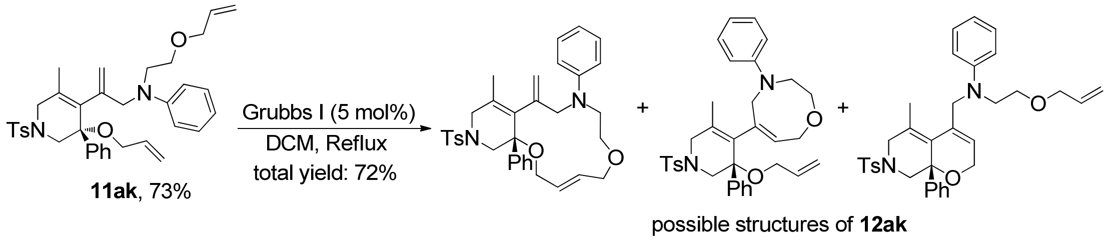

Under argon atmosphere, compounds **11ak** (0.1 mmo, 1.0 equiv), Grubbs I (0.50 equiv) CH_2Cl_2 (10 mL) were added and then the mixture was heated at 40-45 °C under refluxing for 12 h. Then, the solvent was removed under reduced pressure and the residue was purified by a flash column chromatography (SiO_2) to give the corresponding complex mixtures **12ak**. Disillusionary, the similar polarity of each components of **12ak** makes it difficult to get pure products.

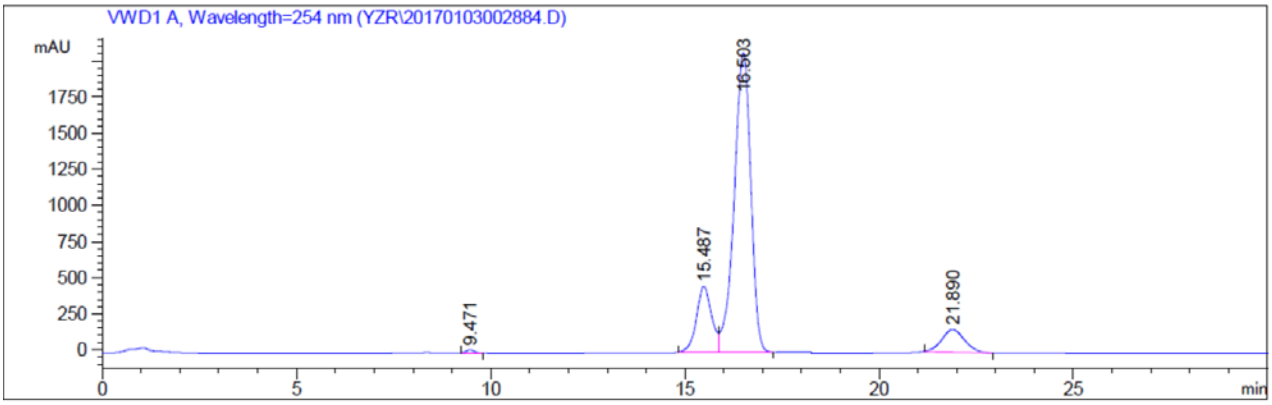

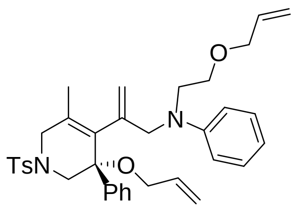

(S)-N-(2-(3-(allyloxy)-5-methyl-3-phenyl-1-tosyl-1,2,3,6-tetrahydropyridin-4-yl)allyl)-N-(2-(allyloxy)ethyl)aniline (11ak)

0.2 mmol scale. A white solid, 73% yield (87 mg). M. P. 70-72 °C. ^1H NMR ($CDCl_3$, 400 MHz, TMS) δ 1.89 (s, 3H), 2.43 (s, 3H), 2.99 (d, J = 12.4 Hz, 1H), 3.26-3.47 (m, 6H), 3.79 (d, J = 19.2

Hz, 1H), 3.87-3.97 (m, 4H), 4.29 (dd, J_1 = 4.8 Hz, J_2 = 12.8 Hz, 1H), 4.49 (s, 1H), 4.53 (dd, J_1 = 4.8 Hz, J_2 = 12.8 Hz, 1H), 4.77 (s, 1H), 5.14-5.18 (m, 2H), 5.23 (dd, J_1 = 1.2 Hz, J_2 = 17.2 Hz, 1H), 5.41 (dd, J_1 = 1.2 Hz, J_2 = 17.2 Hz, 1H), 5.81-5.91 (m, 1H), 5.97-6.06 (m, 1H), 6.24 (d, J = 8.0 Hz, 2H), 6.55 (dd, J_1 = 7.2 Hz, J_2 = 7.2 Hz, 1H), 7.02 (dd, J_1 = 7.6 Hz, J_2 = 8.0 Hz, 2H), 7.24-7.34 (m, 5H), 7.46 (d, J = 7.2 Hz, 2H), 7.64 (d, J = 8.0 Hz, 2H). ^{13}C NMR ($CDCl_3$, 125 MHz, TMS) δ 18.6, 21.5, 49.3, 50.0, 54.9, 55.4, 65.3, 67.5, 72.1, 77.8, 111.2, 115.5, 116.0, 118.8, 127.02, 127.28, 127.78, 127.84, 128.8, 129.8, 131.7, 131.9, 132.7, 134.7, 135.2, 139.3, 142.0, 143.9, 148.0. IR (CH_2Cl_2) ν 3062, 3021, 2966, 2926, 2871, 1598, 1506, 1486, 1447, 1401, 1348, 1289, 1258, 1167, 1108, 1091, 1049, 1022, 989, 956, 921, 811, 768, 746, 702, 669 cm^{-1}. HRMS (ESI) calcd. for $C_{36}H_{43}N_2O_4S$ (M+H)$^+$: 599.2938, Found: 599.2927. $[\alpha]^{20}_D$ = +39.4 (c 1.00, CH_2Cl_2)].

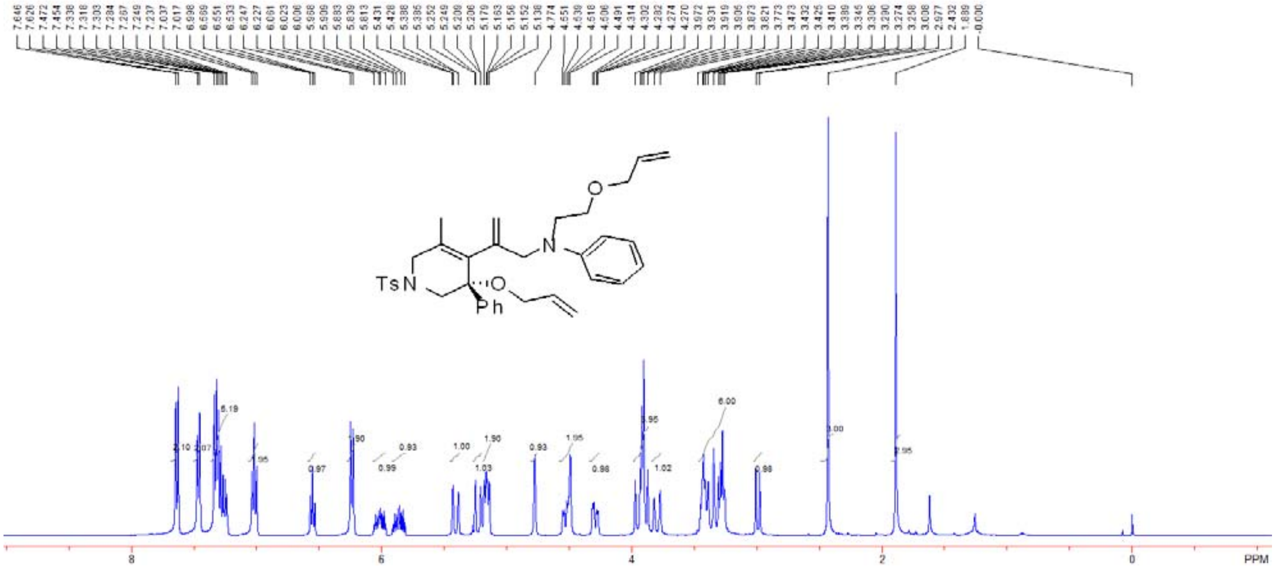

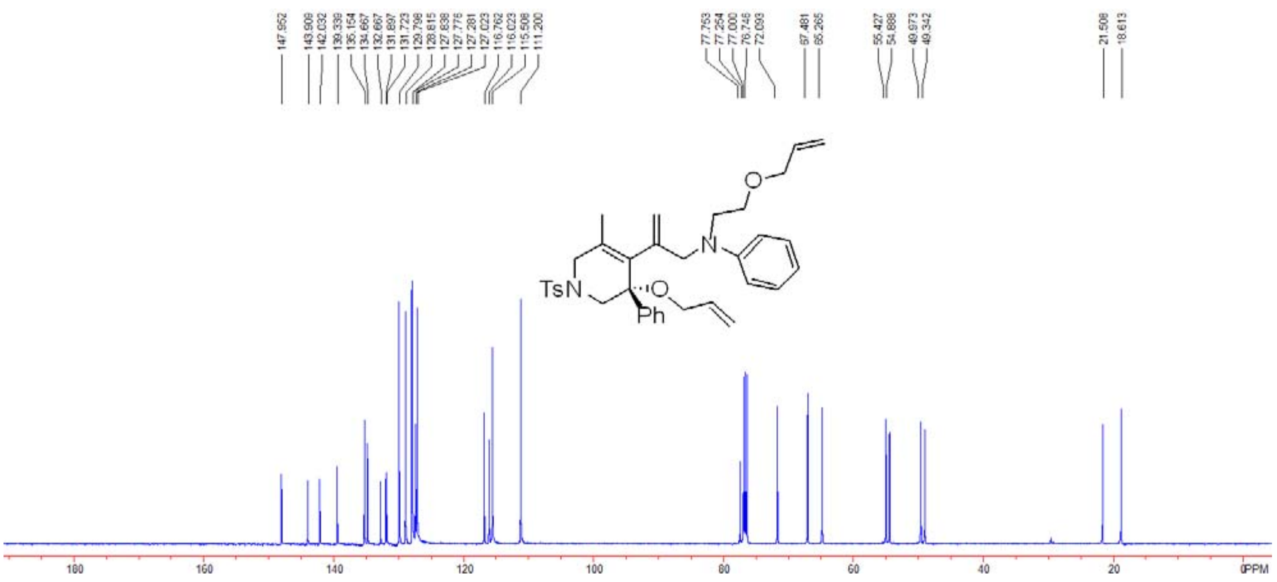

12. X-ray crystallographic information of products *Rac*-**3aa**, *Rac*-**5aa** and **10aa**

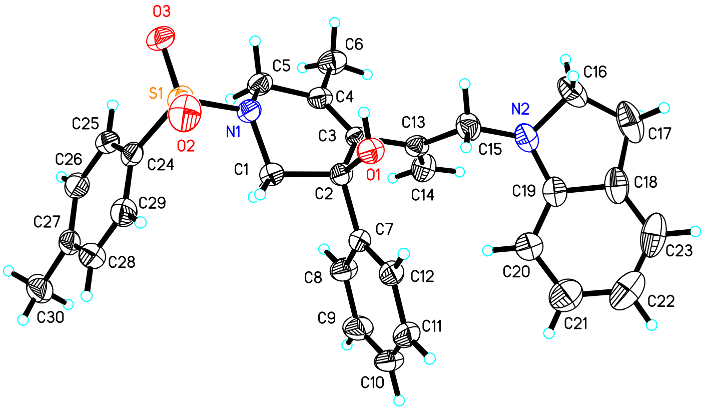

The crystal data of *Rac*-**3aa** have been deposited in CCDC with number 1538522. Empirical Formula: $C_{30}H_{32}N_2O_3S$; Formula Weight: 500.63; Crystal Color, Habit: colorless; Crystal Dimensions: 0.200 x 0.110 x 0.080 mm^3; Crystal System: Monoclinic; Lattice Parameters: a = 14.6758(17)Å, b = 8.1119(10)Å, c = 22.258(3)Å, α = 90°, β = 97.042(3)°, γ = 90°, V = 2629.8(5)Å^3; Space group: P 21/n; Z = 4; D_{calc} = 1.264 g/cm^3; F_{000} = 1064; Final R indices [I>2sigma(I)] R1 = 0.0517, wR2 = 0.1274.

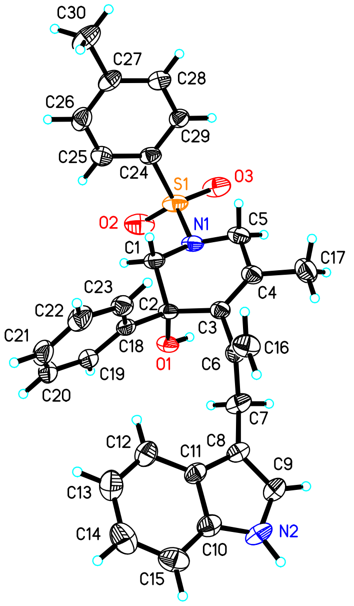

The crystal data of *Rac*-**5aa** have been deposited in CCDC with number 1525845. Empirical Formula: $C_{30}H_{30}N_2O_3S$; Formula Weight: 498.62; Crystal Color, Habit: colorless; Crystal Dimensions: 0.200 x 0.170 x 0.120 mm^3; Crystal System: Monoclinic; Lattice Parameters: a = 13.2526(17)Å, b = 8.5485(11)Å, c = 23.163(3)Å, α = 90°, β = 97.460(3)°, γ = 90°, V = 2602.0(6)Å^3; Space group: P 21/n; Z = 4; D_{calc} = 1.273 g/cm^3; F_{000} = 1056; Final R indices [I>2sigma(I)] R1 = 0.0488, wR2 = 0.1283.

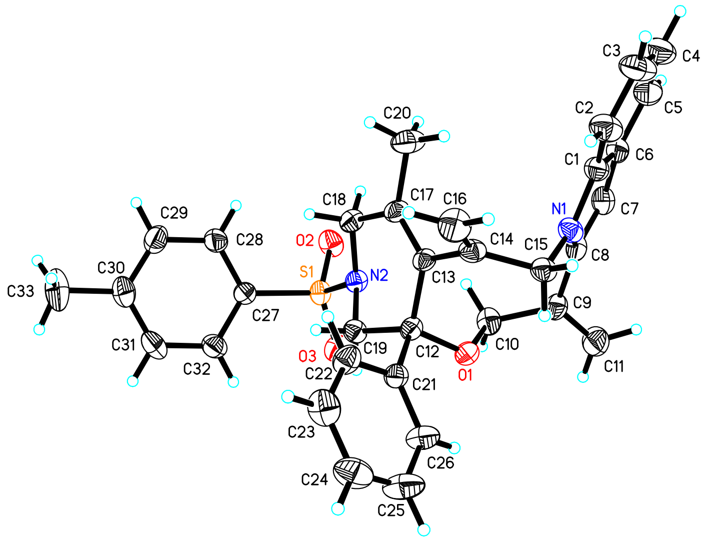

The crystal data of **10aa** have been deposited in CCDC with number 1822276. Empirical Formula: $C_{33}H_{32}N_2O_3S$; Formula Weight: 536.66; Crystal Color, Habit: colorless; Crystal Dimensions: 0.190 x 0.150 x 0.120 mm^3; Crystal System: Orthorhombic; Lattice Parameters: a = 9.5994(4)Å, b = 14.7775(6)Å, c = 19.9833(10)Å, α = 90°, β = 90°, γ = 90°, V = 2834.7(2)Å^3; Space group: P 21 21 21; Z = 4; D_{calc} = 1.257 g/cm^3; F_{000} = 1136; Final R indices [I>2sigma(I)] R1 = 0.0498, wR2 = 0.1071.

13. References

[1] S. Yang, K.-H. R, Q. Xu, M. Shi, M, *J. Am. Chem. Soc.* **2017**, *139*, 5957-5964.
